# An update on the mouse liver proteome

**DOI:** 10.1186/1477-5956-7-35

**Published:** 2009-09-08

**Authors:** Giuseppe Gazzana, Jürgen Borlak

**Affiliations:** 1 Department of Molecular Medicine and Medical Biotechnology, Fraunhofer Institute of Toxicology and Experimental Medicine (ITEM), Hannover, Germany; 2 Center for Pharmacology and Toxicology, Hannover Medical School, Hannover, Germany

## Abstract

**Background:**

Decoding of the liver proteome is subject of intense research, but hampered by methodological constraints. We recently developed an improved protocol for studying rat liver proteins based on 2-DE-MALDI-TOF-MS peptide mass finger printing.

This methodology was now applied to develop a mouse liver protein database.

**Results:**

Liver proteins were extracted by two different lysis buffers in sequence followed by a liquid-phase IEF pre-fractionation and separation of proteins by 2 DE at two different pH ranges, notably 5-8 and 7-10. Based on 9600 in gel digests a total of 643 mouse liver proteins with high sequence coverage (> 20 peptides per protein) could be identified by MALDI-TOF-MS peptide mass finger printing. Notably, 255 proteins are novel and have not been reported so far by conventional two-dimensional electrophoresis proteome mapping. Additionally, the results of the present findings for mouse liver were compared to published data of the rat proteome to compile as many proteins as possible in a rodent liver database.

**Conclusion:**

Based on 2-DE MALDI-TOF-MS a significantly improved proteome map of mouse liver was obtained. We discuss some prominent members of newly identified proteins for a better understanding of liver biology.

## Background

Life is incompatible without the liver as this organ performs essential metabolic functions. Estimates suggest an excess of > 10,000 biochemical reactions at any given time point, and this includes basic carbohydrate, fat and protein metabolism; storage of vitamins and minerals; many regulatory functions that control blood sugar and hormone levels. Indeed, the liver is the primary organ for the synthesis of many different proteins, such as plasma albumin, fibrinogen and most globulins, as well as lipids and lipoproteins (phospholipids, cholesterol) and is responsible for bile acid production and excretion. Being exposed to a wide range of xenobiotics and toxins the liver has the remarkable capacity for regenerative growth. In fact, up to 75% of the liver can be surgically removed, before it ceases to function [1], but will return to its original size through regenerative growth within a few weeks.

It is of considerable importance that the human and mouse genome display > 99% DNA sequence similarity. Consequently, this laboratory animal is widely used in biomedical research, for instance, in the evaluation of novel experimental therapeutics. Despite the genetic similarity, their proteomes differ considerably. Therefore, translating findings from mice to humans requires an understanding of the differences in regard to physiology, pathology and response to toxicants [2]. In the past, several investigators attempted to identify mouse [3-5] and rat [6-8] liver proteins by use of the 2-D PAGE technique, aiming to build up databases of rodent proteomes [9]. As early as 1981, Klose and Feller investigated the mouse liver proteome by 2-DE PAGE [3]. At present, several databases of mouse liver [10-13] and of subcellular fractions such as mitochondria [14], microsomes [15], peroxisomes [16] and nuclear proteome [17] are available. For instance, Fountoulakis et al. [11] reported a total of 256 unique mouse liver proteins in their efforts to identify proteins regulated by the analgesic drug acetaminophen and to explore possible mechanisms of hepatotoxicity. Furthermore, 328 unique proteins (cytosolic and mitochondrial/microsomal proteins) were reported in a subsequent publication to increase knowledge on the mouse liver proteome [12]. In this regard, 107 mouse liver proteins were identified by Sanchez et al. [13] by 2-DE PAGE followed by in gel tryptic digest and mass spectrometry. Finally, a total of 182 unique proteins were reported by Da Cruz et al. [14] and traced back to the mouse liver mitochondrial inner membrane.

Here, we report our efforts to map the proteome of mouse liver. In total, we identified 643 proteins and some isoforms. Indeed, 255 proteins identified by us have not been reported so far by two-dimensional electrophoresis proteome mapping and when compared with published databases such as the one published by Fountoulakis et al. [11,12] (see also ). Specifically, we applied our recently published protocol to map liver proteins by use of a combination of different fractionation methods (a liquid-phase IEF pre-fractionation, the use of two different pH ranges, 5-8 and 7-10 and two different lysis buffers in sequence) in order to reduce the complexity of the protein extract and to increase the probability of detecting low-abundance proteins.

Overall, we aimed at developing a proteome map and to make this database publicly available to assist researchers in their studies on liver biology.

## Materials and methods

### Materials

A UP 200S Sonicator (Dr. Hielscher GmbH, Stuttgart, Germany) was used to homogenate the samples. For the first dimension, immobilized pH gradient (IPG) strips (17 cm, pH 5-8 and 7-10) were purchased from Bio-Rad (Hercules, CA, USA). The pre-fractionation was carried out with a Rotofor Cell (Bio-Rad). The focusing chamber was a Protean Isoelectric Focusing (IEF) Cell (Bio-Rad). For the second dimension a Protean plus Dodeca Cell (Bio-Rad) was used.

Reagents (tris, urea, thiourea, CHAPS, dithiothreitol, bromophenol blue, glycerin, sodium dodecyl sulfate, glycin, temed, ammonium peroxodisulfate, ammonium sulfate, ammonium bicarbonate, colloidal coomassie blue and acrylamide) were purchased from Roth (Karlsruhe, Germany). Iodacetamide was from SERVA (Heidelberg, Germany). Benzonase was purchased from Novagen (Darmstadt, Germany). Ampholytes (biolyte 3-10) were purchased from Bio-Rad. DeStreak was purchased from Amersham Bioscience (Freiburg, Germany).

### Animal care

A total of n = 6 C57/Bl6 male mice (aged 6-8 months) weighing 25-33 g were housed in Makrolon^® ^Type III cages. Drinking water and food (V1124-000, SSNIFF, The Netherlands) were given *ad libitum*. The temperature and relative humidity were 22 ± 2°C and 40-70%, respectively. Furthermore, a 12-h day and night cycle was used. For liver explantation, mice were anesthetized with Ketamin 10% 100 μL/100 g and Xylazin 2% 50 μL/100 g, and after surgical removal the liver was washed until free of blood.

### Mouse liver sample preparation

**Figure 1:**
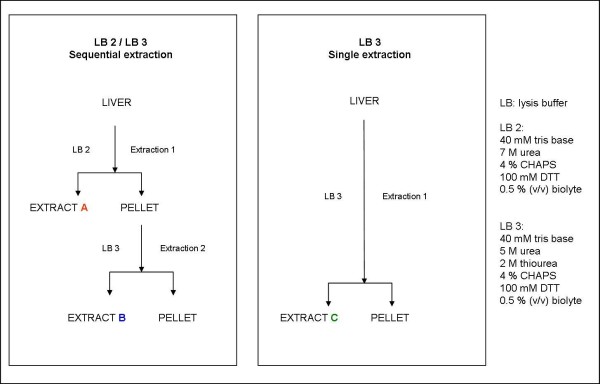
**Outline of protein extraction procedures**. A) sequential extraction with LB2 (7 M urea) and LB3 (5 M urea, 2 M thiourea). B) direct extraction with LB3 (5 M urea, 2 M thiourea).

Approximately 0.1 g of the liver sample was ground in a mortar under liquid nitrogen flow. Then, the samples were processed with 0.5 mL of a buffer containing 40 mM tris base, 7 M urea, 4% CHAPS, 100 mM DTT and 0.5% (v/v) biolyte 3-10 first (LB2). The suspensions were homogenized by sonication (3 × 20 s) and after addition of 3 μL of benzonase (endonuclease that degrades DNA and RNA) were incubated at room temperature for 20 min. The samples were then centrifuged at 12,000 g for 20 min. The pellets were washed and sonicated for 5 min with a further 0.5 mL of LB2 and centrifuged at 12,000 g for another 20 min, and the resulting two fractions of supernatant were collected (extract A) (Figure 1). Finally, the pellets were dissolved with 0.5 mL of buffer containing 40 mM tris base, 5 M urea, 2 M thiourea, 4% CHAPS, 100 mM DTT, 0.5% (v/v) biolyte 3-10 (LB3), sonicated and centrifuged at 12,000 g for 20 min. The pellet was collected, and the supernatant was marked as extract B (Figure 1).

From the same animals, a further 0.1-g portion was ground in a mortar, but treated this time with 0.5 mL of LB3. The suspensions were sonicated, incubated with benzonase and centrifuged. The pellets were then washed with another 0.5 mL of LB3, sonicated and centrifuged, and the supernatants were collected (extract C) (Figure 1).

**Figure 2:**
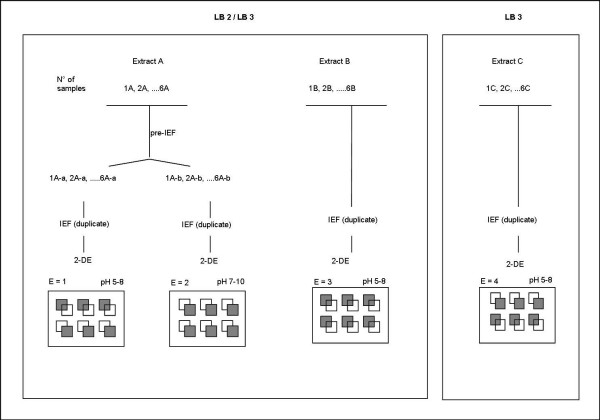
**Proteome mapping of mouse liver**. A pre-fractionation step prior to the IEF was carried out for the samples 1A-6A to separate proteins in two fractions: acid proteins with pH values < 7 (1A-a; 6A-a) and basic proteins having pH values > 7 (1A-b; 6A-b). All fractions were run in duplicate modus for the first and second dimensions. In summary, three experiments (E = 1, E = 3, E = 4) were repeated for the samples in the pH range 5-8. One experiment (E = 2) was carried out using the pH range 7-10. A total of 24 gels (grey small boxes) were used for cutting, digestion and MS identification of the spots/proteins. (N° = number; E = experiment; A-a = extract in LB2, acidic fraction; A-b = extract in LB3, basic fraction).

Proteome mapping was done under a variety of conditions, e.g. extraction with lysis buffers 2 and 3. In addition, proteins were separated at two different pH ranges (5-8 and 7-10). A total of 4 experiments were carried out, and duplicate measurements were run for each experiment (Figure 2). Overall approximately 9600 spots, cut from 24 gels, could thus be investigated. The protein concentration of all extracts was determined using the Bradford assay.

### Liquid-phase IEF pre-fractionation

The liquid-phase IEF pre-fractionation was performed in the Rotofor Cell system (Bio-Rad) following the instructions of the supplier. Ion exchange membranes were equilibrated overnight in the appropriate electrolyte (anion exchange membranes in NaOH 0.1 M and cation exchange membranes in H_3_PO_4 _0.1 M). After four runs, ion exchange membranes were always discarded and replaced by new membranes for the other samples. For each run, the electrode chambers were filled with fresh appropriate electrolytes (30 mL). Initially, the cell was filled with pure water and run for 5 min at 5 W constant power to remove residual ionic contaminants from the membrane core and ion exchange membranes. Approximately 32 mL of LB2 were used to fill the cell. A total of 60 mg of total proteins in approximately 2 mL of LB2 were added to the cell to reach the maximum loadable volume (40 mL). Focusing started at 12 W constant power. After approximately 4 h, the voltage increased to 3000 V and the wattage decreased to 3 W. The focused proteins were harvested in 20 ~ 1.5 mL fractions, and pH values were checked. Fractions having pH values between 3 and 7.0 were collected and denoted as "A-a" (acid) (Figure 2). Fractions having pH values > 7.0 were collected and denoted as "A-b" (basic) (Figure 2). Again the protein concentration was determined for both fractions (A-a and A-b) by the Bradford method. Approximately 30 mg of protein were recovered at the end of the liquid-phase IEF pre-fractionation from an initial 60 mg load. The losses are accounted for by the multi-step pre-fractionation procedure, but are not the result of a precipitate that could not be dissolved in our lysis buffer. Other investigations have reported similar losses during pre-fractionation; for instance, see Fountoulakis *et al*. [18], or P.G. Righetti *et al*. [19]. After each run, the membrane core was cleaned with NaOH 0.1 M overnight and sonicated for 5 min in water before the new focusing.

### Two-dimensional gel electrophoresis

#### Isoelectric focusing (IEF) - first dimension

IEF was performed using precast linear IPG strips. The 17-cm IPG strips 7-10 and 5-8 were loaded with 1.5 mg of proteins by active rehydration (12 h, 50 V). Samples destined to be separated by IPG strips 7-10 received an excess of hydroxyethyl disulfide (HED) (DeStreak™) prior to the focusing run. Focusing began at 250 V for 20 min in rapid mode, increasing to 10,000 V for 5 h in linear mode and 10,000 V for 50,000 Vh in rapid mode (for the IPG strips 5-8). IEF for the strips 7-10 was carried out at 250 V for 60 min in rapid mode, then at 10,000 V for 3 h in linear mode and at 10,000 V for 50,000 Vh in rapid mode. Each sample was analyzed in duplicate (Figure 2).

#### 2-DE - second dimension

After IEF, the IPG strips were either stored at -80°C or transferred to 10 mL of equilibration buffer (6 M urea, 30% w/v glycerin, 2% w/v SDS, 50 mM Tris-HCl pH 8.8) with 2% w/v DTT and 0.5% v/v bromophenol blue solution (0.25% w/v bromophenol blue, 1.5 M Tris-HCl pH 8.8, 0.4% w/v SDS) and incubated for 20 min at room temperature. Strips were removed and incubated in equilibration buffer with 4% w/v iodoacetamide and 0.5% v/v bromophenol blue solution for further 20 min at room temperature. Finally, the strips and 10 μL SDS-PAGE molecular weight standard on filter paper were placed on top of the 20 cm × 20.5 cm 12% second-dimension gel (12% v/v acrylamide/bis solution, 375 mM Tris, pH 8.8, 0.1% v/v SDS, 1/2000 TEMED, 0.05% v/v APS). Both were fixed in place with a 0.5% w/v agarose overlay. Gels were run in PROTEAN Plus Dodeca cell from Bio-Rad at 70 V for approximately 14 h, followed by 200 V until the bromophenol blue dye reached the bottom of the gel. The running buffer (25 mM Tris, 0.2 M glycin, 0.1% SDS) was cooled externally to 16°C.

Gels/proteins were fixed overnight in 30% ethanol and 2% phosphoric acid and washed 3 × 20 min with 2% phosphoric acid. The gels were equilibrated with 15% ammonium sulfate, 18% ethanol and 2% phosphoric acid for 15 min and finally stained with colloidal coomassie blue for 48 h.

#### Gel scanning and image analysis

After staining, gels were washed 10 min with pure water and scanned on a Molecular FX Scanner Bio-Rad at 100 μm resolution. Protein spots were imaged first automatically and then manually and analyzed using the PDQuest™ software Bio-Rad. The normalization was carried out in total density in gel mode according to the manufacturer's recommendation.

### Matrix-assisted laser desorption ionization mass spectrometry (MALDI-MS)

A total of 9600 spots derived from 24 gels were excised using the spot cutter of Bio-Rad and placed into 96-well microtiter plates. Excised gel spots were washed manually with 20 μL of water for 10 min and destained twice, first with 15 μL ammonium bicarbonate 50 mM for 5 min and then with 15 μL 50% ammonium bicarbonate 50 mM - 50% acetonitrile for 5 min. Finally, the gel particles were covered by acetonitrile until gel pieces shrunk and left to dry for 10 min. All gels/proteins were digested manually in situ with 4 μL of ammonium bicarbonate 50 mM containing 20 ng trypsin (Sequencing Grade Modified Trypsin Promega). After 15 min, each gel piece was re-swelled with 10 μL of ammonium bicarbonate 50 mM and incubated for 4 h at 37°C. After 4 h, the reaction was stopped by adding 10 μL of trifluoroacetic acid 1% containing 1.5% (w/v) n-octyl-β-D-glucopyranoside (OGP) (AppliChem). For the application of the samples, 4 μL of peptide solution were loaded on an MTP Anchor Chip Target 600/384 (Bruker Daltonics) previously prepared with a saturated solution of matrix, α-cyano-4-hydroxy-cinnamic acid (α-HCCA) (Bruker Daltonics). An external calibration was performed by spotting on the 96 calibration positions of the Anchor Chip Target 1 μL of the peptide calibration standards (Bruker Daltonics) containing the following peptides: angiotensin II (1046.5420 Da), angiotensin I (1296.6853 Da), substance P (1347.7361 Da), bombesin (1619.8230 Da), ACTH clip 1-17 (2093.0868 Da), ACTH clip 18-39 (2465.1990 Da), somatostatin 28 (3147.4714 Da) and OGP 1.5% (w/v). Samples were analyzed in a MALDI-TOF-TOF spectrometer (Ultraflex, Bruker Daltonics) using an accelerating voltage of 25 kV for the Peptide Mass Fingerprint (PMF) mode. When necessary, MALDI-Post Source Decay (PSD) analysis was carried out using the LIFT special technique delivered by Bruker (the basic idea of LIFT is to lift the potential to fragment the selected peptides of interest). Peptide matching and protein searches were performed automatically with the MASCOT software. For the PMF search the parameters were the following: C-carbaimidomethyl (fixed modification), M-oxidation (variable modification), monoisotopic (mass value), 100 ppm (peptide mass tolerance), 1 (max missed cleavage), mammalia (taxonomy). Five matching peptides and at least 10% peptide coverage of the theoretical sequences was the minimal requirement for an identity assignment. For the MS/MS search (PSD) the parameters were the same except the peptide mass tolerance, which was 200 ppm. The identified proteins were organized with the ProteinScape™ database (Bruker Daltonics) and checked individually and only mouse proteins or highly homologous sequences from other mammalian species, like *Homo sapiens *or *Rattus Norvegicus*, having pI and M*w *values close to the theoretical, were considered (a total of n = 39 proteins).

### Optimization of the IEF for proteins in the basic pH range

Isoelectric focusing of proteins in the acidic and basic pH ranges is often associated with streaking, due to disappearance of the reducing agent, normally DTT, from the basic part of the IPG strip, followed by oxidation of the protein thiol groups, resulting in a heterogeneous mixture of inter and intra chain -S-S- bonds and causing a train of extra artifactual spots.

To decrease the streaking of basic proteins we tested: a) different IEF programs, b) the use of an electrode paper pad at the cathode as a source of DTT during focusing and c) the use of hydroxyethyl disulfide (DeStreak™).

## Results

### Protein extraction and separation

We employed two strategies to extract mouse liver proteins: a) sequential extraction with lysis buffers LB2 and LB3; b) extraction with LB3 only (Figure 1). For some proteins the use of two lysis buffers in sequence improved considerably the number of detectable and identifiable spots as reported herein.

**Figure 3:**
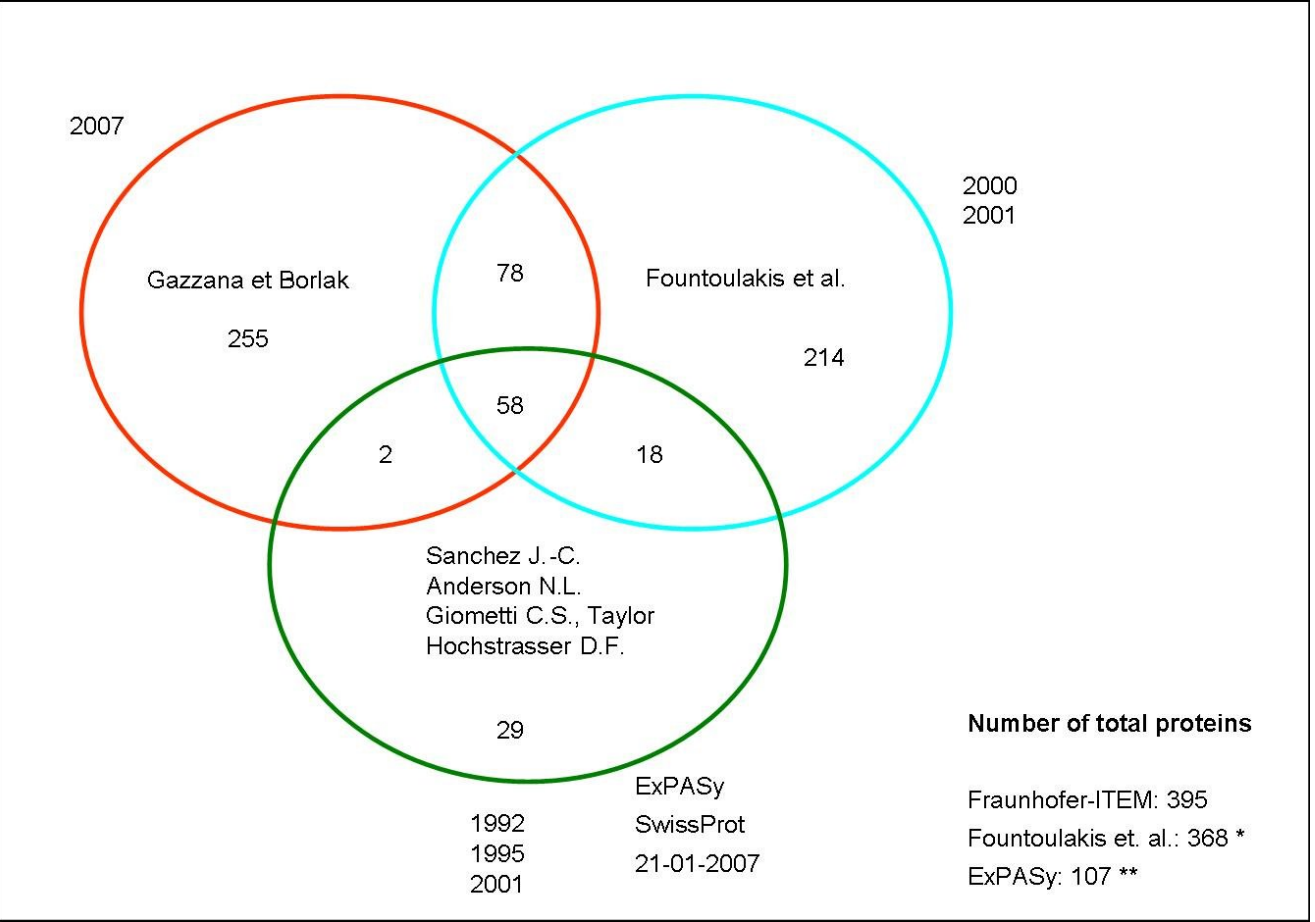
**Comparison of three mouse liver proteomes**. Our map of the mouse liver proteome comprises 390 proteins (643 including the isoforms), 255 of which were not previously identified by others. * The number ofproteins was obtained by adding the data of two articles [11,12]. ** The proteins are freely available at .

We optimized the separation of proteins in the basic pH range during the IEF [8]. We analyzed mouse liver protein extracts on two narrow pH range IPG strips (pH 5-8 and pH 7-10) (Figure 2) and processed 24 2-DE gels to yield approximately 9600 gel digests. Of the approximately 9600 spots studied many were redundant proteins. Taken collectively, a total of 643 unique proteins were identified, including several isoforms. To the best of our knowledge, 255 proteins have not been reported so far in previous 2-DE liver proteome maps (Figure 3, Additional File 1).

We also investigated the sensitivity, linear range and compatibility of stain for identification by mass spectrometry [8] and used a liquid-phase IEF pre-fractionation in order to reduce the complexity of the samples and to enrich for low-abundance proteins [8]. The additional files 2 and 3 depict two gels each for the pH ranges 5-8 and 7-10.

### Peptide mass fingerprinting

After in gel tryptic digest the proteins were analyzed by MALDI-TOF/TOF (Ultraflex, Bruker Daltonic). Identification of proteins was carried out by database searches with MASCOT. A total of 643 coomassie-stained proteins were identified and could be traced back to 390 different gene products. Not all isoforms could be identified, because in some cases the identified peptides were identical in sequence (see Additional file 4 where sequences of all peptide mass fingerprints are given). Additional file 5 gives additional information, e.g. the names of the identified proteins, accession numbers in SwissProt, gene names, functions and biological processes. Furthermore, we provide information on the lysis buffer used, how frequently the protein was identified, how many spots for each protein were observed, the subcellular location and which of the proteins are in common between mouse and rat liver. In Additional file 4 we report: the molecular weight, theoretical pI, MASCOT score, together with sequence coverage (%), the number of identified peptides and the sequence of peptides. Notably, on average 20 peptides per protein could be identified giving rise to a high sequence coverage.

### Subcellular location

**Figure 4:**
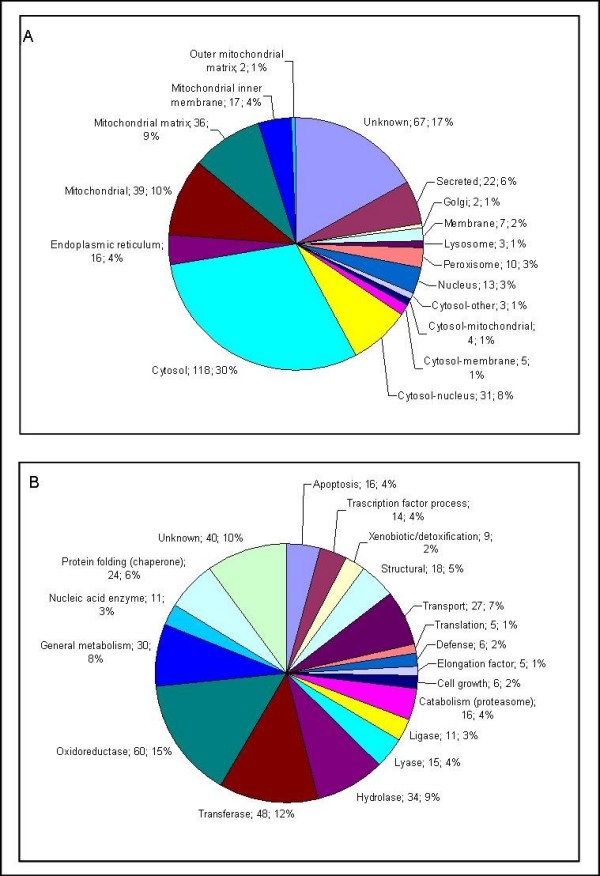
**A: Subcellular location of identified mouse liver proteins**. The Swiss-Prot and Gene Ontology databases were used for the protein annotation. **B**: Biological function of identified mouse liver proteins. The proteins were classified into 19 functional groups. The metabolism group was subdivided into 8 subgroups, such as oxidoreductase, transferase etc. The Swiss-Prot and Gene Ontology databases were used for the protein annotation.

The identified proteins were assigned to various subcellular compartments of the cell, e.g. the cytoplasm, mitochondria, endoplasmic reticulum (ER) and the nucleus. For about 83% of the proteins in the Additional file 5, entries in the Swiss-Prot or Gene Ontology (GO) databases were found. The distribution of the identified proteins according to cell compartments is depicted in Figure 4A. Notably, most of the proteins are cytosolic or mitochondrial (118 and 39, respectively). For a total of 67 (17%) proteins no subcellular locations are known. It is of considerable importance that after extraction with LB2 78 proteins could be identified and were of cytosolic, mitochondrial, or mitochondrial matrix origin (see Additional file 5 column "lysis buffer"). These proteins were neither found in extract B nor C.

### Biological function

The identified proteins were categorized according to their biological function (Figure 4B). For approximately 90% of the proteins at least one annotation term within the Gene Ontology (GO) was found. About 214 proteins (54%) are involved in metabolism and code, for example, for oxidoreductase, transferases, hydrolases, lyase and ligases. A total of 16 proteins were identified with inferred functions in apoptosis. Among them was the 78 kDa glucose-regulated protein, which plays a role in facilitating the assembly of multimeric protein complexes inside the ER (Swiss-Prot: P07823). A total of 24 chaperone proteins, involved in protein folding, were identified, including a 170 kDa glucose-regulated protein which delivers copper to zinc superoxide dismutase.

Further proteins with different biological functions were identified, including structural proteins (5%), transport proteins (7%), nucleic acid enzymes (3%) and proteins (4%) involved in catabolic processes, like the proteasome machinery. We also identified 14 proteins (4%) involved in transcription, such as the similar to transcriptional activator protein pur-alpha (purine-rich single-stranded DNA-binding protein alpha) and 9 (2%) xenobiotic/detoxification proteins (Figure 4B). Finally, 40 (10%) proteins had no entries in GO concerning their biological function.

## Discussion

Because of the liver's tremendous importance in basic metabolism and its frequent injury by drugs and chemicals, research on the liver proteome has received much attention. As of today, several reports on mouse liver proteomes are available, but differ with regard to number and abundance of reported proteins [2-17]. Here, we compared our mouse liver proteome map with those reported by others, thereby evidencing which proteins are novel. To the best of our knowledge, a total of 368 proteins have so far been reported for mouse liver based on 2-DE/MS [11,12]. We report further insight into the mouse liver proteome by employing a combination of different lysis buffers, a pre-fractionation method of proteins prior to the first dimension and the use of two different pH gradients to map as many proteins as possible from mouse liver extracts. As described in our previous work, the use of thiourea in addition to urea improved solubility, especially of membrane proteins.

To separate complex protein mixtures, additional dissolving power is needed. The use of narrow-range IPGs (e.g. pH 5-8 and 7-10) allowed us to further reduce the complexity of the protein extract and enabled visualization of a greater number of protein spots. The pH range 3-10 was not used in this study, because we already demonstrated its limited advantage in our previous work [8]. Most of the proteins have a pI in the pH range of 5-8. Here, we report 643 proteins, 255 of which have not been reported so far by two-dimensional electrophoresis proteome mapping, when compared with published findings. These proteins are listed in Additional file 1 and are grouped according to their biological processes. Some interesting examples are discussed below.

We identified proteins involved in programmed cell death [20,21]. This process is tightly controlled and involves the coding of death factors and death receptors. Apoptosis is frequently disabled in tumour growth to foster cell proliferation. Specifically, we identified the eukaryotic initiation factor 5A (Swiss-Prot: P63242). This nucleocytoplasmic shuttle protein is involved in cell proliferation and apoptosis [22]. Overexpression of eIF5A, a protein known to undergo posttranslational modification by deoxyhypusine synthase, induced apoptosis in colon carcinoma cells and was shown to be required for expression of p53 following an induction of apoptosis upon treatment of the cells with actinomycin D. Unhypusinated eIF5A may have pro-apoptotic functions and is rapidly translocated to the nucleus following the induction of apoptotic cell death [22].

A further example of the newly identified mouse liver protein is the hepatoma-derived growth factor (Swiss-Prot: P51859), e.g. a heparin-binding protein with mitogenic activity in fibroblasts. This protein plays an important role in liver development and regeneration as well as hepatocarcinogenesis [23].

Among the **transcription factor **proteins identified, we report the similar to transcriptional activator protein pur-alpha (Swiss-Prot: P42669) as an example. Probably, this transcription activator binds the purine-rich single strand of the PUR element located upstream of the c-Myc gene. It may play a role in the initiation of DNA replication, in recombination and may even be involved in apoptosis, cell differentiation, cell proliferation, negative regulation of transcription, positive regulation of cell proliferation and regulation of transcription [24].

Furthermore, we identified several **xenobiotic **defense proteins, examples of which are aldehyde dehydrogenase 1 family, member B1(Q9CZS1), biphenyl hydrolase-like (Q8R164), esterase D/formylglutathione hydrolase (Q9R0P3) and aldo-keto reductase family 1, members C12 and C13 (Q91X42, Q8VC28). All of these proteins play a role in detoxification of alcohol-derived acetaldehyde, corticosteroids, biogenic amines, neurotransmitters and lipid peroxidation products [25-28].

Moreover, we identified more than 10 proteins involved in protein folding. Examples are heat shock protein gp96 (Q29092), trap1 protein (Q922Z3) and the 170 kDa glucose-regulated protein (Q60432).

**Figure 5:**
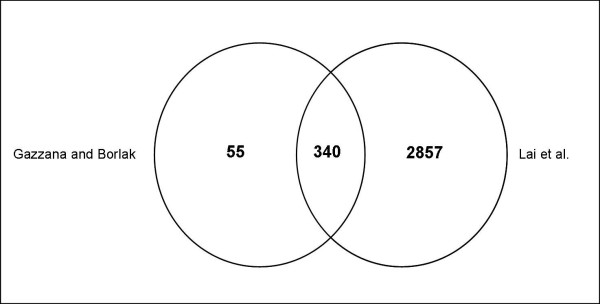
**VENN diagram of identified proteins reported by Lai et al**. [29]**and the present study**.

Notably, in a most recent study of Lai et al. [29], a comprehensive and quantitative proteome map of the mouse liver and plasma was reported [29]. The authors compiled 7099 proteins based on an identification of two peptides per protein. This extraordinary high number of proteins was, however, reduced to 2857, when 4 peptides were determined for their identification. In our efforts we obtained on average a sequence coverage of 20 peptides or more to yield 643 unique proteins including some isoforms. Based on our experimental strategy > 50 novel proteins could be identified that were not reported by Lai et al. (Figure 5) [Additional file 6]. This was surprising as many are of high abundance. Importantly, these proteins are of great importance in liver biology and regulation of some of these proteins in disease is well documented. For instance, we uniquely identified heat shock protein 90 (Hsp90) that has become a target for therapy in diverse human malignancies [30]. Another protein identified by us is cofilin-1 that was found to be down-regulated in liver cancer [31]. This protein controls reversibly actin polymerization and depolymerization in a pH-sensitive manner and it is the major component of intranuclear and cytoplasmic actin rods. In an attempt to identify biomarkers that could distinguish cancerous and non malignant liver tissues, Lee and co-workers developed a classifier model based on the expression of six biomarkers that included cathepsin B and cytochrome b5. Once again these proteins were not reported in the study of Lai et al but were clearly identified in our approach. Notably, these biomarkers were significantly associated with serum AFP levels in clinical cases of HCC [32]. Others not reported by Lai et al but identified in the present study are catalase, glutathione synthetase, ferritin light chain, peroxiredoxin-1, major urinary protein 1, all of which are abundant proteins. Presumably, the sample work flow in the study of Lai et al and probably low efficiencies in the ionisation of these proteins may hamper their identification. Overall, our study highlights the need to confirm independently the growing database for liver proteins. Apart from the uniquely identified proteins there was good agreement between the data reported by Lai et al [29] and the one reported herein.

## Conclusion

In conclusion, we aimed at an identification of as many proteins as possible to further map the mouse liver proteome by using two different lysis buffers, a pre-fractionation method prior to the IEF and the application of two different IPG strips. The proteins were identified by MALDI-TOF/TOF mass spectrometry after in gel digests of approximately 9600 spots, derived from 24 gels. The protocol described herein is not limited to the mouse liver and may well be applied to other tissues. Our efforts to decode the mouse liver proteome resulted in an identification of 643 proteins, and they contribute to an improved understanding of liver biology.

## Conflict of interest statement

The authors declare that they have no competing interests.

## Authors' contributions

JB initiated the study and was responsible for the experimental design. GG carried out the studies as part of his PhD studies. Both authors analysed the data, drafted the manuscript, read and approved the final manuscript.

## Supplementary Material

Additional file 1 **Table 1**. List of the 255 proteins identified from mouse liver extracts. Proteins were identified by MALDI-TOF MS and are grouped into 19 different biological processes. Click here for file

Additional file 2 **2-DE Gel, pH 5-8**. Representative 2-D gel: pH 5-8 (loading sample 1.5 mg; stain: coomassie blue; 17-cm IPG strips, BioRad). Click here for file

Additional file 3 **2-DE Gel, pH 7-10**. Representative 2-D gel: pH 7-10 (loading sample 1.5 mg; stain: coomassie blue; 17-cm IPG strips, BioRad). Click here for file

Additional file 4 **Table 2**. In the Table 2, the protein name, accession number, theoretical Mr and pI are reported together with MASCOT score, percentage of peptide coverage, number of identified peptides and sequence. Five matching peptides (see column "Pep") and at least 10% peptide coverage of the theoretical sequences was the minimal requirement for an identity assignment (see column "%"). Click here for file

Additional file 5 **Mouse liver proteins**. A total of 9600 spots derived from 24 gels were excised and digested with trypsin (Promega). Peptides were loaded on an MTP Anchor Chip Target 600/384 (Bruker Daltonics) previously prepared with HCCA and analyzed in a MALDI-TOF-TOF spectrometer (Ultraflex, Bruker Daltonics). Peptide matching and protein searches were performed automatically with the MASCOT software. (MASCOT scores are reported in Additional File 4, see column "Score"). The identified proteins were organized with the ProteinScape™ database (Bruker Daltonics), checked individually, and only mouse proteins or highly homologous sequences from other species were considered. In the table, proteins are sorted by accession numbers, and the Swiss-Prot annotation is given. Protein names and gene names are reported as well. Function, subcellular location, and biological process are given herein. In the column "Spot" the minimum number of spots per gel is given for each protein. The column "Gel" indicates in how many different gels of the total 24 gels cut, each protein was identified. Each experiment consisted of 12 gels run at the same time, and a total of four experiments were carried out (see Figure 2). A comparison of our mouse liver proteome with those of two other research groups is given in the column "Who" (i = Fraunhofer ITEM; f = Fountoulakis *et al*.; e = Expasy). For example, the protein Aldehyde dehydrogenase 2, mitochondrial (gi|13529509) was identified in all three laboratories. The column "Lysis buffer (LB)" indicates the buffer in which the protein was identified. The column "M.R." reports which liver proteins are in common between mouse and rat. For the subcellular location the following abbreviations were adopted: C, cytosol; M, mitochondria; N, nucleus; P, peroxisome; S, secreted protein; ER, endoplasmic reticulum; G, golgi; L, lysosome; MEM, membrane; MIM, mitochondrial inner membrane; MM, mitochondrial matrix; OMM, outer mitochondrial membrane. Click here for file

Additional file 6 **Table 3**. List of the 55 proteins not found in the work of Lai et al. Click here for file

## References

[B1] Michalopoulos GK, DeFrances MC (1997). Liver regeneration. Science.

[B2] Jiang XS, Zhou H, Zhang L, Sheng QH, Li SJ, Li L, Hao P, Li YX, Xia QC, Wu JR, Zeng R (2004). A high-throughput approach for subcellular proteome: identification of rat liver proteins using subcellular fractionation coupled with two-dimensional liquid chromatography tandem mass spectrometry and bioinformatic analysis. Mol Cell Proteomics.

[B3] Klose J, Feller M (1981). Two-dimensional electrophoresis of membrane and cytosol proteins of mouse liver and brain. Electrophoresis.

[B4] Anderson NL, Swanson M, Giere FA, Tollaksen S, Gemmell A, Nance S, Anderson NG (1986). Effects of aroclor 1254 on proteins of mouse liver: Application of two-dimensional electrophoretic protein mapping. Electrophoresis.

[B5] Anderson NL, Giere FA, Nance SL, Gemmell MA, Tollaksen SL, Anderson NG (1987). Effects of toxic agents at the protein level: quantitative measurement of 213 mouse liver proteins following xenobiotic treatment. Fundam Appl Toxicol.

[B6] Wirth PJ, Vesterberg O (1988). Rat liver cytosolic protein changes after ethanol exposure studied by two-dimensional electrophoresis. Electrophoresis.

[B7] Benjamin T, Niu CH, Parmelee DC, Huggett AC, Yu B, Roller PP, Thorgeirsson SS (1989). Direct N-terminal sequence analysis of rat liver plasma membrane glycoproteins separated by two-dimensional polyacrylamide gel electrophoresis. Electrophoresis.

[B8] Gazzana G, Borlak J (2007). Improved method for proteome mapping of the liver by 2-DE MALDI-TOF MS. J Proteome Res.

[B9] Hochstrasser DF, Frutiger S, Paquet N, Bairoch A, Ravier F, Pasquali C, Sanchez JC, Tissot JD, Bjellqvist B, Vargas R (1992). Human liver protein map: a reference database established by microsequencing and gel comparison. Electrophoresis.

[B10] Giometti CS, Taylor J, Tollaksen SL (1992). Mouse liver protein database: a catalog of proteins detected by two-dimensional gel electrophoresis. Electrophoresis.

[B11] Fountoulakis M, Berndt P, Boelsterli UA, Crameri F, Winter M, Albertini S, Suter L (2000). Two-dimensional database of mouse liver proteins: changes in hepatic protein levels following treatment with acetaminophen or its nontoxic regioisomer 3-acetamidophenol. Electrophoresis.

[B12] Fountoulakis M, Juranville JF, Berndt P, Langen H, Suter L (2001). Two-dimensional database of mouse liver proteins. An update. Electrophoresis.

[B13] Sanchez JC, Chiappe D, Converset V, Hoogland C, Binz PA, Paesano S, Appel RD, Wang S, Sennitt M, Nolan A, Cawthorne MA, Hochstrasser DF (2001). The mouse SWISS-2D PAGE database: a tool for proteomics study of diabetes and obesity. Proteomics.

[B14] Da Cruz S, Xenarios I, Langridge J, Vilbois F, Parone PA, Martinou JC (2003). Proteomic analysis of the mouse liver mitochondrial inner membrane. J Biol Chem.

[B15] Kanaeva IP, Petushkova NA, Lokhov PG, Zgoda VG, Karuzina II, Lisitsa AV, Archakov AI (2004). Study of the mouse liver microsomes by the methods of proteome analysis. Biomed Khim.

[B16] Mi J, Kirchner E, Cristobal S (2007). Quantitative proteomic comparison of mouse peroxisomes from liver and kidney. Proteomics.

[B17] Zhang J, Xu X, Shen H, Zhang X (2006). Analysis of nuclear proteome in C57 mouse liver tissue by a nano-flow 2-D-LC-ESI-MS/MS approach. J Sep Sci.

[B18] Fountoulakis M, Juranville JF, Jiang L, Avila D, Röder D, Jakob P, Berndt P, Evers S, Langen H (2004). Depletion of the high-abundance plasma proteins. Amino Acids.

[B19] Righetti PG, Castagna A, Herbert B, Reymond F, Rossier JS (2003). Prefractionation techniques in proteome analysis. Proteomics.

[B20] Kerr JF (1971). Shrinkage necrosis: a distinct mode of cellular death. J Pathol.

[B21] Kanzler S, Galle PR (2000). Apoptosis and the liver. Semin Cancer Biol.

[B22] Taylor CA, Sun Z, Cliche DO, Ming H, Eshaque B, Jin S, Hopkins MT, Thai B, Thompson JE (2007). Eukaryotic translation initiation factor 5A induces apoptosis in colon cancer cells and associates with the nucleus in response to tumour necrosis factor alpha signalling. Exp Cell Res.

[B23] Yoshida K, Tomita Y, Okuda Y, Yamamoto S, Enomoto H, Uyama H, Ito H, Hoshida Y, Aozasa K, Nagano H, Sakon M, Kawase I, Monden M, Nakamura H (2006). Hepatoma-derived growth factor is a novel prognostic factor for hepatocellular carcinoma. Ann Surg Oncol.

[B24] Liu H, Barr SM, Chu C, Kohtz DS, Kinoshita Y, Johnson EM (2005). Functional interaction of Puralpha with the Cdk2 moiety of cyclin A/Cdk2. Biochem Biophys Res Commun.

[B25] Vasiliou V, Pappa A, Petersen DR (2000). Role of aldehyde dehydrogenases in endogenous and xenobiotic metabolism. Chem Biol Interact.

[B26] Kim I, Song X, Vig BS, Mittal S, Shin HC, Lorenzi PJ, Amidon GL (2004). A novel nucleoside prodrug-activating enzyme: substrate specificity of biphenyl hydrolase-like protein. Mol Pharm.

[B27] Endo S, Matsumoto K, Matsunaga T, Ishikura S, Tajima K, El-Kabbani O, Hara A (2006). Substrate specificity of a mouse aldo-keto reductase (AKR1C12). Biol Pharm Bull.

[B28] Vergnes L, Phan J, Stolz A, Reue K (2003). A cluster of eight hydroxysteroid dehydrogenase genes belonging to the aldo-keto reductase supergene family on mouse chromosome 13. J Lipid Res.

[B29] Lai KK, Kolippakkam D, Beretta L (2008). Comprehensive and quantitative proteome profiling of the mouse liver and plasma. Hepatology.

[B30] Breinig M, Caldas-Lopes E, Goeppert B, Malz M, Rieker R, Bergmann F, Schirmacher P, Mayer M, Chiosis G, Kern MA (2009). Targeting heat shock protein 90 with non-quinone inhibitors: a novel chemotherapeutic approach in human hepatocellular carcinoma. Hepatology.

[B31] Ding SJ, Li Y, Shao XX, Zhou H, Zeng R, Tang ZY, Xia QC (2004). Proteome analysis of hepatocellular carcinoma cell strains, MHCC97-H and MHCC97-L, with different metastasis potentials. Proteomics.

[B32] Lee NP, Chen L, Lin MC, Tsang FH, Yeung C, Poon RT, Peng J, Leng X, Beretta L, Sun S, Day PJ, Luk JM (2009). Proteomic expression signature distinguishes cancerous and nonmalignant tissues in hepatocellular carcinoma. J Proteome Res.

