# Supplementary material for: An update on the mouse liver proteome
Source: Proteome Sci. 2009 Sep 8;7:35. doi: 10.1186/1477-5956-7-35 (PMC2752743; doi:10.1186/1477-5956-7-35)
Supplement: Additional file 1 — Table 1. List of the 255 proteins identified from mouse liver extracts. Proteins were identified by MALDI-TOF MS and are grouped into 19 different biological processes. [file 1477-5956-7-35-S1.doc]

**Table 1**

| **N°** | **PROTEIN** |
| --- | --- |
| **Apoptosis** | |
| O09172 | Glutamate cysteine ligase, modifier subunit |
| P07823 | 78 kDa glucose-regulated protein [Precursor] |
| P21796 | Porin isoform 1 |
| P63242 | Eukaryotic initiation factor 5A isoform I variant CD |
| Q9CPU0 | Glyoxalase 1 |
| Q9JIY5 | Serine protease HTRA2, mitochondrial precursor |
| Q9R108 | Serine protease OMI |
| **Cell growth** | |
| P42208 | Septin 2 |
| P51859 | Hepatoma-derived growth factor |
| Q9CPT4 | Interleukin 25; lymphocyte antigen 6 complex, locus E ligand |
| Q9WTP6 | Adenylate kinase 2 |
| **Defense** | |
| O08677 | Kininogen 1; H-kininigen; L-kininogen |
| P01898 | MHC class I heavy chain |
| P29699 | Alpha-2-HS-glycoprotein |
| Q62418 | Drebrin-like |
| Q700Z7 | MHC class I antigen 2 |
| **Elongation factor** | |
| P57776 | Eukaryotic translation elongation factor 1 delta (guanine nucleotide exchange protein) isoform b |
| Q8BFR5 | Tu translation elongation factor, mitochondrial |
| Q9JHJ0 | Tropomodulin 3 |
|  | **Metabolism (general)** |
| O35244 | Peroxiredoxin 6 |
| P00949 | Phosphoglucomutase Mol_id: 1 |
| P35738 | Branched chain ketoacid dehydrogenase E1, beta polypeptide |
| P68040 | Guanine nucleotide-binding protein subunit beta 2-like 1 |
| Q3UGE3 | Phosphoglucomutase 2 |
| Q64513 | Triosephosphate isomerase |
| Q8BVI4 | Quininoid dihydropteridine reductase |
| Q8R013 | C1-tetrahydrofolate synthase |
| Q8VBT2 | L-serine dehydratase |
| Q8VC12 | Probable urocanate hydratase |
| Q8VCA8 | Secernin 2 |
| Q91W90 | Txndc5 protein |
| Q91XD4 | Formiminotransferase cyclodeaminase |
| Q922R8 | Protein disulfide isomerase-associated 6 |
| Q9CZ13 | Ubiquinol-cytochrome c reductase core protein 1 |
| Q9D6R2 | Isocitrate dehydrogenase [NAD] subunit alpha, mitochondrial [Precursor] |
| Q9DBM2 | L-specific multifunctional beta-oxdiation protein |
| Q9DCG6 | MAWD binding protein homolog 1 |
| Q9DCQ2 | Putative L-aspartate dehydrogenase |
| Q9QXE0 | 2-hydroxyphytanoyl-CoA lyase |
| Q9R257 | Heme-binding protein |
| **Metabolism (catabolism-proteasome)** | |
| O35562 | Protease (prosome, macropain) 28 subunit, beta, b |
| P35998 | Proteasome 26S ATPase subunit 2 |
| P62191 | Proteasome 26S ATPase subunit 1 |
| P62334 | 26S protease regulatory subunit S10B |
| P70195 | Proteasome subunit beta type 7 [Precursor] |
| P97371 | Proteasome (prosome, macropain) 28 subunit, alpha |
| P97372 | Proteasome activator subunit 2 |
| Q63570 | Proteasome 26S ATPase subunit 4 |
| Q9CR00 | Psmd9 |
| Q9D154 | Serpinb1a protein |
| Q9QUM9 | Proteasome (prosome, macropain) subunit, alpha type 6 |
| Q9R1P1 | Proteasome (prosome, macropain) subunit, beta type 3 |
| Q9R1P4 | Proteasome (prosome, macropain) subunit, alpha type 1 |
| **Metabolism (hydrolase activity)** | |
| P06576 | A28701 H+-transporting two-sector ATPase (EC 3.6.3.14) beta chain, mitochondrial |
| P10719 | ATP synthase, H+ transporting, mitochondrial F1 complex, beta subunit precursor; mitochondrial ATP synthetase, beta subunit |
| P25705 | ATP5A1 protein |
| P97823 | Lysophospholipase 1; phospholipase 1a; lysophopholipase 1 |
| Q8VC97 | Ureidopropionase, beta |
| Q8VCR7 | Abhydrolase domain-containing protein 14B (CCG1-interacting factor B) |
| Q91V76 | Ester hydrolase C11orf54 homolog (EC 3.1.-.-) 4931406C07Rik |
| Q91VM9 | Inorganic pyrophosphatase 2 |
| Q99JW2 | Aminoacylase 1 |
| Q99KR3 | Lactamase, beta 2 |
| Q99P30 | Nudix (nucleoside diphosphate linked moiety X)-type motif 7; coenzyme A diphosphatase |
| Q9BSE5 | PREDICTED: agmatine ureohydrolase (agmatinase) |
| Q9CPY7 | Cytosol aminopeptidase (Leucine aminopeptidase) (LAP) |
| Q9CWJ9 | 5-aminoimidazole-4-carboxamide ribonucleotide formyltransferase/IMP cyclohydrolase |
| Q9CWS0 | Dimethylarginine dimethylaminohydrolase 1 |
| Q9CYW4 | Haloacid dehalogenase-like hydrolase domain containing 3 |
| Q9D0S9 | Histidine triad nucleotide-binding protein 2 |
| Q9D1A2 | Cytosolic non-specific dipeptidase 2 (metallopeptidase M20 family) |
| Q9D358 | Low molecular weight phosphotyrosine protein phosphatase |
| Q9D7I5 | Phospholysine phosphohistidine inorganic pyrophosphate phosphatase homolog RIKEN cDNA 2310007H09 |
| Q9D819 | Pyrophosphatase |
| Q9DB20 | ATP synthase, H+ transporting, mitochondrial F1 complex, O subunit |
| Q9DB29 | Hypothetical Lipolytic enzyme |
| Q9JHW2 | Nit protein 2 |
| Q9JKB1 | Ubiquitin carboxyl-terminal esterase L3 (ubiquitin thiolesterase) |
| Q9QXD6 | Liver fructose-1,6-bisphosphatase |
| Q9QYR9 | Mitochondrial acyl-CoA thioesterase 1 |
| Q9R111 | Guanine deaminase |
| **Metabolism (ligase activity)** | |
| Q91VA0 | Butyryl Coenzyme A synthetase 1 |
| Q91ZA3 | Propionyl-Coenzyme A carboxylase, alpha polypeptide |
| Q9WUM5 | Succinate-CoA ligase, GDP-forming, alpha subunit |
| Q9Z2I9 | Succinyl-CoA ligase [ADP-forming] beta-chain, mitochondrial precursor |
| **Metabolism (lyase activity)** | |
| P97807 | Fumarate hydratase 1 |
| Q5FW97 | Hypothetical protein LOC433182 |
| Q8BH95 | Enoyl Coenzyme A hydratase, short chain, 1, mitochondrial |
| Q91WT9 | Cystathionine beta-synthase isoform 1 |
| Q91Y97 | Fructose-bisphosphate aldolase B |
| Q99KI0 | Aconitase 2, mitochondrial |
| Q9JLI6 | Selenocysteine lyase |
| **Metabolism (oxidoreductase activity)** | |
| P00329 | Alcohol dehydrogenase 1 (class I) |
| P07195 | Lactate dehydrogenase B |
| P48758 | Carbonyl reductase 1 |
| Q78JT3 | 3-hydroxyanthranilate 3,4-dioxygenase |
| Q7TNG8 | D-lactate dehydrogenase |
| Q80V68 | Phytanoyl-CoA dioxygenase domain containing 1 |
| Q80XN0 | D-beta-hydroxybutyrate dehydrogenase, mitochondrial [Precursor] |
| Q862E5 | Alpha-tubulin |
| Q8BJ64 | Choline dehydrogenase |
| Q8CHT0 | Delta-1-pyrroline-5-carboxylate dehydrogenase, mitochondrial [Precursor] |
| Q8K0E9 | Dhdh protein |
| Q8K2B3 | Succinate dehydrogenase [ubiquinone] flavoprotein subunit, mitochondrial [Precursor] |
| Q8R086 | Sulfite oxidase |
| Q8R0Y6 | 10-formyltetrahydrofolate dehydrogenase |
| Q8VCX1 | Aldo-keto reductase family 1, member D1 |
| Q8VDQ1 | Zinc binding alcohol dehydrogenase, domain containing |
| Q91VD9 | NADH dehydrogenase (ubiquinone) Fe-S protein 1 (Ndufs1) |
| Q91X52 | Diacetyl/L-xylulose reductase |
| Q91XF0 | Pyridoxine 5'-phosphate oxidase; pyridoxamine-phosphate oxidase |
| Q91Z53 | Glyoxylate reductase/hydroxypyruvate reductase |
| Q923D2 | Biliverdin reductase B |
| Q99L04 | Dehydrogenase/reductase (SDR family) member 1 |
| Q99L13 | 3-hydroxyisobutyrate dehydrogenase, mitochondrial precursor |
| Q99LB7 | Sarcosine dehydrogenase |
| Q9CQM9 | Thioredoxin-like 2 |
| Q9D051 | Pyruvate dehydrogenase (lipoamide) beta |
| Q9D6J6 | NADH dehydrogenase [ubiquinone] flavoprotein 2, mitochondrial [Precursor] |
| Q9DBF1 | Aldehyde dehydrogenase family 7, member A1 |
| Q9DBT9 | Dimethylglycine dehydrogenase precursor |
| Q9DCJ5 | NADH dehydrogenase (ubiquinone) 1 alpha subcomplex, 8 |
| Q9DCT2 | NADH dehydrogenase (ubiquinone) Fe-S protein 3 |
| Q9JHI5 | Isovaleryl coenzyme A dehydrogenase |
| Q9JII6 | Aldo-keto reductase family 1, member A4 |
| Q9JLJ2 | Aldehyde dehydrogenase 9, subfamily A1 |
| **Metabolism (transferase activity)** | |
| O09171 | Betaine-homocysteine methyltransferase |
| O35660 | Glutathione S-transferase Mu 6 |
| P24472 | Glutathione S-transferase A4 A Chain A |
| P46425 | Glutathione S-transferase, pi 2 |
| P55264 | Adenosine kinase |
| P97364 | Selenide,water dikinase 2 (Selenophosphate synthetase 2) (Selenium donor protein 2) |
| Q01205 | Dihydrolipoyllysine-residue succinyltransferase component of 2-oxoglutarate dehydrogenase complex, mitochondrial precursor |
| Q64471 | Glutathione S-transferase, theta 1 |
| Q80X81 | Acetyl-Coenzyme A acetyltransferase 3 |
| Q8BH69 | Selenophosphate synthetase 1 |
| Q8BWT1 | 3-ketoacyl-CoA thiolase, mitochondrial |
| Q8C717 | Hypothetical protein, full insert sequence |
| Q8JZK9 | 3-hydroxy-3-methylglutaryl-Coenzyme A synthase |
| Q8QZT1 | Acetyl-Coenzyme A acetyltransferase 1 precursor |
| Q8VCH0 | 3-ketoacyl-CoA thiolase B, peroxisomal precursor |
| Q91WU5 | Arsenite methyltransferase |
| Q91X83 | Methionine adenosyltransferase I, alpha |
| Q91X91 | Quinolinate phosphoribosyltransferase |
| Q91ZB1 | Dihydrolipoamide S-acetyltransferase precursor |
| Q921H8 | 3-ketoacyl-CoA thiolase A, peroxisomal precursor |
| Q922E4 | Phosphate cytidylyltransferase 2, ethanolamine |
| Q99J99 | 3-mercaptopyruvate sulfurtransferase |
| Q99JY0 | Hydroxyacyl-Coenzyme A dehydrogenase/3-ketoacyl-Coenzyme A thiolase/enoyl-Coenzyme A hydratase (trifunctional protein), beta subunit |
| Q9CZN7 | Serine hydroxymethyl transferase 2 (mitochondrial) |
| Q9D2G2 | Dihydrolipoamide S-succinyltransferase |
| Q9D404 | 3-oxoacyl-[acyl-carrier-protein] synthase, mitochondrial [Precursor] |
| Q9D5J6 | Carbohydrate kinase-like |
| Q9DCS2 | Hypothetical S-adenosyl-L-methionine-dependent methyltransferases structure containing protein, full insert sequence Hypothetical protein LOC68347 |
| Q9QXF8 | Glycine N-methyltransferase |
| Q9R0N0 | Galactokinase 1 |
| **Nucleic acidic enzyme** | |
| O95299 | NADH dehydrogenase [ubiquinone] 1 alpha subcomplex subunit 10, mitochondrial precursor |
| P31943 | Heterogeneous nuclear ribonucleoprotein H |
| P61979 | Heterogeneous nuclear ribonucleoprotein K |
| P70333 | Heterogeneous nuclear ribonucleoprotein H2 |
| P97855 | Ras-GTPase-activating protein SH3-domain binding protein |
| Q9DBP5 | UMP-CMP kinase (Cytidylate kinase) (Deoxycytidylate kinase) (Cytidine monophosphate kinase) |
| Q9Z1D1 | Eukaryotic translation initiation factor 3, subunit 4 (delta) |
| Q9Z2X1 | Heterogeneous nuclear ribonucleoprotein F |
| **Protein folding** | |
| O35501 | Stress-70 protein, mitochondrial precursor (75 kDa glucose-regulated protein) (GRP 75) |
| P14625 | Protein kinase |
| Q29092 | Heat shock protein gp96 precursor |
| Q60432 | 170 kDa glucose-regulated protein |
| Q66HD0 | Similar to Endoplasmin precursor (Endoplasmic reticulum protein 99) (94 kDa glucose-regulated protein) (GRP94) (ERP99) |
| Q7TSZ0 | Heat shock protein, A |
| Q922Z3 | Trap1 protein |
| Q99LP6 | GrpE-like 1, mitochondrial |
| Q99LX0 | DJ-1 protein |
| Q9D1Q6 | Thioredoxin domain containing 4 |
| Q9WU84 | Copper chaperone for superoxide dismutase |
| O35501 | Stress-70 protein, mitochondrial precursor (75 kDa glucose-regulated protein) (GRP 75) |
| **Structural proteins** | |
| O89053 | Coronin, actin binding protein 1A |
| P35527 | Cytokeratin 9 |
| P60710 | Put. beta-actin (aa 27-375) |
| P63260 | Gamma-actin |
| P68372 | Tubulin, beta, 2 |
| P70441 | Solute carrier family 9 (sodium/hydrogen exchanger), isoform 3 regulator 1 |
| Q13885 | class II beta tubulin isotype |
| Q99JY9 | Actin-like protein 3 |
| Q9CPW4 | Actin related protein 2/3 complex, subunit 5 |
| Q9CQ60 | 6-phosphogluconolactonase (6PGL) |
| **Translation** | |
| P11940 | Polyadenylate binding protein II |
| P46782 | Ribosomal protein S5, cytosolic |
| Q91V31 | Protein 40kD (34/67 kDa laminin receptor) |
| Q9CXW4 | Rpl11 protein |
| Q9QZD9 | Eukaryotic translation initiation factor 3, subunit 2 (beta) |
| **Transport** | |
| O35768 | A Chain A, Dimethyl Propionate Ester Heme-Containing Cytochrome B5 |
| P00173 | Cytochrome b5 1205244A |
| P02770 | Albumin |
| P07309 | Transthyretin |
| P34022 | Ranbp1 protein |
| P53808 | Phosphatidylcholine transfer protein |
| P53810 | Phosphatidylinositol transfer protein alpha isoform |
| Q4FZE8 | Major urinary protein 1 |
| Q8BXK9 | Chloride intracellular channel 5 |
| Q8CDN6 | Thioredoxin-like 1 |
| Q91X72 | Hemopexin |
| Q921I1 | Transferrin |
| Q99747 | N-ethylmaleimide-sensitive factor attachment protein, gamma |
| Q99LC5 | Electron transferring flavoprotein, alpha polypeptide |
| Q99PL5 | Ribosome-binding protein 1 |
| Q9CZ44 | NSFL1 cofactor p47 (p97 cofactor) |
| Q9DBG5 | Mannose-6-phosphate receptor-binding protein 1 |
| Q9DCW4 | Etfb protein |
| Q9DCX2 | ATP synthase, H+ transporting, mitochondrial F0 complex, subunit d |
| O35768 | A Chain A, Dimethyl Propionate Ester Heme-Containing Cytochrome B5 |
| **Transcription factors** | |
| O70400 | PDZ and LIM domain protein 1 |
| P17980 | 26S protease regulatory subunit 6A |
| P42669 | Similar to transcriptional activator protein PUR-alpha |
| P63159 | High mobility group protein B1 |
| Q13765 | Nascent-polypeptide-associated complex alpha polypeptide |
| Q61656 | Probable ATP-dependent RNA helicase DDX5 |
| Q8R0M2 | Ugp2 protein |
| Q8VIJ6 | Splicing factor proline/glutamine rich (polypyrimidine tract binding protein associated) |
| Q99020 | Heterogeneous nuclear ribonucleoprotein A/B |
| Q9CQT1 | Hypothetical Initiation factor 2B containing protein, full insert sequence RIKEN cDNA 2410018C20 |
| Q9DCM0 | ETHE1 protein, mitochondrial [Precursor] ( Hepatoma subtracted clone one protein) |
| **Unknown** | |
| O88342 | WD repeat domain 1 |
| P04797 | Glyceraldehyde 3-phosphate-dehydrogenase |
| P57759 | Endoplasmic reticulum protein ERp29 precursor |
| P67778 | Prohibitin |
| P70296 | Phosphatidylethanolamine binding protein |
| Q05816 | Fatty acid binding protein 5, epidermal |
| Q283N4 | OHCU decarboxylase |
| Q2KII5 | HSBO22 histone H2B |
| Q3UZU5 | Hypothetical TPR repeat/TPR-like containing protein, full insert sequence Similar to FLJ20699 protein |
| Q61838 | Alpha-2-macroglobulin [Precursor] |
| Q63610 | Tropomyosin |
| Q76MZ3 | Serine/threonine-protein phosphatase 2A 65 kDa regulatory subunit A alpha isoform |
| Q8C5W3 | Leucine rich repeat containing 35 |
| Q8CAQ8 | Mitochondrial inner membrane protein |
| Q8K0E8 | Fibrinogen, B beta polypeptide |
| Q8K157 | Aldose 1-epimerase |
| Q8K1Z0 | Ubiquinone biosynthesis protein COQ9, mitochondrial [Precursor] |
| Q8VBW8 | cDNA sequence BC021608 |
| Q91Z81 | Similar to peroxiredoxin 1 |
| Q99JB2 | Stomatin-like protein 2 |
| Q99KJ8 | Dynactin 2 |
| Q99PT1 | Rho GDP-dissociation inhibitor 1 |
| Q9CPV4 | Hypothetical Glyoxalase/Bleomycin resistance protein/Dihydroxybiphenyl dioxygenase structure containing protein, full insert sequence RIKEN cDNA 2700085E05 |
| Q9CY58 | Serbp1 protein |
| Q9D172 | ES1 protein homolog, mitochondrial [Precursor] |
| Q9ERE7 | Mesoderm development candidate 2 |
| Q9GLW6 | ERP57 protein |
| Q9JMD3 | START domain containing 10 |
| Q9QYG0 | N-myc downstream regulated gene 2 |
| **Xenobiotic/detoxification** | |
| Q8R164 | Biphenyl hydrolase-like |
| Q8VC28 | Aldo-keto reductase family 1 member C13 |
| Q91X42 | Aldo-keto reductase family 1, member C12 |
| Q9CZS1 | Aldehyde dehydrogenase 1 family, member B1 |
| Q9JI75 | NAD(P)H dehydrogenase, quinone 2 |
| Q9R0P3 | Esterase D/formylglutathione hydrolase |
