# Supplementary material for: An update on the mouse liver proteome
Source: Proteome Sci. 2009 Sep 8;7:35. doi: 10.1186/1477-5956-7-35 (PMC2752743; doi:10.1186/1477-5956-7-35)
Supplement: Additional file 2 — 2-DE Gel, pH 5-8. Representative 2-D gel: pH 5-8 (loading sample 1.5 mg; stain: coomassie blue; 17-cm IPG strips, BioRad). [file 1477-5956-7-35-S2.ppt]

## Slide 1
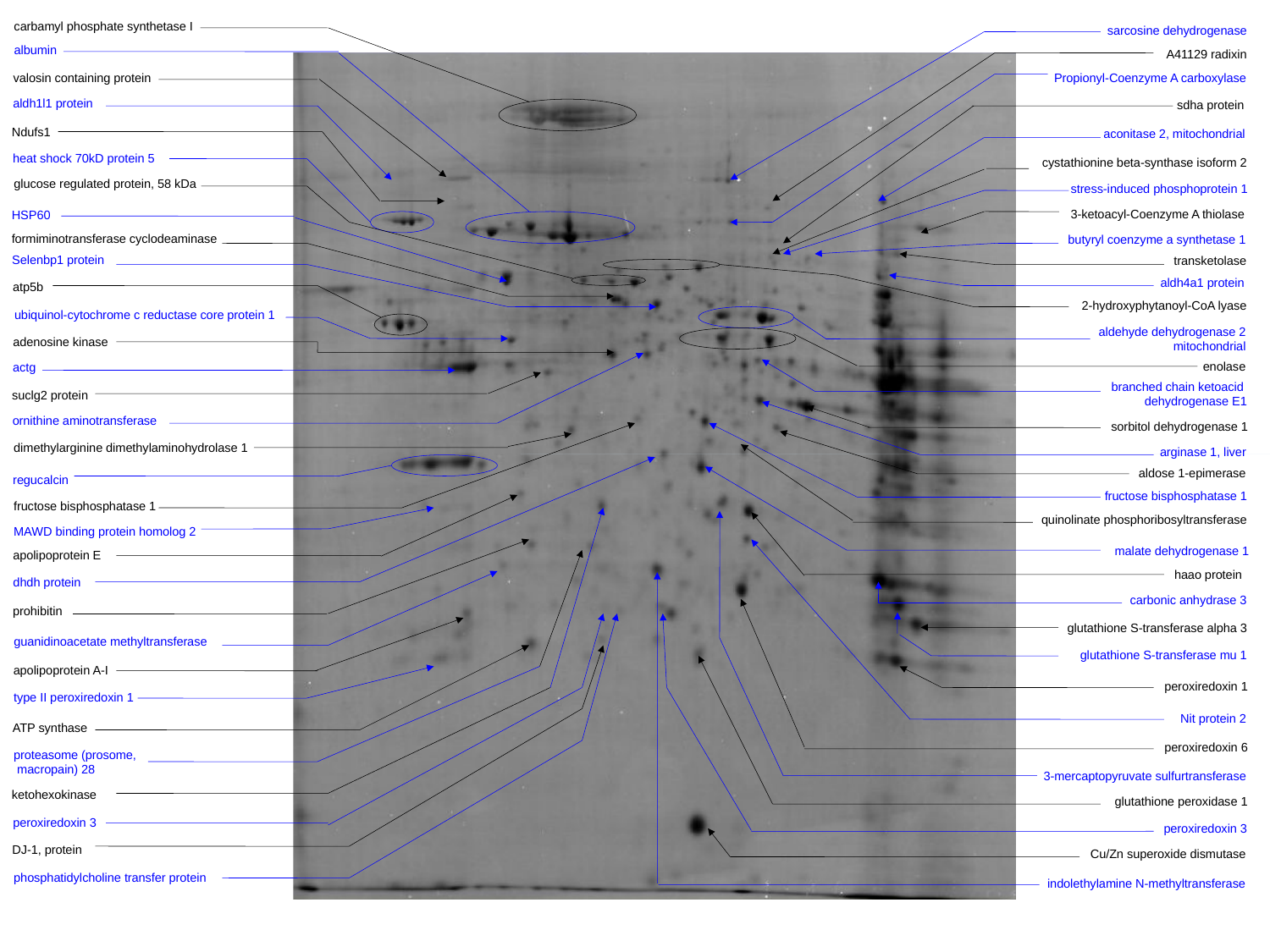

carbamyl phosphate synthetase I
sarcosine dehydrogenase
albumin
A41129 radixin
valosin containing protein
Propionyl-Coenzyme A carboxylase
aldh1l1 protein
sdha protein
Ndufs1
aconitase 2, mitochondrial
heat shock 70kD protein 5
cystathionine beta-synthase isoform 2
glucose regulated protein, 58 kDa
stress-induced phosphoprotein 1
3-ketoacyl-Coenzyme A thiolase
HSP60
formiminotransferase cyclodeaminase
butyryl coenzyme a synthetase 1
Selenbp1 protein
transketolase
aldh4a1 protein
atp5b
2-hydroxyphytanoyl-CoA lyase
ubiquinol-cytochrome c reductase core protein 1
aldehyde dehydrogenase 2
mitochondrial
adenosine kinase
enolase
actg
branched chain ketoacid
dehydrogenase E1
suclg2 protein
ornithine aminotransferase
sorbitol dehydrogenase 1
dimethylarginine dimethylaminohydrolase 1
arginase 1, liver
aldose 1-epimerase
regucalcin
fructose bisphosphatase 1
fructose bisphosphatase 1
quinolinate phosphoribosyltransferase
MAWD binding protein homolog 2
malate dehydrogenase 1
apolipoprotein E
haao protein
dhdh protein
carbonic anhydrase 3
prohibitin
glutathione S-transferase alpha 3
guanidinoacetate methyltransferase
glutathione S-transferase mu 1
apolipoprotein A-I
peroxiredoxin 1
type II peroxiredoxin 1
Nit protein 2
ATP synthase
peroxiredoxin 6
proteasome (prosome,
 macropain) 28
3-mercaptopyruvate sulfurtransferase
ketohexokinase
glutathione peroxidase 1
peroxiredoxin 3
peroxiredoxin 3
DJ-1, protein
Cu/Zn superoxide dismutase
phosphatidylcholine transfer protein
indolethylamine N-methyltransferase
