# Supplementary material for: An update on the mouse liver proteome
Source: Proteome Sci. 2009 Sep 8;7:35. doi: 10.1186/1477-5956-7-35 (PMC2752743; doi:10.1186/1477-5956-7-35)
Supplement: Additional file 3 — 2-DE Gel, pH 7-10. Representative 2-D gel: pH 7-10 (loading sample 1.5 mg; stain: coomassie blue; 17-cm IPG strips, BioRad). [file 1477-5956-7-35-S3.ppt]

## Slide 1
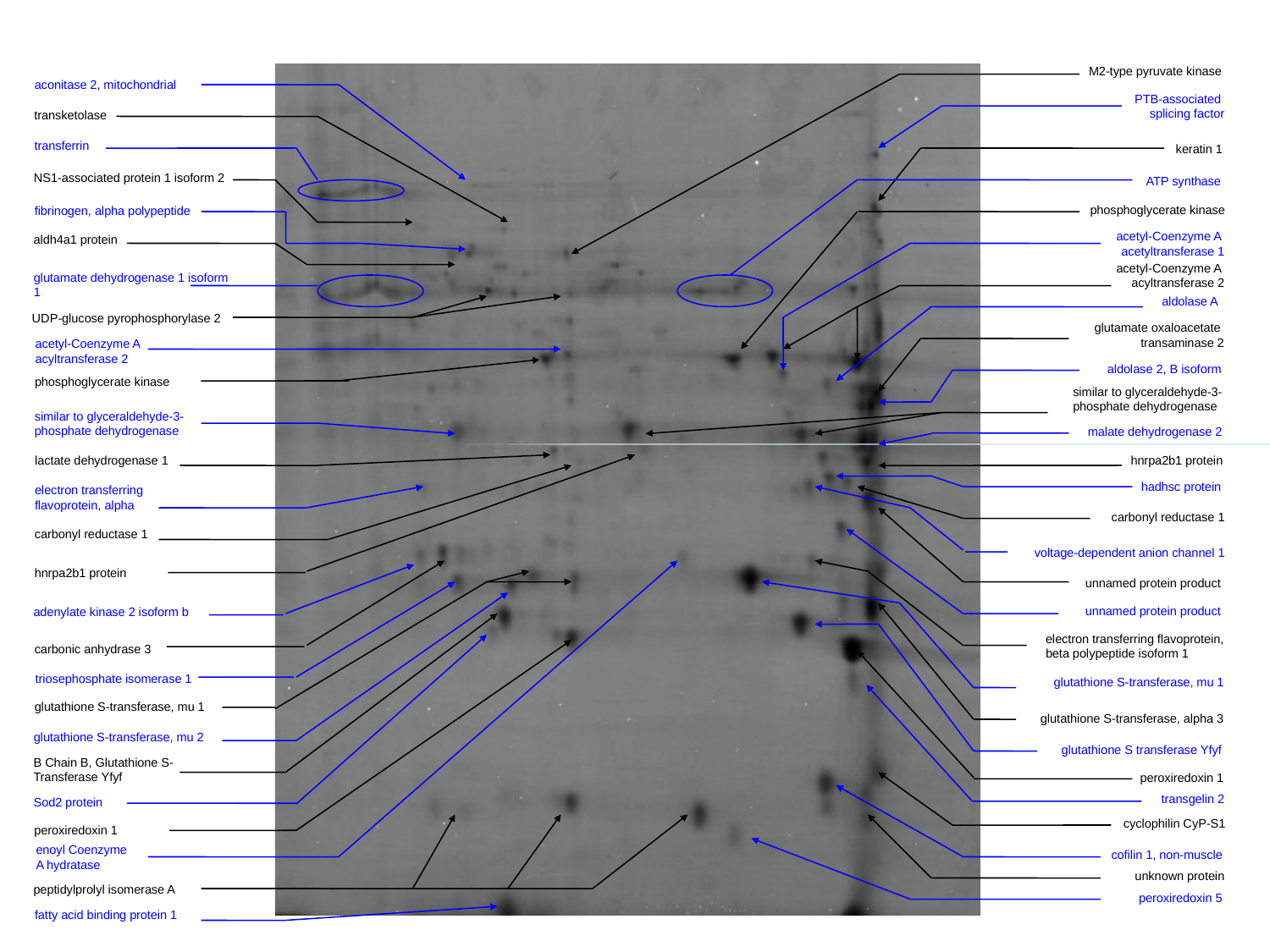

M2-type pyruvate kinase
aconitase 2, mitochondrial
PTB-associated
splicing factor
transketolase
transferrin
keratin 1
NS1-associated protein 1 isoform 2
ATP synthase
phosphoglycerate kinase
fibrinogen, alpha polypeptide
acetyl-Coenzyme A
acetyltransferase 1
aldh4a1 protein
acetyl-Coenzyme A
acyltransferase 2
glutamate dehydrogenase 1 isoform 1
aldolase A
UDP-glucose pyrophosphorylase 2
glutamate oxaloacetate
transaminase 2
acetyl-Coenzyme A
acyltransferase 2
aldolase 2, B isoform
phosphoglycerate kinase
similar to glyceraldehyde-3-
phosphate dehydrogenase
similar to glyceraldehyde-3-
phosphate dehydrogenase
malate dehydrogenase 2
lactate dehydrogenase 1
hnrpa2b1 protein
hadhsc protein
electron transferring
flavoprotein, alpha
carbonyl reductase 1
carbonyl reductase 1
voltage-dependent anion channel 1
hnrpa2b1 protein
unnamed protein product
unnamed protein product
adenylate kinase 2 isoform b
electron transferring flavoprotein,
beta polypeptide isoform 1
carbonic anhydrase 3
triosephosphate isomerase 1
glutathione S-transferase, mu 1
glutathione S-transferase, mu 1
glutathione S-transferase, alpha 3
glutathione S-transferase, mu 2
glutathione S transferase Yfyf
B Chain B, Glutathione S-Transferase Yfyf
peroxiredoxin 1
transgelin 2
Sod2 protein
cyclophilin CyP-S1
peroxiredoxin 1
enoyl Coenzyme
A hydratase
cofilin 1, non-muscle
unknown protein
peptidylprolyl isomerase A
peroxiredoxin 5
fatty acid binding protein 1
