# Supplementary material for: An update on the mouse liver proteome
Source: Proteome Sci. 2009 Sep 8;7:35. doi: 10.1186/1477-5956-7-35 (PMC2752743; doi:10.1186/1477-5956-7-35)
Supplement: Additional file 4 — Table 2. In the Table 2, the protein name, accession number, theoretical Mr and pI are reported together with MASCOT score, percentage of peptide coverage, number of identified peptides and sequence. Five matching peptides (see column "Pep") and at least 10% peptide coverage of the theoretical sequences was the minimal requirement for an identity assignment (see column "%"). [file 1477-5956-7-35-S4.doc]

N°	Protein name	Ncbi	Mr (kDa)	pI	Score	%	Pep.	Sequence	
1	Hypothetical S-adenosyl-L-methionine-dependent methyltransferases structure containing protein, full insert sequence Hypothetical protein LOC68347 	Q9DCS2
	22,7	5,8	125	71	10	AVLITYGPYAVNGK
CcamLDSIAATTR
CcamRNPEWGLR
DTVLLEELGQASGLVLER
ISPQSNVDFDLTLR
NKEPILSVLR
QYVDPAQRCcamVR
SLDLLLCcamINMoxIHISPLNCcamTEGLFR
TKAVLITYGPYAVNGK
VLEVASGSGQHAAHFAQAFPNAEWQPSDVDQR	
2	Putative L-aspartate dehydrogenase (EC 1.4.1.21). RIKEN cDNA 0610012D14 	Q9DCQ2 	30,3	6,3	164	67	12	GALWGSEDISR
GALWGSEDISRLDAAGGLQSLR
HANLLVGSPSALADQTTEQQLLEASKR
IIHESGAQILR
LDAAGGLQSLR
MoxAGSVPPALQLQDLTALEER
MoxAGSVPPALQLQDLTALEERHPDLVVEVAHPK
NSNTMAAAALAAPSLGFDR
NSNTMoxAAAALAAPSLGFDR
TVLYEGPVRGLCcamPLAPR
VIGVLVADLSLTDMoxHVVDVELLGPPGPSGR
VTMoxATHPDGFRLEGPLAAAHSSGPR
WGHTVFVAR	
3	Phospholysine Phosphohistidine Inorganic Pyrophosphate Phosphatase Homolog RIKEN cDNA 2310007H09 	Q9D7I5 	29,1	5	229	77	18	AEVVGKPSPEFFK
AFQVLMELENPVLISLGK
AFQVLMELENPVLISLGKGR
AFQVLMoxELENPVLISLGK
AFQVLMoxELENPVLISLGKGR
ALEYACcamGIKAEVVGKPSPEFFK
FRPGDEHHPEVQADGYVDNLAEAVDLLLK
FRPGDEHHPEVQADGYVDNLAEAVDLLLKYTDK
GLRPHLLIHEGVR
GVLLDISGVLCcamDSSASGATAIAGSVEAVAR
LTGVRGVLLDISGVLCcamDSSASGATAIAGSVEAVAR
oxSALQAIGVEAHQAIMIGDDIVGDVGGAQQCcamGMR
oxYYKETSGLMLDVGGYMK
SALQAIGVEAHQAIMoxIGDDIVGDVGGAQQCcamGMoxR
SLRELVGVLQQLGFDISEEEVTAPAPATCcamQILK
TGKFRPGDEHHPEVQADGYVDNLAEAVDLLLK
YYKETSGLMLDVGGYMK
YYKETSGLMoxLDVGGYMoxK	
4	Hypothetical Initiation factor 2B containing protein, full insert sequence RIKEN cDNA 2410018C20 	Q9CQT1 	39,4	5,5	130	57	13	AGAGGPGLAALVAFVR
AGAGGPGLAALVAFVRDQLR
DLGQVAAQEAER
DLGQVAAQEAEREGATEETVR
EIVIEERPSQELTDLNGVR
GGKVTVLTHCcamNTGALATAGYGTALGVIR
HLLEQTNPR
LEHTFCcamTETRPYNQGAR
LLVAARPTAVNMoxAR
LTAFELVYEQIPATLITDSMoxAAAAMoxAHR
VTVLTHCcamNTGALATAGYGTALGVIR
VVANGDTANKIGTYQLAIVAK
YSPGSLQILDQLQLPEHCcamHYEALSSVQQASEAIR	
5	Hypothetical Glyoxalase/Bleomycin resistance protein/Dihydroxybiphenyl dioxygenase structure containing protein, full insert sequence RIKEN cDNA 2700085E05 (Novel protein) 	Q9CPV4 	33,3	5,2	220	70	19	AACcamNGPYDGKWSK
ALHFVFKVK
ALLGYADNQCcamK
ALLGYADNQCcamKLELQGIQGAVDHAAAFGR
DVLGMoxQVLR
ELPDLEDLMoxKR
ESHSILTPLVSLDTPGK
FQTVHFFR
HEEFEEGCcamK
IYEQDEEKQR
LELQGIQGAVDHAAAFGR
LGNDFMGITLASSQAVSNAR
LGNDFMoxGITLASSQAVSNAR
LLDDAMoxEADKSDEWFATR
NRFQTVHFFR
SLNYWSNLLGMoxK
SPSQSDPVLKVTLAVSDLQK
VAEGIFETEAPGGYK
VAEGIFETEAPGGYKFYLQDR	
6	Ester hydrolase C11orf54 homolog (EC 3.1.-.-) 4931406C07Rik 	Q91V76 	35	5,8	184	61,3	19	AHIMoxPAEFSSCcamPLNSDEAVNK
APLVCcamLPVFVSKDPGLDLR
GLTDNFADVQVSVVDCcamPDLTKEPFTFPVR
IAEVGGVPYLLPLVNK
IAEVGGVPYLLPLVNKK
IDQPKETHAFGR
KVYDLNEIAK
NPADGACcamLLEK
QTLEEHYGDKPVGMGGTFIVQK
QTLEEHYGDKPVGMoxGGTFIVQK
RTGELNFVSCcamMoxR
TGELNFVSCcamMoxR
TGELNFVSCcamMR
VYDLNEIAK
WLHFYEMK
WLHFYEMoxK
YHDFGCcamALLANLFASEGQPGK
YHDFGCcamALLANLFASEGQPGKVIEVQAK
YSQKYHDFGCcamALLANLFASEGQPGK	
7	Hypothetical protein, full insert sequence Unnamed protein product 	Q8C717
	55,3	6,1	166	45	16	ADSDPHGPHTCcamGHVLNVIIGSNSLALAEAQR
AKGPVCcamLLAGGEPTVQLQGSGK
AVLGMAAAAEELLAQHLVQGVISVPK
AVLGMoxAAAAEELLAQHLVQGVISVPK
EMoxLLKPHSR
ENLYLVGFGK
FRGGTHLLHTGLTGTNVMoxDVHLLILHPQ
GATIQELNTIR
GATIQELNTIRK
GPVCcamLLAGGEPTVQLQGSGK
IQVFEGAEDNLPDRDALR
NQELALHVGVELGR
QAEVLGYHAMoxVLSTAMoxQGDVKR
QLFDSAVGAVQPGPMoxLQR
QPLGPIDVLFLSGGTDGQDGPTK
TFQLRENLYLVGFGK	
8	Abhydrolase domain-containing protein 14B (CCG1-interacting factor B). RIKEN cDNA 1810013B01 	Q8VCR7 	22,5	5,6	172	69	12	AVAIDLPGLGR
ETRPGSGQPVR
ETRPGSGQPVRFSVLLLHGIR
FSSETWQNLGTLQR
FSVLLLHGIR
GFVPVAPICcamTDK
GFVPVAPICcamTDKINAVDYASVK
LAEAGYRAVAIDLPGLGR
MoxAGVDQHEGTIQVQGQNLFFR
TPALIVYGDQDPMoxGSSSFQHLK
TPALIVYGDQDPMoxGSSSFQHLKQLPNHR
VLVMoxEGAGHPCcamYLDKPDEWHK	
9	3-ketoacyl-CoA thiolase A, peroxisomal precursor (EC 2.3.1.16) (Beta-  ketothiolase A) (Acetyl-CoA acyltransferase A) (Peroxisomal 3-oxoacyl-  CoA thiolase A 	Q921H8 	44	8,7	241	75	24	AEELGLPILGVLR
AGLTVNDIDIFEINEAFASQAVYCcamVEK
ARDCcamLTPMoxGMoxTSENVAER
DCcamLTPMGMTSENVAER
DGGSTTAGNSSQVSDGAAAVLLAR
FPQASASDVVVVHGR
FPQASASDVVVVHGRR
GGFKNTTPDELLSAVLTAVLQDVR
IAQFLSGIPETVPLSTVNR
LGIPAEKVNPLGGAIALGHPLGCcamTGAR
LKPAFKDGGSTTAGNSSQVSDGAAAVLLAR
LKPEQLGDISVGNVLEPGAGAVMAR
LKPEQLGDISVGNVLEPGAGAVMoxAR
NTTPDELLSAVLTAVLQDVR
oxDCcamLTPMGMTSENVAER
oxNGSYDIGMACcamGVESMSLSGMGNPGNISSR
QCcamSSGLQAVANIAGGIR
QKQDDFALASQQK
QVVTLLNELKR
SKAEELGLPILGVLR
SYAVVGVPPDVMGIGPAYAIPAALQK
SYAVVGVPPDVMoxGIGPAYAIPAALQK
TITVSQDEGVRPSTTMoxQGLAK
TITVSQDEGVRPSTTMQGLAK
VNPLGGAIALGHPLGCcamTGAR	
10	3-ketoacyl-CoA thiolase B, peroxisomal precursor (EC 2.3.1.16) (Beta-  ketothiolase B) (Acetyl-CoA acyltransferase B) (Peroxisomal 3-oxoacyl-  CoA thiolase B) 	Q8VCH0 	44	8,8	146	69	23	AEELGLPILGVLR
AGLTVNDIDIFEINEAFASQAVYCcamVEK
ARDCcamLIPMoxGITSENVAER
DCcamLIPMoxGITSENVAER
DGGSTTAGNSSQVSDGAAAVLLAR
DTTPDELLSAVLTAVLQDVK
GCcamFKDTTPDELLSAVLTAVLQDVK
IAQFLSGIPETVPLSTVNR
LGIPAEKVNPLGGAIALGHPLGCcamTGAR
LKPAFKDGGSTTAGNSSQVSDGAAAVLLAR
LKPEQLGDISVGNVLQPGAGAIMAR
LKPEQLGDISVGNVLQPGAGAIMoxAR
NGSYDIGMACcamGVESMTLSQR
oxNGSYDIGMACcamGVESMTLSQR
QCcamSSGLQAVANIAGGIR
QKQDAFALASQQK
QVVTLLNELK
QVVTLLNELKR
SKAEELGLPILGVLR
SYAVVGVPPDVMoxGIGPAYAIPAALQK
TITVSQDEGVRPSTTMoxQGLAK
TITVSQDEGVRPSTTMQGLAK
VNPLGGAIALGHPLGCcamTGAR	
11	3-ketoacyl-CoA thiolase, mitochondrial (EC 2.3.1.16) (Beta-  ketothiolase) (Acetyl-CoA acyltransferase) (Mitochondrial 3-oxoacyl-  CoA thiolase)	Q8BWT1 	41,8	8,3	255	90	34	AALSAGKVPPETIDSVIVGNVMoxQSSSDAAYLAR
AALSAGKVPPETIDSVIVGNVMQSSSDAAYLAR
AANEAGYFNEEMAPIEVK
AANEAGYFNEEMoxAPIEVK
AGLSLKDMDLIDVNEAFAPQFLSVQK
AGLSLKDMoxDLIDVNEAFAPQFLSVQK
ALDLDPSKTNVSGGAIALGHPLGGSGSR
DAEVVLCcamGGTESMoxSQSPYCcamVR
DAEVVLCcamGGTESMSQSPYCcamVR
DFSATDLTEFAAR
DGTVTAGNASGVSDGAGAVIIASEDAVKK
EDCcamDRYALQSQQR
GGKYAVGSACcamIGGGQGIALIIQNTA
HVGLRVGVPTETGALTLNR
ITAHLVHELR
ITAHLVHELRR
KHNFTPLAR
LCcamGSGFQSIVSGCcamQEICcamSK
LEDTLWAGLTDQHVK
LPMoxGMoxTAENLAAK
oxLPMGMTAENLAAK
QTMoxQVDEHARPQTTLEQLQK
QTMQVDEHARPQTTLEQLQK
RTPFGAYGGLLK
TNVSGGAIALGHPLGGSGSR
TNVSGGAIALGHPLGGSGSRITAHLVHELR
TPFGAYGGLLK
TPFGAYGGLLKDFSATDLTEFAAR
VGVPTETGALTLNR
VPPETIDSVIVGNVMoxQSSSDAAYLAR
VPPETIDSVIVGNVMQSSSDAAYLAR
VVGYFVSGCcamDPTIMGIGPVPAINGALKK
VVGYFVSGCcamDPTIMoxGIGPVPAINGALKK
WKAANEAGYFNEEMAPIEVK
WKAANEAGYFNEEMoxAPIEVK
YAVGSACcamIGGGQGIALIIQNTA
YNISREDCcamDR	
12	Acyl-Coenzyme A dehydrogenase, short chain; acetyl-Coenzyme A dehydrogenase, short chain 	Q07417 	44,9	8,7	116	53	20	ACcamASTGVIMoxSVNNSLYLGPILK
ACcamASTGVIMSVNNSLYLGPILK
ASSTANLIFEDCcamR
ASSTANLIFEDCcamRIPK
ELVPIAAQLDREHLFPTAQVK
GISAFLVPMoxPTPGLTLGK
GISAFLVPMPTPGLTLGK
IGCcamFALSEPGNGSDAGAASTTAR
IGIASQALGIAQASLDCcamAVK
IGIASQALGIAQASLDCcamAVKYAENR
ITEIYEGTSEIQR
KPFTKESAMoxAK
LAASEAATAISHQAIQILGGMoxGYVTEMoxPAER
LADMALALESAR
LADMoxALALESAR
LHTVYQSVELPETHQMLR
LHTVYQSVELPETHQMoxLR
oxIAMQTLDMGR
oxLAASEAATAISHQAIQILGGMGYVTEMPAER
QQWITPFTNGDK	
13	Acetyl-Coenzyme A acetyltransferase 1 precursor 	Q8QZT1 	44,8	8,7	142	66	24	DGLTDVYNKIHMoxGNCcamAENTAK
ENGTITAANASTLNDGAAALVLMoxTAEAAQR
ENGTITAANASTLNDGAAALVLMTAEAAQR
EVYMGNVIQGGEGQAPTR
EVYMoxGNVIQGGEGQAPTR
FASEITPITISVK
GATPYGGVKLEDLIVK
IAAFADAAVDPIDFPLAPAYAVPK
IHMGNCcamAENTAK
IVVHMAHALKPGEFGLASICcamNGGGGASALLIEK
IVVHMoxAHALKPGEFGLASICcamNGGGGASALLIEK
LGTAAIQGAIEK
MLEIDPQKVNIHGGAVSLGHPIGMSGAR
MoxAALVALHGVVRRPLLR
MoxLEIDPQKVNIHGGAVSLGHPIGMoxSGAR
MoxNISRQEQDTYALSSYTR
oxMLEIDPQKVNIHGGAVSLGHPIGMSGAR
QATLGAGLPISTPCcamTTVNKVCcamASGMK
QATLGAGLPISTPCcamTTVNKVCcamASGMoxK
QEQDTYALSSYTR
TPIGSFLGSLASQPATK
TVFQKENGTITAANASTLNDGAAALVLMTAEAAQR
VNIHGGAVSLGHPIGMoxSGAR
VNIHGGAVSLGHPIGMSGAR	
14	Acetyl-Coenzyme A acetyltransferase 3 	Q80X81
	41,5	8,1	158	64	17	AGHFDKEIVPVLVSSR
AKVAPEEVSEVIFGHVLTAGCcamGQNPTR
EAQDKVAVLSQNR
EIVPVLVSSRK
ELGLNPEKVNINGGAIALGHPLGASGCcamR
GLTEVKIDEFPR
GVAALCcamIGGGMoxGVAMoxCcamVQR
ILVTLLHTLERVGGTR
IVSWSQAGVEPSVMGVGPIPAIK
QASVGAGIPYSVPAWSCcamQMoxICcamGSGLK
SDPVVIVSAAR
TAIGSFNGALSTVPVHEMGTTVIKEVLQR
TAIGSFNGALSTVPVHEMoxGTTVIK
TAIGSFNGALSTVPVHEMoxGTTVIKEVLQR
VAPEEVSEVIFGHVLTAGCcamGQNPTR
VGEVPLADSILCcamDGLTDAFHNYHMoxGITAENVAK
VNINGGAIALGHPLGASGCcamR	
15	Aconitase 1 	P28271
	98,1	7,8	197	28	17	ANYLASPPLVIAYAIAGTVR
DFNDTSQDPDFTQVVELDLK
EPLGVNAQGRQVFLK
FVEFFGPGVAQLSIADR
IDFEKEPLGVNAQGR
IIPPGSGIIHQVNLEYLAR
INPVCcamPADLVIDHSIQVDFNR
LDTGKTFQAVMoxR
NIEVPFKPAR
QAPQTVHLPSGETLDVFDAAER
SPPFFESLTLDLQPPK
TSLSPGSGVVTYYLR
VILQDFTGVPAVVDFAAMoxR
WGSQAFCcamNMoxR
YGRLPFSIR
YLQAVGMoxFR
YTINIPEDLKPR	
16	Aconitase 2, mitochondrial 	Q99KI0
	85,4	8,9	364	54	38	AKDINQEVYNFLATAGAK
ATIERDGYAQILR
CcamKSQFTITPGSEQIR
CcamTTDHISAAGPWLK
CcamVIKHPNGTQETILLNHTFNETQIEWFR
DINQEVYNFLATAGAK
DVGGIVLANACcamGPCcamIGQWDRK
FKLEAPDADELPR
FNPETDFLTGK
FNPETDFLTGKDGK
FRGHLDNISNNLLIGAINIENGK
GHLDNISNNLLIGAINIENGK
GHLDNISNNLLIGAINIENGKANSVR
GKCcamTTDHISAAGPWLK
IVYGHLDDPANQEIER
IVYGHLDDPANQEIERGK
LNRPLTLSEK
LTGSLSGWTSPKDVILK
NAVTQEFGPVPDTAR
NDANPETHAFVTSPEIVTALAIAGTLK
NFTGRNDANPETHAFVTSPEIVTALAIAGTLK
oxVAMQDATAQMAMLQFISSGLPK
QGLLPLTFADPSDYNK
RLNRPLTLSEK
SAAVAKQALAHGLK
SDFDPGQDTYQHPPKDSSGQR
SQFTITPGSEQIR
VAMoxQDATAQMoxAMoxLQFISSGLPK
VAMoxSHFEPSEYIR
VAMoxSHFEPSEYIRYDLLEK
VAMSHFEPSEYIR
VAVPSTIHCcamDHLIEAQVGGEK
VAVPSTIHCcamDHLIEAQVGGEKDLR
VGLIGSCcamTNSSYEDMGR
VGLIGSCcamTNSSYEDMoxGR
WVVIGDENYGEGSSR
WVVIGDENYGEGSSREHAALEPR
YDLLEKNINIVR	
17	Mitochondrial acyl-CoA thioesterase 1 	Q9QYR9
	49,6	7	123	50	14	AHAVAQVDAWQQLQTFFHK
ATLFLPPEPGPFPGIIDLFGVGGGLLEYR
DGLLDVVEALQSPLVDKK
GFAVMoxALAYYNYDDLPK
GGELGLAMASFLK
GGELGLAMoxASFLK
GITAAVVINGSVAAVGNTISYKDETIPPVSLLR
GLAPEQPVTLR
SCcamWDEPLSIAVR
SEFYADEISKR
SHPEVKGPGIGLLGISK
SIETMoxHMoxEYFEEAVNYLR
SWARLFVPPPLSTGGR
YRADAGGELNLAR	
18	Acp1 protein 	Q9D358
	18,2	5,9	131	70	10	AKIELLGSYDPQK
IELLGSYDPQK
QITKEDFATFDYILCcamMoxDESNLR
QLIIEDPYYGNDSDFEVVYQQCcamLR
SPIAEAVFRK
SVLFVCcamLGNICcamR
VSDNWA	
19	Butyryl Coenzyme A synthetase 1; acetyl-Coenzyme A synthetase 3	Q91VA0
	64,8	6,6	119	34	15	AFIVLNPEFLSHDQEQLIK
AFIVLNPEFLSHDQEQLIKELQHHVK
AILPFDLQIIDEK
AILPFDLQIIDEKGNILPPNTEGYIGIR
AIVTTASLVPEVESVASECcamPDLK
ATIDEEGYIWFLGR
GDDVINASGYR
GNILPPNTEGYIGIR
KVEFVSELPK
SVTAPYKYPR
TANVFEQICcamGLQQGDHLALILPR
TGIIFMoxPGTTQLK
VIVEVLFKYPITQCcamLAAPGVYR
VPEWWLVTVGCcamMoxR
VPEWWLVTVGCcamMR	
20	Put. beta-actin (aa 27-375)	P60710
	39,2	5,7	236	73	29	DLYANTVLSGGTTMoxYPGIADR
DLYANTVLSGGTTMYPGIADR
DSYVGDEAQSKR
EITALAPSTMoxK
EKMoxTQIMoxFETFNTPAMoxYVAIQAVLSLYASGR
GYSFTTTAER
GYSFTTTAEREIVR
HQGVMoxVGMoxGQK
HQGVMoxVGMoxGQKDSYVGDEAQSK
HQGVMVGMGQK
HQGVMVGMGQKDSYVGDEAQSK
IKIIAPPER
ILTERGYSFTTTAER
IWHHTFYNELR
LCcamYVALDFEQEMoxATAASSSSLEK
LDLAGRDLTDYLMK
LDLAGRDLTDYLMoxK
MoxTQIMoxFETFNTPAMoxYVAIQAVLSLYASGR
MQKEITALAPSTMK
oxHQGVMVGMGQK
oxHQGVMVGMGQKDSYVGDEAQSK
oxMQKEITALAPSTMK
PRAVFPSIVGR
QEYDESGPSIVHR
QEYDESGPSIVHRK
SYELPDGQVITIGNER
TTGIVMDSGDGVTHTVPIYEGYALPHAILR
TTGIVMoxDSGDGVTHTVPIYEGYALPHAILR
VAPEEHPVLLTEAPLNPK	
21	Gamma-actin [Mus musculus]	P63260
	41	5,5	176	68	23	AVFPSIVGRPR
DLYANTVLSGGTTMoxYPGIADR
DLYANTVLSGGTTMYPGIADR
DSYVGDEAQSKR
GYSFTTTAER
GYSFTTTAEREIVR
HQGVMoxVGMoxGQK
HQGVMVGMGQK
ILTERGYSFTTTAER
IWHHTFYNELR
KDLYANTVLSGGTTMoxYPGIADR
KDLYANTVLSGGTTMYPGIADR
LCcamYVALDFEQEMATAASSSSLEK
LCcamYVALDFEQEMoxATAASSSSLEK
LDLAGRDLTDYLMK
LDLAGRDLTDYLMoxK
LVIDNGSGMoxCcamKAGFAGDDAPR
oxFRCcamPEALFQPSFLGMESCcamGIHETTFNSIMK
oxHQGVMVGMGQK
QEYDESGPSIVHR
QEYDESGPSIVHRK
SYELPDGQVITIGNER
TTGIVMDSGDGVTHTVPIYEGYALPHAILR
TTGIVMoxDSGDGVTHTVPIYEGYALPHAILR
VAPEEHPVLLTEAPLNPK	
22	Actr3 protein [Mus musculus]	Q99JY9
	47,4	5,5	314	60	32
	AEPEDHYFLLTEPPLNTPENR
DITYFIQQLLR
DITYFIQQLLRDR
DYEEIGPSICcamR
EFSIDVGYER
FMEQVIFKYLR
FMoxEQVIFKYLR
GVDDLDFFIGDEAIEKPTYATK
HGIVEDWDLMER
HGIVEDWDLMoxER
HIPIAGRDITYFIQQLLR
KDYEEIGPSICcamR
KEFSIDVGYER
LGYAGNTEPQFIIPSCcamIAIK
LGYAGNTEPQFIIPSCcamIAIKESAK
LKLSEELSGGR
LKPKPIDVQVITHHMoxQR
LKPKPIDVQVITHHMQR
LPACcamVVDCcamGTGYTK
NIVLSGGSTMFR
NIVLSGGSTMFRDFGR
NIVLSGGSTMoxFR
NIVLSGGSTMoxFRDFGR
QYTGVNAISKK
RPLYKNIVLSGGSTMoxFR
TLTGTVIDSGDGVTHVIPVAEGYVIGSCcamIK
TLTGTVIDSGDGVTHVIPVAEGYVIGSCcamIKHIPIAGR
VMoxKGVDDLDFFIGDEAIEKPTYATK
WPIRHGIVEDWDLMER
WPIRHGIVEDWDLMoxER
YLRAEPEDHYFLLTEPPLNTPENR
YSYVCcamPDLVK	
23	Aminoacylase 1 [Mus musculus]	Q99JW2
	45,8	5,7	207	64	24	AGFVLDEGLANPTDAFTVFYSER
AMNLTLEPEIFPAATDSR
AMoxNLTLEPEIFPAATDSR
AVGIPALGFSPMNR
AVGIPALGFSPMoxNR
DPESEHPSVTLFR
FIEDTAAEKLHK
GMoxELFVKRPEFQALR
ICcamTVQPNPDYGGAITFLEER
LEGGVAYNVVPATMoxSASFDFR
LEGGVAYNVVPATMSASFDFR
LQANPHLKEGAVTSVNLTK
MoxTPTDDSDPWWAAFSGACcamK
oxTIHMTFVPDEEVGGHKGMELFVK
QRLQANPHLK
SPWWVQVTSTGKPGHASR
SVSIQYLEAVR
TIHMoxTFVPDEEVGGHK
TIHMoxTFVPDEEVGGHKGMoxELFVK
TIHMTFVPDEEVGGHK
TPVLLHDHNER
TPVLLHDHNERLHEDIFLR
VAPDVDMoxKAFEK
VISSILAFREK	
24	Adipose differentiation-related protein	P43883
	46,7	6,2	150	57,2	18	HLGVMAGDIYSVFR
HLGVMoxAGDIYSVFR
KVEGFDMoxVQKPSNYER
KVEGFDMVQKPSNYER
LCcamSRAYHQALSR
LEPQIAVANTYACcamK
LPILNQPTSEIVASAR
MoxAAAVVDPQQSVVMoxR
MoxEERLPILNQPTSEIVASAR
NAASFKEVSDGVLTSSK
NLTQQLQTTCcamQTVLVNAQGLPQNIQDQAK
QKSQETISQLHSTVHLIEFAR
SEMoxLVDQYFPLTQEELEMoxEAK
SIGYDDTDESHCcamVEHIESR
SQETISQLHSTVHLIEFAR
TVTSAAMoxTSALPIIQK
VANLPLVSSTYDLVSSAYVSTK
VANLPLVSSTYDLVSSAYVSTKDQYPYLR	
25	Alcohol dehydrogenase 1 (class I) [Mus musculus]	P00329
	39,7	9,6	159	54	17	AAVLWELHKPFTIEDIEVAPPKAHEVR
DSVPKLVADFMoxAK
GKQIHNFISTSTFSQYTVVDDIAVAK
GTLREGTSR
HPESNFCcamSR
ICcamKHPESNFCcamSR
IDGASPLDKVCcamLIGCcamGFSTGYGSAVK
IKMoxVATGVCcamR
INEAFDLLR
KFPLDPLITHVLPFEK
LVADFMoxAK
MVATGVCcamR
QIHNFISTSTFSQYTVVDDIAVAK
TWKGAIFGGFK
VIPLFSPQCcamGECcamR
VTPGSTCcamAVFGLGGVGLSVIIGCcamK
VTPGSTCcamAVFGLGGVGLSVIIGCcamKAAGAAR	
26	Adenosine kinase [Mus musculus]	P55264
	40,1	5,7	194	62	20	AADAHVDAHYYEQNEQPTGTCcamAACcamITGGNR
AATFFGCcamIGIDKFGEILK
AGHYAASVIIR
AGHYAASVIIRR
ARVYYIAGFFLTVSPESVLK
EALMoxDVMoxPYVDILFGNETEAATFAR
FKVEYHAGGSTQNSMK
FKVEYHAGGSTQNSMoxK
HLDLERNWVLVEK
KAADAHVDAHYYEQNEQPTGTCcamAACcamITGGNR
LKVEAPQALSENVLFGMoxGNPLLDISAVVDK
NWVLVEKAR
RTGCcamTFPEKPDFH
SLVANLAAANCcamYK
SLVANLAAANCcamYKK
TGCcamTFPEKPDFH
VAQWLIQEPHK
VFTLNLSAPFISQFFK
VYYIAGFFLTVSPESVLK
YAAENNRVFTLNLSAPFISQFFK	
27	PREDICTED: agmatine ureohydrolase (agmatinase) [Mus musculus]	Q9BSE5
	38,3	8	108	51	16
	EAYQNVLAAGCcamIPLTLGGDQTITYPILQAVAK
EESLMoxLGAVNPSTGALPFQSLR
EHGPVGLVHVGAHTNTTDKPR
IREESLMLGAVNPSTGALPFQSLR
IREESLMoxLGAVNPSTGALPFQSLR
LPLQSSPEGLDAAFIGVPLDTGTSNRPGAR
SLVPLMAEVR
SLVPLMoxAEVR
SQGFRVVLAEDCcamWMK
SQGFRVVLAEDCcamWMoxK
SVDEGLLDSKR
TLDPYRYSR
VADLGNVNVNLYNLQDSCcamLLIR
VVLAEDCcamWMK
VVLAEDCcamWMoxK
VYHRTPFR	
28	S-adenosylhomocysteine hydrolase [Mus musculus]	P50247
	47,7	5,9	238	67	27	ALDIAENEMoxPGLMoxR
DGPLNMoxILDDGGDLTNLIHTK
DGPLNMoxILDDGGDLTNLIHTKYPQLLSGIR
EGNIFVTTTGCcamVDIILGR
EMoxYSASKPLKGAR
EMYSASKPLKGAR
GCcamAQALRGFGAR
GISEETTTGVHNLYK
HFEQMoxKDDAIVCcamNIGHFDVEIDVK
IAGCcamLHMoxTVETAVLIETLVALGAEVR
IAGCcamLHMTVETAVLIETLVALGAEVR
KALDIAENEMoxPGLMoxR
KLDEAVAEAHLGK
LTEKQAQYLGMoxPINGPFKPDHYR
oxALDIAENEMPGLMR
oxKALDIAENEMPGLMR
oxMMSNGILNVPAINVNDSVTKSK
QAQYLGMoxPINGPFKPDHYR
QAQYLGMoxPINGPFKPDHYRY
QAQYLGMPINGPFKPDHYRY
SKFDNLYGCcamR
VADIGLAAWGR
VADIGLAAWGRK
VAVVAGYGDVGKGCcamAQALR
VNIKPQVDRYWLK
WLNENAVEK
WSSCcamNIFSTQDHAAAAIAK	
29	Alpha-2-HS-glycoprotein [Mus musculus]	P29699
	37,3	6,1	70	47	12	ANLMHNLGGEEVSVACcamK
ANLMoxHNLGGEEVSVACcamK
AQNVPLPVSTLVEFVIAATDCcamTAK
CcamNLLAEKQHGFCcamK
ELACcamDDPEAEQVALLAVDYLNNHLLQGFK
EVTDPAKCcamNLLAEK
HAFSPVASVESASGETLHSPK
QLTEHAVEGDCcamDFHILK
QLTEHAVEGDCcamDFHILKQDGQFR
QVLNQIDKVK
VGQPGAAGPVSPMoxCcamPGR
VGQPGAAGPVSPMoxCcamPGRIR	
30	Adenylate kinase 2 [Mus musculus]	Q9WTP6
	25,6	6,7	89	61	14	AVLLGPPGAGKGTQAPK
EKLDSVIEFSIQDSLLIR
GIHCcamAIDASQTPDIVFASILAAFSK
LAENFCcamVCcamHLATGDMLR
LAENFCcamVCcamHLATGDMoxLR
LDSVIEFSIQDSLLIR
LEAYHTQTTPLVEYYR
LVSDEMoxVVELIEK
NGFLLDGFPR
NGFLLDGFPRTVR
NLETPSCcamKNGFLLDGFPR
oxQAEMLDDLMEK
RGIHCcamAIDASQTPDIVFASILAAFSK
TRLEAYHTQTTPLVEYYR	
31	Aldo-keto reductase family 1, member A4 (aldehyde reductase)	Q9JII6
	36,6	7,1	177	64	22	ALGLSNFNSR
AVPREELFVTSK
AWRHPDEPVLLEEPVVLALAEK
DAGHPLYPFNDPY
GLEVTAYSPLGSSDR
HHPEDVEPALR
HHPEDVEPALRK
HIDCcamASVYGNETEIGEALK
HIDCcamASVYGNETEIGEALKESVGSGK
HPDEPVLLEEPVVLALAEK
ILQNIQVFDFTFSPEEMK
ILQNIQVFDFTFSPEEMoxKQLDALNK
LWNTKHHPEDVEPALR
MoxPLIGLGTWK
TASSVLLHTGQK
TLADLQLEYLDLYLMHWPYAFER
TLADLQLEYLDLYLMoxHWPYAFER
YDSTHYKETWK
YIVPMITVDGK
YIVPMITVDGKR
YIVPMoxITVDGK
YIVPMoxITVDGKR	
32	Akr1c12 protein [Mus musculus]	Q91X42
	37	6,1	135	67	14	ENLQVFEFQLSPEDMoxK
GIVPLAQSFKENEMoxR
HVDTAYAYQVEEEIGQAIQSK
LNDGHLIPALGFGTYKPK
LWCcamGCcamFRPELVKPALEK
QHYVKLNDGHLIPALGFGTYKPK
SIGVSNFNHR
SKDIVLVAYGALGTQR
SLEAACcamLALDVGYR
SPALIALRYLFQR
TLDGLNKNFR
YKEWVDQNSPVLLNDPVLCcamDVAK
YKPVCcamNQVECcamHLYLNQSK
YLPAEFLADHPEYPFSEEY	
33	Aldo-keto reductase family 1 member C13	Q8VC28
	37,1	6,5	225	64	16	ENLQVFGFQLSPEDMoxK
GIVPLAQSFKENEMoxR
LLDYCcamESKDIVLVAYGALGTQR
LNDGHLIPALGFGTYKPK
LNDGHLIPALGFGTYKPKEVPK
LQLDYVDLYIMoxHYPVPMoxK
QHCcamVKLNDGHLIPALGFGTYKPK
SIGVSNFNHR
SKSLEAACcamLALDVGYR
SLEAACcamLALDVGYR
SPALIALRYLIQR
TLDGLNKNFR
YKEWVDQNSPVLLNDPVLCcamDVAK
YKPVCcamNQVECcamHLYLNQR
YKPVCcamNQVECcamHLYLNQRK
YLPAEFLVDHPEYPFVEEY	
34	Aldo-keto reductase family 1, member D1 [Mus musculus]	Q8VCX1
	37,3	6,3	192	61	18	ALKLDYIDLYIIELPMoxAFKPGK
EIYPRDENGR
FCcamQQHDIVIVAHSPLGTCcamR
HIDGAYVYHNEHEVGEAIR
HIDGAYVYHNEHEVGEAIREK
IIYDKTNLCcamATWEALEACcamK
LLKFCcamQQHDIVIVAHSPLGTCcamR
LWNTEHVPSMoxVLPALER
LWNTEHVPSMVLPALER
NPSWVNVSSPPLLNDELLTSLGK
NPSWVNVSSPPLLNDELLTSLGKK
QLELILNKPGLK
REEIFYCcamGK
RQLELILNKPGLK
SLGVSNFNRR
TQAQIVLRFNIQR
YKPVTNQVECcamHPYFTQTK
YNKTQAQIVLR	
35	Aminolevulinate, delta-, dehydratase; delta-aminolevulinate dehydratase [Mus musculus]	P10518
	36	6,2	191	57	26	AGADIIITYFAPQLLK
AGCcamQVVAPSDMoxMoxDGR
AGCcamQVVAPSDMoxMoxDGRVEAIK
CcamVLIFGVPSR
CcamVLIFGVPSRVPK
CcamYQLPPGAR
DAAQSSPAFGDR
DAAQSSPAFGDRR
DEQGSAADSEDSPTIEAVR
DIQEGADMoxLMoxVKPGLPYLDMoxVR
FASCcamFYGPFR
GLALRAVAR
HGLGNRVSVMoxSYSAK
HGLGNRVSVMSYSAK
LAEVALAYAK
oxDIQEGADMLMVKPGLPYLDMVR
oxDIQEGADMLMVKPGLPYLDMVR
QRLAEVALAYAK
RAGADIIITYFAPQLLK
RCcamYQLPPGAR
TAVLETMoxTAFR
TAVLETMoxTAFRR
TAVLETMTAFRR
VPKDEQGSAADSEDSPTIEAVR
YGVNQLEEMLRPLVEAGLR
YGVNQLEEMoxLRPLVEAGLR	
36	Albumin [Rattus norvegicus]	P02770
	68,7	6	371	62	33	AADKDNCcamFATEGPNLVAR
AETFTFHSDICcamTLPDKEK
APQVSTPTLVEAAR
CcamCcamAEGDPPACcamYGTVLAEFQPLVEEPK
CcamCcamSGSLVER
CcamCcamTLPEAQR
CcamCcamTLPEAQRLPCcamVEDYLSAILNR
CcamPYEEHIKLVQEVTDFAK
DVFLGTFLYEYSR
EAHKSEIAHR
ECcamCcamHGDLLECcamADDRAELAK
FKDLGEQHFK
FPNAEFAEITK
GLVLIAFSQYLQK
HPDYSVSLLLR
LPCcamVEDYLSAILNR
LQACcamCcamDKPVLQK
LVQEVTDFAK
MoxSQRFPNAEFAEITK
NECcamFLQHK
NYAEAKDVFLGTFLYEYSR
QEPERNECcamFLQHK
RHPDYSVSLLLR
RHPYFYAPELLYYAEK
RPCcamFSALTVDETYVPK
SIHTLFGDKLCcamAIPK
SQCcamLAETEHDNIPADLPSIAADFVEDKEVCcamK
TVMGDFAQFVDK
TVMoxGDFAQFVDK
TVMoxGDFAQFVDKCcamCcamK
YMoxCcamENQATISSK
YNEVLTQCcamCcamTESDKAACcamLTPK
YTQKAPQVSTPTLVEAAR	
37	Albumin 1; serum albumin variant [Mus musculus]	P07724
	68,7	5,7	444	65	41	AETFTFHSDICcamTLPEKEK
APQVSTPTLVEAAR
ATAEQLKTVMoxDDFAQFLDTCcamCcamK
CcamCcamAEANPPACcamYGTVLAEFQPLVEEPK
CcamCcamAEANPPACcamYGTVLAEFQPLVEEPKNLVK
CcamCcamSGSLVER
CcamCcamTLPEDQRLPCcamVEDYLSAILNR
CcamSSMoxQKFGER
CcamSYDEHAK
CcamSYDEHAKLVQEVTDFAK
DVFLGTFLYEYSR
EAHKSEIAHR
ENPTTFMGHYLHEVAR
ENPTTFMoxGHYLHEVAR
ESCcamLTPK
GLVLIAFSQYLQK
HPDYSVSLLLR
KYEATLEK
LPCcamVEDYLSAILNR
LQTCcamCcamDKPLLK
LQTCcamCcamDKPLLKK
LSQTFPNADFAEITK
LSQTFPNADFAEITKLATDLTK
LVQEVTDFAK
NYAEAKDVFLGTFLYEYSR
QEPERNECcamFLQHK
QTALAELVK
RHPDYSVSLLLR
RPCcamFSALTVDETYVPK
RPCcamFSALTVDETYVPKEFK
SLHTLFGDKLCcamAIPNLR
TCcamVADESAANCcamDK
TNCcamDLYEKLGEYGFQNAILVR
TPVSEHVTK
TVMDDFAQFLDTCcamCcamK
TVMoxDDFAQFLDTCcamCcamK
VCcamLLHEKTPVSEHVTK
YMoxCcamENQATISSK
YNDLGEQHFK
YNDLGEQHFKGLVLIAFSQYLQK
YTQKAPQVSTPTLVEAAR	
38	Aldehyde dehydrogenase family 1, subfamily A1; aldehyde dehydrogenase 1, liver cytosolic (class 1); alcohol dehydrogenase family 1, subfamily A2; alcohol dehydrogenase family 1, subfamily A1 [Mus musculus]	P24549
 	54,5	7,9	167	50	23	ANNTTYGLAAGLFTK
DRLLLATMEALNGGK
DRLLLATMoxEALNGGK
ELGEHGLYEYTELK
ELGEHGLYEYTELKTVAMoxK
EQHDKILDLIESGK
GFFVQPTVFSNVTDEMoxR
GFFVQPTVFSNVTDEMR
IAKEEIFGPVQQIMK
IAKEEIFGPVQQIMoxK
IFINNEWHNSVSGK
IFVEESVYDEFVK
IFVEESVYDEFVKR
IGPALSCcamGNTVVVKPAEQTPLTALHLASLIK
KFPVLNPATEEVICcamHVEEGDK
LADLMoxERDR
LLLATMEALNGGKVFANAYLSDLGGCcamIK
LLLATMoxEALNGGK
QAFQIGSPWR
RANNTTYGLAAGLFTK
VFANAYLSDLGGCcamIK
YCcamAGWADKIHGQTIPSDGDIFTYTR
YVLGNPLTPGINQGPQIDKEQHDK	
39	Aldehyde dehydrogenase 1 family, member B1 [Mus musculus]	Q9CZS1
	57,6	6,4	229	58	26	EAGFPPGVVNIITGYGPTAGAAIAQHMoxDVDK
EEIFGPVQPLFK
ELGEDGLRAYTEVK
ESGNGRELGEDGLR
GFFIKPTVFGDVQDGMoxR
GFFIKPTVFGDVQDGMR
HEPVGVCcamGQIIPWNFPLVMoxQGWK
IAKEEIFGPVQPLFK
KTFPTVNPTTGEVIGHVAEGDR
LADLVERDR
LAPALATGNTVVMK
LAPALATGNTVVMoxK
LFINNEWHDAVSK
LFINNEWHDAVSKK
TFPTVNPTTGEVIGHVAEGDR
TFPTVNPTTGEVIGHVAEGDRADVDLAVK
TFVEESIYR
TFVEESIYREFLER
TIPMDGEHFCcamFTR
TIPMoxDGEHFCcamFTR
VAEQTPLSALYLASLIK
VAFTGSTEVGHLIQK
VGNPFELDTQQGPQVDKEQFER
VYLASLETLDNGKPFQESYVLDLDEVIK
YFAGWADKWHGK
YGLAAAVFTR	
40	10-formyltetrahydrofolate dehydrogenase Unnamed protein product [Mus musculus]	Q8R0Y6
	18,6	5,5	124	69	11	ALYVSDKLQAGTVFVNTYNK
ANATEFGLASGVFTR
DLGEAALNEYLR
DTNHGPQNHEAHLR
EESFGPIMIISR
EESFGPIMoxIISR
FADGDVDAVLSR
IGNPLDRDTNHGPQNHEAHLR
KLVEYCcamQR
QSGFGKDLGEAALNEYLR
TDVAAPFGGFK	
41	Fthfd protein [Mus musculus]	P28037
	98,7	5,6	375	55	41	ADPLGLEAEKDGVPVFK
AGLILFGNDDR
AGLILFGNDDRMLLVK
AGLILFGNDDRMoxLLVK
ANATEFGLASGVFTR
DGVPVFKFPR
DLGEAALNEYLR
DTNHGPQNHEAHLR
EAFENGLWGK
ECcamDVLPDDTVSTLYNR
EESFGPIMIISR
EESFGPIMoxIISR
ETAMoxINWDQPAEAIHNWIR
FADGDVDAVLSR
FLFPEGIKGMoxVQAVR
GASAINWTLIHGDKK
GMVQAVRLIAEGTAPR
GNDKVPGAWTEACcamGQK
GSASSALELTEEELATAEAVR
GVVNILPGSGSLVGQR
HGSIIYHPSLLPR
IAVIGQSLFGQEVYCcamQLR
IGFTGSTEVGKHIMoxK
IGNPLDRDTNHGPQNHEAHLR
ILPNVPEVEDSTDFFK
IQGATIPINQARPNR
KLVEYCcamQR
LFVEDSIHDQFVQK
LFVEDSIHDQFVQKVVEEVGK
LRGEDGESECcamVINYVEK
NIQLEDGKMoxMoxPASQFFK
QSGFGKDLGEAALNEYLR
RPQPEEGATYEGIQK
RPQPEEGATYEGIQKK
SPLIIFADCcamDLNK
SSWMRILPNVPEVEDSTDFFK
TAACcamLAAGNTVVIKPAQVTPLTALK
THVGMoxSIQTFR
THVGMSIQTFR
YFAGWCcamDKIQGATIPINQARPNR
YQALGAELNVLPFCcamSQFIPMoxEVINAPR	
42	Aldehyde dehydrogenase 2, mitochondrial [Mus musculus]	P47738
	56,5	7,4	225	61	30	AAFQLGSPWR
AAFQLGSPWRR
ANDSKYGLAAAVFTK
DGMoxTIAKEEIFGPVMoxQILK
DGMTIAKEEIFGPVMQILK
EAGFPPGVVNIVPGFGPTAGAAIASHEGVDK
EEIFGPVMoxQILK
ELGEYGLQAYTEVK
FKTIEEVVGR
GYFIQPTVFGDVK
GYFIQPTVFGDVKDGMoxTIAK
LADLIERDR
LGPALATGNVVVMK
LGPALATGNVVVMoxK
LLCcamGGGAAADRGYFIQPTVFGDVK
MoxSGSGRELGEYGLQAYTEVK
MSGSGRELGEYGLQAYTEVK
oxDGMTIAKEEIFGPVMQILK
SRVVGNPFDSR
TEQGPQVDETQFK
TEQGPQVDETQFKK
TFPTVNPSTGEVICcamQVAEGNKEDVDK
TFVQENVYDEFVERSVAR
TIPIDGDFFSYTR
TYLAALETLDNGKPYVISYLVDLDMoxVLK
VAEQTPLTALYVANLIK
VAFTGSTEVGHLIQVAAGSSNLK
VAFTGSTEVGHLIQVAAGSSNLKR
VVGNPFDSRTEQGPQVDETQFK
YYAGWADKYHGK	
43	Aldh4a1 protein [Mus musculus]	Q8CHT0
	60,3	8,2	192	49	23	AADMoxLSGPR
AIVQEATR
ASGTNDKPGGPHYILR
ETHKPLGDWR
FAVELEGEQPISVPPSTNHTVYR
FCcamYADK
LAGECcamGGKNFHFVHSSADVDSVVSGTLR
LVDSTTSYGLTGAVFAQDK
NAAGNFYINDK
NAAGNFYINDKSTGSVVGQQPFGGAR
NFHFVHSSADVDSVVSGTLR
QVAQNLDR
QVAQNLDRFR
SLWPQIK
SSPSLSILAGGQCcamNESVGYYVEPCcamIIESK
STGSVVGQQPFGGAR
TVIQAEIDAAAELIDFFR
VANEPILAFSQGSPER
VANEPILAFSQGSPERDALQK
VGDPAEDFGTFFSAVIDAK
WKHTSSLK
WLEHAR
WTSPQVIK	
44	Aldehyde dehydrogenase family 6, subfamily A1 [Mus musculus]	Q02253
	57,9	8,3	112	54	20	AFPAWADTSILSR
AFPAWADTSILSRQQVLLR
AISFVGSNQAGEYIFER
CcamMoxALSTAILVGEAKK
DMoxDLYSYR
EGASILLDGRR
ERVCcamNLIDSGTK
GYENGNFVGPTIISNVKPSMoxTCcamYK
IVNDNPYGNGTAIFTTNGATAR
LFIDGKFVESK
NHGVVMoxPDANKENTLNQLVGAAFGAAGQR
oxMAAAVAAAAAMR
oxMAAAVAAAAAMRSR
QGIQFYTQLK
SDKWIDIHNPATNEVVGR
SSFRGDTNFYGK
TITSQWKEEDATLSSPAVVMoxPTMoxGR
TLADAEGDVFR
VNAGDQPGADLGPLITPQAK
YQQLIKENLK	
45	Aldehyde dehydrogenase family 7, member A1; aldehyde dehydrogenase 7 family, member A1 Mus musculus	Q9DBF1
	55,6	5,9	264	58	32	AWNIWADIPAPK
DERVNLLSFTGSTQVGK
DLGRIFR
EKIQLLGR
ESGSDAWKQYMoxR
EVALMoxVQER
EVALMVQER
GAPTTSLVSVAVTK
GEVITTYCcamPANNEPIAR
GSDCcamGIVNVNIPTSGAEIGGAFGGEK
GSDCcamGIVNVNIPTSGAEIGGAFGGEKHTGGGR
IFRWLGPK
IGDAFREK
IIAQVLEDNLLPGAICcamSLVCcamGGADIGTTMoxAR
ILVEGIGEVQEYVDVCcamDYAAGLSR
KIGDAFR
LFLHESIHNEVVDR
LFLHESIHNEVVDRLR
LRSAYSQIR
QASLKDYEETIGK
QAVSMFVR
QAVSMoxFVR
QGGTVVYGGK
QGLSSSIFTKDLGR
RLFLHESIHNEVVDR
RSTCcamTINYSTSLPLAQGIK
SAYSQIR
STCcamTINYSTSLPLAQGIK
STCcamTINYSTSLPLAQGIKFQ
VGNPWDPNILYGPLHTK
VNLLSFTGSTQVGK
VNLLSFTGSTQVGKEVALMoxVQER	
46	Aldehyde dehydrogenase 9, subfamily A1; aldehyde dehydrogenase 9A; EST AA139417 [Mus musculus]	Q9JLJ2
	53,5	6,4	187	51	19	AGAPPGLFNVVQGGAATGQFLCcamHHR
AGAPPGLFNVVQGGAATGQFLCcamHHREVAK
ANDTTFGLAAGVFTR
ENGRVTIEYYSQLK
GALMANFLTQGQVCcamCcamNGTR
GALMoxANFLTQGQVCcamCcamNGTR
GGARVEPVDASGTEK
GVKPITLELGGKSPLIIFSDCcamNMENAVK
ISFTGSVPTGVK
ISFTGSVPTGVKIMoxEMoxSAK
LGDPLLEDTR
LGDPLLEDTRMoxGPLINAPHLER
MoxGPLINAPHLER
MoxGPLINAPHLERVLGFVK
REPLGVCcamVGIGAWNYPFQIACcamWK
SGLERCcamQVLLEAAR
VAAELQAGTCcamYINNYNVSPVELPFGGYKK
VIATFACcamSGEKEVNLAVENAK
VTIEYYSQLK	
47	ALDOB_RAT Fructose-bisphosphate aldolase B (Liver-type aldolase)	Q91Y97
	39,6	8,7	134	54	18	ALNDHHVYLEGTLLKPNMLTAGHACcamTKK
ALQASALAAWGGK
ATQEAFMoxKR
CcamPLPRPWK
CcamPLPRPWKLSFSYGR
DGVDFGKWR
ETTIQGLDGLSER
GILAADESVGTMGNR
GILAADESVGTMoxGNR
GILAADESVGTMoxGNRLQR
GIVVGIKLDQGGAPLAGTNK
ISDQCcamPSSLAIQENANALAR
IVANGKGILAADESVGTMoxGNR
KYTPEQVAMATVTALHR
LDQGGAPLAGTNKETTIQGLDGLSER
TVPAAVPSICcamFLSGGMoxSEEDATLNLNAIYR
YTPEQVAMATVTALHR
YTPEQVAMoxATVTALHR	
48	Aldolase 2, B isoform [Mus musculus]	P00884
	39,5	8,5	236	71	27	ALNDHHVYLEGTLLKPNMoxVTAGHACcamTK
ALNDHHVYLEGTLLKPNMVTAGHACcamTK
ALQASALAAWGGK
AMANCcamQAAQGQYVHTGSSGAAATQSLFTASYTY
AMoxANCcamQAAQGQYVHTGSSGAAATQSLFTASYTY
ATQEAFMKR
ATQEAFMoxKR
CcamPLPRPWKLSFSYGR
DSQGNLFR
ELLFSVDNSISQSIGGVILFHETLYQK
ELSEIAQR
ETTIQGLDGLSER
GILAADESVGTMGNR
GILAADESVGTMoxGNR
GIVVGIKLDQGGAPLAGTNK
IADQCcamPSSLAIQENANALAR
KATQEAFMK
KATQEAFMoxK
KYTPEQVAMATVTALHR
KYTPEQVAMoxATVTALHR
LDQGGAPLAGTNKETTIQGLDGLSER
LSFSYGR
QFRELLFSVDNSISQSIGGVILFHETLYQK
TVPAAVPGICcamFLSGGMoxSEEDATLNLNAINR
TVPAAVPGICcamFLSGGMSEEDATLNLNAINR
YTPEQVAMATVTALHR
YTPEQVAMoxATVTALHR	
49	Anxa3	O35639
	36,4	5,4	160	64	17	ALLTLADGRR
DIEDSIKGELSGHFEDLLLAIVHCcamAR
EISQAYYTVYKK
GAGTDEFTLNR
GLGTDEKTLINILTER
GTGTDEDALIEILTTR
GTIKDYPGFSPSVDAEAIR
HYGYSLYSAIQSDTSGDYR
HYGYSLYSAIQSDTSGDYRTVLLK
LHQALKGAGTDEFTLNR
NTPAFLAERLHQALK
SEIDLLDIRHEFK
SFPQLKLTFDEYR
SLGDDISSETSGDFR
SMKGTGTDEDALIEILTTR
SMoxKGTGTDEDALIEILTTR
TLINILTER
WGTDEDKFTEVLCcamLR	
50	Annexin A4; annexin IV [Mus musculus]	P97429
	36	5,3	271	63	28	AASGFNATEDAQTLR
AASGFNATEDAQTLRK
AEIDMLDIR
AEIDMoxLDIR
AMoxKGAGTDEGCcamLIEILASR
AMoxKGLGTDEDAIIGILAYR
DIEQSIKSETSGSFEDALLAIVK
FLSILCcamSR
GAGTDEGCcamLIEILASR
GLGTDDNTLIR
GLGTDEDAIIGILAYR
GLGTDEDAIIGILAYRNTAQR
INQTYQQQYGR
NHLLHVFDEYKR
NRNHLLHVFDEYK
NTAQRQEIR
RINQTYQQQYGR
SETSGSFEDALLAIVK
SETSGSFEDALLAIVKCcamMoxR
SKPSYFAER
SKPSYFAERLYK
SLEEDICcamSDTSFMFQR
SLEEDICcamSDTSFMoxFQR
SLYSFIKGDTSGDYR
SMoxKGLGTDDNTLIR
TPEEIRR
WGTDEVK
WGTDEVKFLSILCcamSR	
51	Annexin A5; annexin V [Mus musculus]	P48036
	35,8	4,8	268	76	30	ALLLLCcamGGEDD
AMKGLGTDEDSILNLLTSR
AMoxKGLGTDEDSILNLLTSR
ETSGNLEQLLLAVVK
ETSGNLEQLLLAVVKSIR
GAGTDDHTLIR
GAGTDDHTLIRVVVSR
GAGTDEKVLTEIIASR
GLGTDEDSILNLLTSR
GTVTDFPGFDGR
GTVTDFPGFDGRADAEVLR
KNFATSLYSMoxIK
LIVAMMKPSR
LYDAYELKHALK
NFATSLYSMIK
oxLIVAMMKPSR
QEIAQEFKTLFGR
QVYEEEYGSNLEDDVVGDTSGYYQR
SEIDLFNIR
SEIDLFNIRK
SIPAYLAETLYYAMK
SIPAYLAETLYYAMKGAGTDDHTLIR
SIPAYLAETLYYAMoxK
SIPAYLAETLYYAMoxKGAGTDDHTLIR
SNAQRQEIAQEFK
TPEELSAIKQVYEEEYGSNLEDDVVGDTSGYYQR
VFDKYMoxTISGFQIEETIDR
VFDKYMTISGFQIEETIDR
VVVSRSEIDLFNIR
WGTDEEKFITIFGTR	
52	Anxa7 protein [Mus musculus]	Q07076
	49,9	5,7	149	49	28	AMKGFGTDEQAIVDVVSNR
AMoxKGFGTDEQAIVDVVSNR
AMoxQGAGTQERVLIEILCcamTR
CcamYQLEFGRDLEK
DLLSSVSREFSGYVESGLK
EFSGYVESGLKTILQCcamALNRPAFFAER
GFGTDEQAIVDVVSNR
IVVTRSEIDLVQIK
LGTDESCcamFNMILATR
LGTDESCcamFNMoxILATR
LLVSMCcamQGNRDER
LLVSMoxCcamQGNRDER
LYQAGEGRLGTDESCcamFNMILATR
LYQAGEGRLGTDESCcamFNMoxILATR
LYYSMKGAGTDDSTLVR
LYYSMoxKGAGTDDSTLVR
MANRDLLSSVSR
MoxANRDLLSSVSR
QSVNHQMAQEDAQR
QSVNHQMoxAQEDAQR
SDTSGHFER
SFPQLKATMEAYSR
SFPQLKATMoxEAYSR
SNDQRQQIK
TILQCcamALNRPAFFAER
TLSTMIASDTSGDYRK
TLSTMoxIASDTSGDYRK
TNQEIRDIVR	
53	Apoa4 protein [Mus musculus]	P06728
	45	5,3	224	76	29	ALVQQLEQFR
ALVQQLEQFRQQLGPNSGEVESHLSFLEK
ATIDQNLEDLRR
DRMoxMoxPHANK
FLKAAVLTLALVAITGTR
KGSPDQPQALPLPEQAQEQAQEQAQEQVQPKPLES
LGDASTYADGVHNK
LNHQMEGLAFQMK
LNHQMoxEGLAFQMoxK
LNHQMoxEGLAFQMoxKK
LQEHLKPYAVDLQDQINTQTQEMK
LQEHLKPYAVDLQDQINTQTQEMoxK
LVPFVVQLSGHLAQETER
MoxMoxPHANKVTQTFGENMoxQK
MoxQTTIKENVDNLHTSMoxMoxPLATNLK
NLAPLVEDVQSK
NMEELKGHLTPR
NMoxEELKGHLTPR
oxLNHQMEGLAFQMK
oxLNHQMEGLAFQMKK
oxTVEPMGEMFNK
QLEQQVEEFRR
QQLGPNSGEVESHLSFLEK
SLAPLTVGVQEK
TDVTQQLSTLFQDK
TDVTQQLSTLFQDKLGDASTYADGVHNK
TVEPMoxGEMoxFNK
VKGNTEGLQK
VNSFMoxSTLEK	
54	Precursor Apoprotein A-I [Mus musculus]	Q00623
	30,6	5,6	180	65	22	AQSVIDKASETLTAQ
ARPALEDLR
DFANVYVDAVKDSGR
DFWDNLEKETDWVR
ETDWVR
HSLMoxPMoxLETLK
HSLMPMLETLK
LAELKSNPTLNEYHTR
LQELQGR
LSPVAEEFR
LSPVAEEFRDR
MRTHVDSLR
QEMoxNKDLEEVK
QKLQELQGR
TQLAPHSEQMoxR
TQLAPHSEQMoxRESLAQR
TQLAPHSEQMR
TQLAPHSEQMRESLAQR
VAPLGAELQESARQK
VKDFANVYVDAVK
VQPYLDEFQKK
WKEDVELYR	
55	Apoe [Mus musculus]	P08226
	35,9	5,5	220	54	24	AGAREGAER
AQAFGDRIR
ELEEQLGPVAEETR
ELEEQLGPVAEETRAR
ERLGPLVEQGR
FWDYLR
GRLEEVGNQAR
GVSAIRER
LGADMEDLR
LGADMEDLRNR
LGADMoxEDLRNR
LGKEVQAAQAR
LGPLVEQGR
LGQYRNEVHTMoxLGQSTEEIR
LKGWFEPIVEDMHR
LKGWFEPIVEDMoxHR
LQAEIFQAR
NEVHTMoxLGQSTEEIR
NEVHTMoxLGQSTEEIRAR
QWANLMoxEK
SKMEEQTQQIR
SKMoxEEQTQQIR
TANLGAGAAQPLR
TANLGAGAAQPLRDR	
56	Acidic ribosomal phosphoprotein P0 [Mus musculus]	P14869
	34,2	5,7	216	58	17	AFLADPSAFAAAAPAAAATTAAPAAAAAPAK
AGAIAPCcamEVTVPAQNTGLGPEK
AGAIAPCcamEVTVPAQNTGLGPEKTSFFQALGITTK
AIRGHLENNPALEK
ATWKSNYFLK
CcamFIVGADNVGSK
CcamFIVGADNVGSKQMoxQQIR
GHLENNPALEK
GHLENNPALEKLLPHIR
GTIEILSDVQLIK
IIQLLDDYPK
ISRGTIEILSDVQLIK
NVASVCcamLQIGYPTVASVPHSIINGYKR
TSFFQALGITTK
TSFFQALGITTKISR
VLALSVETEYTFPLTEK
VLALSVETEYTFPLTEKVK	
57	Arginase 1, liver [Mus musculus]	Q61176
	34,8	6,6	306	82	28	ANEELAGVVAEVQK
ANEELAGVVAEVQKNGR
DHGDLAFVDVPNDSSFQIVK
DHGDLAFVDVPNDSSFQIVKNPR
DIVYIGLRDVDPGEHYIIK
EGLYITEEIYK
EGLYITEEIYKTGLLSGLDIMEVNPTLGK
EGLYITEEIYKTGLLSGLDIMoxEVNPTLGK
EGNHKPGTDYLKPPK
GGVEKGPAALR
GKFPDVPGFSWVTPCcamISAK
KRPIHLSFDVDGLDPAFTPATGTPVLGGLSYR
LKETEYDVR
NGRVSVVLGGDHSLAVGSISGHAR
RPIHLSFDVDGLDPAFTPATGTPVLGGLSYR
SLEIIGAPFSK
SLEIIGAPFSKGQPR
STVNTAVALTLACcamFGTQR
STVNTAVALTLACcamFGTQREGNHKPGTDYLKPPK
SVGKANEELAGVVAEVQK
TAEEVKSTVNTAVALTLACcamFGTQR
TGLLSGLDIMoxEVNPTLGK
VMEETFSYLLGR
VMEETFSYLLGRK
VMoxEETFSYLLGR
VMoxEETFSYLLGRK
VSVVLGGDHSLAVGSISGHAR
YFSMoxTEVDKLGIGK	
58	Rho GDP-dissociation inhibitor 1
Unnamed protein product [Mus musculus]	Q99PT1
	22,9	5..1	149	70	15	AEEYEFLTPMEEAPK
AEEYEFLTPMoxEEAPK
AEEYEFLTPMoxEEAPKGMoxLAR
FTDDDKTDHLSWEWNLTIK
IDKTDYMoxVGSYGPR
IDKTDYMVGSYGPR
LTLVCcamSTAPGPLELDLTGDLESFK
LTLVCcamSTAPGPLELDLTGDLESFKK
QSFVLKEGVEYR
SIQEIQELDKDDESLR
TDYMVGSYGPR
VAVSADPNVPNVIVTR
VNREIVSGMK
VNREIVSGMoxK
YIQHTYRK	
59	Actin related protein 2/3 complex, subunit 5 [Mus musculus]	Q9CPW4
	16,3	5,2	124	58	8	ALAAGGVGSIVR
ALAAGGVGSIVRVLTAR
AVQSLDKNGVDLLMoxK
GFESPSDNSSAVLLQWHEK
QGNMoxTAALQAALK
QGNMoxTAALQAALKNPPINTK
VLISFKANDIEK
YIYKGFESPSDNSSAVLLQWHEK	
60	Arsenite methyltransferase; Methyltransferase Cyt19 [Mus musculus]	Q91WU5
	41,8	5,7	133	54	14	AKPVPSYIRESLQNVHEDVSSR
DADEIHKDVQNYYGNVLK
DCcamYVLSQLVGEK
DLAIIAQKIGFCcamPPR
FAPDFLFTPVDASLPAPQGR
FGFQAPNVTFLHGR
GHVTGIDMoxTK
HGGELYFSDVYASLEVPEDIKSHK
ILDLGSGSGRDCcamYVLSQLVGEK
LVTADIITVENKELEGVLGDCcamR
MoxAASRDADEIHK
VLWGECcamLGGALYWK
YYGCcamGLTVPER
YYGCcamGLTVPERLENCcamR	
61	Argininosuccinate synthetase 1; arginosuccinate synthetase 1 [Mus musculus]	P16460
	46,6	8,5	175	57	24	EGAKYVSHGATGK
ESPLSLYNEELVSMNVQGDYEPIDATGFININSLR
FAELVYTGFWHSPECcamEFVR
FELTCcamYSLAPQIK
GIYETPAGTILYHAHLDIEAFTMoxDR
GNDQVRFELTCcamYSLAPQIK
GRNDLMEYAK
GRNDLMoxEYAK
HGVGRIDIVENR
IDIVENRFIGMK
IDIVENRFIGMoxK
MoxPEFYNRFK
QHGIPIPVTPK
QVEIAQREGAK
TQDPAKAPNSPDVLEIEFK
TTSLELFMoxYLNEVAGK
TTSLELFMoxYLNEVAGKHGVGR
TTSLELFMYLNEVAGK
TTSLELFMYLNEVAGKHGVGR
VIAPWRMoxPEFYNR
VIAPWRMPEFYNR
VQVSVFKGQVYILGR
YLLGTSLARPCcamIAR
YVSHGATGKGNDQVR	
62	5-aminoimidazole-4-carboxamide ribonucleotide formyltransferase/IMP cyclohydrolase [Mus musculus]	Q9CWJ9
	64,1	6,3	223	52	21	AFTHTAQYDEAISDYFR
APSQLALFSVSDKTGLVEFAR
DAGLAVRDVSELTGFPEMoxLGGR
DGQVIGIGAGQQSR
DVSELTGFPEMLGGR
DVSELTGFPEMoxLGGR
EVSDGIVAPGYEEEALK
EVSDGIVAPGYEEEALKILSK
GAVDIPAAASFK
HVSPAGAAVGVPLSEDEAR
LAGDKANSWWLR
LDFNLVRVVVCcamNLYPFVK
MoxSSFGDFVALSDICcamDVPTAK
SGVAYIVAPSGSTADKVVIEACcamDELGIVLAHTDLR
SLASLGLSLVASGGTAK
TLHPAVHAGILAR
TVASPDVTVEAAVEQIDIGGVTLLR
VCcamMoxVYDLYPTLTPLAVAYAR
VCcamMVYDLYPTLTPLAVAYAR
VKTLHPAVHAGILAR
VVVCcamNLYPFVK
YGMoxNPHQTPAQLYTLKPK	
63	ATP5A1 protein [Homo sapiens]	P25705
	60,8	9,1	184	49	25	AVDSLVPIGR
DNGKHALIIYDDLSK
EAYPGDVFYLHSR
EIVTNFLAGFEA
ELIIGDRQTGK
EVAAFAQFGSDLDAATQQLLSR
GIRPAINVGLSVSR
HALIIYDDLSK
ILGADTSVDLEETGR
ILGADTSVDLEETGRVLSIGDGIAR
ISVREPMoxQTGIK
ISVREPMQTGIK
LYCcamIYVAIGQKR
NFHASNTHLQK
NVQAEEMVEFSSGLK
QGQYSPMAIEEQVAVIYAGVR
QGQYSPMoxAIEEQVAVIYAGVR
QVAGTMKLELAQYR
TGAIVDVPVGEELLGR
TGTAEMSSILEER
TSIAIDTIINQK
TSIAIDTIINQKR
VGLKAPGIIPR
VLSIGDGIAR
VVDALGNAIDGKGPIGSK	
64	ATP synthase, H+ transporting, mitochondrial F1 complex, alpha subunit, isoform 1 [Mus musculus]	Q03265
	59,8	9,2	215	62	33	AVDSLVPIGRGQR
DNGKHALIIYDDLSK
EAYPGDVFYLHSR
ELIIGDRQTGK
EVAAFAQFGSDLDAATQQLLSR
FENAFLSHVISQHQSLLGNIR
GIRPAINVGLSVSR
GMSLNLEPDNVGVVVFGNDK
GQRELIIGDR
HALIIYDDLSK
ILGADTSVDLEETGRVLSIGDGIAR
ISVREPMoxQTGIK
ISVREPMQTGIK
ITKFENAFLSHVISQHQSLLGNIR
LKEIVTNFLAGFEP
LSVRVAAAVAR
LYCcamIYVAIGQK
LYCcamIYVAIGQKR
NVQAEEMoxVEFSSGLK
NVQAEEMVEFSSGLK
QAVAYRQMoxSLLLR
QGQYSPMAIEEQVAVIYAGVR
QGQYSPMoxAIEEQVAVIYAGVR
QVAGTMKLELAQYR
QVAGTMoxKLELAQYR
RPPGREAYPGDVFYLHSR
RTGAIVDVPVGEELLGR
TGAIVDVPVGEELLGR
TSIAIDTIINQK
TSIAIDTIINQKR
VGLKAPGIIPR
VGSAAQTRAMoxK
VVDALGNAIDGKGPIGSK	
65	A28701 H+-transporting two-sector ATPase (EC 3.6.3.14) beta chain, mitochondrial - rat (fragment)	P06576
	51,2	4,9	165	53	16	AHGGYSVFAGVGER
AIAELGIYPAVDPLDSTSR
DQEGQDVLLFIDNIFR
FLSQPFQVAEVFTGHMoxGK
FTQAGSEVSALLGR
IGLFGGAGVGKTVLIMoxELINNVAK
IMoxDPNIVGSEHYDVAR
IMoxNVIGEPIDERGPIK
IPSAVGYQPTLATDMoxGTMoxQER
LVLEVAQHLGESTVR
SLQDIIAILGMoxDELSEEDKLTVSR
TREGNDLYHEMoxIESGVINLK
TVLIMoxELINNVAK
VALVYGQMNEPPGAR
VALVYGQMoxNEPPGAR
VLDSGAPIKIPVGPETLGR	
66	ATP synthase, H+ transporting, mitochondrial F1 complex, beta subunit precursor; mitochondrial ATP synthetase, beta subunit [Homo sapiens]	P10719
	56,6	5,2	231	54	21	AHGGYSVFAGVGER
AIAELGIYPAVDPLDSTSR
DQEGQDVLLFIDNIFR
EGNDLYHEMoxIESGVINLK
FLSQPFQVAEVFTGHMoxGK
FTQAGSEVSALLGR
IMoxNVIGEPIDER
IMoxNVIGEPIDERGPIK
IPSAVGYQPTLATDMoxGTMoxQER
IPVGPETLGRIMNVIGEPIDER
LVLEVAQHLGESTVR
QFAPIHAEAPEFMEMSVEQEILVTGIK
SLQDIIAILGMoxDELSEEDKLTVSR
TIAMoxDGTEGLVR
TREGNDLYHEMoxIESGVINLK
TVLIMoxELINNVAK
VALTGLTVAEYFR
VALVYGQMNEPPGAR
VALVYGQMoxNEPPGAR
VLDSGAPIKIPVGPETLGR
VVDLLAPYAK	
67	Atp5b protein [Mus musculus]	P56480
	56,7	5,2	316	82	39	AHGGYSVFAGVGER
AIAELGIYPAVDPLDSTSR
DQEGQDVLLFIDNIFR
EGNDLYHEMoxIESGVINLK
FLSQPFQVAEVFTGHMGK
FLSQPFQVAEVFTGHMoxGK
FTQAGSEVSALLGR
GFQQILAGEYDHLPEQAFYMoxVGPIEEAVAK
GGKIGLFGGAGVGK
GSITSVQAIYVPADDLTDPAPATTFAHLDATTVLSR
HASAMoxLSLVGRVASASASGALR
IGLFGGAGVGKTVLIMoxELINNVAK
IMDPNIVGNEHYDVAR
IMNVIGEPIDERGPIK
IMoxDPNIVGNEHYDVAR
IMoxNVIGEPIDER
IMoxNVIGEPIDERGPIK
IPSAVGYQPTLATDMGTMQER
IPSAVGYQPTLATDMoxGTMoxQER
IVAVIGAVVDVQFDEGLPPILNALEVQGR
KGSITSVQAIYVPADDLTDPAPATTFAHLDATTVLSR
LVLEVAQHLGESTVR
oxIPSAVGYQPTLATDMGTMQER
QFAPIHAEAPEFIEMoxSVEQEILVTGIK
SLQDIIAILGMDELSEEDKLTVSR
SLQDIIAILGMoxDELSEEDKLTVSR
TIAMDGTEGLVR
TIAMoxDGTEGLVR
TIAMoxDGTEGLVRGQK
TREGNDLYHEMIESGVINLK
TREGNDLYHEMoxIESGVINLK
TVLIMELINNVAKAHGGYSVFAGVGER
TVLIMoxELINNVAK
VALTGLTVAEYFR
VALTGLTVAEYFRDQEGQDVLLFIDNIFR
VALVYGQMNEPPGAR
VALVYGQMoxNEPPGAR
VLDSGAPIKIPVGPETLGR
VVDLLAPYAK	
68	ATP synthase, H+ transporting, mitochondrial F0 complex, subunit d [Mus musculus]	Q9DCX2
	18,7	5,3	148	86	16	AIGNALKSWNETFHAR
ANVAKPGLVDDFEK
ANVAKPGLVDDFEKK
IQEYEKQLEK
KYPYWPHQPIENL
LALKTIDWVSFVEVMoxPQNQK
LASLSEKPPAIDWAYYR
NIIPFDQMoxTIDDLNEIFPETK
NIIPFDQMoxTIDDLNEIFPETKLDK
NIIPFDQMTIDDLNEIFPETK
SCcamAEFVSGSQLR
SWNETFHAR
TIDWVSFVEVMoxPQNQK
TIDWVSFVEVMPQNQK
YNALKIPVPEDK
YPYWPHQPIENL	
69	ATP synthase, H+ transporting, mitochondrial F1 complex, O subunit [Mus musculus]	Q9DB20
	23,4	10	168	70	18	EKFSPLTANLMNLLAENGR
EKFSPLTANLMoxNLLAENGR
FSPLTANLMNLLAENGR
FSPLTANLMoxNLLAENGR
GEVPCcamTVTTASPLDDAVLSELKTVLK
IGEKYVDMoxSAK
LDQVEKELLR
LEIKTDPSIMoxGGMoxIVR
LGNTQGIISAFSTIMoxSVHR
LGNTQGIISAFSTIMSVHR
LVRPPVQVYGIEGR
SFLSPNQILK
SFLSPNQILKLEIK
TDPSIMoxGGMoxIVR
VSLAVLNPYIK
VSLAVLNPYIKR
YATALYSAASK
YATALYSAASKEK	
70	Adult male pituitary gland cDNA, RIKEN full-length enriched library, clone:5330423B21 product:hypothetical TPR repeat/TPR-like containing protein, full insert sequence Similar to FLJ20699 protein [Mus musculus]	Q3UZU5
	52,2	5,8	262	58	22	ACcamDLWEQILR
DALKPNSPLTER
DGLEFMQQSEGHWK
DHILLFNDAHFLMASLGAR
DHILLFNDAHFLMoxASLGAR
DVFNQLLIHAAMoxTCcamTSSVHK
DVGLPLCcamQALLEAENGNPDR
DVGLPLCcamQALLEAENGNPDRVLELLLPIR
ELLTTLQEASKSPGENCcamQHQLAK
EQLHVSAVEMoxFAK
GIYSFGLMoxETNFYDQAQK
IVQIGGSNAQR
LFDATLTQYVK
LQMoxEGVPLGQR
NAGLPLSTTSNEACcamKLFDATLTQYVK
SLLMoxERDALKPNSPLTER
SPGENCcamQHQLAK
TMoxVELSQTQTLTPR
TMVELSQTQTLTPR
VYPFWTPDIPLNSYVK
WQTVLPVTQK
WTNDKSLGGIEGCcamLSK	
71	cDNA sequence BC021608; hypothetical protein MGC37912 [Mus musculus]	Q8VBW8
	20,1	5	167	56	14	AAEAGDLHTALEK
AAEAGDLHTALEKFGQAISLLPDR
FGQAISLLPDR
FGQAISLLPDRASAYNNR
FQGRDDDAR
LQGDVAGALEDLER
LQGDVAGALEDLERAVTLSGGR
MoxLADMoxMoxGQLR
MoxLADMoxMoxGQLRAPSNGR
oxMLADMMGQLRAPSNGR
QLVLLNPYAALCcamNR
QSFVQSGLLAR
RLQGDVAGALEDLER
RQLVLLNPYAALCcamNR	
72	Branched chain ketoacid dehydrogenase E1, alpha polypeptide; BCKAD E1[a] [Mus musculus]	P50136
	50,4	8,4	237	65	32	AVAENQPFLIEAMoxTYR
AVAENQPFLIEAMTYR
DYPLELFMoxSQCcamYGNVNDPGKGR
GDGIAARGPGYGIMoxSIR
GPGYGIMoxSIRVDGNDVFAVYNATK
HFVTISSPLATQIPQAVGAAYAAK
HLQTYGEHYPLDHFDK
IGHHSTSDDSSAYR
ISFYMoxTNYGEEGTHVGSAAALER
ISFYMTNYGEEGTHVGSAAALER
KLKPNPSLLFSDVYQEMoxPAQLR
LKPNPSLLFSDVYQEMoxPAQLR
LKPNPSLLFSDVYQEMoxPAQLRR
LRQYLLNQGWWDEEQEK
NNGYAISTPTSEQYR
NNGYAISTPTSEQYRGDGIAAR
oxSMTLLNTMDR
QGQIINPSEDPHLPQEEVLK
QGQIINPSEDPHLPQEEVLKFYR
QMoxPVHYGCcamK
QMoxPVHYGCcamKER
QMPVHYGCcamK
QMPVHYGCcamKER
RAVAENQPFLIEAMoxTYR
SMoxTLLNTMoxDRILYESQR
SVDEVNYWDKQDHPISR
TDLVFGQYR
VDGNDVFAVYNATK
VMEAFEQAER
VMEAFEQAERK
VMoxEAFEQAER
VMoxEAFEQAERK	
73	Branched chain ketoacid dehydrogenase E1, beta polypeptide; branched chain keto acid dehydrogenase E1, beta polypeptide [Mus musculus]	P35738
	35,5	5,2	114	58	11	APWGCcamVGHGALYHSQSPEAFFAHCcamPGIK
DPTAVIFGEDVAFGGVFR
EVASMoxAQEKLGVSCcamEVIDLR
GLLLSCcamIEDKNPCcamIFFEPK
LGVSCcamEVIDLR
LLISHEAPLTGGFASEISSTVQEECcamFLNLEAPISR
MoxNLFQSITSALDNSLAK
SGDLFNCcamGSLTIR
TIVPWDVDTVCcamK
VCcamGYDTPFPHIFEPFYIPDKWK
YRSGDLFNCcamGSLTIR	
74	D-beta-hydroxybutyrate dehydrogenase, mitochondrial [Precursor] Unnamed protein product [Mus musculus]	Q80XN0
	38,2	9,2	221	61	26	AILITGCcamDSGFGFSLAK
FGIEAFSDCcamLR
GFLVFAGCcamLMK
GRVVNISSMLGR
GRVVNISSMoxLGR
MoxANPARSPYCcamITK
MoxETYCcamNSGSTDTSSVINAVTHALTAATPYTR
MoxWDDLPEVVR
MoxWDDLPEVVRK
MQIMTHFPGAISDKIYIH
MWDDLPEVVR
MWDDLPEVVRK
SFLPLLRR
SPYCcamITKFGIEAFSDCcamLR
TIQLNVCcamNSEEVEK
TIQLNVCcamNSEEVEKAVETIR
TTKSFLPLLR
VSVVEPGNFIAATSLYSPER
VSVVEPGNFIAATSLYSPERIQAIAK
VVNISSMLGR
VVNISSMoxLGR
YEMHPLGVK
YEMoxHPLGVK
YFDEKIAK
YHPMDYYWWLR
YHPMoxDYYWWLR	
75	Betaine-homocysteine methyltransferase 	O09171
	45	8	128	42	9	AGAAIVGVNCcamHFDPSTSLQTIK
AGPWTPEAAVEHPEAVR
AGSNVMoxQTFTFYASEDKLENR
AIAEELAPERGFLPPASEK
GAAELMoxQQKEATTEQQLR
KNVDFLIAEYFEHVEEAVWAVEALK
LNAGEVVIGDGGFVFALEKR
QGFIDLPEFPFGLEPR
YIGGCcamCcamGFEPYHIR	
76	Betaine-homocysteine methyltransferase 	O35490
	45	8	223	82	24	AGASIVGVNCcamHFDPSVSLQTVK
AGPWTPEAAVEHPEAVR
AGSNVMoxQTFTFYASEDKLENR
AIAEELAPER
AIAEELAPERGFLPPASEK
ASGKPVAATMCcamIGPEGDLHGVPPGECcamAVR
EATTEQQLRELFEK
GAAELMoxQQKEATTEQQLR
HGSWGSGLDMHTKPWIR
HGSWGSGLDMoxHTKPWIR
IASGRPYNPSMoxSRPDAWGVTK
IASGRPYNPSMSRPDAWGVTK
ISGQKVNEAACcamDIAR
KEYWQNLR
KNVDFLIAEYFEHVEEAVWAVEALK
LKAYLMoxSQPLAYHTPDCcamGK
LKAYLMSQPLAYHTPDCcamGK
LMoxKEGLEAAR
LNAGEVVIGDGGFVFALEKR
QGFIDLPEFPFGLEPR
QVADEGDALVAGGVSQTPSYLSCcamK
QVADEGDALVAGGVSQTPSYLSCcamKSEVEVK
VATRWDIQK
YIGGCcamCcamGFEPYHIR	
77	Biliverdin reductase B (flavin reductase (NADPH)) 	Q923D2
	22,2	6,3	167	88	17	CcamLTTNEYDGHTTYPSHQYD
DSSRLPSEGPQPAHVVVGDVR
HDLGHFMLR
HDLGHFMoxLR
KIAIFGATGR
LPSEGPQPAHVVVGDVR
LQDVTDDHIR
LQDVTDDHIRMoxHK
QAADVDKTVAGQEAVIVLLGTGNDLSPTTVMoxSEGTR
QAADVDKTVAGQEAVIVLLGTGNDLSPTTVMSEGTR
TGLTTLAQAVQAGYEVTVLVR
VISKHDLGHFMLR
VISKHDLGHFMoxLR
VVACcamTSAFLLWDPTKVPPR
YVAVMoxPPHIGDQPLTGAYTVTLDGR
YVAVMoxPPHIGDQPLTGAYTVTLDGRGPSR
YVAVMPPHIGDQPLTGAYTVTLDGR	
78	Biphenyl hydrolase-like (serine hydrolase, breast epithelial mucin-associated antigen) 	Q8R164
	32,9	9,1	143	64	18	ARKPLEALYGYDYLAK
FADEFNR
FADEFNRLVEDFLQ
FHADFLLQHVK
FTLVAWDPR
GYGYSRPPDRDFPR
HLLPLVQCcamPTLIVHGEKDPLVPR
IYQGIRDVSK
KMVIWGANAYVTEEDSR
KPLEALYGYDYLAK
MVIWGANAYVTEEDSR
QLPEGNICcamR
RFTLVAWDPR
TCcamEDWVDGISQFK
TDFAPQLQSLNK
VAVNGVHLHYQR
VGEGEHAILLLPGMoxLGSGK
YPSYIRK	
79	Bisphosphate 3'-nucleotidase 1 	Q9Z0S1
	33,2	5,4	288	79	24	ADRLVQMoxSICcamSSLAR
AIAGIINQPYYNYQAGPDAALGR
ASAYVFASPGCcamK
CcamVIAEGDLGIVQK
EAPAGKHIITTTR
EEDLVVWVDPLDGTK
EVKHMoxNSAGVLAALR
EYTEGLLDNVTVLIGIAYEGK
HMNSAGVLAALR
HMoxNSAGVLAALR
KWDTCcamAPEVILHAVGGK
LTDIHGNALQYNK
LTDIHGNALQYNKEVK
LVASAYSIAQK
LVASAYSIAQKAGTIVR
LVQMoxSICcamSSLAR
LVQMSICcamSSLAR
NYEYYASHVPESVK
NYEYYASHVPESVKNALIP
SHSNQLVTDCcamISAMNPDTVLR
SHSNQLVTDCcamISAMoxNPDTVLR
TIWGVLGLGAFGFQLK
VGGAGNKIIQLIEGK
WDTCcamAPEVILHAVGGK	
80	Capping protein (actin filament) muscle Z-line, alpha 2 	P47754
	32,9	5,5	181	63	15	AYVKEHYPNGVCcamTVYGK
EATDPRPYEAENAIESWR
EHYPNGVCcamTVYGK
FIIHAPPGEFNEVFNDVR
FIIHAPPGEFNEVFNDVRLLLNNDNLLR
FTVTPSTTQVVGILK
ICcamFKFDHLR
IDWNKILSYK
IQVHYYEDGNVQLVSHK
IQVHYYEDGNVQLVSHKDIQDSLTVSNEVQTAK
IVEAAENEYQTAISENYQTMoxSDTTFK
KVDGQQTIIACcamIESHQFQAK
LLLNNDNLLR
SEWKFTVTPSTTQVVGILK
VDGQQTIIACcamIESHQFQAK	
81	F-actin capping protein beta subunit	P47757
	30,6	5,5	180	64	21	GCcamWDSIHVVEVQEK
IKGCcamWDSIHVVEVQEK
KLEVEANNAFDQYR
LEVEANNAFDQYR
LTSTVMoxLWLQTNK
LVEDMENKIR
LVEDMoxENKIR
NLSDLIDLVPSLCcamEDLLSSVDQPLK
NLSDLIDLVPSLCcamEDLLSSVDQPLKIAR
QMEKDETVSDCcamSPHIANIGR
QMoxEKDETVSDCcamSPHIANIGR
RLPPQQIEK
SGSGTMNLGGSLTR
SGSGTMoxNLGGSLTR
SPWSNKYDPPLEDGAMoxPSAR
SPWSNKYDPPLEDGAMPSAR
STLNEIYFGK
STLNEIYFGKTK
TAHYKLTSTVMLWLQTNK
TAHYKLTSTVMoxLWLQTNK
VVGKDYLLCcamDYNR	
82	Carbonic anhydrase 2 	P00920
	29	6,6	208	70	15	AVQQPDGLAVLGIFLK
AVQQPDGLAVLGIFLKIGPASQGLQK
HNGPENWHKDFPIANGDR
IGPASQGLQKVLEALHSIK
KYAAELHLVHWNTK
LIQFHFHWGSSDGQGSEHTVNK
LIQFHFHWGSSDGQGSEHTVNKK
QSPVDIDTATAHHDPALQPLLISYDK
QSPVDIDTATAHHDPALQPLLISYDKAASK
SIVNNGHSFNVEFDDSQDNAVLK
SIVNNGHSFNVEFDDSQDNAVLKGGPLSDSYR
TLNFNEEGDAEEAMoxVDNWRPAQPLK
TLNFNEEGDAEEAMVDNWRPAQPLK
YAAELHLVHWNTK
YAAELHLVHWNTKYGDFGK	
83	Carbonic anhydrase 3 	P16015
	29,3	7,1	205	90	20	DIKHDPSLQPWSASYDPGSAK
DYWTYHGSFTTPPCcamEECcamIVWLLLK
EAPFTHFDPSCcamLFPACcamR
EKGEFQILLDALDK
EPMTVSSDQMAKLR
EWGYASHNGPDHWHELYPIAK
GDNQSPIELHTK
GEFQILLDALDKIK
GGPLSGPYRLR
GKEAPFTHFDPSCcamLFPACcamR
HDPSLQPWSASYDPGSAK
oxEPMTVSSDQMAK
QFHLHWGSSDDHGSEHTVDGVK
QPDGIAVVGIFLK
SLFSSAENEPPVPLVGNWRPPQPVK
VVFDDTYDR
VVFDDTYDRSMLR
VVFDDTYDRSMoxLR
YAAELHLVHWNPK
YNTFGEALKQPDGIAVVGIFLK	
84	Carbohydrate kinase-like 	Q9D5J6
	51,3	5,7	251	58	23	AALLEAAPSLPSGFVVLASCcamAR
AARAETESAVAGPQGR
AETESAVAGPQGR
AETESAVAGPQGREQDVTR
AFPFPVCcamFGQDVDAAFGAALVMoxLQR
AGFPIHLLPDIAEPGSMAGR
AGFPIHLLPDIAEPGSMoxAGR
AGQGCcamEWMoxEGGPAFVFEPR
ALCcamRGIVQNLHSMLPFQQLK
AVSHLVTWQDGR
DTHLTITPTVLGER
GIGVSGQMoxHGILFWK
GIVQNLHSMoxLPFQQLK
GIVQNLHSMoxLPFQQLKEWGVAR
GTQVGIALGDLQASVYSCcamMoxGQR
HLPDQLASVTR
IIQALNECcamLDALPR
ISSSDLSLGHVTR
MoxIQAAAQQKDTHLTITPTVLGER
NEVLKQEVQR
TSHTWFEIPK
VRGIGVSGQMoxHGILFWK
VVGSGSALSRNEVLK	
85	Catalase; catalase 1 [Mus musculus]	P24270
	59,7	8,1	227	69	34	ASQRPDVLTTGGGNPIGDK
ASQRPDVLTTGGGNPIGDKLNIMoxTAGSR
ASQRPDVLTTGGGNPIGDKLNIMTAGSR
AVKNFTDVHPDYGAR
DAILFPSFIHSQK
DAILFPSFIHSQKR
DGPMoxCcamMoxHDNQGGAPNYYPNSFSAPEQQR
EAETFPFNPFDLTK
FHYKTDQGIK
FNSANEDNVTQVR
FSTVAGESGSADTVRDPR
FYTEDGNWDLVGNNTPIFFIR
GAGAFGYFEVTHDITR
GIPDGHRHMNGYGSHTFK
GIPDGHRHMoxNGYGSHTFK
GPLLVQDVVFTDEMAHFDR
GPLLVQDVVFTDEMAHFDRER
GPLLVQDVVFTDEMoxAHFDR
GPLLVQDVVFTDEMoxAHFDRER
HMoxNGYGSHTFK
HRLGPNYLQIPVNCcamPYR
IQALLDKYNAEKPK
LAQEDPDYGLR
LFAYPDTHR
LFAYPDTHRHR
LGPNYLQIPVNCcamPYR
LVNADGEAVYCcamKFHYK
NAIHTYTQAGSHMAAK
NFTDVHPDYGAR
NPVNYFAEVEQMoxAFDPSNMoxPPGIEPSPDKK
RLCcamENIAGHLK
SALEHSVQCcamAVDVK
SALEHSVQCcamAVDVKR
VWPHKDYPLIPVGK	
86	Carbonyl reductase 1 [Mus musculus]	P48758
	30,6	9,5	186	76	22	DEERGQTAVQK
DFLLKEYGGLDVLVNNAGIAFK
DVCcamKELLPLIKPQGR
ELLPLIKPQGR
EYGGLDVLVNNAGIAFK
FHQLDIDNPQSIR
FRSETITEEELVGLMNK
FRSETITEEELVGLMoxNK
FSGDVVLAARDEER
GDKILLNACcamCcamPGWVR
ILLNACcamCcamPGWVR
ILLNACcamCcamPGWVRTDMAGPK
KFSGDVVLAAR
KGVHAEEGWPNSAYGVTK
MoxSSSRPVALVTGANK
NCcamRLELQQK
SETITEEELVGLMoxNK
SPEEGAETPVYLALLPPDAEGPHGQFVQDK
VNDDTPFHIQAEVTMK
VNDDTPFHIQAEVTMoxK
VVNVSSMoxVSLR
VVNVSSMVSLR	
87	Cystathionine beta-synthase isoform 1 [Mus musculus]	Q91WT9
	60,2	6,1	197	52	21	AARELQEGQR
AGNLKPGDTIIEPTSGNTGIGLALAAAVK
AMoxADSPHYHTVLTK
CcamEFFNAGGSVK
CcamEFFNAGGSVKDR
DQAWSGVVGGPTDCcamSNGMoxSSK
FLSDKWMoxLQK
LDMoxLVASAGTGGTITGIARK
LKNEIPNSHILDQYR
LRVQELSLSAPLTVLPTVTCcamEDTIAILR
MoxLIAQEGLLCcamGGSSGSAMoxAVAVK
QQMoxVFGVVTAIDLLNFVAAR
QQMVFGVVTAIDLLNFVAAR
SNDEDSFAFAR
VQELSLSAPLTVLPTVTCcamEDTIAILR
VWIRPDTPSR	
88	Copper chaperone for superoxide dismutase [Mus musculus]	Q9WU84
	28,9	5,6	143	85	13	DCcamNSCcamGDHFNPDGASHGGPQDTDR
DCcamNSCcamGDHFNPDGASHGGPQDTDRHR
FLQLSSELCcamLIEGTIDGLEPGLHGLHVHQYGDLTR
GDLGNVRAEAGGR
GRPIAGQGRK
GVAGVQNVDVQLENQMoxVLVQTTLPSQEVQALLESTGR
QAVLKGMoxGSSQLQNLGAAVAILEGCcamGSIQGVVR
QICcamSCcamDGLTIWEER
QLKVWDVIGR
SAGLFQNPKQICcamSCcamDGLTIWEER
SGDGGTVCcamALEFAVQMoxSCcamQSCcamVDAVHK
SLVIDEGEDDLGR
SLVIDEGEDDLGRGGHPLSK	
89	Chaperonin subunit 2 (beta) [Mus musculus]	P80314
	57,5	5,9	232	73	31	AAHSEGHITAGLDMK
AGADEERAETAR
DAALMoxVTNDGATILK
DAALMVTNDGATILK
EALLSSAVDHGSDEAR
EGTIGDMAVLGITESFQVKR
EGTIGDMoxAVLGITESFQVKR
FWQDLMoxNIAGTTLSSK
GATQQILDEAER
HGINCcamFINR
ILKHGINCcamFINR
KIHPQTIISGWR
LALVTGGEIASTFDHPELVK
LGGSLADSYLDEGFLLDKK
LIEEVMIGEDKLIHFSGVALGEACcamTIVLR
LIEEVMoxIGEDKLIHFSGVALGEACcamTIVLR
LIHFSGVALGEACcamTIVLR
LKGSGNLEAIHVIK
LLTHHKDHFTK
LSSFIGAIAIGDLVKSTLGPK
MLPTIIADNAGYDSADLVAQLR
MoxLPTIIADNAGYDSADLVAQLR
oxTVYGGGCcamSEMLMAHAVTQLANR
QLIYNYPEQLFGAAGVMAIEHADFAGVER
QVLLSAAEAAEVILR
QVLLSAAEAAEVILRVDNIIK
SLHDALCcamVLAQTVKDPR
TPGKEAVAMESFAK
TPGKEAVAMoxESFAK
TVYGGGCcamSEMoxLMoxAHAVTQLANR
VQDDEVGDGTTSVTVLAAELLR	
90	Chaperonin subunit 5 (epsilon) [Mus musculus]	P80316
	59,6	5,7	242	57	28	AFADALEVIPMoxALSENSGMoxNPIQTMoxTEVR
DVDFELIKVEGK
ESNPALGIDCcamLHK
FSELTSEKLGFAGVVQEISFGTTK
GIHPIRIADGYEQAAR
GSNDMoxQYQHVIETLIGK
GSNDMoxQYQHVIETLIGKK
GVIVDKDFSHPQMPK
HKLDVMoxSVEDYK
IADGYEQAAR
IAILTCcamPFEPPKPK
IAIQHLDKISDK
IDDIRKPGESEE
ISDKVLVDINNPEPLIQTAK
KQQISLATQMoxVR
LGFAGVVQEISFGTTK
LGFAGVVQEISFGTTKDK
LMoxGLEALKSHIMoxAAK
LMoxVELSKSQDDEIGDGTTGVVVLAGALLEEAEQLLDR
MoxILKIDDIR
oxQMAEIAVNAVLTVADMERR
QMoxAEIAVNAVLTVADMoxERR
QQISLATQMoxVR
QQISLATQMVR
QVKESNPALGIDCcamLHK
SLHDALCcamVIR
SQDDEIGDGTTGVVVLAGALLEEAEQLLDR
VLVDINNPEPLIQTAK
WVGGPEIELIAIATGGR	
91	Similar to CCT (chaperonin containing TCP-1) zeta subunit [Rattus norvegicus]	P80317
	58	6,5	133	49	20	AEVARAQAALAVNISAAR
ALQFLEQVKVSK
AQAALAVNISAAR
AQLGVQAFADALLIIPK
DGNVLLHEMoxQIQHPTASLIAK
DGNVLLHEMQIQHPTASLIAK
EMDRETLIDVAR
GIDPFSLDALAK
GLQDVLRTNLGPK
GLVLDHGARHPDMoxK
GRAQLGVQAFADALLIIPK
IITEGFEAAKEK
MoxLVSGAGDIKLTK
QADLYISEGLHPR
QLLHSCcamTVIATNILLVDEIMR
TKVHAELADVLTEAVVDSILAIR
VATAQDDITGDGTTSNVLIIGELLK
VCcamGDSDKGFVVINQK
VHAELADVLTEAVVDSILAIR
VLAQNSGFDLQETLVK	
92	Cofilin 1, non-muscle [Mus musculus]	P18760
	18,6	8,2	106	67	10	AVLFCcamLSEDKK
EILVGDVGQTVDDPYTTFVK
HELQANCcamYEEVKDR
KEDLVFIFWAPENAPLK
LGGSAVISLEGKPL
MLPDKDCcamR
MoxLPDKDCcamR
NIILEEGKEILVGDVGQTVDDPYTTFVK
VFNDMoxKVR
YALYDATYETK	
93	Choline dehydrogenase [Mus musculus]	Q8BJ64
	66,4	8,8	178	54	28	ADSAYHPSCcamTCcamKMGR
AQRHELGANMoxYR
AQRHELGANMYR
AVGVEYIKDGQR
DHPVIHPNYLSTETDVEDFRQCcamVR
EGAEGWDYAHCcamLPYFR
EGAEGWDYAHCcamLPYFRK
EIFAQEALAPFR
EIFAQEALAPFRGK
ELQPGSHVQSDKEIDAFVR
EVILSGGAINSPQLLMoxLSGVGNADDLR
GHAEDYNRWHR
GHPALEDKNVPVYKPQTLDTQR
GKTNHPLHQAFLQAAR
HELGANMoxYR
HELGANMYR
KPTQQEAYQVHVGTMoxR
KPTQQEAYQVHVGTMR
LSREIFAQEALAPFR
MWQVLRGWR
oxVVDASIMPSVVSGNLNAPTVMIAEK
SRPGVPHPDIQFHFLPSQVIDHGR
SSDPTAVVDAQTKVIGVENLR
TNHPLHQAFLQAAR
VVDASIMoxPSVVSGNLNAPTVMoxIAEK
VWGGSSSLNAMoxVYIR
VWGGSSSLNAMVYIR
WSTACcamAYLHPVLSRPNLR	
94	Chloride intracellular channel 5 [Mus musculus]	Q8BXK9
	28,6	5,9	103	47	7	AGIDGESIGNCcamPFSQR
DEFTNTCcamAADSEIELAYADVAR
FLDGDELTLADCcamNLLPK
GVVFNVTTVDLKR
HRESNTAGIDIFSK
KPADLHNLAPGTHPPFLTFNGDVK
NYDIPAEMoxTGLWR	
95	KCY_MOUSE UMP-CMP kinase (Cytidylate kinase) (Deoxycytidylate kinase) (Cytidine monophosphate kinase)	Q9DBP5
	22,2	5,5	199	68	16	EMoxDQTMoxAANAQK
IQTYLESTKPIIDLYEEMoxGK
IQTYLESTKPIIDLYEEMoxGKVK
IVEKYGYTHLSAGELLR
IVPVEITISLLK
IVPVEITISLLKR
KNPDSQYGELIEK
MKPLVVFVLGGPGAGK
MoxKPLVVFVLGGPGAGK
MoxKPLVVFVLGGPGAGKGTQCcamAR
NKFLIDGFPR
NQDNLQGWNK
RIQTYLESTKPIIDLYEEMoxGK
SVDEVFGEVVK
YGYTHLSAGELLR
YGYTHLSAGELLRDER	
96	CNDP dipeptidase 2 (metallopeptidase M20 family); cytosolic nonspecific dipeptidase [Mus musculus]	Q9D1A2
	52,8	5,4	294	71	31	ALKTVFGVEPDLTR
AVFQYIDENQDR
AVFQYIDENQDRYVK
DVGAETLLHSCcamKK
EGGSIPVTLTFQEATGK
FAELQSPNKFK
FCcamLEGMEESGSEGLDELIFAQK
FCcamLEGMoxEESGSEGLDELIFAQK
GSTDDKGPVAGWMoxNALEAYQK
ILIPGINDAVAPVTDEEHALYDHIDFDMEEFAK
LAEWVAIQSVSAWPEKR
LGGSVELVDIGK
LNRLNYIEGTK
LVPDMoxIPEVVSEQVSSYLSK
LVPDMoxIPEVVSEQVSSYLSKK
MLAAYLYEVSQLKN
MoxLAAYLYEVSQLKN
MoxMoxEVAAADVQR
NKPCcamITYGLR
NVMoxLLPVGSADDGAHSQNEK
NVMoxLLPVGSADDGAHSQNEKLNR
oxRMMEVAAADVQR
QKLPDGSEIPLPPILLGK
RMoxMoxEVAAADVQR
TGQEIPVNLR
TVFGVEPDLTR
TVFGVEPDLTREGGSIPVTLTFQEATGK
VYMoxGHGGKPWVSDFNHPHYQAGR
VYMoxGHGGKPWVSDFNHPHYQAGRR
WRYPSLSLHGIEGAFSGSGAK
YPSLSLHGIEGAFSGSGAK	
97	Catechol-O-methyltransferase [Mus musculus]	O88587
	29,5	5,5	160	41	10	AVYQGPGSSPVK
AVYQGPGSSPVKS
DRYLPDTLLLEECcamGLLR
EYRPSLVLELGAYCcamGYSAVR
GSSSFECcamTHYSSYLEYMK
GSSSFECcamTHYSSYLEYMoxK
GSSSFECcamTHYSSYLEYMoxKVVDGLEK
GTVLLADNVIVPGTPDFLAYVR
KGTVLLADNVIVPGTPDFLAYVR
VSILIGAPQDLIPQLKK
YLPDTLLLEECcamGLLR
YLPDTLLLEECcamGLLRK	
98	RIKEN cDNA 2310005O14 [Mus musculus]	Q8K1Z0
	35,1	5,5	117	41	13	INDAMoxNMoxGHTAK
LNQVLEEEQK
LNQVLEEEQKLVQLGQAEK
LRMoxLIPYIEHWPR
LVQLGQAEKR
MLIPYIEHWPR
MoxLIPYIEHWPR
NLTGLNQRR
QQPPHSSSQQHSETQGPEFSRPPPR
STGEALVQGLMGAAVTLK
STGEALVQGLMoxGAAVTLKNLTGLNQR
TDQFLRDAVETR
YTDQSGEEEEDYESEEQLQHR	
99	Coronin, actin binding protein 1A; coronin-like actin binding protein; coronin 1a; coronin 1 [Mus musculus]	O89053
	51	6,1	166	39	17	ADQCcamYEDVR
CcamEPIAMoxTVPR
CcamEPIAMoxTVPRK
DAGPLLISLKDGYVPPK
DGALICcamTSCcamR
DRPHEGTRPVHAVFVSEGK
DRPHEGTRPVHAVFVSEGKILTTGFSR
ESQRGMoxGYMoxPK
FMALICcamEASGGGAFLVLPLGK
FMoxALICcamEASGGGAFLVLPLGK
FRHVFGQPAK
GDSSIRYFEITSEAPFLHYLSMoxFSSK
GLEVNKCcamEIAR
HVFGQPAKADQCcamYEDVR
LDRLEETVQAK
VSQTTWDSGFCcamAVNPK
YFEITSEAPFLHYLSMoxFSSK	
100	Cytochrome c oxidase subunit 5A, mitochondrial [Precursor]; Cytochrome oxidase subunit Va; R Chain R, Bovine Heart Cytochrome C Oxidase At The Fully Reduced State	P00426
	12,4	5	102	84	9	EIYPYVIQELRPTLNELGISTPEELGLDKV
GMNTLVGYDLVPEPK
GMoxNTLVGYDLVPEPK
KGMoxNTLVGYDLVPEPK
LNDFASAVRILEVVK
RLNDFASAVR
SHGSHETDEEFDAR
WVTYFNKPDIDAWELR
WVTYFNKPDIDAWELRK	
101	Cox5b protein [Mus musculus]	P19536
	13,8	8,3	84	51	8	CcamPNCcamGTHYK
CcamPNCcamGTHYKLVPHQMAH
CcamPNCcamGTHYKLVPHQMoxAH
EDPNLVPSISNKR
GESQRCcamPNCcamGTHYK
GLDPYNMLPPK
GLDPYNMoxLPPK
IVGCcamICcamEEDNCcamTVIWFWLHKGESQR	
102	Coproporphyrinogen oxidase; clone 560 [Mus musculus]	P36552
	40,6	7	190	66	29	AGVSISVVHGNLSEEAANQMoxR
AGVSISVVHGNLSEEAANQMR
ALAQVDGVADFTVDRWER
CcamSTFMoxSSPVTELR
CcamSTFMSSPVTELR
DKLWQQLR
DSKLPFTAMGVSSVIHPK
EACcamDQHGPDIYPK
EGGGGITCcamVLQDGR
EGGGGITCcamVLQDGRVFEK
FGLFTPGSRIESILMoxSLPLTAR
GIGGIFFDDLDSPSKEEAFR
GRYVEFNLLYDR
GTKFGLFTPGSR
HCcamDDSYTPR
IESILMoxSLPLTAR
IESILMSLPLTAR
KEGGGGITCcamVLQDGR
KHCcamDDSYTPR
NPYAPTMHFNYR
NPYAPTMoxHFNYR
oxTKMELMIMETQAQVCcamR
RCcamSTFMoxSSPVTELR
TCcamAEAVVPSYVPIVK
TCcamAEAVVPSYVPIVKK
TLKEACcamDQHGPDIYPK
WCcamDDYFFIVHR
YVEFNLLYDR
YVEFNLLYDRGTK	
103	Carbamoyl-phosphate synthetase 1, mitochondrial; Carboamyl-phosphate synthetase 1 [Rattus norvegicus]	P07756
	164,6	6,3	350	43	55	AADTIGYPVMIR
AADTIGYPVMoxIR
AEDRSIFSAVLDELK
AERPDGLILGMGGQTALNCcamGVELFKR
AFAISGPFNVQFLVK
AFAMoxTNQILVER
AFAMTNQILVER
ATGYPLAFIAAK
CcamEMASTGEVACcamFGEGIHTAFLK
CcamLGLTEAQTR
DGSIDLVINLPNNNTK
EVEMoxDAVGKEGR
FLGVAEQLHNEGFK
FVHDNYVIR
FVHDNYVIRR
GILIGIQQSFRPR
GKGTTITSVLPKPALVASR
GNDVLVIECcamNLR
GQNQPVLNITNR
GTTITSVLPKPALVASR
GTTITSVLPKPALVASRVEVSK
HLPTLEQPIIPSDYVAIK
IALGIPLPEIK
IALGIPLPEIKNVVSGK
IAPSFAVESMoxEDALKAADTIGYPVMoxIR
ILDIYHQEACcamNGCcamIISVGGQIPNNLAVPLYK
IMGTSPLQIDRAEDR
IMoxGTSPLQIDR
IMoxGTSPLQIDRAEDR
KEPLFGISTGNIITGLAAGAK
LIRDGSIDLVINLPNNNTK
LRDADPILR
LTSIDKWFLYK
MCcamHPSVDGFTPR
MoxCcamHPSVDGFTPR
MoxCcamHPSVDGFTPRLPMoxNK
QADAVYFLPITPQFVTEVIK
QLFSDKLNEINEK
SGFGLANVTSKR
SIFSAVLDELK
SIFSAVLDELKVAQAPWK
TAVDSGIALLTNFQVTK
TAVDSGIALLTNFQVTKLFAEAVQK
TLGVDFIDVATK
TSACcamFEPSLDYMoxVTK
TSACcamFEPSLDYMVTKIPR
TVLMoxNPNIASVQTNEVGLK
TVVVNCcamNPETVSTDFDECcamDKLYFEELSLER
VISHAISEHVEDAGVHSGDATLMoxLPTQTISQGAIEK
VLGTSVESIMATEDR
VLGTSVESIMoxATEDR
VLILGSGGLSIGQAGEFDYSGSQAVK
VLILGSGGLSIGQAGEFDYSGSQAVKAMoxK
VVAVDCcamGIKNNVIR
WDLDRFHGTSSR	
104	Carnitine palmitoyltransferase 2; CPT II [Mus musculus]	P52825
	73,9	8,5	127	37	15	AGLLEPEVFHLNPAR
CcamLEDMFDALEGK
DFENGIGKELHAHLLAQDK
DGTAAVHFEHAWGDGVAVLR
DSTQTPAIAPQSQPAATDSSVSVQK
DSVVLNFNPFMoxAFNPDPK
GVTLPELYQDPAYQR
IPKPSRDELFTDTK
LIHGGNEETLR
LSPDAVAQLAFQMoxAFLR
QYGQTVATYESCcamSTAAFK
SEYNDQLTR
TLTIDAIQFQR
YILSDSSPVPEFPLAYLTSENR
YLSAQKPLLNDSQFR	
105	Cryz protein [Mus musculus]	P47199
	35,3	8,2	84	57	11	AAQAHEDIIHGSGK
AGESVLVHGASGGVGLATCcamQIAR
ETSIIGVSLSSSTKEEFQQFAGLLQAGIEK
GWVKPVIGSEYPLEK
KPALPYTPGSDVAGIIESVGDKVSAFK
LQSDVVVPVPQSHQVLIK
LVLQNGAHEVFNHK
MoxSVGDKDK
QGAALGIPYFTACcamR
VFEFGGPEVLK
VHACcamGVNPVETYIR	
106	Cathepsin B preproprotein [Mus musculus]	P10605
	37,3	5,5	94	35	12	CcamNKSCcamEAGYSPSYK
DQGSCcamGSCcamWAFGAVEAISDR
EQWSNCcamPTIGQIR
GENHCcamGIESEIVAGIPR
HEAGDMoxMoxGGHAIR
HFGYTSYSVSNSVK
ILRGENHCcamGIESEIVAGIPR
NGPVEGAFTVFSDFLTYK
oxHEAGDMMGGHAIR
oxSGVYKHEAGDMMGGHAIR
SCcamEAGYSPSYKEDK
SGVYKHEAGDMoxMoxGGHAIR	
107	Cytochrome b5 1205244A	P00173
	10,5	5,1	146	73	9	ELSKTYIIGELHPDDR
EQAGGDATENFEDVGHSTDAR
EQAGGDATENFEDVGHSTDARELSK
FLEEHPGGEEVLR
FLEEHPGGEEVLREQAGGDATENFEDVGHSTDAR
STWVILHHKVYDLTK
TYIIGELHPDDR
TYIIGELHPDDRSK
VYDLTKFLEEHPGGEEVLR
YYTLEEIQK
YYYLEEIK
STWVILHHK	
108	A Chain A, Dimethyl Propionate Ester Heme-Containing Cytochrome B5	O35768
	10,8	5	188	83	12	ELSKTYIIGELHPDDR
EQAGGDATENFEDVGHSTDAR
EQAGGDATENFEDVGHSTDARELSK
FLEEHPGGEEVLR
FLEEHPGGEEVLREQAGGDATENFEDVGHSTDAR
STWVILHHK
STWVILHHKVYDLTK
TYIIGELHPDDR
TYIIGELHPDDRSK
VYDLTKFLEEHPGGEEVLR
YYTLEEIQK
YYTLEEIQKHK	
109	Es1 protein [Mus musculus]	Q9D172
	28,1	9,1	91	63	11	GGAEVQIFAPDVPQMHVIDHTKGEPSER
GGAEVQIFAPDVPQMoxHVIDHTKGEPSER
GKITSLAQLNAANHDAAIFPGGFGAAK
GVEVTVGHEQEEGGKWPYAGTAEAIK
ITSLAQLNAANHDAAIFPGGFGAAK
KPIGLCcamCcamIAPVLAAK
NLSTFAVDGKDCcamK
VALVLSGCcamGVYDGTEIHEASAILVHLSR
VLKEFHGAK
VLVAPRLASALLPLSR
VNKEVER	
110	Interleukin 25; lymphocyte antigen 6 complex, locus E ligand [Mus musculus]	Q9CPT4
	18	5,9	89	37	7	AELSKLVIVAK
ESDVPLKSEEFEVTK
GAEIEYAMAYSK
GAEIEYAMoxAYSK
SYLYFTQFK
SYLYFTQFKAELR
TAVSHRPGAFKAELSK	
111	Drebrin-like [Mus musculus]	Q62418
	48,4	4,7	101	40	15	AMSTTSVTSSQPGKLR
EQRYQEQHR
ESTSFQDVGPQAPVGSVYQK
FVLINWTGEGVNDVR
FVLINWTGEGVNDVRK
GACcamANHVSTMANFLK
GACcamANHVSTMoxANFLK
NGPALQEAYVR
QEWESAGQQAPHPREIFK
SAGAPSRTGEPEQEAVSR
SPTDWALFTYEGNSNDIR
TGEPEQEAVSR
TRQEWESAGQQAPHPR
VAGTGEGGLEELVEELNSGK
VGKDNFWAK	
112	Dihydrolipoamide branched chain transacylase E2 [Mus musculus]	P53395
	53,3	8,9	190	53	28	AASLGLLQFPILNASVDENCcamQNITYK
AQIMNVSWSADHRVIDGATMSR
ASHNIGIAMDTELGLIVPNVK
ASHNIGIAMoxDTELGLIVPNVK
DRTEPVTGFQK
EGDTVSQFDSICcamEVQSDKASVTITSR
GIKLSFMoxPFFLK
GIKLSFMPFFLK
ILKEDILSFLEK
IPHFGYCcamDEIDLTQLVK
LREELKPVALAR
LSDIGEGIREVTIK
LSEVVGSGKDGR
LSFMoxPFFLK
LSFMPFFLK
SEITPPPPQPK
SEITPPPPQPKDR
SVFEIAMELNR
SVFEIAMELNRLQK
SVFEIAMoxELNR
SVFEIAMoxELNRLQK
SYLENPAFMLLDLK
SYLENPAFMoxLLDLK
TFPTPIAKPPVFTGK
TFPTPIAKPPVFTGKDR
TMoxSAALKIPHFGYCcamDEIDLTQLVK
TMSAALKIPHFGYCcamDEIDLTQLVK
YSQPRHSLR	
113	C1-tetrahydrofolate synthase [Mus musculus]	Q8R013
	101,2	6,6	195	25	22	ASQAPSSFQLLYDLK
CcamTHWAEGGQGALALAQAVQR
EHGAFDAVK
FSDIQIR
FSDIQIRR
GCcamLELIKEAGVQIAGR
GVPTGFVLPIR
GVPTGFVLPIRDIR
IFHELTQTDK
IFHELTQTDKALFNR
KFSDIQIR
LDIDPETITWQR
LGIEKTDPTTLTDDEINR
LVPSVNGIR
QNVFACcamVR
QPSQGPTFGIK
STTTIGLVQALGAHLR
THLSLSHNPEQK
VLDTNDRFLR
VLLSALDRLK
VVGDVAYDEAKER
YSGLQPHVVVLVATVR	
114	Dynactin 2 [Mus musculus]	Q99KJ8
	44,1	5,1	120	60	15	ADPKYADLPGIAR
ASVEDADTQNKVHQLYETIQR
ATAGAPPDSSLVTYELHSRPEQDKFSQAAK
ENLATVEGNFASIDAR
ESATEEKLTPVVLAK
GLDFSDRIGK
LLGPDAAINLADPDGALAKR
LLHEVQELTTEVEKIK
LQSVLGKVNEIAK
QLAALKQQLVASHLEK
RLLLQLEATK
RLTELEATVR
TGYESGDYEMoxLGEGLGVKETPQQK
VSALDLAVLDQVEAR
WSPVASTLPELVQR	
115	Diacetyl/L-xylulose reductase [Cavia porcellus]	Q91X52
	25,7	7,8	228	71	19	AAGAQVVAVSR
ALTNHTVYCcamSTK
ALTNHTVYCcamSTKGALDMoxLTK
ALVTGAGKGIGR
AMLDRIPLGK
AMoxLDRIPLGK
AVIQVSQIVAK
EACcamDTSFNVNLR
FAEVENVVDTILFLLSNR
GVPGAIVNVSSQASQR
IRVNAVNPTVVMoxTPMoxGR
MoxMoxALELGPHK
oxMMALELGPHK
oxMMALELGPHKIR
oxVNAVNPTVVMTPMGR
SGMoxTTGSTLPVDGGFLAT
STVLALKAAGAQVVAVSR
TREDLDDLVR
VNAVNPTVVMoxTPMoxGR	
116	Dimethylarginine dimethylaminohydrolase 1 Mus musculus	Q9CWS0
	31,4	5,5	271	72	23	ATHAVVRAPPESLCcamR
DENATLDGGDVLFTGR
DYAVSTVPVADSLHLK
EFFVGLSKR
GAEILADTFKDYAVSTVPVADSLHLK
GHVLLHRTPEEYPESAK
IMoxQQMoxSDHR
IMoxQQMoxSDHRYDK
LKDHLLIPVSNSEMoxEK
LQLNIVEMKDENATLDGGDVLFTGR
LQLNIVEMoxKDENATLDGGDVLFTGR
LTVPDDMoxAANCcamIYLNIPSK
oxIMQQMSDHRYDK
QHELYVGVLGSK
RSQGEEVDFAR
SFCcamSMAGPNLIAIGSSESAQK
SFCcamSMoxAGPNLIAIGSSESAQK
SQGEEVDFAR
SQGEEVDFARAER
SQYEQLAEQNRK
TPEEYPESAK
TPEEYPESAKVYEK
VDGLLTCcamCcamSVFINK
VDGLLTCcamCcamSVFINKK	
117	Ddx5 protein [Mus musculus]	Q61656
	74,6	8,9	241	43	27	APILIATDVASR
DLERGVEICcamIATPGR
ELAQQVQQVAAEYCcamR
FGNPGEKLVK
FVINYDYPNSSEDYIHR
GGFNTFRDR
GLDVEDVKFVINYDYPNSSEDYIHR
GPQIRDLER
GVEICcamIATPGR
HGKAPILIATDVASR
IVDQIRPDR
KIVDQIRPDR
LIDFLECcamGKTNLR
LLQLVEDRGSGR
LMEEIMSEKENK
MoxLDMoxGFEPQIR
NFYQEHPDLAR
NFYQEHPDLARR
oxMLDMGFEPQIR
QVSDLISVLREANQAINPK
STCcamIYGGAPKGPQIR
TAQEVDTYRR
TGTAYTFFTPNNIK
TLSYLLPAIVHINHQPFLER
TQNGVYSAANYTNGSFGSNFVSAGIQTSFR
TTYLVLDEADRMoxLDMoxGFEPQIR
WNLDELPKFEK	
118	Desmin [Mus musculus]	P31001
	53,5	5,2	177	60	23	APSYGAGELLDFSLADAVNQEFLATR
ELEDRFASEANGYQDNIAR
FLEQQNAALAAEVNR
FLEQQNAALAAEVNRLK
HLREYQDLLNVK
HQIQSYTCcamEIDALKGTNDSLMoxR
IESLNEEIAFLKK
INLPIQTFSALNFR
INLPIQTFSALNFRETSPEQR
LLEGEESRINLPIQTFSALNFR
MoxALDVEIATYR
MoxALDVEIATYRK
NISEAEEWYK
NISEAEEWYKSK
QAKQEMoxMoxEYR
RIESLNEEIAFLK
RQVEVLTNQR
TFGGAPGFSLGSPLSSPVFPR
TIETRDGEVVSEATQQQHEVL
TNEKVELQELNDR
VAELYEEEMoxRELR
VDVERDNLIDDLQR
VSDLTQAANKNNDALR	
119	Dhdh protein [Mus musculus]	Q8K0E9
	36,6	5,7	209	53	23	AEEFAQKFNIPK
AEFGFDLSHIPR
AIGVTFPQDK
AIGVTFPQDKR
AVLCcamEKPMGVNAAEVR
AVLCcamEKPMoxGVNAAEVR
AVLCcamEKPMoxGVNAAEVREMoxVAK
DLNRAEEFAQK
ESPVVPLAESELLAEILEEAR
ESPVVPLAESELLAEILEEARK
EVLVQGTIGDLR
FFPAMEALR
FFPAMEALREVLVQGTIGDLR
FFPAMoxEALR
FFPAMoxEALREVLVQGTIGDLR
GLKESPVVPLAESELLAEILEEAR
KEFPPPVLGK
LWAPTELVVNGER
LWAPTELVVNGERK
SQGVFLMEAIWSR
SQGVFLMoxEAIWSR
VARAEFGFDLSHIPR
WGIVSAGLIANDFTTVLSSLPSSEHQVVAVAAR	
120	Dehydrogenase/reductase (SDR family) member 1 [Mus musculus]	Q99L04
	34	8,7	106	43	13	AGATVYITGR
ATAQEAQSLGGR
CcamVVALATDPNILNLSGK
GHYLCcamSVYGAR
GIGRGIALQLCcamK
GRLDVLVNNAYAGVQAILNTTNK
LAADCcamAHELRR
SLFEQVDR
SLFEQVDREQK
VAPMKGQVCcamVVTGASR
VAPMoxKGQVCcamVVTGASR
VLPSCcamDLAR
VLPSCcamDLARR	
121	Dihydrolipoamide S-acetyltransferase precursor [Mus musculus]	Q91ZB1
	67,9	8,8	196	40	18	AAPAAAAAMoxAPPGPR
ASALACcamLKVPEANSSWMoxDTVIR
GFDVASVMoxSVTLSCcamDHR
GLETIASDVVSLASK
LQPHEFQGGTFTISNLGMoxFGIK
QNHVVDVSVAVSTPAGLITPIVFNAHIK
QTIPHYYLSVDVNMoxGEVLLVRK
VAPAPAGVFTDIPISNIR
VPLPSLSPTMoxQAGTIAR
VPLPSLSPTMQAGTIAR
VVDGAVGAQWLAEFKK
YLEKPITMoxLL	
122	Dihydrolipoamide dehydrogenase [Mus musculus]	O08749
	54,3	8	142	51	23	AEVITCcamDVLLVCcamIGR
ALLNNSHYYHMAHGK
ALLNNSHYYHMAHGKDFASR
ALLNNSHYYHMoxAHGK
ALLNNSHYYHMoxAHGKDFASR
ALTGGIAHLFK
ALTGGIAHLFKQNK
EANLAAAFGKPINF
IGKFPFAANSR
ILGHKSTDR
IPNIYAIGDVVAGPMLAHK
IPNIYAIGDVVAGPMoxLAHK
IPVNNRFQTK
LGADVTAVEFLGHVGGIGIDMoxEISK
LGADVTAVEFLGHVGGIGIDMoxEISKNFQR
RPFTQNLGLEELGIELDPK
RPFTQNLGLEELGIELDPKGR
SEEQLKEEGIEFK
TVCcamIEKNETLGGTCcamLNVGCcamIPSK
VCcamHAHPTLSEAFR
VCcamHAHPTLSEAFREANLAAAFGKPINF
VLGAHILGPGAGEMoxVNEAALALEYGASCcamEDIAR
VVHVNGFGKITGK	
123	ODO2_RAT Dihydrolipoyllysine-residue succinyltransferase component of 2-oxoglutarate dehydrogenase complex, mitochondrial precursor (Dihydrolipoamide succinyltransferase component of 2-oxoglutarate dehydrogenase complex) (E2) (E2K)	Q01205
	47,4	8,1	93	32	14	AAVEDPAVLLLDL
ASAFALQEQPVVNAVIDDATK
DYIDISVAVATPR
EVVYRDYIDISVAVATPR
KMoxVINTVAVFSVR
LIDGREAVTFLR
NDVITVQTPAFAESVTEGDVR
NDVITVQTPAFAESVTEGDVRWEK
NVETMNYADIER
NVETMoxNYADIER
oxVEVRPMMYVALTYDHR
VEGGTPLFTLR
VEGGTPLFTLRK
VEVRPMoxMoxYVALTYDHR	
124	Dihydrolipoamide S-succinyltransferase (E2 component of 2-oxo-glutarate complex) [Mus musculus]	Q9D2G2
	49	9,1	125	39	16	AAVEDPRVLLLDL
AKPAETPAPAHK
ASAFALQEQPVVNAVIDDATK
DYIDISVAVATPR
EVVYRDYIDISVAVATPR
HNLKLGFMoxSAFVK
LIDGREAVTFLR
LKEAQNTCcamAMoxLTTFNEVDMoxSNIQEMoxR
NDVITVQTPAFAESVTEGDVR
NDVITVQTPAFAESVTEGDVRWEK
NVETMNYADIER
NVETMoxNYADIER
oxVEVRPMMYVALTYDHR
VEGGTPLFTLR
VEGGTPLFTLRK
VEVRPMoxMoxYVALTYDHR	
125	Dimethylglycine dehydrogenase precursor [Mus musculus]	Q9DBT9
	97,3	7,6	184	29	17	DGLLFGPYESQEK
DLEGSYYLRQER
DSTQLLDHLFANVIPK
ESYGFNNIVGYPKEER
IMoxSAGQEEGIGDFGTYALNALRLEK
ISYTGELGWELYHR
IVNAAGFWAR
LATTPVRVDEFK
LEEETGQVVGFHQPGSIR
LTSEDLSDDAFKFLQTK
LYERLEEETGQVVGFHQPGSIR
NYPATIIQEPLVLTEPAR
SFNISDIPVTAIR
SLAFAYVPVQLSEVGQQVEVELLGK
VGFTNISHMoxLTPR
VGVIDLSPFGK
VVGNTTSGSYSYSIQK	
126	Enoyl coenzyme A hydratase 1, peroxisomal; peroxisomal/mitochondrial dienoyl-CoA isomerase; dienoyl-CoA isomerase [Mus musculus]	O35459
	36,1	7,4	162	63	23	APEEVSDHNYESIQVTSAQK
CcamPKPVIAAIHGGCcamIGGGVDLVSACcamDIR
DAMLNAAFALAADISSK
DAMoxLNAAFALAADISSK
ELVECcamFQK
EVDMGLAADVGTLQR
EVDMoxGLAADVGTLQR
HVLHVQLNRPEKR
IAWYLRDLISK
KMoxMoxADEALDSGLVSR
LPKVIGNQSLVNELTFSAR
MoxFTSGIDLMoxDMoxASELMoxQPSGDDAAR
MoxMoxADEALDSGLVSR
oxMFTSGIDLMDMASELMQPSGDDAAR
RAPEEVSDHNYESIQVTSAQK
SLSSSAQEASKR
SVQAAMEKR
VFQDKDAMLNAAFALAADISSK
VFQDKDAMoxLNAAFALAADISSK
VIGNQSLVNELTFSAR
VIGNQSLVNELTFSARK
YCcamTQDAFFQIK
YCcamTQDAFFQIKEVDMoxGLAADVGTLQR	
127	enoyl Coenzyme A hydratase, short chain, 1, mitochondrial [Mus musculus]	Q8BH95
	31,5	8,8	121	59	16	AFAAGADIKEMoxQNR
AFAAGADIKEMQNR
ALNALCcamNGLIEELNQALETFEQDPAVGAIVLTGGDK
AQFGQPEILLGTIPGAGGTQR
EGMoxTAFVEKR
ESVNAAFEMoxTLTEGNKLEK
GKNSSVGLIQLNRPK
IFPVEKLVEEAIQCcamAEK
LFYSTFATDDRR
LVEEAIQCcamAEK
NSSVGLIQLNRPK
oxSLAMEMVLTGDR
oxSLAMEMVLTGDRISAQDAK
SLAMoxEMoxVLTGDR
SLAMoxEMoxVLTGDRISAQDAK
TFQDCcamYSSK	
128	Eukaryotic translation elongation factor 1 alpha 1; elongation factor Tu; elongation factor 1 A-1; unnamed protein product [Mus musculus]	P10126
	50,1	9,2	83	39	17	DMoxRQTVAVGVIK
DMRQTVAVGVIK
EHALLAYTLGVK
FAVRDMoxR
IGGIGTVPVGR
NGQTREHALLAYTLGVK
NMITGTSQADCcamAVLIVAAGVGEFEAGISK
NMoxITGTSQADCcamAVLIVAAGVGEFEAGISK
oxSGDAAIVDMVPGKPMCcamVESFSDYPPLGR
SGDAAIVDMoxVPGKPMoxCcamVESFSDYPPLGR
THINIVVIGHVDSGK
THINIVVIGHVDSGKSTTTGHLIYK
VETGVLKPGMoxVVTFAPVNVTTEVK
VETGVLKPGMVVTFAPVNVTTEVK
YEEIVKEVSTYIK
YYVTIIDAPGHR
YYVTIIDAPGHRDFIK	
129	Eukaryotic translation elongation factor 1 delta (guanine nucleotide exchange protein) isoform b [Mus musculus]	P57776
	31,3	5	107	53	13	ATAPQTQHVSPMoxR
ATAPQTQHVSPMoxRQVEPPTK
ATAPQTQHVSPMR
FYEQMNGPVTSGSR
FYEQMoxNGPVTSGSR
GVVQDLQQAISKLEAR
ITSLEVENQNLR
LVPVGYGIRK
QENGASVILRDIAR
RFYEQMoxNGPVTSGSR
SIQLDGLVWGASK
SLAGSSGPGASSGPGGDHSELIVR
SSILLDVKPWDDETDMoxAQLETCcamVR	
130	L-specific multifunctional beta-oxdiation protein; L-bifunctional enzyme [Mus musculus]	Q9DBM2
	78,3	9,2	258	57	36	AIVICcamGANDNFCcamAGADIHGFK
ALQYAFFAEK
CcamLYSLINEAFR
ELSSVDLVIEAVFEDMoxNLK
FAQTVIGKPIEPR
GQGLTGPSLPPGTPTR
GQGLTGPSLPPGTPTRK
GWYQYDKPLGR
HPYEVAIKEEAK
HVGGPMoxYYAASVGLPTVLEK
IGKIGVVVGNCcamYGFVGNR
IGVVVGNCcamYGFVGNR
IHKPDPWLSEFLSQYR
ILNKPVPSLPNMoxDSVFAEAIAK
LCcamNPPVNAISPTVITEVR
LFMoxYLRGSGQAR
LFMYLRGSGQAR
MoxAEYLRLPHSLAMoxIR
QNPDIPQLEPSDYLR
QNPDIPQLEPSDYLRR
RILNKPVPSLPNMoxDSVFAEAIAK
SANKWSTPSGASWK
SGQASAKPNLR
SISKEEILER
TASAQPVSSVGVLGLGTMGR
TASAQPVSSVGVLGLGTMoxGR
TGKGWYQYDKPLGR
VGFPEVMLGILPGAR
VGFPEVMoxLGILPGAR
VGIPVVAVESDPK
VVGVPVALDLITSGR
YQKPVVAAIQGVALGGGLELALGCcamHYR
YSPIADMLCcamEAGR
YSPIADMoxLCcamEAGR
YSSPTTIATVMoxSLSKR
YYRQNPDIPQLEPSDYLR	
131	Eukaryotic translation initiation factor 3, subunit 2 (beta); TGF-beta receptor binding protein; eukaryotic translation initiation factor 3, subunit 2 (beta, 36kD) [Mus musculus]	Q9QZD9
	36,4	5,3	173	54	15	EGDLLFTVAKDPIVNVWYSVNGER
FFHLAFEEEFGR
FFHLAFEEEFGRVK
GHFGPINSVAFHPDGK
IHYFDPQYFEFEFEA
ITSAVWGPLGECcamVIAGHESGELNQYSAK
LFDSTTLEHQK
LWDCcamETGKQLALLK
MKPILLQGHER
MoxKPILLQGHER
QINDIQLSR
SGEVLVNVKEHSR
SITQIKYNR
SYSSGGEDGYVR
VKGHFGPINSVAFHPDGK	
132	Eukaryotic translation initiation factor 3, subunit 4 (delta); translation initiation factor eIF3-p44; eiF3 delta; eukaryotic translation initiation factor 3, subunit 4 (delta, 44 kDa) [Mus musculus]	Q9Z1D1
	35,6	5,6	93,5	38	12	AIAGVSGFGYDHLILNVEWAKPSTN
CcamPYKDTLGPMoxQK
EKLPGELEPVQAAQSK
ETDLQELFRPFGSISR
GESMoxQPNRR
GFAFISFHR
GFAFISFHRR
ICcamKGDHWTTR
LPGELEPVQAAQSK
TGKYVPPSLR
VTNLSEDTRETDLQELFRPFGSISR
YVPPSLRDGASR	
133	Eukaryotic initiation factor 5A isoform I variant CD	P63242
	16,8	5,1	99	67	11	GRPCcamKIVEMoxSTSK
GRPCcamKIVEMSTSK
KYEDICcamPSTHNMoxDVPNIK
MADDLDFETGDAGASATFPMQCcamSALR
NDFQLIGIQDGYLSLLQDSGEVR
NDFQLIGIQDGYLSLLQDSGEVREDLR
NGFVVLKGRPCcamK
RNDFQLIGIQDGYLSLLQDSGEVR
VHLVGIDIFTGK
VHLVGIDIFTGKK
YEDICcamPSTHNMoxDVPNIKR	
134	Epoxide hydrolase 2, cytoplasmic [Mus musculus]	P34914
	62,5	5,9	376	70	38	ACcamGANLPENFSISQIFSQAMAAR
ACcamGANLPENFSISQIFSQAMoxAAR
AKPNEVVFLDDFGSNLKPAR
ASDETGFIAVHK
ATEIGGILVNTPEDPNLSK
AVASLNTPFMoxPPDPDVSPMoxK
AVASLNTPFMoxPPDPDVSPMoxKVIR
DFLLGAYQTEFPEGPTEQLMoxK
DIVLRPEMoxSK
DMoxGMoxVTILVHNTASALR
DMoxGMoxVTILVHNTASALRELEK
GFTTCcamIVTNNWLDDGDKR
GHIEDCcamGHWTQIEKPTEVNQILIK
ITFSQWVPLMDESYRK
ITFSQWVPLMoxDESYR
ITFSQWVPLMoxDESYRK
ITTEEEIEFYIQQFK
ITTEEEIEFYIQQFKK
NMoxEKWIPFLK
oxDMGMVTILVHNTASALR
oxDMGMVTILVHNTASALRELEK
oxILVPALMVTAEKDIVLRPEMSK
oxVRAVASLNTPFMPPDPDVSPMK
RGHIEDCcamGHWTQIEKPTEVNQILIK
RSEEALALPR
SEEALALPRDFLLGAYQTEFPEGPTEQLMoxK
SINRPMLQAAIALK
SINRPMLQAAIALKK
SINRPMoxLQAAIALK
SINRPMoxLQAAIALKK
SIPVFNYQLYFQEPGVAEAELEKNMoxSR
TGFRGPLNWYR
VAAFDLDGVLALPSIAGAFR
VAAFDLDGVLALPSIAGAFRR
VTGTQFPEAPLPVPCcamNPNDVSHGYVTVKPGIR
WLQTEVQNPSVTSK
WLQTEVQNPSVTSKI
YQIPALAQAGFR	
135	Endoplasmic reticulum protein ERp29 precursor; endoplasmic retuclum protein 29 [Mus musculus]	P57759
	28,8	5,7	177	61	14	DGDLENPVLYNGAVK
ESYPVFYLFR
FDTQYPYGEK
FDTQYPYGEKQDEFK
FVLVKFDTQYPYGEK
GALPLDTVTFYK
GALPLDTVTFYKVIPK
GQGVYLGMoxPGCcamLPAYDALAGEFIK
ILDQGEDFPASEMAR
ILDQGEDFPASEMoxAR
LAENSASSEELLVAEVGISDYGDKLNMoxELSEK
LDKESYPVFYLFR
QAILKQGQDGLLSVK
SLNILTAFRK	
136	Esterase D/formylglutathione hydrolase; esterase 10; unnamed protein product [Mus musculus]	Q9R0P3
	31,3	6,5	177	59	19	AFSGYLGPDESKWK
AYDATCcamLVK
FAVYLPPQAESGK
LQEGYDHSYYFIATFIADHIR
MoxSIFGHSMoxGGHGALICcamALKNPGK
MoxYSYVTEELPQLINANFPVDPQR
SVSAFAPICcamNPVLCcamSWGK
SVSAFAPICcamNPVLCcamSWGKK
VFEHSSVELK
VWADERQCcamSGR	
137	Electron transferring flavoprotein, alpha polypeptide; Alpha-ETF [Mus musculus]	Q99LC5
	35	8,6	168	73	22	AAVDAGFVPNDMoxQVGQTGK
AAVDAGFVPNDMQVGQTGK
GLLPEELTPLILETQK
GLLPEELTPLILETQKQFSYTHICcamAGASAFGK
GTSFEAAATSGGSASSEKAPSSSSVGISEWLDQK
IVAPELYIAVGISGAIQHLAGMK
IVAPELYIAVGISGAIQHLAGMKDSK
IVAPELYIAVGISGAIQHLAGMoxK
IVAPELYIAVGISGAIQHLAGMoxKDSK
LGGEVSCcamLVAGTK
LLYDLADQLHAAVGASR
LNVAPVSDIIEIK
LNVAPVSDIIEIKSPDTFVR
QFSYTHICcamAGASAFGK
SDPPELTGAKVVVSGGR
SGENFKLLYDLADQLHAAVGASR
TIVAINKDPEAPIFQVADYGIVADLFK
TIYAGNALCcamTVK
VLVAQHDAYK
VLVAQHDAYKGLLPEELTPLILETQK
VVPEMoxTEILK
VVPEMTEILK	
138	Etfb protein [Mus musculus]	Q9DCW4
	27,6	8,6	93	57	15	AGDLGVDLTSKVSVISVEEPPQR
EIDGGLETLR
EIDGGLETLRLK
EIIAVSCcamGPSQCcamQETIR
GIHVEIPGAQAESLGPLQVAR
HSMNPFCcamEIAVEEAVR
HSMoxNPFCcamEIAVEEAVR
LKLPAVVTADLR
LPAVVTADLRLNEPR
LVKEIIAVSCcamGPSQCcamQETIR
MoxAELRALVAVK
TALAMGADRGIHVEIPGAQAESLGPLQVAR
VETTEDLVAK
VIDFAVKIR
VSVISVEEPPQR	
139	ETHE1 protein [Mus musculus]	Q9DCM0
	27,7	6,6	187	85	19	ASPGHTPGCcamVTFVLNDQSMAFTGDALLIR
ASPGHTPGCcamVTFVLNDQSMoxAFTGDALLIR
DAQLIKELGLK
EAVLIDPVLETAHR
EAVLIDPVLETAHRDAQLIK
GCcamGRTDFQQGCcamAK
IFTLPGNCcamLIYPAHDYHGLTVSTVEEER
LLYAVNTHCcamHADHITGTGVLR
LSGAQADLHIGEGDSIR
LSGAQADLHIGEGDSIRFGR
LSQQSASGAPVLLR
LTLSCcamEEFIK
oxVMDNLNLPKPQQIDIAVPANMR
SCcamTYTYLLGDR
SCcamTYTYLLGDRESR
SLLPGCcamQSVISR
SLLPGCcamQSVISRLSGAQADLHIGEGDSIR
TLYHSVHEK
VMoxDNLNLPKPQQIDIAVPANMoxR	
140	Fatty acid binding protein 1, liver; fatty acid binding protein liver [Mus musculus]	P12710
	14,2	8,9	111	81	19	AIGLPEDLIQK
AIGLPEDLIQKGK
GVSEIVHEGK
GVSEIVHEGKK
IKLTITYGPK
LTITYGPK
MoxVTTFKGIK
MVTTFKGIK
NEFTLGEECcamELETMoxTGEK
NEFTLGEECcamELETMoxTGEKVK
NEFTLGEECcamELETMTGEK
NEFTLGEECcamELETMTGEKVK
SVTELNGDTITNTMoxTLGDIVYKR
SVTELNGDTITNTMTLGDIVYK
SVTELNGDTITNTMTLGDIVYKR
VVRNEFTLGEECcamELETMoxTGEK
VVRNEFTLGEECcamELETMTGEK
YQLQSQENFEPFMK
YQLQSQENFEPFMoxK	
141	Fatty acid binding protein 5, epidermal; keratinocyte lipid binding protein; Fabpe E-FABP; Unknown Klbp, keratinocyte lipid binding protein [Mus musculus]	Q05816
	15,1	6,2	149	82	17	ELGVGLALRK
KMoxAAMoxAKPDCcamIITCcamDGNNITVK
KTETVCcamTFQDGALVQHQQWDGK
LMESHGFEEYMK
LMoxESHGFEEYMoxK
LMoxESHGFEEYMoxKELGVGLALR
MIVECcamVMNNATCcamTR
MoxAAMoxAKPDCcamIITCcamDGNNITVK
MoxAAMoxAKPDCcamIITCcamDGNNITVKTESTVK
MoxIVECcamVMoxNNATCcamTR
oxLMESHGFEEYMK
oxLMESHGFEEYMKELGVGLALR
oxMAAMAKPDCcamIITCcamDGNNITVK
oxMIVECcamVMNNATCcamTR
TETVCcamTFQDGALVQHQQWDGK
TETVCcamTFQDGALVQHQQWDGKESTITR
TTVFSCcamNLGEKFDETTADGR	
142	Fumarylacetoacetate hydrolase [Mus musculus]	P35505
	46,2	6,8	81	34	14	AIDVEQGQTR
ASLQNLLSASQAR
ASSIVVSGTPIR
DIQQWEYVPLGPFLGK
ENALLPNWLHLPVGYHGR
GKENALLPNWLHLPVGYHGR
HLFTGPALSK
IGVAIGDQILDLSVIK
IGVAIGDQILDLSVIKHLFTGPALSK
oxLLDMELEMAFFVGPGNR
QHATNVGIMFR
QHATNVGIMoxFR
TFLLDGDEVIITGHCcamQGDGYR	
143	Liver fructose-1,6-bisphosphatase [Mus musculus]	Q9QXD6
	36,9	6,2	281	71	28	AGGLATTGDKDILDIVPTEIHQK
APVVMGSSEDVQEFLEIYRK
APVVMoxGSSEDVQEFLEIYRK
AQGTGELTQLLNSLCcamTAIK
AQGTGELTQLLNSLCcamTAIKAISSAVR
DFDPAINEYLQR
DFDPAINEYLQRK
FPPDGSAPYGAR
GKYVVCcamFDPLDGSSNIDCcamLVSIGTIFGIYR
GNIYSLNEGYAK
GNIYSLNEGYAKDFDPAINEYLQR
KAQGTGELTQLLNSLCcamTAIK
KLDILSNDLVINMLK
KLDILSNDLVINMoxLK
LLYECcamNPIAYVMEK
LLYECcamNPIAYVMoxEK
LRLLYECcamNPIAYVMEK
LRLLYECcamNPIAYVMoxEK
QAGIAQLYGIAGSTNVTGDQVK
SSYATCcamVLVSEENTNAIIIEPEKR
TLVYGGIFLYPANK
TLVYGGIFLYPANKK
YVGSMoxVADIHR
YVGSMoxVADIHRTLVYGGIFLYPANK
YVGSMVADIHR
YVGSMVADIHRTLVYGGIFLYPANK
YVVCcamFDPLDGSSNIDCcamLVSIGTIFGIYR
YVVCcamFDPLDGSSNIDCcamLVSIGTIFGIYRK	
144	Fibrinogen, B beta polypeptide [Mus musculus]	Q8K0E8
	54,8	6,5	238	68	37	AHYGGFTVQNEASK
AHYGGFTVQNEASKYQVSVNK
EDGGGWWYNR
EEPPSLRPAPPPISGGGYR
ENENVINEYSSILEDQR
GFGNIATNEDAK
GFGNIATNEDAKK
GGETSEMYLIQPDTSIKPYR
GSWYSMoxRR
GSWYSMRR
HGTDDGVVWMNWK
HGTDDGVVWMoxNWK
IQKLESDISAQMEYCcamR
IQKLESDISAQMoxEYCcamR
IRPFFPQQ
KEEPPSLRPAPPPISGGGYR
KGGETSEMoxYLIQPDTSIKPYR
KGGETSEMYLIQPDTSIKPYR
KYCcamGLPGEYWLGNDK
LESDISAQMEYCcamR
LYIDETVNDNIPLNLR
LYIDETVNDNIPLNLRVLR
MoxSMoxKIRPFFPQQ
oxTMTIHNGMFFSTYDRDNDGWVTTDPR
QAQVKENENVINEYSSILEDQR
QCcamSKEDGGGWWYNR
QDGSVDFGRK
SILEDLRSK
SSIAELNNNIQSVSDTSSVTFQYLTLLKDMoxWK
TENGGWTVIQNR
TPCcamTVSCcamNIPVVSGK
TPCcamTVSCcamNIPVVSGKECcamEEIIR
VYCcamDMKTENGGWTVIQNR
VYCcamDMoxKTENGGWTVIQNR
YCcamGLPGEYWLGNDKISQLTR
YYWGGLYSWDMoxSK
YYWGGLYSWDMSK	
145	Fumarate hydratase 1 [Mus musculus]	P97807
	54,4	9,2	143	46	18	AAAEVNQEYGLDPK
AIEMoxLGGELGSK
IYELAAGGTAVGTGLNTR
KPVHPNDHVNK
LMoxNESLMoxLVTALNPHIGYDK
LNDHFPLVVWQTGSGTQTNMoxNVNEVISNR
MoxPIPVIQAFGILK
MoxPIPVIQAFGILKR
MPIPVIQAFGILKR
SGLGELILPENEPGSSIMoxPGK
SGLGELILPENEPGSSIMPGK
SKEFAQVIK
SQSSNDTFPTAMoxHIAAAVEVHK
STMoxNFKIGGATER
THTQDAVPLTLGQEFSGYVQQVQYAMoxVR
THTQDAVPLTLGQEFSGYVQQVQYAMVR
VLLPGLQK
VPTDKYYGAQTVR
YYGAQTVR	
146	Formiminotransferase cyclodeaminase; glutamate formiminotransferase [Mus musculus]	Q91XD4
	58,9	5,7	319	62	33	AFAACcamLEAIKLPK
AISQTPGCcamVLLDVDAGPSTNR
ALLDAAAFYCcamDKEK
EAQELNLPVVGSQLVGLVPLK
FLIAFNINLLSTK
FLIAFNINLLSTKEQAHR
GVSMEECcamVLCcamAK
IALNLREQGR
IIEYLVPDSGPEQSLLDTSLR
IIEYLVPDSGPEQSLLDTSLRGFVR
ISSLLQEAKTQAALVLGSLEAR
KFLIAFNINLLSTK
KVQGIGWYLEEK
LFVLEEEHR
LFVLEEEHRIR
LGLDSLAPFDPK
LGLDSLAPFDPKER
LVVNRLGLDSLAPFDPK
MGALDVCcamPFIPVR
MoxGALDVCcamPFIPVR
QAEWVPDFGPSSFVPSWGATVTGAR
QFDHLDSTMoxR
QFDHLDSTMoxRR
QFDHLDSTMR
QFDHLDSTMRR
RACcamALQEGLR
REAQELNLPVVGSQLVGLVPLK
RLIPPFHAASAQLTSLVDADAR
SAAPGGGSVAAAVAALGAALASMoxVGQMoxTYGR
SAAPGGGSVAAAVAALGAALASMoxVGQMoxTYGRR
TQAALVLGSLEAR
TVYTFVGQPECcamVVEGALHAAR
VQGIGWYLEEK	
147	Ferritin heavy chain 1 [Mus musculus]	P09528
	21,1	5,4	134	55	10	ELGDHVTNLR
ELGDHVTNLRK
LATDKNDPHLCcamDFIETYYLSEQVK
MoxGAPEAGMoxAEYLFDKHTLGHGDES
oxMGAPEAGMAEYLFDKHTLGHGDES
QNYHQDAEAAINR
SIKELGDHVTNLR
SVNQSLLELHK
YFLHQSHEER
YFLHQSHEEREHAEK	
148	Ferritin light chain 1 [Mus musculus]	P29391
	20,8	5,5	133	78	11	ADPHLCcamDFLESHYLDKEVK
ALFQDVQKPSQDEWGK
ASYTYLSLGFFFDRDDVALEGVGHFFR
LLEFQNDRGGR
MoxGNHLTNLR
MoxGNHLTNLRR
NLNQALLDLHALGSAR
QNYSTEVEAAVNR
QNYSTEVEAAVNRLVNLHLR
RVAGPQPAQTGAPQGSLGEYLFER
VAGPQPAQTGAPQGSLGEYLFER	
149	Ras-GTPase-activating protein SH3-domain binding protein; GAP SH3 binding protein [Mus musculus]	P97855
	51,8	5,3	170	50	21		
150	Galactokinase 1 [Mus musculus]	Q9R0N0 
	42,3	5,1	192	46	21	AEHSFAGVPCcamGIMDQLIALLGQK
AEHSFAGVPCcamGIMoxDQLIALLGQK
AFMEEFGAEPELAVSAPGR
AFMoxEEFGAEPELAVSAPGR
ARHVVSEIR
EVRMoxEELEAGR
GYALLIDCcamR
HSLGSSEYPVR
HSLGSSEYPVRR
LAVLITNSNVR
LMVESHYSLR
MEELEAGRELMSK
MoxEELEAGRELMoxSK
oxMEELEAGRELMSK
QCcamEEVAQALGKESLR
RAFMEEFGAEPELAVSAPGR
RAFMoxEEFGAEPELAVSAPGR
RQCcamEEVAQALGK
RTAQAAAAMoxSR
SLETSLVPLSDPKLAVLITNSNVR
VNLIGEHTDYNQGLVLPMoxALELVTVMoxVGSPR	
151	Aldose 1-epimerase [Mus musculus]	Q8K157 	37,8	6,1	146	48	13	ASDVVLGFAELEGYLQK
ASDVVLGFAELEGYLQKQPYFGAVVGR
EPNSLHGGFTGFDK
EYHLPVNREPNSLHGGFTGFDK
FTIGGKEYHLPVNR
ILEVYTTQPGVQFYTGNFLDGTLK
KPVELGTHLQDYHIHGFDHNFCcamLK
KPVELGTHLQDYHIHGFDHNFCcamLKESK
QPYFGAVVGR
SDQLSVDIISWGCcamTITALQVKDR
TVFGELPSGGGTVEK
TVFGELPSGGGTVEKFQLR
VLWTPQVLTNGVQFFR	
152	Guanidinoacetate methyltransferase [Mus musculus]	O35969 	26,3	5,3	155	68	17	AAPAAYDASDTHLQILGKPVMER
AAPAAYDASDTHLQILGKPVMoxER
ENICcamTEVMALVPPADCcamR
ENICcamTEVMoxALVPPADCcamR
GGRVLEVGFGMAIAASR
GGRVLEVGFGMoxAIAASR
LQDWALRQPHK
TGGVLTYCcamNLTSWGELMK
TGGVLTYCcamNLTSWGELMoxK
VLEVGFGMAIAASR
VLEVGFGMoxAIAASR
VQQAPIEEHWIIECcamNDGVFQR
WETPYMHALAAAAASR
WETPYMoxHALAAAAASR
YTDITTMFEETQVPALQEAGFLK
YTDITTMoxFEETQVPALQEAGFLK
YYAFPQMITPLVTK
YYAFPQMITPLVTKH
YYAFPQMoxITPLVTK
YYAFPQMoxITPLVTKH	
153	Glyceraldehyde 3-phosphate-dehydrogenase [Rattus norvegicus]	P04797 	35,8	8,4	118	50	17	DGRGAAQNIIPASTGAAK
GAAQNIIPASTGAAK
GAAQNIIPASTGAAKAVGK
IVSNASCcamTTNCcamLAPLAK
LISWYDNEYGYSNR
LVINGKPITIFQERDPVK
LVTRAAFSCcamDK
oxVVDLMAYMASKE
TVDGPSGKLWR
VIHDNFGIVEGLMoxTTVHAITATQK
VIHDNFGIVEGLMTTVHAITATQK
VIPELNGKLTGMAFR
VIPELNGKLTGMoxAFR
VKVGVNGFGR
VPTPNVSVVDLTCcamR
VVDLMAYMASKE
VVDLMoxAYMoxASKE	
154	Glyceraldehyde-3-phosphate dehydrogenase [Mus musculus]	P16858 
	35,8	8,4	170	68	23	AENGKLVINGKPITIFQER
DGRGAAQNIIPASTGAAK
GAAQNIIPASTGAAK
IVSNASCcamTTNCcamLAPLAK
LISWYDNEYGYSNR
LTGMAFRVPTPNVSVVDLTCcamR
LTGMoxAFRVPTPNVSVVDLTCcamR
LVINGKPITIFQER
LVINGKPITIFQERDPTNIK
oxVEIVAINDPFIDLNYMVYMFQYDSTHGK
oxVIISAPSADAPMFVMGVNHEKYDNSLK
oxVVDLMAYMASKE
TVDGPSGKLWR
VIHDNFGIVEGLMoxTTVHAITATQK
VIHDNFGIVEGLMoxTTVHAITATQKTVDGPSGK
VIHDNFGIVEGLMTTVHAITATQK
VIPELNGKLTGMAFR
VIPELNGKLTGMoxAFR
VPTPNVSVVDLTCcamR
VVDLMAYMASKE
VVDLMoxAYMoxASKE
WGEAGAEYVVESTGVFTTMEK
WGEAGAEYVVESTGVFTTMoxEK	
155	Vitamin D-binding protein [Mus musculus]	P21614 	53,1	5,2	229	61	26	CcamCcamESTSEDCcamMoxASELPEHTIK
DLCcamGQSTTQAMDQYTFELSR
DLCcamGQSTTQAMoxDQYTFELSR
ECcamCcamDTQDSVACcamFSTQSPLLK
ECcamCcamDTQDSVACcamFSTQSPLLKR
EVVSLTEECcamCcamAEGADPTCcamYDTR
FSSSTFEQVNQLVK
GFADQFLYEYSSNYGQAPLPLLVAYTK
GQEMoxCcamADYSENTFTEYK
HLSLLTTMoxSNR
HLSLLTTMSNR
KFSSSTFEQVNQLVK
LAQKVPTANLENVLPLAEDFTEILSR
LQMoxKHLSLLTTMoxSNR
NYLSMoxVGSCcamCcamTSANPTVCcamFVK
NYLSMVGSCcamCcamTSANPTVCcamFVK
RTQVPEVFLSK
SCcamESDAPFPVHPGTPECcamCcamTK
SLSLILYSR
TLRECcamCcamDTQDSVACcamFSTQSPLLK
TPNTSPAELKDMoxVEK
TQVPEVFLSK
VCcamNELAMLGKEDFR
VCcamSQYAAYGK
VCcamSQYAAYGKEK
VPTANLENVLPLAEDFTEILSR	
156	Glutamate cysteine ligase, modifier subunit; 	O09172 	30,5	5,2	79	35	7	AAGALLARASTLHLQTGNLLNWGR
ASTLHLQTGNLLNWGR
CcamPSTHSEELRDCcamIQK
EFPDVLECcamTMoxSHAVEK
LFIVGSNSSSSTR
QFDIQLLTHNDPK
TLNEWSSQISPDLVR	
157	Guanine deaminase [Mus musculus]	Q9R111 	51	5,3	174	51	20	ASDSPIDLFYGDFVGDISEAVIQK
EFDALLINPR
FLYLGDDRNIEEVYVGGK
FRSTDVAEEVYTR
FTLSCcamTETLMoxSELGNIAK
FTLSCcamTETLMSELGNIAK
FVSEMoxLQKNYPR
GASIAHCcamPNSNLSLSSGLLNVLEVLK
GASIAHCcamPNSNLSLSSGLLNVLEVLKHK
IGLGTDVAGGYSYSMLDAIR
IGLGTDVAGGYSYSMoxLDAIRR
IVFLEESSQQEK
NLTLKEVFR
NYTDVYDKNNLLTNK
STDVAEEVYTR
STDVAEEVYTRVVR
THDLYIQSHISENREEIEAVK
TVMAHGCcamYLSEEELNIFSER
TVMoxAHGCcamYLSEEELNIFSER	
158	Glyoxalase 1 (Unknown ) (protein for MGC:101960) [Mus musculus]	Q9CPU0 	20,8	5,1	162	72	15	DFLLQQTMoxLR
FSLYFLAYEDKNDIPK
GFGHIGIAVPDVYSACcamK
GFGHIGIAVPDVYSACcamKR
GLAFIQDPDGYWIEILNPNK
KATLELTHNWGTEDDETQSYHNGNSDPR
KSLDFYTR
LDFPAMK
RFEELGVK
SEKTAWTFSR
SLDFYTR
TAWTFSR
TAWTFSRK
VLGLTLLQK
VLGLTLLQKLDFPAMK
VLGLTLLQKLDFPAMoxK	
159	Glutamate dehydrogenase 1 [Mus musculus]	P26443 	61,3	8	271	60	35	AKVYEGSILEADCcamDILIPAASEK
AQHSQHRTPCcamK
CcamAVVDVPFGGAK
CcamVGVGESDGSIWNPDGIDPK
DDGSWEVIEGYR
DDGSWEVIEGYRAQHSQHR
DIVHSGLAYTMER
DSNYHLLMoxSVQESLER
DSNYHLLMSVQESLER
ELEDFKLQHGSILGFPK
FGKHGGTIPVVPTAEFQDR
GFIGPGIDVPAPDMoxSTGER
GGIRYSTDVSVDEVK
GVFHGIENFINEASYMoxSILGMoxTPGFGDK
HGGTIPVVPTAEFQDR
IIKPCcamNHVLSLSFPIR
IIKPCcamNHVLSLSFPIRR
ISGASEKDIVHSGLAYTMER
ISGASEKDIVHSGLAYTMoxER
KGFIGPGIDVPAPDMoxSTGER
KGFIGPGIDVPAPDMSTGER
NIMoxVIPDLYLNAGGVTVSYFEWLK
NLNHVSYGR
NYTDNELEK
NYTDNELEKITR
oxGVFHGIENFINEASYMSILGMTPGFGDK
RDDGSWEVIEGYR
TAAYVNAIEKVFK
TAMKYNLGLDLR
TAMoxKYNLGLDLR
TFVVQGFGNVGLHSMoxR
TFVVQGFGNVGLHSMR
VFKVYNEAGVTFT
VYEGSILEADCcamDILIPAASEK
YSTDVSVDEVKALASLMTYK	
160	Glutamate-ammonia ligase (glutamine synthase); glutamine synthetase [Mus musculus]	P15105 	42,1	6,5	274	67	28	ACcamLYAGVK
AMoxREENGLK
AYDPKGGLDNAR
AYGRDIVEAHYR
CcamIEEAIDKLSK
DIVEAHYR
DPNKLVLCcamEVFK
GIKQMoxYMoxSLPQGEK
GYFEDRRPSANCcamDPYAVTEAIVR
ITGTNAEVMoxPAQWEFQIGPCcamEGIR
ITGTNAEVMPAQWEFQIGPCcamEGIR
KGYFEDR
KPAETNLR
LTGFHETSNINDFSAGVANR
LVLCcamEVFKYNR
MATSASSHLNK
MGDHLWIAR
MoxGDHLWIAR
QMoxYMoxSLPQGEK
RHQYHIR
RLTGFHETSNINDFSAGVANR
RPSANCcamDPYAVTEAIVR
TCcamLLNETGDEPFQYK
TCcamLLNETGDEPFQYKN
VCcamEDFGVIATFDPKPIPGNWNGAGCcamHTNFSTK
VQAMoxYIWVDGTGEGLR
VQAMYIWVDGTGEGLR
YNRKPAETNLR	
161	G-beta like protein; Guanine nucleotide-binding protein subunit beta 2-like 1; [Mus musculus]	P68040 	31	8,1	174	67	15	AEPPQCcamTSLAWSADGQTLFAGYTDNLVR
DETNYGIPQR
DVLSVAFSSDNR
DVLSVAFSSDNRQIVSGSR
FSPNSSNPIIVSCcamGWDK
FSPNSSNPIIVSCcamGWDKLVK
FVGHTKDVLSVAFSSDNR
IIVDELKQEVISTSSK
LTRDETNYGIPQR
LWDLTTGTTTR
LWDLTTGTTTRR
TNHIGHTGYLNTVTVSPDGSLCcamASGGK
VWNLANCcamKLK
VWQVTIGTR
YTVQDESHSEWVSCcamVR
YWLCcamAATGPSIK	
162	Glycine N-methyltransferase [Mus musculus]	Q9QXF8 	32,7	6,9	214	65	18	AHMoxVTLDYTVQVPGTGR
AHMVTLDYTVQVPGTGR
CcamQHSVLGDFKPYKPGQAYVPCcamYFIHVLK
CcamQHSVLGDFKPYKPGQAYVPCcamYFIHVLKK
DGSPGFSKFR
FRLSYYPHCcamLASFTELVR
LALKNIASMoxVRPGGLLVIDHR
LSYYPHCcamLASFTELVR
LSYYPHCcamLASFTELVRAAFGGR
NIASMoxVRPGGLLVIDHR
NIASMVRPGGLLVIDHR
NYDYILSTGCcamAPPGK
NYDYILSTGCcamAPPGKNIYYK
oxVLDVACcamGTGVDSIMLVEEGFSVMSVDASDK
SLGVAAEGLPDQYADGEAAR
SLGVAAEGLPDQYADGEAARVWQLYIGDTR
TAEYKAWLLGLLR
VWQLYIGDTR	
163	Glutamate oxaloacetate transaminase 2, mitochondrial; mitochondrial aspartate aminotransferase; plasma membrane fatty acid binding protein [Mus musculus]	P05202 	47,4	9,2	208	66	25	ASAELALGENNEVLK
ASAELALGENNEVLKSGR
DAGMoxQLQGYR
DAGMQLQGYR
DDNGKPYVLPSVR
DGRISVAGVTSGNVGYLAHAIHQVTK
EGSSHNWQHITDQIGMFCcamFTGLKPEQVER
FVTVQTISGTGALR
FVTVQTISGTGALRVGASFLQR
HFIEQGINVCcamLCcamQSYAK
IAATILTSPDLR
IAATILTSPDLRK
IISMRTQLVSNLK
ILIRPLYSNPPLNGAR
ISVAGVTSGNVGYLAHAIHQVTK
LTKEFSVYMoxTK
LTKEFSVYMTK
MNLGVGAYRDDNGKPYVLPSVR
MoxNLGVGAYRDDNGKPYVLPSVR
NLDKEYLPIGGLAEFCcamK
NLFAFFDMoxAYQGFASGDGDKDAWAVR
QWLQEVKGMoxADR
SGRFVTVQTISGTGALR
TCcamGFDFSGALEDISK
VGAFTVVCcamKDAEEAK	
164	Glutathione peroxidase 1; cellular GPx [Mus musculus]	P11352 	22,3	6,2	215	86	16	AHPLFTFLR
CcamEVNGEKAHPLFTFLR
DYTEMoxNDLQKR
FLVGPDGVPVR
FLVGPDGVPVRR
FRTIDIEPDIETLLSQQSGNS
GLVVLGFPCcamNQFGHQENGKNEEILNSLK
LSAAAQSTVYAFSARPLTGGEPVSLGSLR
LSAAAQSTVYAFSARPLTGGEPVSLGSLRGK
NALPTPSDDPTALMoxTDPK
NDIAWNFEK
TIDIEPDIETLLSQQSGNS
YIIWSPVCcamR
YVRPGGGFEPNFTLFEK
YVRPGGGFEPNFTLFEKCcamEVNGEK	
165	Glyoxylate reductase/hydroxypyruvate reductase [Mus musculus]	Q91Z53 	35,3	7,3	187	50	14	AALAQAADCcamEVEQWNSDDPIPR
DLEQGVVGAHGLLCcamR
KDLEQGVVGAHGLLCcamR
KLLDAAGANLR
MKNTAIFINISR
MoxKNTAIFINISR
NCcamVILPHIGSATYK
NCcamVILPHIGSATYKTR
NTAIFINISR
NTMoxSLLAANNLLAGLR
NTMSLLAANNLLAGLR
RLKPFGVQR
RLPEAIEEVK
VFVTGPLPAEGR
VGYTPGVLTDATAELAVSLLLTTCcamR
VISTLSVGVDHLALDEIK
VISTLSVGVDHLALDEIKK	
166	GrpE-like 1, mitochondrial [Mus musculus]	Q99LP6 	24,3	8,6	148	62	12	ALADTENLRQR
ATQSVPKEEISNNNPHLK
FDPYEHEALFHTPVEGKEPGTVALVSK
HGLLRLDPIGAK
LYGIQGFCcamKDLLEVADILEK
RSLPALALSFRPSPR
SLYEGLVMoxTEVQIQK
SLYEGLVMoxTEVQIQKVFTK
TLRPALVGVVK
TLRPALVGVVKDA	
167	Glutathione synthetase [Mus musculus]	P51855 	52,2	5,5	180	63	23	AAKCcamPDIAIQLAGTK
ALKQIEINTISASFGGLASR
ASYILMoxEKIEPEPFR
ATSWGSILQDEK
AVEHADGGVAAGVAVLDNPYPV
AVENELLDR
AVENELLDRK
AWELYGSANAVVLLIAQEKER
CcamPDIAIQLAGTK
CcamPDIAIQLAGTKK
DGYMoxPSQYNSQNWEAR
EGGGNNLYGEEMoxVQALEQLKDSEER
EGIAQTVFLGLNR
GRFEDVSER
ILSNNPSKGLALGIAK
LFMoxDDQEVAVVYFR
NCcamLLRPGSPAQVVQCcamISELGIFGVYVR
QGTTLVMoxNKHVGHLLR
QIEINTISASFGGLASR
SDYMoxFQCcamGADGSK
TKAVEHADGGVAAGVAVLDNPYPV
TPAVHRHVLNVLNK
VGLLEALLPGQPEAVAR	
168	Glutathione S-transferase, alpha 3 [Mus musculus]	P30115 	25,4	8,8	170	64	14	AILNYIASKYNLYGK
KPFDDAKCcamVESAK
LRSDGSLMoxFQQVPMoxVEIDGMoxK
LSRADIALVELLYHVEELDPGVVDNFPLLK
MAGKPVLHYFDGR
MoxEPIRWLLAAAGVEFEEK
NRYFPAFEK
oxSDGSLMFQQVPMVEIDGMK
SDGSLMoxFQQVPMoxVEIDGMoxK
SDGSLMoxFQQVPMoxVEIDGMoxKLVQTK
SHGQDYLVGNR
VLKSHGQDYLVGNR
WLLAAAGVEFEEK
WLLAAAGVEFEEKFLK	
169	Glutathione S-transferase A4 A Chain A, Crystal Structure Of Mgsta4-4 In Complex With Gsh Conjugate Of 4-Hydroxynonenal In One Subunit And Gsh In The Other: Evidence Of Signaling Across Dimer Interface In Mgsta4-4	P24472 	25,4	7,6	105	48	9	AILSYLAAKYNLYGK
DGHLLFGQVPLVEIDGMoxMoxLTQTR
EKEESYDLILSR
KFLQPGSQR
KPPPDGPYVEVVR
TRYFPVFEK
WLLAAAGVEFEEEFLETR
WLLAAAGVEFEEEFLETREQYEK
YNLYGKDLK	
170	Glutathione S-transferase, mu 1; glutathione-S-transferase, mu 1 [Mus musculus]	P10649 	26	8,7	235	84	26	ADIVENQVMoxDTR
CcamLDAFPNLR
CcamLDAFPNLRDFLAR
FKLGLDFPNLPYLIDGSHK
IRADIVENQVMDTR
IRADIVENQVMoxDTR
ITQSNAILR
KHHLDGETEEER
LGLDFPNLPYLIDGSHK
LYSEFLGK
MLLEYTDSSYDEKR
MoxAHWSNK
MoxLLEYTDSSYDEKR
MoxQLIMoxLCcamYNPDFEK
MoxQLIMoxLCcamYNPDFEKQKPEFLK
MQLIMLCcamYNPDFEK
oxMQLIMLCcamYNPDFEK
PMILGYWNVR
PMoxILGYWNVR
RPWFAGDK
RYTMoxGDAPDFDR
VTYVDFLAYDILDQYRMoxFEPK
YIATPIFSK
YTMGDAPDFDRSQWLNEK
YTMoxGDAPDFDR
YTMoxGDAPDFDRSQWLNEK	
171	Gstm6 protein; unnamed protein product [Mus musculus]	O35660 	25,6	5,7	148	64	13	FKLDLDFPNLPYLIDGSHK
FLPSPVYLK
GLPDQLRLYSEFLGK
KHNLCcamGETEEER
LDLDFPNLPYLIDGSHKVTQSNAILR
LLLEYTETGYEEKR
PVTLGYWDIR
PVTLGYWDIRGLGHAIR	
172	Glutathione S-transferase omega 1; glutathione-S-transferase like; glutathione S-transferase like [Mus musculus]	O09131 	27,5	7,7	144	56	14	GIRHEVININLK
GSAPPGPVPEGQIR
GSAPPGPVPEGQIRVYSMoxR
LEALELKECcamLAHTPK
LFPDDPYKK
LWMoxAAMoxQQDPVASSHKIDAK
MoxTLESFSKVPPLIASFVR
MTLESFSKVPPLIASFVR
NKPEWFFEK
SLGKGSAPPGPVPEGQIR
TYREYLNLYLQDSPEACcamDYGL
VPPLIASFVR
VYSMoxRFCcamPFAQR
VYSMRFCcamPFAQR	
173	Glutathione S-transferase, pi 1; Gst p-1	P19157 	23,6	8,8	151	63	16	AFLSSPEHVNRPINGNGK
ALPGHLKPFETLLSQNQGGK
EAAQMDMVNDGVEDLRGK
EAAQMoxDMoxVNDGVEDLRGK
FEDGDLTLYQSNAILR
GRCcamEAMoxR
IKAFLSSPEHVNRPINGNGK
MoxPPYTIVYFPVR
MoxPPYTIVYFPVRGR
MPPYTIVYFPVR
oxCcamEAMRMLLADQGQSWK
oxEAAQMDMVNDGVEDLRGK
PPYTIVYFPVR
PPYTIVYFPVRGR
SLGLYGKNQR
YVTLIYTNYENGKNDYVK	
174	Glutathione S-transferase, pi 2 [Rattus norvegicus]	P46425 	23,4	6,5	111	54	11	ALPGHLKPFETLLSQNQGGK
EAALVDMoxVNDGVEDLR
EAALVDMoxVNDGVEDLRCcamK
EAALVDMVNDGVEDLR
FEDGDLTLYQSNAILR
MLLADQGQSWKEEVVTIDVWLQGSLK
PPYTIVYFPVR
PPYTIVYFPVRGR
SLGLYGKDQK
STCcamLYGQLPK
STCcamLYGQLPKFEDGDLTLYQSNAILR	
175	Glutathione S-transferase, theta 1 [Mus musculus]	Q64471 	27,4	6,6	106	45	19	ARVDEYLAWQHTGLR
KGEHLSDAFAR
NNIPFQMHTVELRK
NNIPFQMoxHTVELR
NNIPFQMoxHTVELRK
VEAAVGKDLFR
VLELYLDLLSQPCcamR
VLELYLDLLSQPCcamRAIYIFAK
VPAMoxMoxDGGFTLCcamESVAILLYLAHK
YKVPDHWYPQDLQAR	
176	MHC class I heavy chain [Bos taurus]	P01898 	37	5,1	74	31	8	AKGNEQSFHVSLR
APWMEQEGPEYWER
AYLEAECcamVEWLLR
FIIVGYVDDTQFVR
GNEQSFHVSLR
TDPPKTHVTHHPGSEGDVTLR
YFETSVSRPGLGEPR
YLELGKETLLR	
177	Haao protein; (3-hydroxyanthranilate 3,4-dioxygenase) [Mus musculus]	Q78JT3 	32,8	6	420	96	35	AQGSVALSVTQDPAR
ASFQPPVCcamNK
ASFQPPVCcamNKLMoxHQEQLK
DLGTQLAPIIQEFFHSEQYR
DLGTQLAPIIQEFFHSEQYRTGKPNPDQLLK
DVPIRQGEIFLLPAR
DYHIEEGEEVFYQLEGDMoxILR
ELPFPLNTR
ELQAGTSLSLFGDSYETQVIAHGQGSSK
ELQAGTSLSLFGDSYETQVIAHGQGSSKGPR
FANTMGLVIER
FANTMGLVIERR
FANTMoxGLVIER
FANTMoxGLVIERR
IMFVGGPNTR
IMFVGGPNTRK
IMoxFVGGPNTR
IMoxFVGGPNTRK
KDYHIEEGEEVFYQLEGDMoxILR
LMoxHQEQLKIMoxFVGGPNTR
oxLMHQEQLKIMFVGGPNTR
oxSIMKPMSLKAWLDGHSR
QDVDVWLWQQEGSSK
QGEIFLLPAR
QGEIFLLPARVPHSPQR
RLESELDGLR
SIMoxKPMoxSLKAWLDGHSR
SWVEENRASFQPPVCcamNK
TGKPNPDQLLK
TGKPNPDQLLKELPFPLNTR
VKSWVEENR
VLEQGQHRDVPIR
VTMoxGGQCcamIALAPDDSLLVPAGTSYVWER
YYVGDTEDVLFEK
YYVGDTEDVLFEKWFHCcamK	
178	2-hydroxyphytanoyl-CoA lyase [Mus musculus]	Q9QXE0 	63,6	5,9	261	61	33	ALQSADVIVLFGAR
AQDFHWLTR
DSFIVSEGANTMDIGR
DSFIVSEGANTMoxDIGR
FIQIDICcamAEELGNNVRPSVILLGDIDAVSK
FSARPSTIELIPFIIEK
FSARPSTIELIPFIIEKAVR
GAAYSHAEDSIR
GAAYSHAEDSIRK
GVVPDNHPNCcamVGAAR
HRLDAGSFGTMGVGLGFAIAAALVAK
HRLDAGSFGTMoxGVGLGFAIAAALVAK
KAQDFHWLTR
KLVEQCcamSLPFLPTPMGK
LDAGSFGTMoxGVGLGFAIAAALVAK
LNWILHFGLPPR
LNWILHFGLPPRYQADVK
LVEQCcamSLPFLPTPMGK
LVEQCcamSLPFLPTPMoxGK
NQEAMGAFQEFPQVEACcamR
NQEAMoxGAFQEFPQVEACcamR
PESNSAEGSDR
PESNSAEGSDRSEEQVSGAK
QALQDTSKPCcamLLNIMIEPQSTR
QALQDTSKPCcamLLNIMoxIEPQSTR
SLPMNYYTVFYHVQEQLPR
SLPMoxNYYTVFYHVQEQLPR
SRALQSADVIVLFGAR
SSIYGRPGACcamYIDIPADFVTLQANVTSIK
TMLQNCcamLPR
TPEELQHSLR
TQDVEYMFGVVGIPVTEIALAAQELGIK
TQDVEYMoxFGVVGIPVTEIALAAQELGIK
VICcamVEGDSAFGFSGMEVETICcamR
VICcamVEGDSAFGFSGMoxEVETICcamR	
179	Hadhsc protein [Mus musculus]	Q61425 	31,8	7,6	132	76	17	DTPGFIVNRLLVPYLIEAVR
FAGLHFFNPVPMoxMoxK
FTENPKAGDEFVEK
HPVSCcamKDTPGFIVNR
HVTVIGGGLMoxGAGIAQVAAATGHTVVLVDQTEDILAK
LDKFAAEHTIFASNTSSLQITNIANATTR
LGAGYPMoxGPFELLDYVGLDTTK
LKNELFQR
LLVPYLIEAVRLHER
LVEVIKTPMoxTSQK
oxFAGLHFFNPVPMMK
oxQDRFAGLHFFNPVPMMK
QDRFAGLHFFNPVPMMK
QDRFAGLHFFNPVPMoxMoxK
TFESLVDFCcamK
TGEGFYKYK
TLSCcamLSTSTDAASVVHSTDLVVEAIVENLK	
180	Hydroxyacyl-Coenzyme A dehydrogenase/3-ketoacyl-Coenzyme A thiolase/enoyl-Coenzyme A hydratase (trifunctional protein), alpha subunit [Mus musculus]	Q64428 	82,7	9,2	336	64	37	ADMoxVIEAVFEDLGVK
AGLEQGSDAGYLAESQK
AGLEQGSDAGYLAESQKFGELALTK
ALMoxGLYNGQVLCcamK
ALMoxGLYNGQVLCcamKK
ALTSFERDSIFSNLIGQLDYK
CcamLAPMoxMoxSEVMoxR
DSIFSNLIGQLDYK
DSIFSNLIGQLDYKGFEK
EVESVTPEHCcamIFASNTSALPINQIAAVSK
EVQSEFIEVMoxNEIWANDQIR
FGGGSVELLKQMoxVSK
GFYIYQEGSK
GFYIYQEGSKNK
GLVEKLTTYAMoxTVPFVR
GLYPAPLKIIDAVK
KLDALTTGFGFPVGAATLADEVGVDVAQHVAEDLGK
KMoxGLVDQLVEPLGPGIK
KTVLGVPEVLLGILPGAGGTQR
LPAKPEVSSDEDVQYR
LTTYAMoxTVPFVR
LTTYAMTVPFVR
MoxGLVDQLVEPLGPGIKSPEER
MoxVGVPAAFDMoxMoxLTGR
NVQQLAILGAGLMoxGAGIAQVSVDK
oxCcamLAPMMSEVMR
SAVLISSKPGCcamFVAGADINMoxLSSCcamTTPQEATR
SGKGFYIYQEGSK
SLNSEMDNILANLR
SLNSEMoxDNILANLR
SPKPVVAAISGSCcamLGGGLELAIACcamQYR
TIEYLEEVAVNFAK
TLLKDTTVTGLGR
TVLGVPEVLLGILPGAGGTQR
VIGMoxHYFSPVDK
VIIVVKDGPGFYTTR
YESAYGTQFTPCcamQLLLDHANNSSKK	
181	Hydroxyacyl-Coenzyme A dehydrogenase/3-ketoacyl-Coenzyme A thiolase/enoyl-Coenzyme A hydratase (trifunctional protein), beta subunit [Mus musculus]	Q99JY0 	51,4	9,5	210	59	25	AALSGLLHRTNIPK
AGLTMoxNDIDAFEFHEAFSGQILANFK
ALAMoxGYKPKAYLR
AMoxDSDWFAQNYMoxGR
AMoxDSDWFAQNYMoxGRK
AQDEGHLSDIVPFKVPGK
AYLRDFIYVSQDPK
DFIYVSQDPKDQLLLGPTYATPK
DGGQYALVAACcamAAGGQGHAMoxIVEAYPK
DNGIRPSSLEQMAK
DNGIRPSSLEQMoxAK
DVVDYIIFGTVIQEVK
DVVDYIIFGTVIQEVKTSNVAR
FNIWGGSLSLGHPFGATGCcamR
IPFLLSGTSYK
IPFLLSGTSYKDLMoxPHDLAR
IPFLLSGTSYKDLMPHDLAR
LAAAFAVSRMoxEQDEYALR
LNFLSPELPAVAEFSTNETMoxGHSADR
MEQDEYALR
MEQDEYALRSHSLAK
MoxEQDEYALR
MoxEQDEYALRSHSLAK
NIVVVEGVRIPFLLSGTSYK
oxAMDSDWFAQNYMGR
oxAMDSDWFAQNYMGRK
TNIPKDVVDYIIFGTVIQEVK
VGSPPLEKFNIWGGSLSLGHPFGATGCcamR	
182	Hal protein [Mus musculus]	P35492 	72,2	5,9	301	53	26	AFDTDIHAVRPHR
AFDTDIHAVRPHRGQIEVAFR
ALDYLAIGVHELAAISER
ALDYLAIGVHELAAISERR
EGLALINGTQMITSLGCcamEALER
EGLALINGTQMoxITSLGCcamEALER
EVIDSIIKER
FCcamDRVQDAYTLR
FMAPDIEAAHR
FMoxAPDIEAAHR
GETISGGNFHGEYPAK
GEWLAVPCcamQDGKLTVGWLGR
GYSGISLETLKQVIEAFNASCcamLSYVPEK
LLLDQKVWEVAAPYIEK
NKPDNGGFTSVDEVQFLVHR
QADIVAALTLEVLK
QADIVAALTLEVLKGTTK
SHSSGVGKPLSPER
SLLDSDHHPSEIAESHR
SVVRPWIKDR
TVIPANKLQELQVNLVR
TVVYGITTGFGK
TVVYGITTGFGKFAR
VVEHVEQVLAIELLAACcamQGIEFLRPLK
YIALDGDSLSTEDLVNLGKGR
YVLEAHGLKPIVLKPK	
183	Alpha-globin	P01942 	15,1	7,8	127	86	10	IGGHGAEYGAEALER
IGGHGAEYGAEALERMoxFASFPTTK
KVADALASAAGHLDDLPGALSALSDLHAHK
LLSHCcamLLVTLASHHPADFTPAVHASLDK
LRVDPVNFK
TYFPHFDVSHGSAQVK
TYFPHFDVSHGSAQVKGHGK
VADALASAAGHLDDLPGALSALSDLHAHK
VADALASAAGHLDDLPGALSALSDLHAHKLR
VLSGEDKSNIK	
184	Heme-binding protein Mus musculus	Q9R257 	21,1	5	165	86	15	EADYVAHATQLR
EGITVYSTQFGGYAK
EGITVYSTQFGGYAKEADYVAHATQLR
FATVEVTDKPVDEALR
FATVEVTDKPVDEALREAMoxPK
FATVEVTDKPVDEALREAMPK
GVGMoxGMoxTVPVSFAVFPNEDGSLQK
GVGMoxGMoxTVPVSFAVFPNEDGSLQKK
IEEREGITVYSTQFGGYAK
IPNQFQGSPPAPSDESVK
IPNQFQGSPPAPSDESVKIEER
NSLFGSVETWPWQVLSTGGKEDVSYEER
RNEVWLVK
TTLEGTPATYQGDVYYCcamAGYDPPMoxKPYGR
VWFRIPNQFQGSPPAPSDESVK	
185	Hepatoma-derived growth factor [Mus musculus]	P51859 	26,3	4,8	117	46	10	AGDVLEDSPKRPK
DLFPYEESKEK
ESGDHEEEDKEIAALEGERPLPVEVEK
GFSEGLWEIENNPTVK
IDEMPEAAVKSTANK
KGFSEGLWEIENNPTVK
MKGYPHWPAR
MoxKGYPHWPAR
STANKYQVFFFGTHETAFLGPK
YQVFFFGTHETAFLGPK	
186	Haloacid dehalogenase-like hydrolase domain containing 3 [Mus musculus]	Q9CYW4 	28	6,2	226	61	17	AHGVVVEDITVEQAFR
AQSHNFPNYGLSR
ARAHGVVVEDITVEQAFR
DSVPKEHILPSLSHLLPALDLLEASSPMoxS
DVVLHTFR
EHFDFVLTSEAVGCcamPKPDPR
EHILPSLSHLLPALDLLEASSPMoxS
GLTSRQWWK
IFREALQR
LAVVSNFDR
LAVVSNFDRR
LEDILTGLGLR
LLTWDVKDTLIK
LRRPVGEEYASK
QWWKDVVLHTFR
RLEDILTGLGLR
RPVGEEYASK	
187	3-hydroxyisobutyrate dehydrogenase, mitochondrial precursor; 3-hydroxyisobutyrate dehydrogenase, mitochondrial; EST AI265272 [Mus musculus]	Q99L13 	35,4	8,4	188	57	22	DFSSVFQYLR
DFSSVFQYLREEEPF
DLGLAQDSATSTKTPILLGSLAHQIYR
EAGEQVASSPAEVAEK
EAGEQVASSPAEVAEKADR
EFKEAGEQVASSPAEVAEK
GSLLIDSSTIDPSVSK
GSLLIDSSTIDPSVSKELAK
HGYPLILYDVFPDVCcamK
HGYPLILYDVFPDVCcamKEFK
ICcamNNMoxLLAISMoxIGTAEAMoxNLGIR
IITMoxLPSSMoxNAVEVYSGANGILK
IITMoxLPSSMoxNAVEVYSGANGILKK
KDFSSVFQYLR
MGAVFMDAPVSGGVGAAR
MoxGAVFMoxDAPVSGGVGAAR
oxICcamNNMLLAISMIGTAEAMNLGIR
oxIITMLPSSMNAVEVYSGANGILKK
oxMGAVFMDAPVSGGVGAAR
oxMMCcamSKGYSK
oxTPVGFIGLGNMGNPMAK
TPILLGSLAHQIYR
TPVGFIGLGNMoxGNPMoxAK	
188	Histidine triad nucleotide binding protein 1; histidine triad nucleotide-binding protein; protein kinase C inhibitor 1; PKC inhibitor/ interacting protein Mus musculus	P70349 	13,8	6,2	115	79	9	AQVAQPGGDTIFGK
CcamAADLGLKR
CcamLAFHDISPQAPTHFLVIPK
EIPAKIIFEDDR
HISQISVADDDDESLLGHLMoxIVGKK
IIFEDDR
IIFEDDRCcamLAFHDISPQAPTHFLVIPK
MoxVVNEGADGGQSVYHIHLHVLGGR
MVVNEGADGGQSVYHIHLHVLGGR	
189	Hint2 protein [Mus musculus]	Q9D0S9 	17,3	9,8	117	63	8	AAPGGASPTIFSR
DVAPQAPVHFLVIPR
IAQAQGLKDGYR
ISQAEEDDQQLLGHLLLVAK
ISQAEEDDQQLLGHLLLVAKK
LVVNDGKMoxGAQSVYHLHIHVLGGR
MGAQSVYHLHIHVLGGR
MoxGAQSVYHLHIHVLGGR
SLPADILYEDQQCcamLVFR	
190	HSBO22 histone H2B - bovine	Q2KII5 	13,8	10,4	114	70	11	AMoxGIMoxNSFVNDIFER
AMoxGIMoxNSFVNDIFERIAGEASR
EIQTAVRLLLPGELAK
ESYSVYVYK
KESYSVYVYK
LLLPGELAKHAVSEGTK
oxAMGIMNSFVNDIFER
PEPAKSAPAPK
QVHPDTGISSK
STITSREIQTAVR
VLKQVHPDTGISSK	
191	MHC class I antigen 2 [Homo sapiens]	Q700Z7 	20,8	5,6	70	52	8	AYLEGTCcamVEWLRR
FISVGYVDDTQFVR
GYYNQSEDGSHTLQR
KLEAAHAAEQLR
LEAAHAAEQLR
MoxSGCcamDLGPDGR
SHSMoxRYFDTAVSRPGR
SWTAADTAAQITQRK	
192	High mobility group protein B1;(Dna-Binding Hmg-Box Domain B Of Rat Hmg1) (Nmr, 30 Structures)	P63159 	8,8	9,6	75	77	6	IKGEHPGLSIGDVAK
KLGEMWNNTAADDK
LGEMoxWNNTAADDKQPYEK
LGEMWNNTAADDK
RPPSAFFLFCcamSEYRPK
YEKDIAAYR	
193	Hmgcl protein [Mus musculus]	P38060 	34,2	8,7	156	67	20	EVSVFGAVSELFTR
EVSVFGAVSELFTRK
FAGVMoxQAAQAASISVR
FPGINYPVLTPNMoxK
GASGNLATEDLVYMLNGLGIHTGVNLQK
GASGNLATEDLVYMoxLNGLGIHTGVNLQK
GFEEAVAAGAKEVSVFGAVSELFTR
GIQKFPGINYPVLTPNMK
GIQKFPGINYPVLTPNMoxK
GYVSCcamALGCcamPYEGK
GYVSCcamALGCcamPYEGKVSPAK
IRLIDMoxLSEAGLPVIEATSFVSPK
IVEVGPRDGLQNEK
LIDMLSEAGLPVIEATSFVSPK
LIDMoxLSEAGLPVIEATSFVSPK
LLEAGDFICcamQALNRK
NANCcamSIEESFQR
oxKLYSMGCcamYEISLGDTIGVGTPGLMK
SIVPTPVKIR
WVPQMADHSDVLK	
194	3-hydroxy-3-methylglutaryl-Coenzyme A synthase ; Hmgcs1 protein;  [Mus musculus]	Q8JZK9 	57,6	5,6	191	50	25	AFMKASSELFNQK
AFMoxKASSELFNQK
ASSELFNQKTK
GTHMoxQHAYDFYKPDMoxLSEYPVVDGK
HSLSYDCcamIGR
HSLSYDCcamIGRLEVGTETIIDK
LEDTYFDRDVEK
LPATSAESESAVISNGEH
LSIQCcamYLSALDR
LSIQCcamYLSALDRCcamYSVYR
MFLNDFLNDQNR
MFLNDFLNDQNRDK
MGFCcamTDREDINSLCcamLTVVQK
MoxFLNDFLNDQNR
MoxFLNDFLNDQNRDK
MoxGFCcamTDREDINSLCcamLTVVQK
PGSLPLNAEACcamWPK
RPFTNDHSLDEGMoxGLVHSNTATEHIPSPAK
RVGVFSYGSGLAATLYSLK
TCcamVAPDVFAENMK
TCcamVAPDVFAENMoxK
TCcamVAPDVFAENMoxKLR
VGVFSYGSGLAATLYSLK
VTQDATPGSALDKITASLCcamDLK
YDGVDAGKYTIGLGQAR	
195	3-hydroxy-3-methylglutaryl CoA synthase	P54869 	56,9	8,5	316	62	31	APLVLEQGLR
ASLDMoxFNQK
CcamYAAYR
DASPGSTLEK
DVGILALEVYFPAQYVDQTDLEK
EQFYHK
FNNVEAGK
FNNVEAGKYTVGLGQTR
GTHMENAYDFYKPNLASEYPLVDGK
IGAFSYGSGLAASFFSFR
LEVGTETIIDK
LMFNDFLSSSSDKQNNLYK
LMoxFNDFLSSSSDKQNNLYK
LSIQCcamYLR
LVSSVSDLPK
LVSSVSDLPKR
MGFCcamSVQEDINSLCcamLTVVQR
MoxGFCcamSVQEDINSLCcamLTVVQR
MoxSPEEFTEIMoxNQR
MQRLLAPAR
MSPEEFTEIMNQR
oxMSPEEFTEIMNQR
QAGNNQPFTLDDVQYMoxIFHTPFCcamK
RMoxSPEEFTEIMoxNQR
TKLPWDAVGR
VDEMoxHR
VDEMoxHRR
VNFSPPGDTSNLFPGTWYLER
VSKDASPGSTLEK
YALVVCcamGDIAVYPSGNARPTGGAGAVAMoxLIGPK
YTVGLGQTR	
196	Heterogeneous nuclear ribonucleoprotein A2/B1 isoform 1 [Mus musculus]	O88569 	36	8,7	115	59	14	ALSRQEMoxQEVQSSR
AVAREESGKPGAHVTVK
GFGDGYNGYGGGPGGGNFGGSPGYGGGR
GFGFVTFDDHDPVDKIVLQK
GFGFVTFSSMoxAEVDAAMoxAARPHSIDGR
GGGGNFGPGPGSNFR
GGGGNFGPGPGSNFRGGSDGYGSGR
IDTIEIITDR
NMGGPYGGGNYGPGGSGGSGGYGGR
NMoxGGPYGGGNYGPGGSGGSGGYGGR
NYYEQWGK
SGRGGNFGFGDSR
YHTINGHNAEVR
YHTINGHNAEVRK	
197	Heterogeneous nuclear ribonucleoprotein A/B [Mus musculus]	Q99020 	30,8	7,5	157	41	14	DYFTKFGEVVDCcamTIK
EVYQQQQYGSGGR
EVYQQQQYGSGGRGNR
FGEVVDCcamTIK
FGEVVDCcamTIKMDPNTGR
FGEVVDCcamTIKMoxDPNTGR
FHTVSGSKCcamEIK
GFGFILFKDSSSVEK
GGHQNNYKPY
IFVGGLNPEATEEKIR
IREYFGQFGEIEAIELPIDPK
RGGHQNNYKPY
SRGFGFILFK
VAQPKEVYQQQQYGSGGR	
198	Heterogeneous nuclear ribonucleoprotein F [Mus musculus]	Q9Z2X1 	45,7	5,2	184	56	21	ATENDIYNFFSPLNPVR
DLSYCcamLSGMoxYDHR
DLSYCcamLSGMYDHR
DRANMoxQHR
FMoxSVQRPGPYDRPGTAR
FMSVQRPGPYDRPGTAR
GLPYKATENDIYNFFSPLNPVR
HSGPNSADSANDGFVR
ITGEAFVQFASQELAEK
QSGEAFVELESEDDVK
QSGEAFVELESEDDVKLALK
SGAYSAGYGGYEEYSGLSDGYGFTTDLFGR
SHRTEMoxDWVLK
SYSDPPLKFMoxSVQRPGPYDRPGTAR
VHIEIGPDGR
VTGEADVEFATHEEAVAAMoxSKDR
VTGEADVEFATHEEAVAAMSK
VTGEADVEFATHEEAVAAMSKDR
YGDSEFTVQSTTGHCcamVHMoxR
YIEVFKSHR
YIEVFKSSQEEVR	
199	HNRPH1 [Homo sapiens]	P31943 	49,1	5,7	159	61	21	ATENDIYNFFSPLNPVR
DLNYCcamFSGMoxSDHR
DLNYCcamFSGMSDHR
EGRPSGEAFVELESEDEVK
EGRPSGEAFVELESEDEVKLALK
FFSDCcamKIQNGAQGIR
GAYGGGYGGYDDYNGYNDGYGFGSDR
GLPFGCcamSKEEIVQFFSGLEIVPNGITLPVDFQGR
HTGPNSPDTANDGFVR
SNNVEMoxDWVLKHTGPNSPDTANDGFVR
STGEAFVQFASQEIAEK
VHIEIGPDGR
VHIEIGPDGRVTGEADVEFATHEDAVAAMoxSK
VRGLPWSCcamSADEVQR
VTGEADVEFATHEDAVAAMoxSK
VTGEADVEFATHEDAVAAMoxSKDK
VTGEADVEFATHEDAVAAMSK
YGDGGSTFQSTTGHCcamVHMoxR
YGDGGSTFQSTTGHCcamVHMR
YIEIFKSSGAEVR
YVELFLNSTAGASGGAYEHR	
200	Heterogeneous nuclear ribonucleoprotein H2; heterogeneous nuclear ribonucleoprotein H' [Mus musculus]	P70333 	49,3	5,8	154	51	22	ATENDIYNFFSPLNPMoxR
ATENDIYNFFSPLNPMR
DKANMoxQHR
DKANMQHR
DLNYCcamFSGMoxSDHR
DLNYCcamFSGMSDHR
DRETMGHR
DRETMoxGHR
EGRPSGEAFVELESEDEVK
FFSDCcamKIQNGTSGVR
GAYGGGYGGYDDYGGYNDGYGFGSDR
GLPWSCcamSAEEVMoxR
GLPWSCcamSAEEVMR
HTGPNSPDTANDGFVR
LRGLPFGCcamSK
MoxMoxLSTEGR
STGEAFVQFASQEIAEK
THYDPPR
THYDPPRK
VHIEIGPDGR
VTGEADVEFATHEDAVAAMoxAK
YGDGGSSFQSTTGHCcamVHMoxR
YGDGGSSFQSTTGHCcamVHMR	
201	Heterogeneous nuclear ribonucleoprotein K [Mus musculus]	P61979 	51	5,3	159	45	20	ALRTDYNASVSVPDSSGPER
ARNLPLPPPPPPR
DYDDMoxSPR
GSDFDCcamELRLLIHQSLAGGIIGVK
GSYGDLGGPIITTQVTIPK
GSYGDLGGPIITTQVTIPKDLAGSIIGK
IILDLISESPIK
IILDLISESPIKGR
IITITGTQDQIQNAQYLLQNSVK
ILSISADIETIGEILK
ILSISADIETIGEILKK
LFQECcamCcamPHSTDR
LLIHQSLAGGIIGVK
RGRPVGFPMR
RPAEDMoxEEEQAFKR
SRNTDEMoxVELR
SRNTDEMVELR
TDYNASVSVPDSSGPER
VVECcamIKIILDLISESPIK
VVLIGGKPDR	
202	4-hydroxyphenylpyruvate dioxygenase [Mus musculus]	P49429 	45,1	6,6	147	49	13	ERGTEFLAAPSSYYK
FAVLQTYGDTTHTLVEK
FAVLQTYGDTTHTLVEKINYTGR
FLHFHSVTFWVGNAK
FLPGFEAPTYKDTLLPK
FWSVDDTQVHTEYSSLR
GRFLHFHSVTFWVGNAK
GTEFLAAPSSYYK
HGDGVKDIAFEVEDCcamDHIVQK
IVFVLCcamSALNPWNKEMoxGDHLVK
MoxGFEPLAYR
SIVVTNYEESIKMoxPINEPAPGR
SQIQEYVDYNGGAGVQHIALKTEDIITAIR	
203	Hemopexin [Mus musculus]	Q91X72 	51,3	9	231	48	19	DYFVSCcamPGR
DYFVSCcamPGRGHGRPR
ELGSPPGISLETIDAAFSCcamPGSSR
FNPVTGEVPPR
FNPVTGEVPPRYPLDAR
GATYAFTGSHYWR
GECcamQSEGVLFFQGNR
GECcamQSEGVLFFQGNRK
GGNNLVSGYPK
KWFWDFATR
LFQEEFPGIPYPPDAAVECcamHR
SGAQATWTEVSWPHEK
SGAQATWTEVSWPHEKVDGALCcamLDK
SLGPNTCcamSSNGSSLYFIHGPNLYCcamYSSIDK
SLPQPQKVNSILGCcamSQ
WFWDFATR
WKNPITSVDAAFR
YYCcamFQGNK
YYCcamFQGNKFLR	
204	HS9B_HORSE Heat shock protein HSP 90-beta (HSP 84)	P11499 	82	5	358	59	47	ADHGEPIGR
AEADKNDK
ALLFIPR
ALLFIPRR
AQALRDNSTMoxGYMoxMoxAK
CcamLELFSELAEDK
DLVVLLFETALLSSGFSLEDPQTHSNR
ELISNASDALDKIR
ELKIDIIPNPQER
EQVANSAFVER
FYEAFSK
GFEVVYMoxTEPIDEYCcamVQQLK
GVVDSEDLPLNISR
HFSVEGQLEFR
HLEINPDHPIVETLR
HNDDEQYAWESSAGGSFTVR
HSQFIGYPITLYLEK
HSQFIGYPITLYLEKER
IDIIPNPQER
KHLEINPDHPIVETLR
KHSQFIGYPITLYLEK
LGIHEDSTNR
LGIHEDSTNRR
LVSSPCcamCcamIVTSTYGWTANMER
LVSSPCcamCcamIVTSTYGWTANMoxER
NLKLGIHEDSTNR
NNIKLYVR
NPDDITQEEYGEFYK
oxDNSTMGYMMAK
oxDNSTMGYMMAKK
RAPFDLFENK
RLSELLR
RVFIMDSCcamDELIPEYLNFIR
RVFIMoxDSCcamDELIPEYLNFIR
SIYYITGESKEQVANSAFVER
SLTNDWEDHLAVK
SLTNDWEDHLAVKHFSVEGQLEFR
TKPIWTR
TKPIWTRNPDDITQEEYGEFYK
TLTLVDTGIGMoxTKADLINNLGTIAK
TLTLVDTGIGMTKADLINNLGTIAK
VFIMDSCcamDELIPEYLNFIR
VILHLKEDQTEYLEER
VVVITKHNDDEQYAWESSAGGSFTVR
YHTSQSGDEMTSLSEYVSR
YIDQEELNK
YIDQEELNKTKPIWTR	
205	Protein kinase [Sus scrofa]	P14625 	92,9	4,7	202	48	32	ALWVLGLCcamCcamVLLTFGSVR
AQAYQTGKDISTNYYASQK
EFEPLLNWMKDK
EFEPLLNWMoxK
ELISNASDALDKIR
ESDDPMoxAYIHFTAEGEVTFK
FAFQAEVNR
FQNVAKEGVK
GVVDSDDLPLNVSRETLQQHK
GYEVIYLTEPVDEYCcamIQALPEFDGKR
HPLIRDMLR
HPLIRDMoxLR
KEAESSPFVER
LGVIEDHSNR
LGVIEDHSNRTR
LISLTDENALAGNEELTVK
LTESPCcamALVASQYGWSGNMER
MLRLSLNIDPDAK
MMKLIINSLYK
NLLHVTDTGVGMoxTR
NLLHVTDTGVGMoxTREELVK
NLLHVTDTGVGMTREELVK
oxRVFITDDFHDMMPK
oxVFITDDFHDMMPK
QDKIYFMAGSSR
RVFITDDFHDMoxMoxPK
SEKFAFQAEVNR
SILFVPTSAPR
TDDEVVQREEEAIQLDGLNASQIR
TETVEEPMEEEEAAKEEK
TETVEEPMoxEEEEAAKEEK
VEKTVWELGLMNDIKPIWQRPSK
VFITDDFHDMoxMoxPK
YLNFVKGVVDSDDLPLNVSR
YSQFINFPIYVWSSK	
206	Heat shock protein gp96 precursor; Endoplasmin [Precursor] [Homo sapiens]	Q29092 	90,2	4,7	103	26	23	DDEVDVDGTVEEDLGKSR
EAESSPFVER
EFEPLLNWMKDK
EFEPLLNWMoxK
ELISNASDALDKIR
FAFQAEVNR
GVVDSDDLPLNVSR
HPLIRDMoxLR
KEAESSPFVER
KYSQFINFPIYVWSSK
LGVIEDHSNR
LGVIEDHSNRTR
LTESPCcamALVASQYGWSGNMER
LTESPCcamALVASQYGWSGNMoxER
NLLHVTDTGVGMoxTR
NLLHVTDTGVGMoxTREELVK
NLLHVTDTGVGMTR
NLLHVTDTGVGMTREELVK
SEKFAFQAEVNR
SILFVPTSAPR
TETVEEPMoxEEEEAAKEEK
TFEINPRHPLIR
YLNFVKGVVDSDDLPLNVSR	
207	Heat shock protein 4; heat shock protein, 110 kDa; heat shock 70 kDa protein 4 [Mus musculus]	Q61316 	94,1	5,1	278	42	33	AFSDPFVEAEK
AFSDPFVEAEKSNLAYDIVQLPTGLTGIK
AGGIETIANEYSDR
ALLRLSQECcamEK
CcamTPACcamVSFGPK
EFSITDVVPYPISLR
ELSTTLNADEAVTR
FDEVLVNHFCcamEEFGKK
FLEMoxCcamDDLLAR
FQESEERPK
FVSEDDRNTFTLK
GCcamALQCcamAILSPAFK
GKFLEMCcamDDLLAR
GKFLEMoxCcamDDLLAR
IKELTSICcamSPIISKPKPK
KPVVDCcamVVSVPSFYTDAERR
LKVLATAFDTTLGGR
LMoxNETTAVALAYGIYKQDLPALEEKPR
LSQECcamEKLK
NAVEEYVYEMoxR
NAVEEYVYEMR
NKEDQYEHLDAADVTK
QDLPALEEKPR
QIQQYMKVISSFK
SNLAYDIVQLPTGLTGIK
SVMDATQIAGLNCcamLR
SVMoxDATQIAGLNCcamLR
VEPPLRSVLEQSK
VLATAFDTTLGGR
VREFSITDVVPYPISLR
VTYMoxEEERNFTTEQVTAMoxLLSK
WNSPAEEGLSDCcamEVFPK	
208	78 kDa glucose-regulated protein [Precursor]; A27414 dnaK-type molecular chaperone GRP78 precursor - Chinese hamster	P07823 	72,4	5	295	56	36	AKFEELNMDLFR
AKFEELNMoxDLFR
DAGTIAGLNVMoxR
DNHLLGTFDLTGIPPAPR
EFFNGKEPSR
ELEEIVQPIISK
ETMEKAVEEK
FEELNMoxDLFR
FLPFKVVEK
FPMVAAALLLLCcamAVR
GVPQIEVTFEIDVNGILR
IDTRNELESYAYSLK
IEIESFFEGEDFSETLTR
IINEPTAAAIAYGLDKR
ITITNDQNR
ITITNDQNRLTPEEIER
ITPSYVAFTPEGER
ITPSYVAFTPEGERLIGDAAK
KSQIFSTASDNQPTVTIK
KTKPYIQVDIGGGQTK
KVTHAVVTVPAYFNDAQR
NGRVEIIANDQGNR
NQLTSNPENTVFDAK
oxMKFPMVAAALLLLCcamAVR
QATKDAGTIAGLNVMoxR
RALSSQHQAR
SDIDEIVLVGGSTR
SQIFSTASDNQPTVTIK
STMoxKPVQKVLEDSDLK
TFAPEEISAMoxVLTK
TKPYIQVDIGGGQTK
TWNDPSVQQDIK
VEIIANDQGNR
VTHAVVTVPAYFNDAQR
VYEGERPLTK
VYEGERPLTKDNHLLGTFDLTGIPPAPR	
209	heat shock 70kD protein 5 (glucose-regulated protein); glucose regulated protein, 78 kDa; heat shock 70kD protein 5 (glucose-regulated protein, 78kD) [Mus musculus]	P20029 	72,4	5	298	61	38	AKFEELNMDLFR
AKFEELNMoxDLFR
DAGTIAGLNVMR
DNHLLGTFDLTGIPPAPR
EFFNGKEPSR
ELEEIVQPIISK
ETMEKAVEEK
FEELNMoxDLFR
FLPFKVVEK
GVPQIEVTFEIDVNGIIR
IDTRNELESYAYSLK
IEIESFFEGEDFSETLTR
IEWLESHQDADIEDFKAK
IINEPTAAAIAYGLDKR
ITITNDQNR
ITPSYVAFTPEGER
ITPSYVAFTPEGERLIGDAAK
KSQIFSTASDNQPTVTIK
KTKPYIQVDIGGGQTK
KVTHAVVTVPAYFNDAQR
LTPEEIER
LYGSGGPPPTGEEDTSEKDEL
NGRVEIIANDQGNR
NQLTSNPENTVFDAK
NQLTSNPENTVFDAKR
oxMMKFTVVAAALLLLGAVR
QATKDAGTIAGLNVMoxR
RALSSQHQAR
SDIDEIVLVGGSTR
SQIFSTASDNQPTVTIK
TFAPEEISAMoxVLTK
TFAPEEISAMVLTK
TKPYIQVDIGGGQTK
TWNDPSVQQDIK
VEIIANDQGNR
VMoxEHFIK
VTHAVVTVPAYFNDAQR
VYEGERPLTK	
210	Heat shock 70kDa protein 8 isoform 1; heat shock cognate protein, 71-kDa; heat shock 70kd protein 10; heat shock cognate protein 54; constitutive heat shock protein 70; lipopolysaccharide-associated protein 1; LPS-associated protein 1; heat shock 70kD protein 8 [Homo sapiens]	P63017 	70,9	5,3	234	51	33	ARFEELNADLFR
DAGTIAGLNVLR
DNNLLGKFELTGIPPAPR
ELEKVCcamNPIITK
FEELNADLFR
FELTGIPPAPR
GPAVGIDLGTTYSCcamVGVFQHGKVEIIANDQGNR
HWPFMoxVVNDAGRPK
HWPFMVVNDAGRPK
IINEPTAAAIAYGLDK
IINEPTAAAIAYGLDKK
LLQDFFNGK
LLQDFFNGKELNK
MoxKEIAEAYLGK
MoxVNHFIAEFK
MoxVNHFIAEFKR
MVNHFIAEFK
MVNHFIAEFKR
NQVAMNPTNTVFDAKR
NQVAMoxNPTNTVFDAK
NQVAMoxNPTNTVFDAKR
NSLESYAFNMoxK
NTTIPTKQTQTFTTYSDNQPGVLIQVYEGER
QATKDAGTIAGLNVLR
QTQTFTTYSDNQPGVLIQVYEGER
RFDDAVVQSDMK
SFYPEEVSSMVLTK
STAGDTHLGGEDFDNRMoxVNHFIAEFK
TLSSSTQASIEIDSLYEGIDFYTSITR
TTPSYVAFTDTER
TTPSYVAFTDTERLIGDAAK
TVTNAVVTVPAYFNDSQR
VEIIANDQGNR
VQVEYKGETK	
211	GR75_CRIGR Stress-70 protein, mitochondrial precursor (75 kDa glucose regulated protein) (GRP 75)	O35501 	73,7	5,8	268	50	33	AKCcamELSSSVQTDINLPYLTMoxDASGPK
AMoxQDAEVSKSDIGEVILVGGMoxTR
AQFEGIVTDLIKR
ASNGDAWVEAHGK
CcamELSSSVQTDINLPYLTMoxDASGPK
EMAGDNKLLGQFTLIGIPPAPR
EMoxAGDNKLLGQFTLIGIPPAPR
EQQIVIQSSGGLSKDDIENMoxVK
ETAENYLGHTAK
ETGVDLTKDNMALQR
ETGVDLTKDNMoxALQR
GAVVGIDLGTTNSCcamVAVMoxEGK
GVPQIEVTFDIDANGIVHVSAK
GVPQIEVTFDLDANGIVHVSAKDK
HQDGWNGLSHEAFR
LLGQFTLIGIPPAPR
LYSPSQIGAFVLMK
LYSPSQIGAFVLMoxK
MoxKETAENYLGHTAK
MoxPKVQQTVQDLFGR
NAVITVPAYFNDSQR
oxAMQDAEVSKSDIGEVILVGGMTR
QATKDAGQISGLNVLR
QAVTNPNNTFYATK
QAVTNPNNTFYATKR
SDIGEVILVGGMoxTR
SQVFSTAADGQTQVEIK
SQVFSTAADGQTQVEIKVCcamQGER
STNGDTFLGGEDFDQALLR
TTPSVVAFTADGER
VCcamQGEREMAGDNK
VQQTVQDLFGR
VQQTVQDLFGRAPSK	
212	Heat shock protein, A (Unnamed protein product (mortalin mot-2=hsp70 homolog perinuclear form))	Q7TSZ0 	73,5	5,7	316	55	34	AKCcamELSSSVQTDINLPYLTMoxDASGPK
ALLAGKDSETGENIR
AMoxQDAEVSKSDIGEVILVGGMoxTR
AQFEGIVTDLIKR
ASNGDAWVEAHGK
CcamELSSSVQTDINLPYLTMoxDASGPK
DAGQISGLNVLR
EMoxAGDNKLLGQFTLIGIPPAPR
EQQIVIQSSGGLSKDDIENMoxVK
EQQIVIQSSGGLSKDDIENMVK
ETAENYLGHTAK
ETGVDLTKDNMALQR
ETGVDLTKDNMoxALQR
GAVVGIDLGTTNSCcamVAVMoxEGK
GVPQIEVTFDIDANGIVHVSAK
GVPQIEVTFDIDANGIVHVSAKDK
LLGQFTLIGIPPAPR
LYSPSQIGAFVLMK
LYSPSQIGAFVLMoxK
MoxKETAENYLGHTAK
MoxPKVQQTVQDLFGR
NAVITVPAYFNDSQR
oxAMQDAEVSKSDIGEVILVGGMTR
QAASSLQQASLK
QATKDAGQISGLNVLR
QAVTNPNNTFYATK
QAVTNPNNTFYATKR
SQVFSTAADGQTQVEIK
STNGDTFLGGEDFDQALLR
TTPSVVAFTADGER
VCcamQGEREMAGDNK
VIAVYDLGGGTFDISILEIQK
VQQTVQDLFGR
VQQTVQDLFGRAPSK	
213	Heat shock protein 1; heat shock protein, 25 kDa; heat shock 27kDa protein 1 [Mus musculus]	P14602 	22,9	6,5	101	52	8	AQIGGPEAGKSEQSGAK
AVTQSAEITIPVTFEAR
KYTLPPGVDPTLVSSSLSPEGTLTVEAPLPK
LFDQAFGVPR
TPSWGPFRDWYPAHSR
VSLDVNHFAPEELTVK
VSLDVNHFAPEELTVKTK
YTLPPGVDPTLVSSSLSPEGTLTVEAPLPK	
214	Heat shock protein 1 (chaperonin) [Mus musculus]	P63038 	61	5,8	149	50	20	AAVEEGIVLGGGCcamALLR
ALMoxLQGVDLLADAVAVTMoxGPK
CcamEFQDAYVLLSEK
CcamEFQDAYVLLSEKK
CcamIPALDSLKPANEDQK
DIGNIISDAMoxKK
GRTVIIEQSWGSPK
GVMoxLAVDAVIAELKK
GYISPYFINTSK
ILQSSSEVGYDAMoxLGDFVNMoxVEK
IQEITEQLDITTSEYEKEK
ISSVQSIVPALEIANAHR
KISSVQSIVPALEIANAHR
KPLVIIAEDVDGEALSTLVLNR
LPTVLRQMRPVSR
LVQDVANNTNEEAGDGTTTATVLAR
NAGVEGSLIVEK
TLNDELEIIEGMoxKFDR
TVIIEQSWGSPK
VGEVIVTKDDAMoxLLK	
215	HRA2_MOUSE Serine protease HTRA2, mitochondrial precursor (High temperature requirement protein A2) (HtrA2) (Omi stress-regulated endoprotease) (Serine proteinase OMI)	Q9JIY5 	49,3	9,6	140	34	12	AGLRPGDVILAIGEK
EPLPTLPLGR
IQTKEPLPTLPLGR
KNSWFGTSGSQR
LAQNAEDVYEAVR
NSWFGTSGSQR
NSWFGTSGSQRR
RGSETLTLYVTPEVTE
SQYNFIADVVEK
TAPAVVYIEILDRHPFSGR
VTAGISFAIPSDR
VTAGISFAIPSDRLR	
216	170 kDa glucose regulated protein (OXRP_CRIGR 150 kDa oxygen-regulated protein precursor) (Orp150) (Hypoxia up-regulated 1)	Q60432 	111,3	5,1	131	28	25	AKFTKPRPRPK
ATLRYFQHLLGK
DAVIYPILVEFTR
EAGMoxQPQLQIR
EAGMQPQLQIR
EETEAPVEDTSQPPPPEPKGDAAPEGEKPDEK
FTKPRPRPK
GDAAPEGEKPDEKESGGK
KLCcamQGLFFR
LCcamQGLFFRVEER
MoxAGLKVLQLINDNTATALSYGVFR
MoxMoxALDREVQYLLNK
MoxVEEIGVELAVLDLPDLPEDELAR
oxMMALDREVQYLLNK
QADNPHVALYR
SLAEDFAEQPIKDAVITVPAFFNQAER
SRFPEHELIVDPQR
TKEAGMoxQPQLQIR
TKEAGMQPQLQIR
TLGGLEMELR
TLGGLEMoxELR
TVLSANADHMoxAQIEGLMoxDDVDFKAK
VKPFVVRDAVIYPILVEFTR
VLQLINDNTATALSYGVFR
YSHDFNFHINYGDLGFLGPEDLR	
217	Hypothetical protein 4833421E05Rik [Mus musculus]	Q9DB29 	28	5,2	158	71	13	AASGSALLWPR
DCcamGTDVLDLWTLMoxQK
DCcamGTDVLDLWTLMQK
DSQDFSSYLSDGLHLSPMoxGNEFLFLNLCcamPLLDKK
DVEEAKPELSLLGDGDY
GPGMoxENPVAVTIFFGANDSSLKDENPK
LNRLNSVVGEYANACcamLQVAR
LNSVVGEYANACcamLQVAR
QHVPLDEYSANLR
QHVPLDEYSANLRDMoxVQYLR
QHVPLDEYSANLRDMVQYLR
VILITPPPLCcamEAAWEKECcamVLK
VSSLPWLLPYWK	
218	NADP-dependent isocitrate dehydrogenase	O88844 	46,7	6,3	274	80	33	AKLDNNTELSFFAK
ALEDVCcamIETIEAGFMoxTK
ALEDVCcamIETIEAGFMTK
ATDFVVPGPGKVEITYTPK
DIFQEIYDKK
DLAACcamIKGLPNVQR
GQETSTNPIASIFAWSR
GWPLYLSTK
HAYGDQYRATDFVVPGPGK
IIWELIKEK
KIQGGSVVEMoxQGDEMoxTR
LDNNTELSFFAK
LIDDMoxVAQAMoxKSEGGFIWACcamK
LILPYVELDLHSYDLGIENR
LILPYVELDLHSYDLGIENRDATNDQVTK
LVTGWVKPIIIGR
MoxYQKGQETSTNPIASIFAWSR
NILGGTVFREAIICcamK
NYDGDVQSDSVAQGYGSLGMoxMoxTSVLICcamPDGK
oxIQGGSVVEMQGDEMTR
oxKIQGGSVVEMQGDEMTR
oxVTYMVHDFEEGGGVAMGMYNQDK
oxVTYMVHDFEEGGGVAMGMYNQDK
SDYLNTFEFMDKLGENLK
SDYLNTFEFMoxDK
SDYLNTFEFMoxDKLGENLK
SEGGFIWACcamK
SIEDFAHSSFQMALSK
SIEDFAHSSFQMoxALSK
SQFEAQNICcamYEHR
TVEAEAAHGTVTR
VTYMoxVHDFEEGGGVAMoxGMoxYNQDK
YKSQFEAQNICcamYEHR	
219	Isocitrate dehydrogenase 1 (NADP+), soluble [Mus musculus]	O88844 	46,7	6,3	274	80	33	AKLDNNTELSFFAK
ALEDVCcamIETIEAGFMoxTK
ALEDVCcamIETIEAGFMTK
ATDFVVPGPGKVEITYTPK
DIFQEIYDKK
DLAACcamIKGLPNVQR
GQETSTNPIASIFAWSR
GWPLYLSTK
HAYGDQYRATDFVVPGPGK
IIWELIKEK
KIQGGSVVEMoxQGDEMoxTR
LDNNTELSFFAK
LIDDMoxVAQAMoxKSEGGFIWACcamK
LILPYVELDLHSYDLGIENR
LILPYVELDLHSYDLGIENRDATNDQVTK
LVTGWVKPIIIGR
MoxYQKGQETSTNPIASIFAWSR
NILGGTVFREAIICcamK
NYDGDVQSDSVAQGYGSLGMoxMoxTSVLICcamPDGK
oxIQGGSVVEMQGDEMTR
oxKIQGGSVVEMQGDEMTR
oxVTYMVHDFEEGGGVAMGMYNQDK
oxVTYMVHDFEEGGGVAMGMYNQDK
SDYLNTFEFMDKLGENLK
SDYLNTFEFMoxDK
SDYLNTFEFMoxDKLGENLK
SEGGFIWACcamK
SIEDFAHSSFQMALSK
SIEDFAHSSFQMoxALSK
SQFEAQNICcamYEHR
TVEAEAAHGTVTR
VTYMoxVHDFEEGGGVAMoxGMoxYNQDK
YKSQFEAQNICcamYEHR	
220	Idh3a protein [Mus musculus]	Q9D6R2 	31,4	5,6	198	56	18	CcamSDFTEEICcamR
CcamSDFTEEICcamRR
DMoxANPTALLLSAVMoxMoxLR
ENTEGEYSGIEHVIVDGVVQSIK
GPLKTPIAAGHPSMoxNLLLR
HMoxGLFDHAAK
IAEFAFEYAR
IEAACcamFATIKDGK
IPPEAKESMDK
KTFDLYANVRPCcamVSIEGYK
NNHRSNVTAVHK
SNVTAVHKANIMoxR
TFDLYANVRPCcamVSIEGYK
TPIAAGHPSMNLLLR
TPIAAGHPSMNLLLRK
TPIAAGHPSMoxNLLLR
TPIAAGHPSMoxNLLLRK
TPYTDVNIVTIR	
221	Unnamed protein product (mitofilin) [Mus musculus]	Q8CAQ8 	78,7	6,7	225	42	22	EIAGATPHITAAEGR
FVNQLKGESR
GIEQAVQSHAVAEEEAR
GMoxTGKLSTDDLNSLIAHAHR
GVYSEETLR
GVYSEETLRAR
IAGAGLLFVGGGIGGTILYAK
IDQLNRELAQQK
KAHQLWLSVEALK
LHNMIVDLDNVVK
LHNMoxIVDLDNVVK
LHNMoxIVDLDNVVKK
LSEQELEFR
LSTDDLNSLIAHAHR
LSTDDLNSLIAHAHRR
QAAAHTDHLRDVLK
RSQEQMoxDSFTLDINTAYAR
SLEDALNRTSSVTLQTITAQNAAVQAVK
SQEQMDSFTLDINTAYAR
SQEQMoxDSFTLDINTAYAR
TIPYSDKLFGMoxVLGSAPYTVPLPK
TSSAEMoxPTIPLGSAVEAIR
TSSVTLQTITAQNAAVQAVK
VNCcamSDNEFTQALTAAIPPESLTR
VVSQYHELVVQAR
WDSHFRESVEK	
222	Thioether S-methyltransferase [Mus musculus]	P40936 	29,5	5,8	150	64	15	AIQDAGCcamQVLK
CcamNCcamVSLSYSEAYCcamSHDGLCcamFVVAR
DYLTTYYSFHSGPVAEQEIVK
EIIVTDYTPQNLQELQK
EPGAYDWSSIVQHACcamELEGDR
FQHYMoxVGPK
FQHYMoxVGPKK
FQHYMVGPK
FSGVYLEKEVVEK
KEPGAYDWSSIVQHACcamELEGDR
KFSGVYLEK
LAGLLKPGGHLVTLVTLR
LAGLLKPGGHLVTLVTLRFQHYMoxVGPK
RLAGLLKPGGHLVTLVTLR
TPPLGSAQVPLADCcamVLTFLAMoxECcamACcamPDIDTYR	
223	Isovaleryl coenzyme A dehydrogenase; isovaleryl dehydrogenase precursor [Mus musculus]	Q9JHI5 	46,3	8,7	213	60	23	ACcamDEGHIIPK
AQEIDQTNDFK
AQEIDQTNDFKNLR
FWITNGPDADILVVYAK
GITAFIVEKGMoxPGFSTSK
GSNTCcamELVFEDCcamKVPAANVLSQESK
GVYVLMoxSGLDLER
HTISKFLQENLAPK
IGQFQLMQGK
LISGEFIGALAMoxSEPNAGSDVVSMoxK
LRPSPSPLAVPR
LVLAGGPLGIMoxQAVLDHTIPYLHVR
LYEIGAGTSEVR
LYEIGAGTSEVRR
oxLISGEFIGALAMSEPNAGSDVVSMK
QLGSLGVLGITAPVQYGGSGLGYLEHVLVMoxEEISR	
224	Steroid dehydrogenase; Estradiol 17-beta-dehydrogenase 8	P50171 	26,6	5,8	200	55	13	AGVIGLTQTAAR
CcamNSVLPGFIATPMoxTQK
CcamNSVLPGFIATPMoxTQKMoxPEK
GKHAAFQADVSQGPAAR
GSIINISSIIGK
GTFLVTQAAAQALVSSGGR
HAAFQADVSQGPAAR
HAAFQADVSQGPAARR
LAAEGAAVAACcamDLDGAAAQDTVR
LLGSPGSEDGAPR
LRSALALVTGAGSGIGR
SALALVTGAGSGIGR
VGNIGQTNYASSK	
225	Ketohexokinase [Mus musculus]	P97328 	32,7	5,6	196	74	17	CcamAFMGSLAPGHVADFLVADFR
CcamAFMGSLAPGHVADFLVADFRQR
CcamAFMoxGSLAPGHVADFLVADFR
CcamAFMoxGSLAPGHVADFLVADFRQR
FKWIHIEGR
GATLVCcamAWAEEGADALGPDGQLLHSDAFPPPR
GGNASNSCcamTVLSLLGAR
GVDVSQVTWQSQGDTPCcamSCcamCcamIVNNSNGSR
HLGFQSAVEALR
HLGFQSAVEALRGLYSR
IEEHNAKQPLPQK
KCcamGLQGFDGIV
MoxLQRIEEHNAK
TIILYDTNLPDVSAK
TIILYDTNLPDVSAKDFEK
VSVEIEKPREELFQLFSYGEVVFVSK
VVDTLGAGDTFNASVIFSLSK	
226	Kininogen 1; H-kininigen; L-kininogen [Mus musculus]	O08677 	47,9	5,7	217	45	20	ATSQVVAGTKYVIEFIAR
AYFPCcamIGCcamVHAISTDSPDLEPVLK
CcamQALDMoxTEMoxAR
DAEEAATGECcamTATVGK
DIPVDSPELKEVLGHSIAQLNAENDHPFYYK
ENEFFIVTQTCcamK
EVLGHSIAQLNAENDHPFYYK
FPSLHGDCcamVALPNGDDGECcamR
GNLFMoxDINNK
HLGQSLDCcamNANVYMoxRPWENK
RPPGFSPFR
SGNQYMLHR
SGNQYMoxLHR
SVTVQETKEGR
TDGSPTFYSFK
VIEGTKTDGSPTFYSFK	
227	Cytokeratin	P05784 	47,5	5,2	364	81	36	AQIFANSVDNAR
AQMoxEQLNGVLLHLESELAQTR
AQYEALAQK
DAETTLTELRR
DWGHYFK
GPQGVRDWGHYFK
IIEDLRAQIFANSVDNAR
IREHLEK
IVDGRVVSETNDTR
IVLQIDNAR
LAADDFR
LASYLDKVK
LEAEIATYRR
LQLETEIEALKEELLFMoxK
NHEEEVQGLEAQIASSGLTVEVDAPK
NQNINLENSLGDVEAR
QAQEYEALLNIK
QAQEYEALLNIKVK
QSVESDIHGLR
QSVESDIHGLRK
SAEIRDAETTLTELR
SLETENRR
SLGSVRTPSQR
SQDLSKIMADIR
STTFSTNYR
SVWGGSVGSAGLAGMoxGGIQTEK
TLQTLEIDLDSMoxK
TLQTLEIDLDSMoxKNQNINLENSLGDVEAR
VKLEAEIATYR
VKYETELAMoxR
VKYETELAMR
VRPASSAASVYAGAGGSGSRISVSR
VVDDTNITR
VVSETNDTRVLR
YKAQMEQLNGVLLHLESELAQTR
YKAQMoxEQLNGVLLHLESELAQTR	
228	K2C8_MOUSE Keratin, type II cytoskeletal 8 (Cytokeratin 8) (Cytokeratin endo A)	P11679 	54,4	5,5	154	50	26	AEAETMoxYQIKYEELQTLAGK
AQYEDIANR
ASLEAAIADAEQR
ASLEAAIADAEQRGEMoxAIK
DGKLVSESSDVVSK
DVDEAYMNKVELESR
DVDEAYMoxNKVELESR
ELQSQISDTSVVLSMDNSR
FASFIDKVR
FLEQQNKMoxLETK
LALDIEITTYRK
LEGLTDEINFLR
LESGMoxQNMoxSIHTK
LLEGEESRLESGMoxQNMoxSIHTK
NINRLQEIEALK
oxLLEGEESRLESGMQNMSIHTK
oxSNMDNMFESYINNLRR
QIHEEEIRELQSQISDTSVVLSMoxDNSR
QLREYQELMNVK
QLREYQELMoxNVK
SLDMDGIIAEVR
SLDMoxDGIIAEVR
SNMoxDNMoxFESYINNLRR
SRAEAETMoxYQIK
SYKMSTSGPR
TEMoxENEFVLIKK	
229	Cytokeratin 9 [Homo sapiens]	P35527 	62,1	5,1	133	42	21	DIENQYETQITQIEHEVSSSGQEVQSSAK
QGVDADINGLR
FEMoxEQNLR
FSSSGGRGGGGR
FSSSSGYGGGSSR
FSSSSGYGGGSSRVCcamGR
GGGGSFGYSYGGGSGGGFSASSLGGGFGGGSR
GGSGGSHGGGSGFGGESGGSYGGGEEASGSGGGYGGGSGK
GGSGGSYGGGGSGGGYGGGSGSR
HGVQELEIELQSQLSK
HGVQELEIELQSQLSKK
IKFEMEQNLR
IKFEMoxEQNLR
MTLDDFR
QFSSSYLSR
SGGGGGGGLGSGGSIR
SSYSRFSSSGGR
STMQELNSR
TLLDIDNTR
TLNDMoxRQEYEQLIAK
TLNDMRQEYEQLIAK	
230	Lactamase, beta 2 [Mus musculus]	Q99KR3 	32,8	5,8	183	51	15	ANIIYPGHGPVIHNAEAK
DNFEKSFTVTELR
EEQIISLFR
IFYTTTPVKK
IKANIIYPGHGPVIHNAEAK
ILIDTGEPSVPEYISCcamLK
MAEHNLLLHLR
MoxAEHNLLLHLR
NNREEQIISLFR
NPQREEIIGNGEQQFIYIENGDVVK
RILIDTGEPSVPEYISCcamLK
TMoxIYKDVPENLHK
VLGCcamNPGPMoxTLQGTNTYLVGTGSR
VLGCcamNPGPMoxTLQGTNTYLVGTGSRR
VLGCcamNPGPMTLQGTNTYLVGTGSR	
231	AMPL_MOUSE Cytosol aminopeptidase (Leucine aminopeptidase) (LAP) (Leucyl aminopeptidase) (Proline aminopeptidase) (Prolyl aminopeptidase)	Q9CPY7 	52,7	6,4	262	69	29	ADMoxGGAATICcamSAIVSAAK
DKDDDLPQFTSAGESFNK
GITFDSGGISIK
GSEEPPVFLEIHYMoxGSPNATEAPLVFVGK
HLMoxESPANEMoxTPTR
HLMoxESPANEMoxTPTRFAEIIEK
KGMoxSGRPTR
LFEASVETGDRVWR
LHGSGDLEAWEK
LHGSGDLEAWEKGVLFASGQNLAR
LILADALCcamYAHTFNPK
LNLPINIIGLAPLCcamENMoxPSGK
LREMoxLNISGPPLK
MoxPLFEHYTR
MPLFEHYTR
QVIDCcamQLADVNNLGKYR
SAGACcamTAAAFLR
SAGACcamTAAAFLREFVTHTK
SAGVDDQENWHEGKENIR
SWIEEQEMGSFLSVAK
SWIEEQEMoxGSFLSVAK
TFYGLHQDFPSVVVVGLGK
TFYGLHQDFPSVVVVGLGKR
TIQVDNTDAEGR
TIQVDNTDAEGRLILADALCcamYAHTFNPK
TKGLVLGIYAK
TRTFYGLHQDFPSVVVVGLGK
WAHLDIAGVMoxTNKDEIPYLR
WAHLDIAGVMTNK	
232	Lactate dehydrogenase 1, A chain; lactate dehydrogenase-A [Mus musculus]	P06151 	36,5	8,8	165	73	22	DLADELALVDVMoxEDK
DQLIVNLLKEEQAPQNK
FRYLMoxGER
GEMoxMoxDLQHGSLFLK
GLYGINEDVFLSVPCcamILGQNGISDVVK
GYTSWAIGLSVADLAESIMK
ITVVGVGAVGMACcamAISILMK
LKGEMoxMoxDLQHGSLFLK
LKGYTSWAIGLSVADLAESIMK
LKGYTSWAIGLSVADLAESIMoxK
LLIVSNPVDILTYVAWK
NVNIFKFIIPNIVK
oxGEMMDLQHGSLFLKTPK
oxITVVGVGAVGMACcamAISILMK
QQEGESRLNLVQR
QVVDSAYEVIK
RVHPISTMIK
RVHPISTMoxIK
SADTLWGIQK
SLNPELGTDADKEQWK
VIGSGCcamNLDSAR
VTLTPEEEAR	
233	Lactate dehydrogenase B (A Chain A, Human Heart L-Lactate Dehydrogenase H Chain, Ternary Complex With Nadh And Oxamate)	P07195 	36,5	5,6	117	32	10	EVHKMVVESAYEVIK
FRYLMoxAEK
GMoxYGIENEVFLSLPCcamILNAR
HRVIGSGCcamNLDSAR
IVADKDYSVTANSK
IVVVTAGVRQQEGESR
NVNVFKFIIPQIVK
QQEGESRLNLVQR
VIGSGCcamNLDSAR
VIGSGCcamNLDSARFR	
234	D-lactate dehydrogenase [Mus musculus]	Q7TNG8 	51,8	6,1	288	74	35	AFAENLGRR
ALALGGTCcamTGEHGIGLGKR
AVVGSPHVSTASAVR
AVVGSPHVSTASAVREQHGHDESMoxHR
AYSTDVCcamVPISR
AYSTDVCcamVPISRLPEILVETK
CcamQPPDAVVWPQNVDQVSR
DNVINLEVVLPDGR
DNVINLEVVLPDGRLLHTAGR
DSGLWFPVDPGADASLCcamGMoxAATGASGTNAVR
EQHGHDESMHR
EQHGHDESMoxHR
GSQGGLSQDFVEALK
GSQGGLSQDFVEALKAVVGSPHVSTASAVR
HNAWYAALALSPGSK
HSKLNCcamPVAPTLFLEFHGSQQTLAEQLQR
IEFLDDVMMDACcamNR
IEFLDDVMoxMoxDACcamNR
KSAAGYNLTGLFVGSEGTLGIITSTTLR
LHPAPEATVAATCcamAFPSVQAAVDSTVQILQAAVPVAR
LNCcamPVAPTLFLEFHGSQQTLAEQLQR
LPEILVETKEEIK
LTGAIVGHVGDGNFHCcamILLVDPDDAEEQR
LTGAIVGHVGDGNFHCcamILLVDPDDAEEQRR
NELWAARHNAWYAALALSPGSK
oxIEFLDDVMMDACcamNR
QLKNTLDPR
QLLQEEVGPVGVETMoxR
QLLQEEVGPVGVETMoxRQLK
QLLQEEVGPVGVETMR
RQLLQEEVGPVGVETMoxR
SAAGYNLTGLFVGSEGTLGIITSTTLR
VKAFAENLGR
YGTMoxRDNVINLEVVLPDGR
YGTMRDNVINLEVVLPDGR	
235	Lamin B1 [Mus musculus]	P14733 	66,9	5,1	162	48	23	ALDDTARER
ALYETELADAR
ALYETELADARR
ASAPATPLSPTR
ASAPATPLSPTRLSR
DQIAQLEASLSAAKK
ELNDRLAVYIDK
IGDTSVSYKYTSR
LALDMoxEISAYRK
LAQALHEMoxREQHDAQVR
LLEGEEERLK
LREYEAALNSK
LSSEMoxNTSTVNSAREELMoxESR
LVEVDSGRQIEYEYK
LYKEELEQTYHAK
MoxRIESLSSQLSNLQK
NQNSWGTGEDVKVILK
NSQGEEVAQR
QLADETLLKVDLENR
SLEGDLEDLKDQIAQLEASLSAAK
SLETENSALQLQVTER
SLETENSALQLQVTEREEVR
TTIPEEEEEEEEEPIGVAVEEER	
236	Similar to microsomal glutathione S-transferase 3 [Mus musculus]	Q283N4
	31	5,6	156	37	12	CcamHPDLAGRDLQQGTLTAESQR
DLQQGTLTAESQR
EQSQAGLTSLDTDDRLR
ERFGFPFVLAAR
FGFPFVLAAR
LQCcamQPESELR
LQCcamQPESELRTALGEVK
LQQLNAQYR
LQQLNAQYRER
LSDRATVPR
RLQCcamQPESELR
VNSMoxDFGEFVDVFGNIVEK	
237	Alpha enolase (Hypothetical protein LOC433182) [Mus musculus]	Q5FW97
	47,1	6,4	341	81	32	AASEKSCcamNCcamLLLK
AAVPSGASTGIYEALELR
AAVPSGASTGIYEALELRDNDK
AGAVEKGVPLYR
AGYTDQVVIGMDVAASEFYR
AGYTDQVVIGMoxDVAASEFYR
DATNVGDEGGFAPNILENKEALELLK
EAMoxRIGAEVYHNLK
EIFDSRGNPTVEVDLYTAK
FGANAILGVSLAVCcamK
FGANAILGVSLAVCcamKAGAVEK
FTASAGIQVVGDDLTVTNPK
FTASAGIQVVGDDLTVTNPKR
GNPTVEVDLYTAK
GVSQAVEHINK
GVSQAVEHINKTIAPALVSK
HIADLAGNPEVILPVPAFNVINGGSHAGNK
IGAEVYHNLK
IGAEVYHNLKNVIK
LAKYNQILR
LAMoxQEFMoxILPVGASSFR
LAMQEFMILPVGASSFR
LAQSNGWGVMoxVSHR
SCcamNCcamLLLKVNQIGSVTESLQACcamK
SFVQNYPVVSIEDPFDQDDWGAWQK
SGETEDTFIADLVVGLCcamTGQIK
SKFGANAILGVSLAVCcamK
SPDDPSRYITPDQLADLYK
VNQIGSVTESLQACcamK
YGKDATNVGDEGGFAPNILENK
YITPDQLADLYK
YNQILRIEEELGSK	
238	Lysophospholipase 1; phospholipase 1a; lysophopholipase 1 [Mus musculus]	P97823
	24,7	5,9	134	65	11	ALIDQEVKNGIPSNR
ALINPANVTFK
ASFSQGPINSANR
ATAAVIFLHGLGDTGHGWAEAFAGIK
DISVLQCcamHGDCcamDPLVPLMFGSLTVER
DISVLQCcamHGDCcamDPLVPLMoxFGSLTVER
IILGGFSQGGALSLYTALTTQQK
IYEGMoxMoxHSSCcamQQEMoxMoxDVK
KATAAVIFLHGLGDTGHGWAEAFAGIK
LAGVTALSCcamWLPLR
LKALINPANVTFK
NGIPSNRIILGGFSQGGALSLYTALTTQQK	
239	Mannose-6-phosphate receptor-binding protein 1
Unnamed protein product [Mus musculus]	Q9DBG5
	46,9	5,9	171	51	14	GLDRLQESLPILQQPTEK
LEPQIATASEYAHR
LQESLPILQQPTEK
LRNHAYEHSLGK
NHAYEHSLGKLQNAR
QEQNYFVR
QHCcamGVRGPEHSSHVAGGPLR
QKAQETLQQLTSVLGLMoxESVK
SQVNDLQATFSGIHSFQDLSAGVLAQTR
TLTTAAVSTAQPILSKLEPQIATASEYAHR
TPQDAEKDPAKPEQVEAR
VGQMoxVISGVDR
VGQMoxVISGVDRVLVK
VGQMVISGVDR
VLADTKELVSSTVSGAQEMoxVSSSVSSAK
VTGAVDVTLGAVQNSVDKTK	
240	Adenylate kinase isozyme 3 [Mus musculus]	Q9WTP7
	24,6	8,9	173	75	19	AVIMGAPGSGKGTVSSR
AVIMoxGAPGSGKGTVSSR
AYEAQTEPVLQYYQK
AYEAQTEPVLQYYQKK
GVLETFSGTETNKIWPHVYSFLQTK
HLSSGDLLR
ITKHFELK
LIPDDVMoxTR
LIPDDVMTR
LKAYEAQTEPVLQYYQK
QNMLQGTEIGVLAK
QNMoxLQGTEIGVLAK
TFIDQGKLIPDDVMoxTR
TFIDQGKLIPDDVMTR
TLTQCcamSWLLDGFPR
TVGIDDLTGEPLIQR
TVGIDDLTGEPLIQREDDKPETVIK
VYNIEFNPPK
WIHPASGR	
241	Methionine adenosyltransferase I, alpha; S-adenosylmethionine synthetase [Mus musculus]	Q91X83
	43,5	5,4	77	25	9	AVVPAKYLDEDTVYHLQPSGR
DLDLKKPIYQK
FVIGGPQGDAGVTGR
FVIGGPQGDAGVTGRK
NFDLRPGVIVR
SEFPWEVPK
SGVLPWLRPDSK
TQVTVQYMoxQDNGAVIPVR
YLDEDTVYHLQPSGR	
242	DEMSMC malate dehydrogenase (EC 1.1.1.37), cytosolic - mouse	P14152
	36,5	5,9	138	47	13	DLDVAVLVGSMoxPR
DLDVAVLVGSMPR
EVGVYEALKDDSWLK
FVEGLPINDFSREK
GEFITTVQQR
IFKSQGTALEK
LGVTADDVKNVIIWGNHSSTQYPDVNHAK
NVIIWGNHSSTQYPDVNHAK
SAPSIPKENFSCcamLTR
SVKVIVVGNPANTNCcamLTASK
TWKFVEGLPINDFSR
VIVVGNPANTNCcamLTASK
VLVTGAAGQIAYSLLYSIGNGSVFGK	
243	Malate dehydrogenase 2, NAD (mitochondrial); malate dehydrogenase, mitochondrial [Mus musculus]	P08249
	35,6	9	262	72	26	AGAGSATLSMAYAGAR
AGAGSATLSMoxAYAGAR
AKAGAGSATLSMAYAGAR
AKAGAGSATLSMoxAYAGAR
ANTFVAELKGLDPAR
ANVKGYLGPEQLPDCcamLK
ETECcamTYFSTPLLLGKK
FVFSLVDAMoxNGKEGVVECcamSFVQSK
GCcamDVVVIPAGVPR
GLDPARVNVPVIGGHAGK
GYLGPEQLPDCcamLK
GYLGPEQLPDCcamLKGCcamDVVVIPAGVPR
HGVYNPNKIFGVTTLDIVR
IQEAGTEVVKAK
ITPFEEKMoxIAEAIPELK
LTLYDIAHTPGVAADLSHIETR
MIAEAIPELK
MIAEAIPELKASIK
NLGIGKITPFEEK
TIIPLISQCcamTPK
TIIPLISQCcamTPKVDFPQDQLATLTGR
VAVLGASGGIGQPLSLLLK
VAVLGASGGIGQPLSLLLKNSPLVSR
VDFPQDQLATLTGR
VNVPVIGGHAGK
VNVPVIGGHAGKTIIPLISQCcamTPK	
244	Mesoderm development candidate 2; Unnamed protein product [Mus musculus]	Q9ERE7
	21,2	5	102	66	13	CcamAEVTLEGQMoxYPGK
CcamAEVTLEGQMoxYPGKGGGSK
CcamAEVTLEGQMYPGK
CcamAEVTLEGQMYPGKGGGSK
DGSYAWEIKDFLVSQDR
DIRDYNDADMAR
DIRDYNDADMoxAR
ETEEITSLWQGSLFNANYDVQR
FIVGSDRAIFMLR
FIVGSDRAIFMoxLR
LLEQWEKDDDIEEGDLPEHK
RPSAPIDFSK
RPSAPIDFSKLDPGKPESILK	
245	Protein DJ-1; Parkinson disease protein 7 homolog; MGC98210 protein [Xenopus laevis]	Q99LX0
	20	6,4	178	84	15	DKMoxMoxNGSHYSYSESR
DVMoxICcamPDTSLEDAK
EILKEQESR
GAEEMETVIPVDVMR
GAEEMETVIPVDVMRR
GAEEMoxETVIPVDVMoxR
GAEEMoxETVIPVDVMoxRR
GLIAAICcamAGPTALLAHEVGFGCcamK
GPGTSFEFALAIVEALVGK
GPGTSFEFALAIVEALVGKDMoxANQVK
KGLIAAICcamAGPTALLAHEVGFGCcamK
MoxMoxNGSHYSYSESR
oxDKMMNGSHYSYSESR
oxGAEEMETVIPVDVMR
TQGPYDVVVLPGGNLGAQNLSESPMoxVK
VEKDGLILTSR
VTVAGLAGKDPVQCcamSR	
246	3-mercaptopyruvate sulfurtransferase [Mus musculus]	Q99J99
	33	6	203	67	19	AAGRFQGTQPEPR
AFGHHSVSLLDGGFR
AFGHHSVSLLDGGFRHWLNQNLPISSGK
ALVSAQWVAEALKAPR
AQPEHIISEGR
DGIEPGHIPGSVNIPFTEFLTNEGLEK
FQGTQPEPR
HIPGAAFFDIDR
HWLNQNLPISSGK
KVDLSKPLVATCcamGSGVTACcamHVVLGAFLCcamGK
LLDASWYLPK
SDVPVYDGSWVEWYMoxR
SDVPVYDGSWVEWYMR
SHSEPAEFSAQLDPSFIK
SHSEPAEFSAQLDPSFIKTHEDILENLDAR
SSQPLKLLDASWYLPK
THEDILENLDAR
THEDILENLDARR
VDLSKPLVATCcamGSGVTACcamHVVLGAFLCcamGK	
247	Major urinary protein 1; A Chain A, Crystal Structure Of Recombinant Major Mouse Urinary Protein (Rmup) At 1.75 A Resolution	Q4FZE8
	18,7	4,7	116	57	9	AGEYSVTYDGFNTFTIPK
EKIEDNGNFR
ENIIDLSNANR
FAQLCcamEEHGILR
FAQLCcamEEHGILRENIIDLSNANR
IEDNGNFRLFLEQIHVLEK
INGEWHTIILASDKR
LFLEQIHVLEK
TDYDNFLMoxAHLINEK	
248	uMUP-VIII=18.695 kda major urinary protein [mice, Balb/c, urine, Peptide, 162 aa]	P02762
	18,7	4,7	138	79	13	AGEYSVTYDGFNTFTIPK
DGETFQLMGLYGR
DGETFQLMoxGLYGR
EKIEDNGNFR
ENIIDLSNANR
FAQLCcamEEHGILR
FAQLCcamEEHGILRENIIDLSNANR
FHTVRDEECcamSELSMoxVADK
FHTVRDEECcamSELSMVADK
INGEWHTIILASDKR
LFLEQIHVLENSLVLK
TDYDNFLMAHLINEK
TDYDNFLMoxAHLINEK	
249	Nascent-polypeptide-associated complex alpha polypeptide [Homo sapiens]	Q13765
	23,4	4,5	93	41	9	ALKNNSNDIVNAIMoxELTMox
DIELVMoxSQANVSR
IEDLSQQAQLAAAEK
IEDLSQQAQLAAAEKFK
LGLRQVTGVTR
NILFVITKPDVYK
oxALKNNSNDIVNAIMELTM
SKNILFVITKPDVYK
SPASDTYIVFGEAK	
250	N-ethylmaleimide-sensitive factor attachment protein, gamma; soluble NSF attachment protein; gamma SNAP [Homo sapiens]	Q99747
	34,7	5,2	130	52	15	ASMoxMoxYLENGTPDTAAMoxALER
AVQLYQQTANVFENEER
AVQLYQQTANVFENEERLR
EIENYPTCcamYKK
EMoxQKLPEAVQLIEK
FDEAALSIQKEK
INEGLEHLAK
LGLSLVVPGGGIK
LGLSLVVPGGGIKK
LIENVDPEKAVQLYQQTANVFENEER
oxAYEQAGMMLKEMQK
QFEQAKDACcamLR
TIAQVLVHLHR
TIAQVLVHLHRNDYVAAER
WKPDYDSAASEYGK	
251	N-myc downstream regulated gene 2; N-myc downstream regulated 2 [Mus musculus]	Q9QYG0
	40,8	5,2	212	65	16	CcamPVMoxLVVGDQAPHEDAVVECcamNSK
DLNFERGGETTLK
FGDMoxQEIIQNFVR
FGDMQEIIQNFVR
GIIQHAPNLENIELYWNSYNNR
GIIQHAPNLENIELYWNSYNNRR
ILLDQGQTHSVETPYGSVTFTVYGTPKPK
LDPTQTSFLK
LTGLTSSIPDMoxILGHLFSQEELSGNSELIQK
MoxADSGGQPQLTQPGK
MoxADSGGQPQLTQPGKLTEAFK
RPAIFTYHDVGLNYK
SCcamFQPLFR
TASLTSAASIDGSR
TASLTSAASIDGSRSR
YALNHPDTVEGLVLINIDPNAK
YFLQGMoxGYMoxASSCcamMoxTR	
252	NADH dehydrogenase [ubiquinone] 1 alpha subcomplex subunit 10,  mitochondrial precursor (EC 1.6.5.3) (EC 1.6.99.3) (NADH-ubiquinone  oxidoreductase 42 kDa subunit) (Complex I-42kD) (CI-42kD). Unnamed protein product [Mus musculus]	O95299
	38,1	6,4	171	55	15	FYDDPKSNDGNSYR
IYNSFRELPGR
LLQYADALEHLLSTGQGVVLER
LQSWLYASR
LRYGLLAAILGDK
LTLPEYLPPHAVIYIDVPVPEVQSR
QCcamVDHYNEIKR
QDDWTFHYLR
SIYSDFVFLEAMoxYNQGYIR
SIYSDFVFLEAMoxYNQGYIRK
SNDGNSYRLQSWLYASR
VITVDGNICcamSGK
VTSAYLQDIENAYKK
YAPGYNAEVGDKWIWLK
YGLLAAILGDKTTK	
253	NADH dehydrogenase (ubiquinone) 1 alpha subcomplex, 8 [Mus musculus]	Q9DCJ5
	20	8,8	145	56	13	ARPEPNPVIEGDLKPAK
ARPEPNPVIEGDLKPAKHGTR
FDQCcamVLDKLGWVRPDLGQLSK
HGTRFFFWTV
LGWVRPDLGQLSK
LVNGCcamALNFFR
PGIVELPTLEELKVEEVK
TDRPLPENPYHSR
VKTDRPLPENPYHSR
WEEKDPR	
254	NADH dehydrogenase (ubiquinone) Fe-S protein 1 (Ndufs1)  (unnamed protein product) [Mus musculus]	Q91VD9
	79,7	5,5	240	51	28	ALSEIAGITLPYDTLDQVR
AVEDKNIGPLVK
DDGAAILVAVSNMoxVQK
FASEIAGVDDLGTTGR
FEAPLFNAR
GLLTYTSWEDALSR
GWNILTNSEK
HSFCcamEVLKDAK
IASQVAALDLGYKPGVEAIR
IASQVAALDLGYKPGVEAIRK
KPMoxVVLGSSALQR
KPMVVLGSSALQR
KTESIDVMoxDAVGSNIVVSTR
LVNQEVLADPLVPPQLTIKDFYMoxTDSISR
LVNQEVLADPLVPPQLTIKDFYMTDSISR
MCcamLVEIEKAPK
MHEDINEEWISDKTR
MLFLLGADGGCcamITR
MoxHEDINEEWISDKTR
MoxLFLLGADGGCcamITRQDLPK
NPPKMoxLFLLGADGGCcamITR
SATYVNTEGR
SNYLLNTTIAGVEEADVVLLVGTNPR
VALIGSPVDLTYR
VALIGSPVDLTYRYDHLGDSPK
VAVTPPGLAREDWK	
255	NADH dehydrogenase (ubiquinone) Fe-S protein 3 [Mus musculus]	Q9DCT2
	30,1	6,8	204	48	16	DFPLTGYVELR
ESAAADKRPTVRPR
FDLNSPWEAFPAYR
FDLNSPWEAFPAYRQPPESLK
FEIVYNLLSLR
FEIVYNLLSLRFNSR
ILTDYGFEGHPFR
ILTDYGFEGHPFRK
KFDLNSPWEAFPAYR
QLSAFGEYVAEILPK
QNRFEIVYNLLSLR
SDVTHKQLSAFGEYVAEILPK
SLADLTAVDVPTR
SLADLTAVDVPTRQNR
VVAEPVELAQEFR
VVAEPVELAQEFRK	
256	Ndufv2 protein [Mus musculus]	Q9D6J6
	27,3	6,7	166	62	13	ATGLAAQWGRHAR
DSDSILETLQRK
DTPENNPDTPFDFTPENYKR
FCcamCcamEPAGGLTSLTEPPKGPGFGVQAGL
IEAIVKNYPEGHQAAAVLPVLDLAQR
NYPEGHQAAAVLPVLDLAQR
QNGWLPISAMNK
QNGWLPISAMoxNK
VAEVLQVPPMoxR
VAEVLQVPPMR
VYEVATFYTMoxYNR
VYEVATFYTMoxYNRKPVGK
VYEVATFYTMYNR
YHIQVCcamTTTPCcamMLR
YHIQVCcamTTTPCcamMoxLR	
257	Nit protein 2 [Mus musculus]	Q9JHW2
	30,5	6,2	347	91	23	ADLYTVESKKP
AGTEETILYSDIDLK
AGTEETILYSDIDLKK
ARAVDNQVYVATASPAR
ASYVAWGHSTVVDPWGQVLTK
AVDNQVYVATASPAR
AVDNQVYVATASPARDDK
DDKASYVAWGHSTVVDPWGQVLTK
ESSIYLIGGSIPEEDAGK
FAELAQIYAQR
GCcamQLLVYPGAFNLTTGPAHWELLQR
IHLFDIDVPGK
IHLFDIDVPGKITFQESK
KIHLFDIDVPGK
LAEIRQQIPILK
LALIQLQVSSIK
LALIQLQVSSIKSDNLTR
LYNTCcamSVFGPDGSLLVK
QGANIVSLPECcamFNSPYGTTYFPDYAEKIPGESTQK
RADLYTVESK
TLSPGDSFSTFDTPYCcamK
VGLGICcamYDMoxR
VGLGICcamYDMR	
258	Nucleoside-diphosphate kinase 2; nucleoside diphosphate kinase B [Mus musculus]	Q01768
	17,4	6,6	147	80	14	ASEEHLKQHYIDLK
DRPFFPGLVK
NIIHGSDSVESAEKEIHLWFKPEELIDYK
oxYMNSGPVVAMVWEGLNVVK
oxYMNSGPVVAMVWEGLNVVKTGR
QHYIDLKDRPFFPGLVK
SCcamAHDWVYE
TFIAIKPDGVQR
TGRVMoxLGETNPADSKPGTIR
VMLGETNPADSKPGTIR
VMLGETNPADSKPGTIRGDFCcamIQVGR
VMoxLGETNPADSKPGTIR
VMoxLGETNPADSKPGTIRGDFCcamIQVGR
YMoxNSGPVVAMoxVWEGLNVVK	
259	Alpha-tubulin [Macaca mulatta]	Q862E5
	49,1	4,9	215	68	23	AFVHWYVGEGMoxEEGEFSEAR
AVCcamMLSNTTAIAEAWAR
AVCcamMoxLSNTTAIAEAWAR
AVFVDLEPTVIDEVR
AVFVDLEPTVIDEVRTGTYR
AYHEQLSVAEITNACcamFEPANQMoxVK
FDGALNVDLTEFQTNLVPYPR
GHYTIGKEIIDLVLDR
HGKYMoxACcamCcamLLYR
IHFPLATYAPVISAEK
KLADQCcamTGLQGFLVFHSFGGGTGSGFTSLLMoxER
LDHKFDLMoxYAK
LIGQIVSSITASLR
NLDIERPTYTNLNR
QLFHPEQLITGK
QLFHPEQLITGKEDAANNYAR
TIGGGDDSFNTFFSETGAGK
TIGGGDDSFNTFFSETGAGKHVPR
TIQFVDWCcamPTGFK
VGINYQPPTVVPGGDLAK
YMACcamCcamLLYRGDVVPK
YMoxACcamCcamLLYR
YMoxACcamCcamLLYRGDVVPK	
260	Similar to peroxiredoxin 1 [Bos taurus]	Q91Z81 	11,6	5,6	104	69	8	GLFIIDDKGILR
IGYPAPNFK
LNCcamQVIGASVDSHFCcamHLAWINTPKK
QGGLGPMNIPLISDPKR
QGGLGPMoxNIPLISDPKR
QITINDLPVGR
RTIAQDYGVLK
TIAQDYGVLKADEGISFR	
261	ERP57 protein [Cricetulus griseus]	Q9GLW6 	56,8	5,9	133	41	18	AASNLRDNYR
DLFSDGHSEFLK
DLLTAYYDVDYEK
FISDKDASVVGFFR
FLDAGHKLNFAVASR
FVMoxQEEFSR
FVMoxQEEFSRDGK
FVMQEEFSR
GFPTIYFSPANK
IFRDGEEAGAYDGPR
KTFSHELSDFGLESTTGEVPVVAIR
LAPEYEAAATR
MDATANDVPSPYEVK
TFSHELSDFGLESTTGEVPVVAIR
TVVYTEQKMTSGK
VDCcamTANTNTCcamNK
VDCcamTANTNTCcamNKYGVSGYPTLK
YLKSEPIPETNDGPVK	
262	NAD(P)H dehydrogenase, quinone 2; NAD(P)H menadione oxidoreductase 2, dioxin inducible; NRH: quinone oxidoreductase [Mus musculus]	Q9JI75 	26,2	6,3	203	79	18	ALTSDIFEEQR
ALTSDIFEEQRK
ATRNDITGAPSNPDVFSYGIETHEAYK
DGVSGDFRYFLWPLQHGTLHFCcamGFK
GFAFDIPGFYDSGFLK
GFAFDIPGFYDSGFLKGK
KVLIVYAHQEPK
KVMLASWAQR
KVMoxLASWAQR
LALLSLTTGGTAEMoxYTK
LALLSLTTGGTAEMYTK
NDITGAPSNPDVFSYGIETHEAYK
NDITGAPSNPDVFSYGIETHEAYKK
QGCcamTVTVSDLYSMoxNFEPR
SIWKEEPIHCcamTPPWYFQE
VLAPQISFGLDVSSEEER
VLAPQISFGLDVSSEEERK
VLIVYAHQEPKSFNGSLK
VMoxLASWAQR
YFLWPLQHGTLHFCcamGFK	
263	NF1C_MOUSE NSFL1 cofactor p47 (p97 cofactor)	Q9CZ44 	40,7	5	234	71	22	ASSSILINEAEPTTNIQIR
DLIHDQDEEEEEEEGQR
DLIHDQDEEEEEEEGQRFYAGGSER
EANLLNAVIVQR
EFVAVTGTEEDRAR
ELADENQTLKEANLLNAVIVQR
GAFKAFTGEGQK
GAKEHGAVAVER
GTAPSDNRVTSFR
HSGQDVHVVLKLWK
LAHGGQVNLDMoxEDHRDEDFVKPK
LGAAPEEESAYVAGER
LGAAPEEESAYVAGERR
LGSTAPQVLNTSSPAQQAENEAK
LVQKFNHSHR
SPGETSKPRPFAGGGYR
SPNELVDDLFK
SPNELVDDLFKGAK
SYQDPSNAQFLESIR
SYQDPSNAQFLESIRR
TGFSLDNGDLR
VTKSPGETSKPRPFAGGGYR	
264	Nudix (nucleoside diphosphate linked moiety X)-type motif 7; coenzyme A diphosphatase [Mus musculus]	Q99P30 	26,9	5,5	85	37	10	DFIMoxHCcamFEYK
EPGEVCcamFPGGK
EPGEVCcamFPGGKR
FSVLVPLLAR
GGKLYLMFTVR
IDFDLHDLIPSCcamER
LYLMoxFTVR
RDPVDTDDTATALR
TFLWRYSLSK
YSHLSSNKFSVLVPLLAR	
265	Ornithine aminotransferase	P29758 	48,4	6,1	301	69	29	AFYNNVLGEYEEYITK
AKIVFADGNFWGR
ALQDPNVAAFMoxVEPIQGEAGVIVPDPGYLTGVR
DCcamDAWKVCcamLR
ELCcamTRHQVLFIADEIQTGLAR
ELMKLPSDVVTSVR
ELMoxKLPSDVVTSVR
ESKYGAHNYHPLPVALER
GKGIYMoxWDVEGR
GKGIYMWDVEGR
GKGLLNAIVIR
GLLNAIVIRETK
HQVLFIADEIQTGLAR
IAIAALEVLEEENLAENADKMGAILR
IAIAALEVLEEENLAENADKMoxGAILR
IVFADGNFWGR
KTEQGPPSSEYIFER
LRDNGLLAKPTHGDIIR
LSKLASLQTIAALR
QYFDFLSAYGAVSQGHCcamHPK
SQVDKLTLTSR
TEQGPPSSEYIFER
TEQGPPSSEYIFERESK
TGRWLAVDHENVRPDMoxVLLGK
VLPMoxNTGVEAGETACcamK
WGYTVKGIQK
WLAVDHENVRPDMoxVLLGK
WLAVDHENVRPDMVLLGK
YGAHNYHPLPVALERGK	
266	Serine protease OMI [Mus musculus]	Q9R108 	49,3	9,6	140	34	12	AGLRPGDVILAIGEK
EPLPTLPLGR
IQTKEPLPTLPLGR
KNSWFGTSGSQR
LAQNAEDVYEAVR
NSWFGTSGSQR
NSWFGTSGSQRR
RGSETLTLYVTPEVTE
SQYNFIADVVEK
TAPAVVYIEILDRHPFSGR
VTAGISFAIPSDR
VTAGISFAIPSDRLR	
267	Otc protein [Mus musculus]	P11725 	39,4	8,8	214	68	26	EASIPIVNGLSDLYHPIQILADYLTLQEHYGSLK
FGMHLQAATPK
FGMoxHLQAATPK
GLTLSWIGDGNNILHSIMoxMoxSAAK
GYEPDPNIVK
GYEPDPNIVKLAEQYAK
KPEEVDDEVFYSPR
KWTIMoxVSLR
LQAFQGYQVTMK
LQAFQGYQVTMoxK
LQAFQGYQVTMoxKTAK
LSMoxTNDPLEAAR
LSMTNDPLEAAR
LSTETGFALLGGHPSFLTTQDIHLGVNESLTDTAR
QKGEYLPLLQGK
RLQAFQGYQVTMK
RLQAFQGYQVTMoxK
SLGMIFEKR
SLGMoxIFEKR
SLVFPEAENR
SLVFPEAENRK
TAKVAASDWTFLHCcamLPR
VAASDWTFLHCcamLPR
VLSSMoxTDAVLAR
VLSSMTDAVLAR
VYKQSDLDTLAK	
268	RIKEN cDNA 4933425A18 [Mus musculus]	Q9D404 	48,6	6,5	149	56	15	DAGVSPEQISYVNAHATSTPLGDAAENR
DGFVMoxGEGAAVLVLEEHEHAVQR
FIAHGDADVMoxVAGGTDSCcamISPLSLAGFSR
GATGHLLGAAGAVEATFTALACcamYHQK
GPHEGQFNEENFVSK
GPHEGQFNEENFVSKSDAK
GYNKVSPFFVPK
ILINMoxAAGQVSIR
ILINMoxAAGQVSIRYK
IYAEILGYGLSGDAGHITAPDPEGEGALR
LACcamRPFHPER
LFRDHACcamALAISSTK
LKGPNHSVSTACcamTTGAHAVGDSFR
LLRGESGIVSVVGDEYK
NIPCcamSVAAYVPR	
269	Prolyl 4-hydroxylase, beta polypeptide [Mus musculus]	P09103 	57,1	4,7	279	59	33	ALAPEYAK
EADDIVNWLK
EYTAGREADDIVNWLK
FDEGRNNFEGEITK
FFPASADR
GYPTIKFFK
IFGGEIKTHILLFLPK
IKPHLMoxSQEVPEDWDKQPVK
ILEFFGLK
ILEFFGLKK
ILFIFIDSDHTDNQR
ITEFCcamHR
KSNFEEALAAHK
LGETYKDHENIIIAK
LITLEEEMoxTK
LITLEEEMTK
LKAEGSEIR
LLDFIKHNQLPLVIEFTEQTAPK
MDSTANEVEAVKVHSFPTLK
MoxDSTANEVEAVKVHSFPTLK
NGDTASPKEYTAGR
QFLLAAEAIDDIPFGITSNSGVFSK
SNFEEALAAHK
THILLFLPK
TLDGFKK
TVIDYNGER
VDATEESDLAQQYGVR
VLVGANFEEVAFDEK
VLVGANFEEVAFDEKK
YKPESDELTAEKITEFCcamHR
YQLDKDGVVLFK	
270	Polyadenylate binding protein II - human	P11940 	58,5	9,3	239	47	25	AKEFTNVYIK
ALDTMoxNFDVIKGKPVR
ALYDTFSAFGNILSCcamK
AVDEMNGKELNGK
EFSPFGTITSAK
FGPALSVKVMoxTDESGK
FSPAGPILSIR
GFGFVCcamFSSPEEATK
GFGFVSFERHEDAQK
GYGFVHFETQEAAER
ITGMoxLLEIDNSELLHMoxLESPESLR
ITRYQGVNLYVK
IVATKPLYVALAQR
KEFSPFGTITSAK
MoxNGMoxLLNDRK
NLDDGIDDERLR
QAHLTNQYMoxQR
QAHLTNQYMQR
RSLGYAYVNFQQPADAER
SKGFGFVCcamFSSPEEATK
SKGFGFVSFER
SKVDEAVAVLQAHQAK
SLGYAYVNFQQPADAER
YQGVNLYVK	
271	DJ-1 protein [Mus musculus]	Q99LX0 	20	6,4	134	84	17	DKMoxMoxNGSHYSYSESR
DVMoxICcamPDTSLEDAK
EILKEQESR
GAEEMETVIPVDVMR
GAEEMETVIPVDVMRR
GAEEMoxETVIPVDVMoxR
GAEEMoxETVIPVDVMoxRR
GLIAAICcamAGPTALLAHEVGFGCcamK
GPGTSFEFALAIVEALVGK
GPGTSFEFALAIVEALVGKDMoxANQVK
KGLIAAICcamAGPTALLAHEVGFGCcamK
MoxMoxNGSHYSYSESR
oxDKMMNGSHYSYSESR
oxGAEEMETVIPVDVMR
TQGPYDVVVLPGGNLGAQNLSESPMoxVK
VEKDGLILTSR
VTVAGLAGKDPVQCcamSR	
272	MAWD binding protein homolog 1 [Mus musculus]	Q9DCG6 	32	5,1	318	82	27	AAIGDTLVQDIR
AEDGIVLDFPVYPTFPQDFHEVEDLIK
EMNLSETAFIR
EMNLSETAFIRK
EMoxNLSETAFIR
EMoxNLSETAFIRK
GEPGGQTAPYDFYSR
GGELDISLRPDGR
GLILTVKGEPGGQTAPYDFYSR
GNPAAVCcamLLER
KLQPTDSFTQSSR
LQPTDSFTQSSR
LSDSYDRSFLESLK
MKLPIFIADAFTATAFR
MoxKLPIFIADAFTATAFR
NSTLTFVTMoxSGELK
NSTLTFVTMoxSGELKAR
NSTLTFVTMSGELK
NSTLTFVTMSGELKAR
RAEDGIVLDFPVYPTFPQDFHEVEDLIK
RGGELDISLRPDGR
SFLESLKVNTEPLPAIEK
TLEEDAHQQIAR
WFTPVSEVPLCcamGHATLASAAVLFHK	
273	Propionyl-Coenzyme A carboxylase, alpha polypeptide [Mus musculus]	Q91ZA3 	79,9	6,7	263	53	31	EAGGNMoxSIQFLGTVYK
EAGGNMoxSIQFLGTVYKVHILTK
EIGYPVMoxIK
FLSDVYPDGFKGHTLTLSER
FSSQEAASSFGDDR
FSSQEAASSFGDDRLLIEK
GVTHNIPLLR
GVTHNIPLLREVIINTR
HIEIQVLGDKHGNALWLNER
HKQEDIPISGWAVECcamR
IAWDDEETRDGFR
LAAEDVTFIGPDTHAIQAMGDKIESK
LAAEDVTFIGPDTHAIQAMoxGDKIESK
LAAELNKFMoxLEK
LQVEHPVTECcamITGLDLVQEMoxILVAK
LSQYQEPIHLPGVR
MADEAVCcamVGPAPTSK
MEDALDNYVIR
MoxADEAVCcamVGPAPTSK
MoxEDALDNYVIR
NFYFLEMNTR
NFYFLEMoxNTR
NQKVVEEAPSIFLDPETR
NQLLAIASSVFVASQLR
RMEDALDNYVIR
SYLNMDAIMEAIKK
TVAIHSDVDASSVHVK
VDSGIQPGSDISIYYDPMoxISK
VNTIPGFDGVVKDADEAVR
VPVIRPDVAKWELSVK
VVEEAPSIFLDPETR
YSSAGTVEFLVDSQK	
274	Phosphoenolpyruvate carboxykinase 1, cytosolic [Mus musculus]	Q9Z2V4 	69,3	6,2	179	34	19	AINPENGFFGVAPGTSVK
EIISFGSGYGGNSLLGK
FLWPGFGENSR
IFHVNWFR
IFHVNWFRK
IGIELTDSPYVVASMoxR
IGIELTDSPYVVASMR
LTPIGYIPKENALNLK
MoxGISVLEALGDGEFIK
NKEWRPQDAEPCcamAHPNSR
oxTMYVIPFSMGPLGSPLAK
PPQLHNGLDFSAK
TMoxYVIPFSMoxGPLGSPLAK
TVIITQEQRDTVPIPK
VIQGSLDSLPQAVR
VIQGSLDSLPQAVRK
VLEWMFGRIEGEDSAK
VLEWMoxFGRIEGEDSAK
YLAAAFPSACcamGK	
275	Phosphatidylcholine transfer protein [Mus musculus]	P53808 	20,5	8,9	128	49	9	IYVVLAQSISAPQFPEK
IYVVLAQSISAPQFPEKSGVIR
KIYVVLAQSISAPQFPEK
LLDQPSGLYEYK
NGVPNFLKDMoxVK
NGVPNFLKDMVK
VFGVLEGCcamSPALLADVYMoxDLDYR
VFGVLEGCcamSPALLADVYMoxDLDYRK
YPFPLSNRDYVYTR	
276	Pyruvate carboxylase; pyruvate decarboxylase [Mus musculus]	Q05920 	129,7	6,2	382	46	47	ADFAQACcamQDAGVR
AEAEAQAEELSFPR
AYSEALAAFGNGALFVEK
AYSEALAAFGNGALFVEKFIEKPR
DFTATFGPLDSLNTR
DMAGLLKPAACcamTMLVSSLR
EGPEGFARAVR
ENGVDAVHPGYGFLSER
ENGVDAVHPGYGFLSERADFAQACcamQDAGVR
FCcamEVAKENGMDVFR
FCcamEVAKENGMoxDVFR
FIGPSPEVVR
FIGPSPEVVRK
GANAVGYTNYPDNVVFK
GLAPVQAYLHIPDIIK
GLYAAFDCcamTATMoxK
GTPLDTEVPLER
HIEVQILGDQYGNILHLYER
HQKVVEIAPATHLDPQLR
HYFIEVNSR
IAEEFEVELER
IAPYVAHNFNK
IEGRPGASLPPLNLK
INGCcamAIQCcamR
IVGDLAQFMoxVQNGLSR
KIAPYVAHNFNK
LDNASAFQGAVISPHYDSLLVK
LQELRELIPNIPFQMoxLLR
LQVEHTVTEEITDVDLVHAQIHVSEGR
MoxETVVTSPMoxEGTIRK
QKADEAYLIGR
QVFFELNGQLR
SFQPDTGRIEVFR
SILVKDTQAMoxK
SLPDLGLRQENIR
SVVEFLQGYIGIPHGGFPEPFR
TKYSLEYYMoxGLAEELVR
TVAVYSEQDTGQMoxHR
VAKENGVDAVHPGYGFLSER
VFDYSEYWEGAR
VIAHGKDHPTAATK
VVEIAPATHLDPQLR
VVHSYEELEENYTR	
277	Phosphate cytidylyltransferase 2, ethanolamine [Mus musculus]	Q922E4 	45,2	6	144	54	18	ELAFLEATKQQEAPPGGEID
GIFYQIDSGSDLTTDLIVQR
GKNYPIMoxNLHER
HKGPPVFTQEER
NGHGAASAAGLKGPGDQR
NYPIMoxNLHER
RPYVIAGLHFDQEVNR
RTQGVSTTDLVGR
TQGVSTTDLVGR	
278	Pyruvate dehydrogenase E1 alpha 1; pyruvate dehydrogenase E1alpha subunit [Mus musculus]	P35486 	43,2	8,5	114	44	12	EATKFAAAYCcamR
GDFIPGLRVDGMoxDILCcamVR
GFCcamHLCcamDGQEACcamCcamVGLEAGINPTDHLITAYR
GLPVRAILAELTGR
LEEGPPVTTVLTR
LEEGPPVTTVLTREDGLK
LPCcamIFICcamENNR
MoxVNSNLASVEELKEIDVEVR
NFYGGNGIVGAQVPLGAGIALACcamK
SGKGPILMELQTYR
SGKGPILMoxELQTYR
TREEIQEVR	
279	Pyruvate dehydrogenase (lipoamide) beta [Mus musculus]	Q9D051 	38,9	6,5	229	64	20	EAINQGMDEELERDEK
EAINQGMoxDEELERDEK
EGIECcamEVINLR
GPNGASAGVAAQHSQCcamFAAWYGHCcamPGLK
IMEGPAFNFLDAPAVR
IMoxEGPAFNFLDAPAVR
oxTIRPMDIEAIEASVMK
QGTHITVVAHSRPVGHCcamLEAAAVLSK
SAIRDNNPVVMoxLENELMoxYGVAFELPAEAQSK
TIRPMDIEAIEASVMK
TIRPMoxDIEAIEASVMoxK
TNHLVTVEGGWPQFGVGAEICcamAR
VFLLGEEVAQYDGAYKVSR
VLEDNSVPQVKDIIFAVK
VTGADVPMoxPYAK
VTGADVPMPYAK
VVSPWNSEDAK
VVSPWNSEDAKGLIK	
280	Protein disulfide isomerase associated 3 ; unnamed protein product [Mus musculus]	P27773 	56,6	5,7	268	66	35	ALEQFLQEYFDGNLKR
DGEEAGAYDGPR
DLFSDGHSEFLK
DLLTAYYDVDYEK
DLLTAYYDVDYEKNAK
EATNPPIIQEEKPK
ELNDFISYLQR
FIQDSIFGLCcamPHMoxTEDNKDLIQGK
FISDKDASVVGFFR
FLDAGHKLNFAVASR
FVMoxQEEFSR
FVMoxQEEFSRDGK
FVMQEEFSR
FVMQEEFSRDGK
GFPTIYFSPANK
IFRDGEEAGAYDGPR
KTFSHELSDFGLESTTGEVPVVAIR
LAPEYEAAATR
MRFSCcamLALLPGVALLLASAR
QAGPASVPLRTEEEFK
TADGIVSHLKK
TFSHELSDFGLESTTGEVPVVAIR
VDCcamTANTNTCcamNKYGVSGYPTLK
YGVSGYPTLK
YLKSEPIPESNEGPVK	
281	Protein disulfide isomerase-associated 6 [Mus musculus]	Q922R8 	48,7	5	146	50	21	ALDLFSDNAPPPELLEIINEDIAK
ALDLFSDNAPPPELLEIINEDIAKK
GESPVDYDGGR
GSFSEQGINEFLR
GSFSEQGINEFLRELSFGR
GSTAPVGGGSFPTITPR
IFGANKNKPEDYQGGR
IFQKGESPVDYDGGR
LAAVDATMNQVLASR
NKPEDYQGGR
NKPEDYQGGRTGEAIVDAALSALR
NLEPEWAAAATEVKEQTK
NSYLEVLLK
SGGYSSGKQGR
TCcamEEHQLCcamVVAVLPHILDTGAAGR
TGEAIVDAALSALR
TGEAIVDAALSALRQLVK
VGAVNADKHQSLGGQYGVQGFPTIK
VKLAAVDATMNQVLASR
VKLAAVDATMoxNQVLASR
YGIKGFPTIK	
282	Pdlim1 protein [Mus musculus]	O70400 	35,8	6,2	177	78	19	AAIANLCcamIGDLITAIDGEDTSSMoxTHLEAQNK
CcamGTGIVGVFVK
DFEQPLAISR
DHHRHPECcamYVCcamTDCcamGINLK
GCcamADNMoxTLTVSR
GHFFVEDQIYCcamEK
HPYKMoxNLASEPQEVLHIGSAHNR
IKGCcamADNMoxTLTVSR
IKGCcamADNMTLTVSR
IWSPLVTEEGKR
LVGGKDFEQPLAISR
MNLASEPQEVLHIGSAHNR
MoxNLASEPQEVLHIGSAHNR
QKGHFFVEDQIYCcamEK
QSTSFLVLQEILESDGKGDPNKPSGFR
SAMPFTASPAPSTR
TSASGEEANSRPVVQPHPSGSLIIDKDSEVYK
VITNQYNSPTGLYSSENISNFNNAVESK
VTPPEGYDVVTVFRE	
283	Phosphatidylethanolamine binding protein [Mus musculus]	P70296 	20,8	5,2	135	86	14	AADISQWAGPLCcamLQEVDEPPQHALR
EWHHFLVVNMK
EWHHFLVVNMoxK
FREWHHFLVVNMK
FREWHHFLVVNMoxK
GNDISSGTVLSDYVGSGPPSGTGLHR
KYNLGAPVAGTCcamYQAEWDDYVPK
LYTLVLTDPDAPSR
LYTLVLTDPDAPSRK
VDYAGVTVDELGK
VLTPTQVMNRPSSISWDGLDPGK
VLTPTQVMoxNRPSSISWDGLDPGK
YNLGAPVAGTCcamYQAEWDDYVPK
YVWLVYEQEQPLSCcamDEPILSNK	
284	Phosphoglycerate mutase 1 (brain) [Homo sapiens]	P18669 	28,8	6,8	224	72	18	ALPFWNEEIVPQIK
ALPFWNEEIVPQIKEGK
DAGYEFDICcamFTSVQK
DAGYEFDICcamFTSVQKR
FSGWYDADLSPAGHEEAK
FSGWYDADLSPAGHEEAKR
HGEAQVKIWR
HGESAWNLENR
HYGGLTGLNKAETAAK
LNERHYGGLTGLNK
NLKPIKPMoxQFLGDEETVR
RVLIAAHGNSLR
SYDVPPPPMoxEPDHPFYSNISK
SYDVPPPPMoxEPDHPFYSNISKDR
TLWTVLDAIDQMWLLVVR
VLIAAHGNSLR
VLIAAHGNSLRGIVK
YADLTEDQLPSCcamESLKDTIAR	
285	Phosphoglycerate kinase 1 [Mus musculus]	P09411 	44,6	8	133	72	23	AEPAKIDAFR
AHSSMoxVGVNLPQK
AHSSMVGVNLPQK
ALESPERPFLAILGGAK
DCcamVGPEVENACcamANPAAGTVILLENLR
ELNYFAK
FHVEEEGKGK
GCcamITIIGGGDTATCcamCcamAK
ITLPVDFVTADKFDENAK
KYAEAVGR
LGDVYVNDAFGTAHR
LSSPATLNSR
NNQITNNQR
oxVVMRVDFNVPMK
QIVWNGPVGVFEWEAFAR
"SLLGKDVLFLK
SLLGKDVLFLK"
TGQATVASGIPAGWMGLDCcamGTESSKK
TGQATVASGIPAGWMoxGLDCcamGTESSKK
"VDFNVPMKNNQITNNQR
VDFNVPMKNNQITNNQR"
VDFNVPMoxKNNQITNNQR
VLNNMEIGTSLYDEEGAK
VLNNMoxEIGTSLYDEEGAK
VLPGVDALSNV
WNTEDKVSHVSTGGGASLELLEGK	
286	6PGL_MOUSE 6-phosphogluconolactonase (6PGL)	Q9CQ60 	27,3	5,4	230	71	15	AASCcamLEGDRGR
DLPAAAAPAGPASFAR
EKIVAPISDSPKPPPQR
FALGLSGGSLVSMLAR
FALGLSGGSLVSMoxLAR
ILEDKEGTLPAALVQPR
IVAPISDSPKPPPQR
IVAPISDSPKPPPQRVTLTLPVLNAAQSIIFVATGEGK
LLSVPFEKHSTL
LPIPDSQVLTINPALPVEDAAEDYAR
LVPFDHAESTYGLYR
TGALCcamWFLDEAAAR
THLLSKLPIPDSQVLTINPALPVEDAAEDYAR
VTLTLPVLNAAQSIIFVATGEGK
WTLGFCcamDER	
287	Phosphoglucomutase Mol_id: 1; Molecule: Alpha-D-Glucose-1,6-Bisphosphate; Chain: A, B; Synonym: Phosphoglucomutase; Ec: 5.4.2.2; Heterogen: Mg	P00949
	61,4	6,6	116	34	11	ADNFEYHDPVDGSVSKNQGLR
FKPFTVEIVDSVEAYATMoxLR
HGFFVNPSDSVAVIAANIFSIPYFQQTGVR
INQDPQVMoxLAPLISIALK
IRIDAMoxHGVVGPYVK
LSLCcamGEESFGTGSDHIR
LVIGQNGILSTPAVSCcamIIR
LVIGQNGILSTPAVSCcamIIRK
NIFDFNALKELLSGPNR
SGEHDFGAAFDGDGDRNMoxILGK
SMPTSGALDRVANATK	
288	Phosphoglucomutase 2; phosphoglucomutase 1 unnamed protein product [Mus musculus]	Q3UGE3
	61,4	6	221	54	20	ADNFEYSDPVDGSISKNQGLR
AIGGIILTASHNPGGPNGDFGIK
DLEALMoxLDR
FKPFTVEIVDSVEAYATMoxLR
FYMoxTEAIQLIVR
HGFFVNPSDSVAVIAANIFSIPYFQQTGVR
IDAMoxHGVVGPYVK
IDAMoxHGVVGPYVKK
INQDPQVMoxLAPLISIALK
IRIDAMoxHGVVGPYVK
LIFADGSRIIFR
LSLCcamGEESFGTGSDHIR
LVIGQNGILSTPAVSCcamIIR
LYIDSYEKDVAK
NFFTRYDYEEVEAEGANK
NIFDFNALKELLSGPNR
SGEHDFGAAFDGDGDRNMoxILGK
SMoxPTSGALDRVANATK
TQAYPDQKPGTSGLR
VYTVEKADNFEYSDPVDGSISK	
289	Prohibitin [Mus musculus]	P67778
	29,8	5,4	335	93	24	AAELIANSLATAGDGLIELR
AAIISAEGDSKAAELIANSLATAGDGLIELR
AATFGLILDDVSLTHLTFGK
AVIFDRFR
DLQNVNITLR
FDAGELITQR
FDAGELITQRELVSR
FGLALAVAGGVVNSALYNVDAGHR
FVVEKAEQQK
GVQDIVVGEGTHFLIPWVQKPIIFDCcamR
ILFRPVASQLPR
IYTSIGEDYDER
IYTSIGEDYDERVLPSITTEILK
KLEAAEDIAYQLSR
NITYLPAGQSVLLQLPQ
NVPVITGSKDLQNVNITLR
QVAQQEAER
QVAQQEAERAR
QVSDDLTER
SRNITYLPAGQSVLLQLPQ
SVVARFDAGELITQR
VFESIGKFGLALAVAGGVVNSALYNVDAGHR
VLPSITTEILK
VLPSITTEILKSVVAR	
290	Lrrc8 protein [Mus musculus]	Q80V68
	32,5	5,8	175	64	13	APSDSGPGTSFLGSDPAWASNLFVPLPVR
APSDSGPGTSFLGSDPAWASNLFVPLPVRR
FFFEKGVFDEK
GGLVLIHGEVVHKSEQNHSDHSR
GVFDEKGNFLVPPEK
IGEIVAEMDVPLHCcamR
IGEIVAEMoxDVPLHCcamR
QPHFGGEVSPHQDATFLYTEPLGR
SINKIGHALHAHDPVFR
SITHSPKVQALVR
SLGLQMoxPVVVQSMoxYIFK
TDYFLSSGDKIR
TEFSTQEDEQLQTQGK	
291	Phosphatidylinositol transfer protein alpha isoform	P53810
	31,8	6	109	43	11	AWNAYPYCcamR
DCcamPYMCcamAYKLVTVK
GPLGPNWKQELVNQK
HVEAIYIDIADR
HVEAIYIDIADRSQVLSK
IYHLQSKVPTFVR
MLAPEGALNIHEK
MoxLAPEGALNIHEK
RLFTNFHR
TGRGPLGPNWK
VILPVSVDEYQVGQLYSVAEASK	
292	Phosphomannomutase 2 (unnamed protein product) [Mus musculus]	Q9Z2M7
	27,7	5,8	99	44	13	GTFIEFRNGMLNVSPIGR
GTFIEFRNGMoxLNVSPIGR
HLEHAGYKTIYFFGDK
ITEEMoxDGFLQKLR
NGMLNVSPIGR
NGMoxLNVSPIGR
QKITEEMoxDGFLQK
TIYFFGDKTMoxPGGNDHEIFTDPR
TIYFFGDKTMPGGNDHEIFTDPR
TMoxPGGNDHEIFTDPR
TMPGGNDHEIFTDPR
TVGYTVTAPEDTR
TVGYTVTAPEDTRR	
293	Purine-nucleoside phosphorylase; purine-nucleoside phopshorylase [Mus musculus]	P23492
	32,2	5,6	231	77	21	AFSAWKQMoxGEQR
ANHMoxEVLDAGKAAAQTLER
DHINLPGFCcamGQNPLR
DHINLPGFCcamGQNPLRGPNDER
EAQIFDYNEIPNFPQSTVQGHAGR
FHMoxYEGYSLSK
FHMYEGYSLSK
FPAMoxSDAYDRDMoxR
FVSILMESIPLPDR
FVSILMESIPLPDRGS
FVSILMoxESIPLPDR
FVSILMoxESIPLPDRGS
HCcamGLRVFGFSLITNK
KLQEGTYVMLAGPNFETVAESR
KLQEGTYVMoxLAGPNFETVAESR
LQEGTYVMoxLAGPNFETVAESR
MLGADAVGMSTVPEVIVAR
MoxLGADAVGMoxSTVPEVIVAR
oxCcamCcamVMMQGR
oxFPAMSDAYDRDMR
oxMLGADAVGMSTVPEVIVAR
VFGFSLITNK
WLLQHTEYRPQVAVICcamGSGLGGLTAHLK	
294	Pyridoxine 5'-phosphate oxidase; pyridoxamine-phosphate oxidase [Mus musculus]	Q91XF0
	30,1	8,4	120	41	11	EAENYFHSRPK
ELDSNPFASLVFYWEPLNR
FFTNYESR
FFTNYESRK
GFGKDGFR
GKELDSNPFASLVFYWEPLNR
GLATGDSPLGPMoxTHHGEEDWVYER
LPEKEAENYFHSRPK
QSSVIPDREYLR
QVRVEGPVK
SSQIGAVVSR	
295	Pyrophosphatase [Mus musculus]	Q9D819
	32,6	5,3	177	75	22	AAPFTLEYR
AAPFTLEYRVFLK
AIVDALPPPCcamESACcamSLPTDVDKWFHQQK
DFAVDIIKSTHDYWK
DVFHMoxVVEVPR
DVFHMVVEVPR
GISCcamMNTTVSESPFK
GISCcamMNTTVSESPFKCcamDPDAAK
GISCcamMoxNTTVSESPFK
GISCcamMoxNTTVSESPFKCcamDPDAAK
GQYISPFHDVPIYADKDVFHMoxVVEVPR
GQYISPFHDVPIYADKDVFHMVVEVPR
HTGCcamCcamGDNDPIDVCcamEIGSK
LKPGYLEATVDWFR
LKPGYLEATVDWFRR
NKDFAVDIIK
VCcamARGEIIR
VIAINVDDPDAANYKDISDVER
VKVLGILAMoxIDEGETDWK
VLGILAMIDEGETDWK
VLGILAMoxIDEGETDWK
VPDGKPENEFAFNAEFK
YKVPDGKPENEFAFNAEFK
YVANLFPYK	
296	Inorganic pyrophosphatase 2 [Mus musculus]	Q91VM9
	38,1	6,6	180	62	19	AFALDVINSAHER
ARNDEYENLFNMoxVVEIPR
ARNDEYENLFNMVVEIPR
ESNVEEEVWHFLRN
FAFNGEFKNK
FKPGYLEATLNWFR
GAISCcamVNVHICcamDSPFHCcamTMoxEEAR
HVAGHYISPFHDIPLK
HVAGHYISPFHDIPLKADCcamK
ILGTLALIDQSETDWK
LYKVPDGKPENK
MEIATEEPLNPIKQDIK
MoxEIATEEPLNPIKQDIK
NDEYENLFNMoxVVEIPR
NDEYENLFNMVVEIPR
NKAFALDVINSAHER
SQDYRLFFK
STDCcamCcamGDNDPIDVCcamEIGSK
VLSRGDVVHVK
YTPNIFPHK	
297	Peptidylprolyl isomerase A (Unknown) (protein for MGC:117694) [Mus musculus]	P17742
	18	7,5	158	93	20	ALSTGEKGFGYK
EGMNIVEAMER
EGMoxNIVEAMoxER
GFGYKGSSFHR
HTGPGILSMANAGPNTNGSQFFICcamTAK
HTGPGILSMoxANAGPNTNGSQFFICcamTAK
IIPGFMCcamQGGDFTR
IIPGFMCcamQGGDFTRHNGTGGR
IIPGFMoxCcamQGGDFTR
IIPGFMoxCcamQGGDFTRHNGTGGR
KITISDCcamGQL
oxEGMNIVEAMER
oxVKEGMNIVEAMER
SIYGEKFEDENFILK
TAENFRALSTGEK
TEWLDGKHVVFGK
VKEGMNIVEAMER
VKEGMoxNIVEAMoxER
VNPTVFFDITADDEPLGR
VSFELFADKVPK	
298	Peptidylprolyl isomerase B; cyclophilin B [Mus musculus]	P24369
	22,7	9,5	147	62	16	DFMIQGGDFTR
DTNGSQFFITTVK
FPDENFK
FPDENFKLK
GDGTGGKSIYGER
HYGPGWVSMoxANAGK
IEVEKPFAIAKE
LKHYGPGWVSMANAGK
SIYGERFPDENFK
TSWLDGKHVVFGK
TVDNFVALATGEK
TVDNFVALATGEKGFGYK
VIKDFMIQGGDFTR
VIKDFMoxIQGGDFTR
VLEGMDVVR
VLEGMoxDVVR
VYFDLQIGDESVGR	
299	Serine/threonine-protein phosphatase 2A 65 kDa regulatory subunit A alpha isoform; alpha isoform of regulatory subunit A, protein phosphatase 2 (Pp2a, Pr65alpha)	Q76MZ3
	65,3	5	199	38	23	AISHEHSPSDLEAHFVPLVKR
IGPILDNSTLQSEVKPILEK
KLSTIALALGVER
LAGGDWFTSR
LNIISNLDCcamVNEVIGIR
LSTIALALGVER
LTQDQDVDVKYFAQEALTVLSLA
MAGDPVANVRFNVAK
MoxAGDPVANVRFNVAK
NLCcamSDDTPMoxVR
NLCcamSDDTPMoxVRR
NLCcamSDDTPMVR
NLCcamSDDTPMVRR
QAAEDKSWR
RLAGGDWFTSR
SALASVIMoxGLSPILGK
TDLVPAFQNLMoxKDCcamEAEVR
TSACcamGLFSVCcamYPR
VKEFCcamENLSADCcamR
VLAMoxSGDPNYLHR
VLAMSGDPNYLHR	
300	Peroxiredoxin 1 [Mus musculus]	P35700
	22,2	8,6	179	80	19	ATAVMoxPDGQFK
ATAVMoxPDGQFKDISLSEYK
ATAVMPDGQFK
ATAVMPDGQFKDISLSEYK
GLFIIDDKGILR
HGEVCcamPAGWKPGSDTIKPDVNK
KQGGLGPMNIPLISDPK
KQGGLGPMoxNIPLISDPK
LNCcamQVIGASVDSHFCcamHLAWINTPK
LNCcamQVIGASVDSHFCcamHLAWINTPKK
LVQAFQFTDK
QGGLGPMNIPLISDPK
QGGLGPMNIPLISDPKR
QGGLGPMoxNIPLISDPK
QGGLGPMoxNIPLISDPKR
QITINDLPVGR
TIAQDYGVLK
TIAQDYGVLKADEGISFR
YVVFFFYPLDFTFVCcamPTEIIAFSDR	
301	Peroxiredoxin 3 [Mus musculus]	P20108
	28,1	6,9	145	45	11	AFQFVETHGEVCcamPANWTPESPTIKPSPTASK
DYGVLLESAGIALR
EYFEKVHQ
GLFIIDPNGVVK
GLFIIDPNGVVKHLSVNDLPVGR
GTAVVNGEFKELSLDDFK
HLSVNDLPVGR
KNGGLGHMNITLLSDITK
KNGGLGHMoxNITLLSDITK
NGGLGHMNITLLSDITK
NGGLGHMoxNITLLSDITK
QISRDYGVLLESAGIALR
SVEETLRLVK	
302	Peroxiredoxin 4; antioxidant enzyme AOE372; Prx IV [Mus musculus]	O08807
	31,1	6,4	150	67	12	DYGVYLEDSGHTLR
ENECcamHFYAGGQVYPGEASR
GLFIIDDKGVLR
ISKPAPYWEGTAVINGEFKELK
LVQAFQYTDKHGEVCcamPAGWKPGSETIIPDPAGK
QGGLGPIRIPLLSDLNHQISK
QITLNDLPVGR
SINTEVVACcamSVDSQFTHLAWINTPR
TESLQGLESDER
TRENECcamHFYAGGQVYPGEASR
VSVADHSLHLSK
VSVADHSLHLSKAK	
303	Peroxiredoxin 5 [Mus musculus]	P99029
	17	7,4	261	93	17	ALNVEPDGTGLTCcamSLAPNILSQL
APIKVGDAIPSVEVFEGEPGK
ATDLLLDDSLVSLFGNR
ATDLLLDDSLVSLFGNRR
FSMoxVIDNGIVK
FSMVIDNGIVK
GAQVVACcamLSVNDVFVIEEWGR
GVLFGVPGAFTPGCcamSK
KGVLFGVPGAFTPGCcamSK
KVNLAELFK
LLADPTGAFGK
LLADPTGAFGKATDLLLDDSLVSLFGNR
RFSMoxVIDNGIVK
THLPGFVEQAGALK
THLPGFVEQAGALKAK
VGDAIPSVEVFEGEPGK
VGDAIPSVEVFEGEPGKK
VRLLADPTGAFGK	
304	Peroxiredoxin 6; 1-Cys peroxiredoxin; thiol-specific antioxidant protein [Rattus norvegicus]	O08709
	24,8	5,5	126	63	10	DFTPVCcamTTELGR
DINAYNGAAPTEK
FHDFLGDSWGILFSHPR
KGESVMoxVLPTLPEEEAK
LIALSIDSVEDHFAWSK
LKLSILYPATTGR
LSILYPATTGR
PGGLLLGDEAPNFEANTTIGHIR
VVDSLQLTASNPVATPVDWK
VVFIFGPDKK	
305	Peroxiredoxin 6; acidic calcium-independent phospholipase A2; peroxiredoxin 5; 1-Cys Prx; anti-oxidant protein 2 [Mus musculus]	O35244
	24,8	5,7	282	93	24	DANNMoxPVTAR
DFTPVCcamTTELGR
DINAYNGETPTEK
DLAILLGMoxLDPVEK
DLAILLGMoxLDPVEKDANNMoxPVTAR
FHDFLGDSWGILFSHPR
GESVMoxVVPTLSEEEAKQCcamFPK
GRDLAILLGMoxLDPVEK
GVFTKELPSGK
IRFHDFLGDSWGILFSHPR
KGESVMoxVVPTLSEEEAK
KGESVMVVPTLSEEEAK
LIALSIDSVEDHLAWSK
LKLSILYPATTGR
LPFPIIDDK
LPFPIIDDKGR
LSILYPATTGR
LSILYPATTGRNFDEILR
PGGLLLGDEAPNFEANTTIGR
VVDSLQLTGTKPVATPVDWK
VVDSLQLTGTKPVATPVDWKK
VVFIFGPDK
VVFIFGPDKK
YLRYTPQP	
306	Proteasome (prosome, macropain) subunit, alpha type 1 [Mus musculus]	Q9R1P4
	29,5	5,9	205	83	19	ALRETLPAEQDLTTK
AQPSQAAEEPAEKADEPMoxEH
DLEFTIYDDDDVSPFLDGLEERPQRK
FVFDRPLPVSR
HMoxSEFMoxECcamNLDELVK
HMSEFMECcamNLDELVK
IHQIEYAMEAVK
IHQIEYAMoxEAVK
ILHVDNHIGISIAGLTADAR
KILHVDNHIGISIAGLTADAR
LLCcamNFMoxRQECcamLDSR
LLCcamNFMRQECcamLDSR
LVSLIGSKTQIPTQR
NQYDNDVTVWSPQGR
NVSIGIVGKDLEFTIYDDDDVSPFLDGLEERPQR
oxHMSEFMECcamNLDELVK
oxHMSEFMECcamNLDELVKHGLR
RPYGVGLLIAGYDDMoxGPHIFQTCcamPSANYFDCcamR
THAVLVALKR	
307	Proteasome (prosome, macropain) subunit, alpha type 2 [Mus musculus]	P49722
	25,9	6,5	121	47	10	GYSFSLTTFSPSGK
GYSFSLTTFSPSGKLVQIEYALAAVAGGAPSVGIK
HIGLVYSGMGPDYR
HIGLVYSGMoxGPDYR
HIGLVYSGMoxGPDYRVLVHR
KLAQQYYLVYQEPIPTAQLVQR
LAQQYYLVYQEPIPTAQLVQR
LTPTEVRDYLAAIA
LVQIEYALAAVAGGAPSVGIK
YNEDLELEDAIHTAILTLK	
308	Proteasome (prosome, macropain) subunit, alpha type 6 [Mus musculus]	Q9QUM9
	27,4	6	200	56	16	AINQGGLTSVAVR
ARYEAANWK
ATAAGVKQTESTSFLEK
CcamDPAGYYCcamGFK
GSSAGFDRHITIFSPEGR
HITIFSPEGR
ILTEAEIDAHLVALAERD
ITESIGCcamVMoxTGMoxTADSR
LLDSSTVTHLFK
LYQVEYAFK
LYQVEYAFKAINQGGLTSVAVR
oxITESIGCcamVMTGMTADSR
YGYEIPVDMLCcamKR
YGYEIPVDMoxLCcamK
YGYEIPVDMoxLCcamKR
YKYGYEIPVDMoxLCcamK	
309	Proteasome (prosome, macropain) subunit, beta type 3 (Unknown) (protein for MGC:105457) [Rattus norvegicus]	Q9R1P1
	23	5,7	147	56	13	FGIQAQMoxVTTDFQK
FGIQAQMVTTDFQK
FGPYYTEPVIAGLDPK
FRLNLYELK
IFPMoxGDRLYIGLAGLATDVQTVAQR
LNLYELKEGR
LYIGLAGLATDVQTVAQR
LYIGLAGLATDVQTVAQRLK
NCcamVAIAADRR
oxSVMSYNGGAVMAMKGK
QIKPYTLMoxSMoxVANLLYEK
QIKPYTLMoxSMoxVANLLYEKR
RFGPYYTEPVIAGLDPK	
310	Proteasome beta 4 subunit; proteasome (prosome, macropain) subunit, beta type, 4 [Mus musculus]	P99026
	29,1	5,3	143	55	14	EVLEKQPVLSQTEAR
FDGGVVIAADMLGSYGSLAR
FDGGVVIAADMoxLGSYGSLAR
GVEIEGPLSAQTNWDIAHMISGFE
GVEIEGPLSAQTNWDIAHMoxISGFE
IMoxRVNDSTMoxLGASGDYADFQYLK
MEAFWESR
QPVLSQTEAR
QPVLSQTEARELVER
QVLGQMoxVIDEELLGDGHSYSPR
QVLGQMVIDEELLGDGHSYSPR
SYNRFQIATVTEK
TQNPMoxVTGTSVLGVK
VNDSTMLGASGDYADFQYLK	
311	Psmb7 protein [Mus musculus]	P70195
	29,8	8,1	130	30	9	FRPDMoxEEEEAK
FRPDMoxEEEEAKK
KLVSEAIAAGIFNDLGSGSNIDLCcamVISK
LDFLRPFSVPNK
LDFLRPFSVPNKK
LPYVTMGSGSLAAMAVFEDK
LVSEAIAAGIFNDLGSGSNIDLCcamVISK
MoxLKQMoxLFR
SKLDFLRPFSVPNK	
312	Proteasome 26S ATPase subunit 1 [Homo sapiens]	P62191
	49,2	5,8	112	30	12	AVANQTSATFLR
EIQRTMLELLNQLDGFDSR
GPDAASKLPLVTPHTQCcamR
IETLDPALIRPGR
KQEGTPEGLYL
KYEPPVPTR
LPLVTPHTQCcamR
TMoxLELLNQLDGFDSRGDVK
VAEEHAPSIVFIDEIDAIGTK
VAEEHAPSIVFIDEIDAIGTKR
VVGSELIQKYLGDGPK
YDSNSGGEREIQR	
313	Proteasome 26S ATPase subunit 2; proteasome 26S subunit, ATPase, 2; mammalian suppressor of sgv-1 of yeast; protease 26S subunit 7 [Homo sapiens]	P35998
	48,6	5,6	255	67	36	ACcamLIFFDEIDAIGGAR
ALDEGDIALLK
ALDEGDIALLKTYGQSTYSR
AVANRTDACcamFIR
DIRFELLAR
EVVETPLLHPER
FVNLGIEPPKGVLLFGPPGTGK
FVVDLSDQVAPTDIEEGMoxR
FVVDLSDQVAPTDIEEGMR
IATEKDFLEAVNK
IEFSLPDLEGR
IINADSEDPKYIINVK
KACcamLIFFDEIDAIGGAR
KIEFSLPDLEGR
LCcamPNSTGAEIR
LREVVETPLLHPER
MoxVRELFEMoxAR
NKYQIHIPLPPK
PDYLGADQRK
QIKQVEDDIQQLLK
QVEDDIQQLLKK
SVCcamTEAGMFAIR
SVCcamTEAGMoxFAIR
THIFKIHAR
TMLELINQLDGFDPR
TMoxLELINQLDGFDPR
TYGQSTYSR
TYGQSTYSRQIK
VIGSELVQKYVGEGAR
VLMoxATNRPDTLDPALMoxRPGRLDR	
314	PSMC3 [Homo sapiens]	P17980
	45,2	5,3	291	77	38	AMoxEVDERPTEQYSDIGGLDK
APSIIFIDELDAIGTK
APSIIFIDELDAIGTKR
ARIMoxQIHSR
AVCcamVEAGMIALR
AVCcamVEAGMIALRR
AVCcamVEAGMoxIALR
AVCcamVEAGMoxIALRR
DSYLILETLPTEYDSR
EKAPSIIFIDELDAIGTK
EKFENLGIQPPK
FENLGIQPPK
GATELTHEDYMEGILEVQAK
GATELTHEDYMoxEGILEVQAK
GVLMoxYGPPGTGK
IEFPMoxPNEEAR
KIEFPMoxPNEEAR
KIEFPMPNEEAR
KMNVSPDVNYEELAR
KMoxNVSPDVNYEELAR
LAGPQLVQMFIGDGAK
LAGPQLVQMoxFIGDGAK
LKPGDLVGVNKDSYLILETLPTEYDSR
LLDSEIKIMoxK
MSTEEIIQRTR
QIQELVEAIVLPMoxNHK
QTYFLPVIGLVDAEK
RGATELTHEDYMoxEGILEVQAK
TLPYLVSNVIELLDVDPNDQEEDGANIDLDSQR
TLPYLVSNVIELLDVDPNDQEEDGANIDLDSQRK
TMLELLNQLDGFQPNTQVK
TMoxLELLNQLDGFQPNTQVK
VDILDPALLR
VDILDPALLRSGR
VIAATNRVDILDPALLR
VKAMoxEVDERPTEQYSDIGGLDK
VTHELQAMKDK
VTHELQAMoxKDK	
315	Proteasome 26S ATPase subunit 4 [Rattus norvegicus]	Q63570
	47,4	5,1	268	71	23	ADTLDPALLRPGR
ADTLDPALLRPGRLDR
AVAHHTTAAFIR
EAVELPLTHFELYK
EFLHAQEEVKR
ENAPAIIFIDEIDAIATK
ENAPAIIFIDEIDAIATKR
FDAQTGADREVQR
GVLMoxYGPPGCcamGK
IEFPLPDRR
ILLELLNQMDGFDQNVNVK
ILSTIDRELLKPNASVALHK
IQDEIPALSVSRPQTGLSFLGPEPEDLEDLYSR
IQSIPLVIGQFLEAVDQNTAIVGSTTGSNYYVR
ISGADINSICcamQESGMLAVR
ISGADINSICcamQESGMoxLAVR
KDEQEHEFYK
LAKENAPAIIFIDEIDAIATK
LQQELEFLEVQEEYIKDEQK
MNLSEEVDLEDYVARPDK
MoxNLSEEVDLEDYVARPDK
QIGIDPPRGVLMoxYGPPGCcamGK
YIVLAKDFEK	
316	Similar to proteasome 26S ATPase subunit 6 [Rattus norvegicus]	P62334
	45,8	7,5	260	71	35	ADHDFVVQEDFMK
ADHDFVVQEDFMoxK
ALPGIPYERR
ALQSVGQIVGEVLK
ALQSVGQIVGEVLKQLTEEK
AVASQLDCcamNFLK
DHQPCcamIIFMDEIDAIGGR
DHQPCcamIIFMDEIDAIGGRR
DHQPCcamIIFMoxDEIDAIGGR
DHQPCcamIIFMoxDEIDAIGGRR
ELREVIELPLTNPELFQR
EVDPLVYNMSHEDPGNVSYSEIGGLSEQIR
EVIELPLTNPELFQR
FSEGTSADREIQR
GCcamLLYGPPGTGK
HGEIDYEAIVK
IHIDLPNEQAR
KIHIDLPNEQAR
LIREMoxFNYAR
LLEHKEIDGR
LSDGFNGADLR
LSDGFNGADLRNVCcamTEAGMFAIR
MALPGIPYER
NVCcamTEAGMFAIR
NVCcamTEAGMoxFAIR
oxTLMELLNQMDGFDTLHR
QLTEEKFIVK
SENDLKALQSVGQIVGEVLK
TLLARAVASQLDCcamNFLK
TLMELLNQMDGFDTLHR
TLMoxELLNQMoxDGFDTLHR
VALDMoxTTLTIMoxR
VALDMTTLTIMRYLPR
VGIIPPKGCcamLLYGPPGTGK
VVSSSIVDKYIGESAR	
317	Psmd9 (Unknown (protein for MGC:66876)) [Mus musculus]	Q9CR00
	24,7	5,8	148	51	12	AAAVSDIQDLMoxR
ADVDLYQVR
ALMoxKQVEEALHQLHAR
DMoxAEAREEAMoxNR
GIGMoxNEPLVDCcamEGYPR
GIGMoxNEPLVDCcamEGYPRADVDLYQVR
GLLGCcamNIIPLQR
HNIICcamLQNDHKALMoxK
LASNSPVLPQAFAR
LIPTRWAGK
QVEEALHQLHAR
RLASNSPVLPQAFAR	
318	Proteasome (prosome, macropain) 28 subunit, alpha; protease (prosome, macropain) 28 subunit, alpha; PA28 alpha subunit [Mus musculus]	P97371
	28,7	5,6	148	75	18	AAKQPHVGDYR
APLDIPVPDPVKEK
IEDGNNFGVAVQEK
ISELDAFLKEPALNEANLSNLK
IVVLLQR
KISELDAFLK
LEGFHTQISK
LKPEIKDVTEQLNLVTTWLQLQIPR
LMoxVMoxEIR
NAYAVLYDIILK
NAYAVLYDIILKNFEK
QLVHELDEAEYQEIR
QPHVGDYR
TENLLGSYFPK
VDVFREDLCcamSK
VFELMoxTNLHTK
VFELMoxTNLHTKLEGFHTQISK
YFSERGDAVAK	
319	Proteasome activator subunit 2; protease (prosome, macropain) 28 subunit, beta [Mus musculus]	P97372
	27,1	5,4	210	75	19	AFYAELYHIISSNLEK
AFYAELYHIISSNLEKIVNPK
ALVHERDEAAYGALR
AMoxVLDLRAFYAELYHIISSNLEK
ASKDTHVMDYR
ASKDTHVMoxDYR
CcamGYLPGNEK
DTHVMoxDYR
EVPKCcamGYLPGNEK
IEDGNDFGVAIQEK
IEDGNDFGVAIQEKVLER
IISLSQLLQEDSLNVADLSSLR
IVNPKGEEKPSMoxY
KIISLSQLLQEDSLNVADLSSLR
LLALLALVKPEVWTLKEK
QNLFQEADDFLCcamTFLPR
QVDVFRQNLFQEADDFLCcamTFLPR
TKVEAFQTTISK
YFSERGDAVAK	
320	Protease (prosome, macropain) 28 subunit, beta, b; PA28 beta subunit (PA28b) pseudogene [Mus musculus]	P97372
	27,1	5,4	210	75	18	AFYAELYHIISSNLEK
AFYAELYHIISSNLEKIVNPK
ALVHERDEAAYGALR
AMoxVLDLRAFYAELYHIISSNLEK
ASKDTHVMDYR
ASKDTHVMoxDYR
CcamGYLPGNEK
DTHVMoxDYR
EVPKCcamGYLPGNEK
IEDGNDFGVAIQEK
IEDGNDFGVAIQEKVLER
IISLSQLLQEDSLNVADLSSLR
IVNPKGEEKPSMoxY
KIISLSQLLQEDSLNVADLSSLR
LLALLALVKPEVWTLKEK
QNLFQEADDFLCcamTFLPR
QVDVFRQNLFQEADDFLCcamTFLPR
TKVEAFQTTISK
YFSERGDAVAK	
321	Similar to TRANSCRIPTIONAL ACTIVATOR PROTEIN PUR-ALPHA (PURINE-RICH SINGLE-STRANDED DNA-BINDING PROTEIN ALPHA) [Rattus norvegicus]	P42669
	34,9	6	105	54	14	AACcamEQLHQQQQQQQEETTAATLLLQGEEEGEED
FFFDVGSNKYGVFMoxR
FFFDVGSNKYGVFMR
FLKIAEVGAGGNK
FYLDVKQNAK
GPGLGSTQGQTIALPAQGLIEFR
GPGLGSTQGQTIALPAQGLIEFRDALAK
LIDDYGVEEEPAELPEGTSLTVDNKR
LTLSMoxSVAVEFR
NSITVPYKVWAK
QTVNRGPGLGSTQGQTIALPAQGLIEFR
VSEVKPTYR
YYMDLKENQR
YYMoxDLKENQR	
322	Pzp protein [Mus musculus]	Q61838
	165,9	6,2	151	12	19	APFALQVNTLPLNFDK
APFALQVNTLPLNFDKAGDHR
GSGSGCcamVYLQTSLK
GSGSGCcamVYLQTSLKYNILPVADGK
KLQDQPNIQR
LLLQEVRLPDLPGNYVTK
LPDLPGNYVTK
LQDQPNIQR
LTNQTLGFSFAVEQDIPVK
MoxVSGFIPMoxKPSVKK
QQNSHGGFSSTQDTVVALQALSK
SQKEVLVTIESSGTFSK
TEVNTNHVLIYIEK
TFAPEEISAMoxVLTK
TFHVNSGNR
TFQIRINVSYTGERPSSNMVIVDVK	
323	Quininoid dihydropteridine reductase [Mus musculus]	Q8BVI4
	25,6	7,4	102	66	12	AALDGTPGMoxIGYGMoxAK
GAVHQLCcamQSLAGK
HLKEGGLLTLAGAK
LLGDQKVDAILCcamVAGGWAGGNAK
MoxTDSFTEQADQVTADVGK
NSGMoxPPGAAAIAVLPVTLDTPMoxNR
NSGMoxPPGAAAIAVLPVTLDTPMoxNRK
oxAALDGTPGMIGYGMAK
QSMoxWTSTISSHLATK
RPNSGSLIQVVTTDGKTELTPAYF
SLFKNCcamDMoxMoxWK
VDAILCcamVAGGWAGGNAK	
324	Quinolinate phosphoribosyltransferase; nicotinate-nucleotide pyrophosphorylase [Mus musculus]	Q91X91
	31,5	6,1	168	53	16	AAEAGADLVMLDNFKPEELHPTAATLK
AAEAGADLVMoxLDNFKPEELHPTAATLK
CcamSGIASAAATAVEVAR
DNHVVAAGSMoxER
GPAHHLLLGER
KTTPGFR
LFAEGDTPVPHAR
LVEKYGLQVGGAACcamHR
QAAGFSLKVEVECcamSSLEEAFR
STGWTGHVAGTR
STGWTGHVAGTRK
VAEVKGPAHHLLLGER
VALNTLAR
VEVECcamSSLEEAFR
YDLGGMoxVMoxVK
YGLQVGGAACcamHR	
325	Ranbp1 protein [Mus musculus]	P34022
	23,6	5,1	84	45	8	AWVWNTHADFADECcamPKPELLAIR
DSHEDHDTSTENADESNHDPQFEPIVSLPEQEIK
FASENDLPEWKER
FLNAENAQKFK
ICcamANHYITPMoxMoxELKPNAGSDR
oxICcamANHYITPMMELKPNAGSDR
TLEEDEEELFKMoxR
VAEKLEALSVR	
326	Retinol binding protein 4, plasma; retinol binding protein 4, cellular [Mus musculus]	Q00724
	23,2	5,6	144	62	14	ARFSGLWYAIAK
DPNGLSPETR
DPNGLSPETRR
ENFDKAR
FSGLWYAIAK
GNDDHWIIDTDYDTFALQYSCcamR
KDPEGLFLQDNIIAEFSVDEK
LQNLDGTCcamADSYSFVFSR
MKYWGVASFLQR
MoxKYWGVASFLQR
QEELCcamLER
QRQEELCcamLER
WIEHNGYCcamQSRPSR
YWGVASFLQR	
327	Regucalcin [Mus musculus]	Q64374
	33,4	5,1	253	85	25	CcamGESPVWEEASQSLLFVDIPSK
CcamGESPVWEEASQSLLFVDIPSKIICcamR
DGLNAEGLLR
DGLNAEGLLRQPDAGNIFK
DYSEMoxYVTCcamAR
DYSEMYVTCcamAR
FCcamALNWENQSVFVLAMoxVDEDKK
FNDGKVDPAGR
HQGSLYSLFPDHSVK
HQGSLYSLFPDHSVKK
IFYYIDSLSYTVDAFDYDLQTGQISNR
IFYYIDSLSYTVDAFDYDLQTGQISNRR
KYFDQVDISNGLDWSLDHK
LPVDKTTSCcamCcamFGGK
LWVACcamYNGGR
MEKDEQIPDGMCcamIDAEGK
QLGGYVATIGTK
TTSCcamCcamFGGKDYSEMoxYVTCcamAR
TTSCcamCcamFGGKDYSEMYVTCcamAR
VAVDAPVSSVALR
VECcamVLRENYR
WDTVSNQVQR
YFAGTMAEETAPAVLER
YFAGTMoxAEETAPAVLER
YFDQVDISNGLDWSLDHK	
328	Rpl11 protein [Mus musculus]	Q9CXW4
	20,2	10,3	105	47	10	GAKAEEILEK
KLCcamLNICcamVGESGDR
LCcamLNICcamVGESGDRLTR
NEKIAVHCcamTVR
VLEQLTGQTPVFSK
VLEQLTGQTPVFSKAR
WFQQKYDGIILPGK	
329	Ribosomal protein S5, cytosolic - human	P46782
	22,8	9,6	117	50	13	KAQCcamPIVER
LTNSMMMHGR
LTNSMoxMoxMoxHGR
NIKTIAECcamLADELINAAK
oxLTNSMMMHGR
QAVDVSPLRR
RVNQAIWLLCcamTGAR
TIAECcamLADELINAAK
TIAECcamLADELINAAKGSSNSYAIK
VNQAIWLLCcamTGAR
WSTDDVQINDISLQDYIAVK
WSTDDVQINDISLQDYIAVKEK
YAKYLPHSAGR	
330	Protein 40kD (34/67 kDa laminin receptor)	Q91V31
	32,8	4,7	130	48	13	ADHQPLTEASYVNLPTIALCcamNTDSPLR
AIVAIENPADVSVISSR
AVLKFAAATGATPIAGR
FAAATGATPIAGR
FTPGTFTNQIQAAFR
FTPGTFTNQIQAAFREPR
GAHSVGLMoxWWMoxLAR
KSDGIYIINLK
oxGAHSVGLMWWMLAR
oxMSGALDVLQMKEEDVLK
SDGIYIINLKR
TWEKLLLAAR
YVDIAIPCcamNNK	
331	Ribosome-binding protein 1 [Mus musculus]	Q99PL5
	97	5,7	224	41	28	AAIKLQELLK
AENSQLTER
AENSQLTERIR
AMEALALAER
CcamEELSSLHGQLKEAR
EAEETQNSLQAECcamDQYR
EHTSHLEAELEKHMoxAAASAECcamQNYAK
EQEIAAVQAR
IRSIEALLEAGQAQDTQASHAEANQQQTR
IRTLQEQLENGPNTQLAR
KPPTLEPSMoxDIVLK
LEEVTRELCcamR
LIEILSEKTGVIQDTWHK
LQQENSILR
LQQENSILRDALNQATSQVESK
LREAEETQNSLQAECcamDQYR
LTAEFEEAQR
NWKAMoxEALALAER
QKLTAEFEEAQR
QLLLESQSQLDEAKSEAQK
QLVAREQEIAAVQAR
QSDELALVR
QVLQLQASHKESEEALQK
SHVEDGDVAGSPAVPPAEQDPMoxKLK
SIEALLEAGQAQDTQASHAEANQQQTR
SVEEEERVWK
TEATLEAEQTRR
TGVIQDTWHK
TILAETEGMoxLKDLQK
TLQEQLENGPNTQLAR
VAELHSKLQSSEVEVK
VGAAEEELHKSR	
332	Sarcosine dehydrogenase [Mus musculus]	Q99LB7
	101,7	6,2	307	55	34	AIDSLSIEKGYR
AYGIESHVLSPAETK
CcamQLMoxDSSEDLGMoxLSIQGPASR
DILQDVLDADLSNEAFPFSTHQLVR
DLEEETGLHTGWIQNGGLFIASNQQR
DMoxYSYDIRR
DPLHEELLGQGCcamVFQER
FHHSLTDHTR
FYLLGVDAR
GAAAVFNMoxSYFGK
GAAAVFNMoxSYFGKFYLLGVDAR
GAQVIENCcamAVTGIR
GGTESDLTVSR
HGLVNAGYRAIDSLSIEK
HWHADLRPDDSPLEAGLAFTCcamK
IEGIQNMoxPNVRDHDASVYLR
KAADWLFSADVNRPPGSTVYTCcamMoxLNQR
LGVGGAVLLER
LGVGGAVLLERER
LKTSVPFLGR
LLGDEYTFDFPPHHHMoxIQK
LLGDEYTFDFPPHHHMoxIQKECcamLACcamR
LSFVGELGWELHVPR
MoxAGVKVPLVAMoxHHAYVVTER
MoxGVTYAAQVHLK
NYSVVFPHDEPLAGR
oxCcamQLMDSSEDLGMLSIQGPASR
RADFGFTVNK
RDPLHEELLGQGCcamVFQER
RFHHSLTDHTR
SHESYAKNYSVVFPHDEPLAGR
SLYPLMoxNVDDLYGTLYVPQDGTMoxDPAGTCcamTTLTR
STVCcamGPESFTPDHKPLMoxGEAPELR
TIAYGYIRDPSGGPVSLDFVK
TSVPFLGREALEK
VAAVETEHGSIQTPCcamVVNCcamAGVWASK
VRTDDFGVR	
333	Selenocysteine lyase [Mus musculus]	Q9JLI6
	47,2	6,2	186	57	15	CcamFHEQQTLK
DIRDYLEER
EAWGNPSSSYVSGR
GTTRADVDLIVQDLK
GVGKLTPLYPMoxLFGGGQER
LPLEHLVENQMoxAEVTFVPVSK
LPNTCcamNFSIQGSQLQGYTVLAQCcamR
LTPLYPMoxLFGGGQER
MoxIGGKPQDIIFTSGGTESNNLVIHSMoxVR
NFRPGTENTPMoxIAGLGK
NGALGSVESLPDRK
QAVAQLEGRL
RVDVEDLGVDFLTIVGHK
TLLASVGASCcamHSNHEDRPSPVLLSCcamGIPVDVAR
VLVHTDAAQALGKR	
334	Sterol carrier protein x - mouse	P32020
	59,2	7	185	48	21	ALEDAQIPYSAVEQACcamVGYVYGDSTSGQR
ANLVFKEIEK
AVEIVAQEMoxMoxTDLPSTFEEK
FMKPGGENSR
FMoxKPGGENSRDYPDMoxAK
GDNTYGGKWVINPSGGLISK
GHPLGATGLAQCcamAELCcamWQLR
HSVNNTYSQFQDEYSLEEVMoxK
IAGNMoxGLAMoxKLQNLQLQPGK
LEEEGEQFVKK
LQNLQLQPGK
MGFPEAASSFR
MNPQSAFFQGK
NGKGSVLPNSDK
oxAVEIVAQEMMTDLPSTFEEK
oxFMKPGGENSRDYPDMAK
RVFVVGVGMoxTK
RVFVVGVGMTK
SIIKVVGYDMoxSK
THQVSAAPTSSAGDGFK
VALQHNLGLGGAVVVTLYR
WVINPSGGLISK	
335	secernin 2 [Mus musculus]	Q8VCA8
	46,6	5,4	216	59	21	DEVQEVVFIPAGTHVPGSR
DKESGICcamMDSGGFR
DKESGICcamMoxDSGGFR
EPVGQGEALLGMDLLR
FRAGCcamEMLQR
FRAGCcamEMoxLQR
GHQAALGLMEDEQEQAQQLR
GHQAALGLMoxEDEQEQAQQLR
GLLTGEQTPPAQGLGSLFQAFVER
GLLTGEQTPPAQGLGSLFQAFVEREEQAYA
HQGNITAEVMMGILR
HQGNITAEVMoxMoxGILR
LEQEGLEALR
LQCcamTYIEVEQVGK
NISNQLSIGTDISAEHPELR
NSDRPRDEVQEVVFIPAGTHVPGSR
oxHQGNITAEVMMGILR
QQRLEQEGLEALR
SSTAQEAVHVIAGLLDR
SVFKPFIFEVGVSQSPQVLSPTFGAQDPVR
TTASMoxVSVLPQDPTKPCcamVHFLTATPDPSR	
336	GTP-specific succinyl-CoA synthetase beta subunit [Mus musculus]	Q9Z2I8
	43,9	5,9	230	69	27	DIFAMDDKSENEPIENEAAR
DIFAMoxDDKSENEPIENEAAR
EAQVYEAFK
EIVLKAQILAGGR
ELELKVPLVVR
EQIDIFEGIKDSQAQR
ETYLAILMDR
FFVANTAKEALEAAK
GVFNSGLKGGVHLTK
IDATQVEVNPFGETPEGQVVCcamFDAK
INFDDNAEFR
INFDDNAEFRQK
KLMoxSEHGVR
LEGTNVQEAQNILK
LLTSDPKVEAILVNIFGGIVNCcamAIIANGITK
MAENLGFLGSLK
MoxAENLGFLGSLK
MoxAENLGFLGSLKNQAADQITK
NQAADQITKLYHLFLK
SSGLPITSAVDLEDAAK
SSGLPITSAVDLEDAAKK
VMoxVAEALDISRETYLAILMoxDR
VNKVMoxVAEALDISR
VPLVVRLEGTNVQEAQNILK
VVGELAQQMIGYNLATK
VVGELAQQMoxIGYNLATK	
337	Sdha protein [Mus musculus]	Q8K2B3
	72,3	7,2	283	49	28	AAFGLSEAGFNTACcamLTK
ACcamALSIAESCcamRPGDKVPSIK
AFGGQSLKFGK
ANAGEESVMNLDKLR
ANAGEESVMoxNLDKLR
CcamCcamCcamVADRTGHSLLHTLYGR
FADGSIRTSELR
GCcamGPEKDHVYLQLHHLPPEQLATR
GVIALCcamIEDGSIHR
ISQLYGDLKHLK
KPFGEHWR
LGANSLLDLVVFGR
NTVIATGGYGR
RLALTGAWPGTLQK
SMoxQNHAAVFR
SMoxTLEIREGR
SMQNHAAVFR
TEDGKIYQR
TLNEADCcamATVPPAIR
TYFSCcamTSAHTSTGDGTAMoxVTR
TYFSCcamTSAHTSTGDGTAMVTR
VDEYDYSKPIQGQQK
VGSVLQEGCcamEKISQLYGDLK
VPSIKANAGEESVMNLDK
VRVDEYDYSKPIQGQQK
VSDAISTQYPVVDHEFDAVVVGAGGAGLR
VTLEYRPVIDK
VTLEYRPVIDKTLNEADCcamATVPPAIR	
338	Sds protein [Mus musculus]	Q8VBT2
	33,1	6,4	138	58	14	ALGVNTVGAQTLK
ETLSAKPGAIVLSVGGGGLLCcamGVVQGLR
FVDDEKILVEPACcamGAALAAVYSR
HFVCcamSSAGNAGMATAYAAR
HFVCcamSSAGNAGMoxATAYAAR
HFVCcamSSAGNAGMoxATAYAARR
ILVEPACcamGAALAAVYSR
LAGTSVFLKMoxDSSQPSGSFK
LFYEHPIFSEVISDQEAVSALEK
LGIPATIVVPNTTPALTIER
LKNEGATVEVVGEMLDEAIQVAK
LKNEGATVEVVGEMoxLDEAIQVAK
RLGIPATIVVPNTTPALTIER
TPLRDSMoxALSK	
339	Selenium binding protein 1 [Mus musculus]	P17563
	52,5	5,9	336	75	32	CcamGPGYSTPLEAMKGPR
CcamNVSSLHTSHCcamLASGEVMoxVSTLGDLQGNGK
DGFNPAHVEAGLYGSR
EEIVYLPCcamIYR
EGSMoxMoxLQIDVDTVNGGLK
FLHDPSATQGFVGCcamALSSNIQR
FYKNAEGTWSVEK
GGSVQVLEDQELTCcamQPEPLVVK
GPREEIVYLPCcamIYR
GSFVLLDGETFEVK
HEIIQTLQMoxTDGLIPLEIR
HEIIQTLQMTDGLIPLEIR
HNVMoxVSTEWAAPNVFK
IFVWDWQR
IPGGPQMIQLSLDGK
IPGGPQMIQLSLDGKR
IPGGPQMoxIQLSLDGK
IPGGPQMoxIQLSLDGKR
IYVVDVGSEPR
IYVVDVGSEPRAPK
LAGQIFLGGSIVR
LHKVIEASEIQAK
LILPGLISSR
LNPNFLVDFGKEPLGPALAHELR
NAEGTWSVEK
NKLILPGLISSR
NTGTEAPDYLATVDVDPK
QFYPDLIR
QYDISNPQKPR
SPQYSQVIHR
VIEASEIQAK
YPGGDCcamSSDIWI	
340	Selenium binding protein 2; acetaminophen-binding protein [Mus musculus]	Q63836
	52,6	5,8	347	90	38	CcamGPGYPTPLEAMKGPR
CcamGPGYPTPLEAMoxKGPR
CcamNVSNTHTSHCcamLASGEVMoxVNTLGDLQGNGK
DGFNPAHVEAGLYGSR
EEIVYLPCcamIYR
EGSVMoxLQVDVDTVNGGLK
FLHDPSATQGFVGCcamALSSNIQR
FYKNEEGTWSVEK
GGSVQVLEDQELTCcamQPEPLVVK
GPREEIVYLPCcamIYR
GSFVLLDGETFEVK
GTWEKPGGASPMGYDFWYQPR
GTWEKPGGASPMoxGYDFWYQPR
HEIIQTLQMoxTDGLIPLEIR
HEIIQTLQMTDGLIPLEIR
HNVMoxVSTEWAAPNVFK
HNVMoxVSTEWAAPNVFKDGFNPAHVEAGLYGSR
HNVMVSTEWAAPNVFK
IFVWDWQR
IPGGPQMIQLSLDGK
IPGGPQMIQLSLDGKR
IPGGPQMoxIQLSLDGKR
IYVVDVGSEPR
LILPGLMoxSSR
LILPGLMSSR
LNPNFLVDFGK
LNPNFLVDFGKEPLGPALAHELR
LPMPYLKDELHHSGWNTCcamSSCcamFGDSTK
LTGQIFLGGSIVR
LYATTSLYSDWDKQFYPDLIR
NKLILPGLMoxSSR
NKLILPGLMSSR
NTGTEAPDYLATVDVDPK
oxVKGWMLPEMPGLITDILLSLDDR
QFYPDLIR
QYDISNPQKPR
SPQYSQVIHR
YPGGDCcamSSDIWI	
341	Selenophosphate synthetase 1 [Mus musculus]	Q8BH69
	42,9	5,5	189	50	15	ESFNPETYELDKSFR
HGGLSLVQTTDYIYPIVDDPYMoxMoxGR
LGIGMoxDTCcamVIPLR
LLESLQENHFQEDEQFLGAVMoxPR
NEVSFVIHNLPVLAK
TAAGLMoxHTFNAHAATDITGFGILGHAQNLAK
YGEGHQAWIIGIVEK	
342	SPS2_MOUSE Selenide,water dikinase 2 (Selenophosphate synthetase 2) (Selenium donor protein 2)	P97364
	47,8	5,7	105	36	12	ASGRFGLLQGTSAETSGGLLICcamLPR
EEVELAYQEAMoxFNMoxATLNR
FGLLQGTSAETSGGLLICcamLPR
YGEGHQAWIVGIVEK
HGGLSLVQTTDFFYPLVEDPYMoxMoxGR
MoxVVSREEVELAYQEAMoxFNMoxATLNR
NEVSFVIHNLPIIAK
QQKNEVSFVIHNLPIIAK
SFSNYRPFEPQTLGFSPSWR
SSKYGEGHQAWIVGIVEK
TAAGLMoxHTFNAHAATDITGFGILGHSQNLAK
YGEGHQAWIVGIVEKGNR	
343	Septin 2; neural precursor cell expressed, developmentally down-regulated gene 5 [Mus musculus]	P42208
	41,5	6	233	54	19	AIHNKVNIVPVIAK
ASIPFSVVGSNQLIEAK
DCcamFKTIISYIDEQFER
GRLYPWGVVEVENPEHNDFLK
IIPGAAEKIER
LLKASIPFSVVGSNQLIEAK
LTVVDTPGYGDAINCcamR
LTVVDTPGYGDAINCcamRDCcamFK
LYPWGVVEVENPEHNDFLK
LYPWGVVEVENPEHNDFLKLR
QQPTQFINPETPGYVGFANLPNQVHR
QQPTQFINPETPGYVGFANLPNQVHRK
STLINSLFLTDLYPER
STLINSLFLTDLYPERIIPGAAEK
TIISYIDEQFER
TMoxLITHMoxQDLQEVTQDLHYENFR
TVQIEASTVEIEER
TVQIEASTVEIEERGVK
YLHDESGLNRR	
344	Serbp1 protein  [Mus musculus]	Q9CY58
	42,2	8,4	114	34	16	EFDRHSGSDR
EMoxTLDEWKAIQNK
KPNEGADGQWK
KPNEGADGQWKK
PGHLQEGFGCcamVVTNR
RFEKPLEEK
RPDQQLQGDGK
RPDQQLQGDGKLIDR
SAAQAAAQTNSNAAGK
SEEAHAEDSVMDHHFR
SEEAHAEDSVMoxDHHFR
SKSEEAHAEDSVMDHHFR
SKSEEAHAEDSVMoxDHHFR
SSASAPDVDDPEAFPALA
TDKSSASAPDVDDPEAFPALA
VGRRPDQQLQGDGK	
345	Serpina1a protein [Mus musculus]	Q00896
	46	5,3	170	56	17	AVLTIDETGTEAAAVTVLLAVPYSMoxPPILR
AVLTIDETGTEAAAVTVLLAVPYSMPPILR
DQSPASHEIATNLGDFAISLYR
FDHPFLFIIFEEHTQSPLFVGK
IFNNGADLSGITEENAPLK
IFNNGADLSGITEENAPLKLSQAVHK
KLDQDTVFALANYILFK
KPFDPENTEEAEFHVDESTTVK
LDQDTVFALANYILFK
LSISGEYNLK
NHYQAEVFSVNFAESEEAKK
RLAQIHFPR
SFQHLLQTLNRPDSELQLSTGNGLFVNNDLK
TLMoxSPLGITR
TLMSPLGITR
TLMSPLGITRIFNNGADLSGITEENAPLK
VINDFVEKGTQGK	
346	Serine (or cysteine) proteinase inhibitor, clade A, member 1d; serine protease inhibitor 1-4 [Mus musculus]	Q00897
	46	5,2	187	47	13	AVLTIDETGTEAAAATVLQVATYSMoxPPIVR
DQSPASHEIATNLGDFALR
FDHPFLFIIFEEHTQSPIFVGK
IFNNGADLSGITEENAPLK
IFNNGADLSGITEENAPLKLSK
KLDQDTVFALANYILFK
LDQDTVFALANYILFK
LSISGNYNLK
NHYQAEVFSVNFAESEEAKK
QPFDPENTEEAEFHVDESTTVK
RSDAQIHIPR
TLMoxSPLGITR
VINDFVEKGTQGK	
347	Serpinb1a protein [Mus musculus]	Q9D154
	42,6	5,7	255	54	21	ADLSGMoxSGSRDLFISK
ENLEFIDVHVK
ENLEFIDVHVKLPR
FKIEESYTLNSNLGR
FMoxTEDTTDAPFR
FQSLNAEVSKR
GQTEGKIPELLSVGVVDSMoxTK
HNPTSNVLFLGR
IEESYTLNSNLGR
IPELLSVGVVDSMoxTK
KFPFGYISDLK
LANRLYGEK
LGVQDLFSSSK
LYGEKTYNFLPEYLASTQK
MoxYGADLAPVDFLHASEDAR
MoxYGADLAPVDFLHASEDARK
oxGMWEEKFMTEDTTDAPFR
RENLEFIDVHVK
TFHFDSVEDIHSR
TYNFLPEYLASTQK
VLEMoxPYQGGELSMoxVILLPK	
348	Splicing factor proline/glutamine rich (polypyrimidine tract binding protein associated) [Mus musculus]	Q8VIJ6
	75,4	9,9	199	39	22	ALAEIAKAELDDTPMoxR
AVVIVDDRGR
FAQHGTFEYEYSQR
FATHAAALSVR
FGQGGAGPVGGQGPR
GFGFIKLESR
GGKMoxPGGPKPGGGPGMoxGAPGGHPKPPHR
GGRQHHAPYHQQHHQGPPPGGPGPR
GIVEFASKPAAR
GMoxGPGTPAGYGR
ISDSEGFKANLSLLR
LFVGNLPADITEDEFKR
MoxEELHSQEMoxQKR
MoxGYMoxDPRER
MoxPGGPKPGGGPGMoxGAPGGHPKPPHR
NLSPYVSNELLEEAFSQFGPIER
QHHAPYHQQHHQGPPPGGPGPR
RPGEKTYTQR
SPPPGMoxGLNQNR
STGKGIVEFASKPAAR
VRFATHAAALSVR
YGEPGEVFINK
YGEPGEVFINKGK	
349	Serine hydroxymethyl transferase 1 (soluble) [Mus musculus]	P50431
	52,6	6,4	219	57	26	ALSDALTELGYK
ALSDALTELGYKIVTGGSDNHLILMDLR
ALSDALTELGYKIVTGGSDNHLILMoxDLR
AVLEALGSCcamLNNK
AVLEALGSCcamLNNKYSEGYPGQR
GIELTLQIQSHMATK
GIELTLQIQSHMoxATK
GLLEEDFQKVAHFIHR
IQSAVATLREEVENFASNFSLPGLPDF
ISATSIFFESMoxPYK
ISATSIFFESMPYK
IVTGGSDNHLILMDLR
IVTGGSDNHLILMoxDLR
IYQLQVLANCcamR
LIIAGTSCcamYSR
MoxLSQPLKDSDAEVYSIIK
NTCcamPGDKSALRPSGLR
oxIMGLDLPDGGHLTHGFMTDKK
QAMoxTTEFKIYQLQVLANCcamR
QAMTTEFKIYQLQVLANCcamR
QRVGLELIASENFASR
VGLELIASENFASR
VLEACcamSIACcamNK
VLEACcamSIACcamNKNTCcamPGDK
VYPETGYINYDQLEENASLFHPK
YSEGYPGQR
YYGGTEFIDELEMLCcamQKR
YYGGTEFIDELEMoxLCcamQK
YYGGTEFIDELEMoxLCcamQKR	
350	Serine hydroxymethyl transferase 2 (mitochondrial) [Mus musculus]	Q9CZN7
	55,8	8,7	179	55	24	AALEALGSCcamLNNKYSEGYPGK
AHLLADMAHISGLVAAK
AHLLADMoxAHISGLVAAK
GFPMoxPGFDER
GFPMPGFDER
GLELIASENFCcamSR
GYSLVSGGTDTHLVLVDLRPK
IMoxGLDLPDGGHLTHGYMoxSDVKR
ISATSIFFESMoxPYK
LANLRQQVEQFAR
LIIAGTSAYAR
LNPQTGLIDYDQLALTAR
NAQAMoxADALLKR
oxIMGLDLPDGGHLTHGYMSDVKR
QFREDDFR
RGYSLVSGGTDTHLVLVDLRPK
SFLLKDPETSQR
TGKEIPYTFEDR
VIPSPFKYADVVTTTTHK
VLELVSITANKNTCcamPGDR
VVDFIDEGVNIGLEVKR
YSEGYPGKR
YYGGAEVVDEIELLCcamQR
YYGGAEVVDEIELLCcamQRR	
351	Solute carrier family 9 (sodium/hydrogen exchanger), isoform 3 regulator 1; sodium-hydrogen exchanger regulatory factor [Mus musculus]	P70441
	38,6	5,5	281	73	23	AQDRIVEVNGVCcamMoxEGK
AVDPDSPAEASGLR
AVDPDSPAEASGLRAQDR
EALVEPASESPRPALAR
GPNGYGFHLHGEK
GPNGYGFHLHGEKGK
GPNGYGFNLHSDK
IVEVNGVCcamMEGK
KGPNGYGFNLHSDK
LCcamCcamLEKGPNGYGFHLHGEK
LGVSIREELLRPQEK
LLVVDKETDEFFK
LLVVDPETDERLK
LVEVNGENVEKETHQQVVSR
MSADAAAGEPLPR
QHGDVVSAIKGGGDEAK
QVSTEPSSTSSSSSDPILDLNISLAVAK
RAPQMDWSK
RAPQMoxDWSK
SEQAEPPAAADTHEAGDQNEAEK
SEQAEPPAAADTHEAGDQNEAEKSHLR
VIPSQEHLDGPLPEPFSNGEIQK	
352	Cu/Zn superoxide dismutase 1513495A	P08228
	15,8	5,9	186	63	12	AVCcamVLKGDGPVQGTIHFEQK
DGVANVSIEDR
DGVANVSIEDRVISLSGEHSIIGR
GDGPVQGTIHFEQK
HGGPADEER
HVGDLGNVTAGK
HVGDLGNVTAGKDGVANVSIEDR
KHGGPADEER
LACcamGVIGIAQ
QDDLGKGGNEESTK
TMoxVVHEK
VISLSGEHSIIGR	
353	Sod2 protein [Mus musculus]	P09671
	24,1	9,4	109	50	8	AIWNVINWENVTER
FNGGGHINHTIFWTNLSPK
GDVTTQVALQPALK
GELLEAIKR
HHAAYVNNLNATEEKYHEALAK
LGPVAGAAGSR
LTAVSVGVQGSGWGWLGFNKEQGR
NVRPDYLK	
354	Sorbitol dehydrogenase 1 [Mus musculus]	Q64442
	38,2	6,4	279	79	32	AKEVGADFTIQVGK
AMGAAQVVVTDLSASR
AMoxGAAQVVVTDLSASR
AVEAFETAKK
EVDIKGVFR
EVGADFTIQVGK
EVGADFTIQVGKETPQEIASK
FYKHNADFCcamYK
GENLSLVVHGPGDIR
GENLSLVVHGPGDIRLENYPIPELGPNDVLLK
GSVSLGNKVLVCcamGAGPVGMoxVTLLVAK
GSVSLGNKVLVCcamGAGPVGMVTLLVAK
HLKPGDRVAIEPGVPR
HNADFCcamYK
IGDFVVKKPMoxVLGHEAAGTVTK
IGDFVVKKPMVLGHEAAGTVTK
IGRYNLTPTIFFCcamATPPDDGNLCcamR
KPMoxVLGHEAAGTVTK
KPMVLGHEAAGTVTK
LENYPIPELGPNDVLLK
LPDSVTFEEGALIEPLSVGIYACcamR
MHSVGICcamGSDVHYWEHGR
MoxHSVGICcamGSDVHYWEHGR
oxYCcamNTWPMAISMLASK
TLNVKPLVTHRFPLEK
VAIEPGVPREVDEYCcamK
VLVCcamGAGPVGMoxVTLLVAK
VLVCcamGAGPVGMVTLLVAK
VMIKCcamDPNDQNP
YCcamNTWPMAISMLASK
YCcamNTWPMoxAISMoxLASK
YNLTPTIFFCcamATPPDDGNLCcamR	
355	Sepiapterin reductase [Mus musculus]	Q64105
	27,9	5,3	212	69	21	AARDMoxLYQVLAAEEPSVR
DMLYQVLAAEEPSVR
DTFQSGAHVDFYDCcam
ELPRPEGLQR
ETSKDPELR
LKSDGALVDCcamGTSAQK
LLGLLQKDTFQSGAHVDFYDCcam
LLLINNAATLGDVSK
LLSAVRELPRPEGLQR
LLSPGSVMLVSAR
LLSPGSVMoxLVSAR
QLKEELGAQQPDLK
SDGALVDCcamGTSAQKLLGLLQK
TVVNISSLCcamALQPYK
TVVNISSLCcamALQPYKGWGLYCcamAGK
VLSYAPGPLDNDMoxQQLAR
VLSYAPGPLDNDMQQLAR
VVLAAADLGTEAGVQR	
356	Spermidine synthase [Mus musculus]	Q64674
	34	5,2	118	38	16	AAFVLPEFTR
AAFVLPEFTRK
ETCcamSLWPGQALSLQVEQLLHHR
ETCcamSLWPGQALSLQVEQLLHHRR
FLPGMAVGFSSSK
FLPGMoxAVGFSSSK
KFLPGMAVGFSSSK
KVLIIGGGDGGVLR
LTLHVGDGFEFMK
LTLHVGDGFEFMoxK
NPSTNFREPVQQLTQAQVEQMoxQLK
NPSTNFREPVQQLTQAQVEQMQLK
VLIIGGGDGGVLR
YQDILVFR
YYNSDMHR
YYNSDMoxHR	
357	START domain containing 10; serologically defined colon cancer antigen 10; serologically defined colon cancer antigen 28; phosphatidylcholine transfer protein-like [Mus musculus]	Q9JMD3
	33	6,5	145	53	16	ACcamIKYPEWK
AGVSVWVQAVEMDR
AGVSVWVQAVEMoxDR
AVSIQTGYLIQSTGPK
AVSIQTGYLIQSTGPKSCcamVITYLAQVDPK
CcamRMoxECcamCcamDVPAETLYDVLHDIEYR
ESVQVPDDQDFR
ESVQVPDDQDFRSFR
KWDSNVIETFDIAR
LTVNADVGYYSWR
MECcamCcamDVPAETLYDVLHDIEYR
MoxECcamCcamDVPAETLYDVLHDIEYR
MoxECcamCcamDVPAETLYDVLHDIEYRK
oxSWLPMGADYIIMNYSVKHPK
SCcamVITYLAQVDPK
SECcamEAEVGWNLTYSK
SWLPMoxGADYIIMoxNYSVKHPK	
358	Stress-induced phosphoprotein 1; stress-inducible protein; IEF SSP 3521; Hsp70/Hsp90 organizing protein [Mus musculus]	Q60864
	62,6	6,3	232	59	40	AIEVGRENR
AKELDPTNMoxTYITNQAAVHFEK
AKELDPTNMTYITNQAAVHFEK
ALDLDSSCcamKEAADGYQR
ALSAGNIDDALQCcamYSEAIK
AMoxADPEVQQIMoxSDPAMoxR
AYARIGNSYFK
AYEDGCcamKTVDLKPDWGK
CcamMoxMoxAQYNR
EGLQNMEAR
EGLQNMEARLAER
EGLQNMoxEAR
ELDPTNMoxTYITNQAAVHFEKGDYNK
ELIEQLQNKPSDLGTK
EQERLAYINPDLALEEK
EQVNELKEK
FMNPFNLPNLYQK
FMoxNPFNLPNLYQK
IGNSYFKEEK
IQKLMoxDVGLIAIR
KAAALEFLNR
KFMoxNPFNLPNLYQK
LAYINPDLALEEK
LDPQNHVLYSNR
LDPQNHVLYSNRSAAYAK
LLEFQLALK
LLEFQLALKDCcamEECcamIQLEPTFIK
LMDVGLIAIR
NKGNECcamFQK
oxAMADPEVQQIMSDPAMR
oxAMADPEVQQIMSDPAMR
oxCcamMMAQYNR
RAMoxADPEVQQIMoxSDPAMoxR
SLAEHRTPDVLK
SLLSDPTYR
SLLSDPTYRELIEQLQNKPSDLGTK
TVDLKPDWGK
TVDLKPDWGKGYSR
TYEEGLKHEANNLQLK
YKDAIHFYNK	
359	Stomatin-like protein 2 [Mus musculus]	Q99JB2
	38,4	8,9	139	56	15	APVPGAQNSSQSRR
ASYGVEDPEYAVTQLAQTTMoxR
DVQATDTSIEELGR
ERESLNANIVDAINQAADCcamWGIR
ESLNANIVDAINQAADCcamWGIR
ESMoxQMoxQVEAERR
ILAGALTQHNGDAAASLTVAEQYVSAFSK
ILEPGLNVLIPVLDR
ILEPGLNVLIPVLDRIR
NTVILFVPQQEAWVVER
QAQILASEAEKAEQINQAAGEASAVLAK
RATVLESEGTR
RDVQATDTSIEELGR
VKESMoxQMoxQVEAER
YEIKDIHVPPR	
360	SCB1_MOUSE Succinyl-CoA ligase [ADP-forming] beta-chain, mitochondrial precursor (Succinyl-CoA synthetase, betaA chain) (SCS-betaA) (ATP-specific succinyl-CoA synthetase beta subunit)	Q9Z2I9
	46,2	5,7	140	45	24	CcamDVIAQGIVMAVK
CcamDVIAQGIVMoxAVKDLEIR
DLEIRIPVVVR
EAHVDVKFQLPI
EYYFAITMER
EYYFAITMoxER
GTFTSGLKGGVK
ICcamNQVLVCcamER
ICcamNQVLVCcamERK
ILACcamDDLDEAAKMoxVVK
ILACcamDDLDEAAKMVVK
INFDSNSAYR
INFDSNSAYRQK
IVFSPEEAK
IVFSPEEAKAVSSQMIGQK
IVFSPEEAKAVSSQMoxIGQK
LHGGTPANFLDVGGGATVQQVTEAFK
LQGTRVDDAK
MoxGFPSNIVDSAAENMoxIK
MoxGFPSNIVDSAAENMoxIKLYNLFLK
REYYFAITMER
REYYFAITMoxER
VQAILVNIFGGIMoxR
VQAILVNIFGGIMR	
361	Succinate-CoA ligase, GDP-forming, alpha subunit [Mus musculus]	Q9WUM5
	35	10,2	147	40	18	AKPVVSFIAGITAPPGR
AKPVVSFIAGITAPPGRR
GGQKHLGLPVFNTVK
HLGLPVFNTVK
HLGLPVFNTVKEAK
IICcamQGFTGKQGTFHSQQALEYGTK
ISALQSAGVVVSMoxSPAQLGTTIYKEFEK
LIGPNCcamPGVINPGECcamK
MoxGHAGAIIAGGK
MoxGHAGAIIAGGKGGAK
QGTFHSQQALEYGTK
QGTFHSQQALEYGTKLVGGTTPGK	
362	SCB2_MOUSE Succinyl-CoA ligase [GDP-forming] beta-chain, mitochondrial precursor (Succinyl-CoA synthetase, betaG chain) (SCS-betaG) (GTP-specific succinyl-CoA synthetase beta subunit)	Q9Z2I8
	43,9	5,9	230	69	27	DIFAMDDKSENEPIENEAAR
DIFAMoxDDKSENEPIENEAAR
EAQVYEAFK
EIVLKAQILAGGR
ELELKVPLVVR
EQIDIFEGIKDSQAQR
ETYLAILMDR
FFVANTAKEALEAAK
GVFNSGLKGGVHLTK
IDATQVEVNPFGETPEGQVVCcamFDAK
INFDDNAEFR
INFDDNAEFRQK
KLMoxSEHGVR
LEGTNVQEAQNILK
LLTSDPKVEAILVNIFGGIVNCcamAIIANGITK
MAENLGFLGSLK
MoxAENLGFLGSLK
MoxAENLGFLGSLKNQAADQITK
NQAADQITKLYHLFLK
SSGLPITSAVDLEDAAK
SSGLPITSAVDLEDAAKK
VMoxVAEALDISRETYLAILMoxDR
VNKVMoxVAEALDISR
VPLVVRLEGTNVQEAQNILK
VVGELAQQMIGYNLATK
VVGELAQQMoxIGYNLATK	
363	Sulfite oxidase [Mus musculus]	Q8R086
	54	5,6	247	61	19	AMoxDPEAEVLLAYEMoxNGQPLPR
AQVPAEQKELNIICcamK
DHGFPVRVVVPGVVGAR
FVDLHPGGPSK
HEVTVTLQCcamAGNR
HEVTVTLQCcamAGNRR
IGELNPEDSMoxSPSVEASDPYADDPIRHPALR
INSQRPFNAEPPPELLTEGYITPNPIFFTR
LCcamDSEAHVCcamFEGLDSDPTGTAYGASIPLAR
LCcamDVLAQAGHR
LHVVGAPGGQSLSLSLDDLHKFPK
LMoxLAAGGPLEPFWALYAVHNQPHVR
NHLPVPNLDPHTYR
oxAMDPEAEVLLAYEMNGQPLPR
TGVWVTLGSEVFDVTK
TGVWVTLGSEVFDVTKFVDLHPGGPSK
VDVSVDGGLTWQEAELEGEEQCcamPR
VSVESEESYSHWQR
VSVESEESYSHWQRR	
364	Leucine rich repeat containing 35 [Mus musculus]	Q8C5W3
	48	5,4	212	63	25	ALHSFGIRDGDK
EDSERFFIR
EIAAFCcamAHVSELDLSDNK
GPGVGVHVPATPQGSPMKDR
IVSNVPQLEFLNLSSNPLSLSVLER
LEPLAEVDLRPQSSAK
LLGIPLLQPYTTEER
LLGIPLLQPYTTEERR
LLHITDNNLQDWTEIR
LLHITDNNLQDWTEIRK
LNGSVVTDGER
LNGSVVTDGEREDSER
LNLPSVLVLNSCcamGITCcamAGDER
LQDWHEVSK
oxMDQPSGRSFMQVLCcamEK
RGPGVGVHVPATPQGSPMoxK
SFMoxQVLCcamEK
SGLQSWEDIDKLNSFPK
TCcamAGSFSGVRK
VEVHFNDQVEEMoxSIR
VEVHFNDQVEEMSIR
YGKLEPLAEVDLRPQSSAK
YSPENFPYR
YSPENFPYRR
YYVDVPQEEVPFR
YYVDVPQEEVPFRYHELITK	
365	T-complex protein 1 [Mus musculus]	P11984
	60,4	5,7	260	76	37	AFHNEAQVNPER
AFHNEAQVNPERK
DDKHGSYENAVHSGALDD
DNKQAGVFEPTIVK
EGPLSVFGDRSTGEAVR
EQLAIAEFAR
FATEAAITILR
FATEAAITILRIDDLIK
GANDFMoxCcamDEMoxER
IACcamLDFSLQK
ICcamDDELILIK
IIGINGDYFANMoxVVDAVLAVK
IIGINGDYFANMVVDAVLAVK
ILATGANVILTTGGIDDMoxYLK
LGVQVVITDPEKLDQIR
LLEVEHPAAK
MLVDDIGDVTITNDGATILK
MoxLVDDIGDVTITNDGATILK
NLKWIGLDLVHGKPR
oxGANDFMCcamDEMER
QAGVFEPTIVK
QKIHPTSVISGYR
SLHDALCcamVVKR
SLKFATEAAITILR
SLLVIPNTLAVNAAQDSTDLVAK
SQIESMoxLINGYALNCcamVVGSQGMoxPK
SQNVMAAASIANIVK
SQNVMoxAAASIANIVK
SVVPGGGAVEAALSIYLENYATSMGSR
SVVPGGGAVEAALSIYLENYATSMoxGSR
VLCcamELADLQDKEVGDGTTSVVIIAAELLK
WIGLDLVHGKPR
YFVEAGAMAVR
YFVEAGAMoxAVR
YINENLIINTDELGRDCcamLINAAK
YPVNSVNILK
YTDARGQPR	
366	Transketolase [Mus musculus]	P40142
	60,6	6,6	216	54	29	AVELAANTKGICcamFIR
CcamEAFGWHTIIVDGHSVEELCcamK
DRTVPFCcamSTFAAFFTR
FIECcamYIAEQNMoxVSIAVGCcamATR
GITGIEDKEAWHGKPLPK
IIALDGDTKNSTFSELFK
ILATPPQEDAPSVDIANIR
ILTVEDHYYEGGIGEAVSAAVVGEPGVTVTR
ISSDLDGHPVPK
KISSDLDGHPVPK
LDNLVAIFDINR
LGHASDRIIALDGDTK
LGQSDPAPLQHQVDIYQK
LGQSDPAPLQHQVDIYQKR
MFGIDKDAIVQAVK
MoxFGIDKDAIVQAVK
NMAEQIIQEIYSQVQSK
NMoxAEQIIQEIYSQVQSK
NPHNDRFVLSK
NSTFSELFKK
RCcamEAFGWHTIIVDGHSVEELCcamK
SKDDQVTVIGAGVTLHEALAAAESLK
SVPMoxSTVFYPSDGVATEK
SVPMoxSTVFYPSDGVATEKAVELAANTK
TSRPENAIIYSNNEDFQVGQAK
TVPFCcamSTFAAFFTR
VLDPFTIKPLDR	
367	Tropomodulin 3 [Mus musculus]	Q9JHJ0
	39,5	5	199	57	18	AANAITKNNDLVR
DLGDYKDLDEDELLGK
FGYQFTQQGPR
IDNQRQQLGTSVELEMoxAK
ILPVFDEPPNPTNVEESLK
ILPVFDEPPNPTNVEESLKR
LSESELKQLETVLDDLDPENALLPAGFR
LVEVNLNNIK
QLETVLDDLDPENALLPAGFR
QQLGTSVELEMoxAK
QQLGTSVELEMoxAKMoxLEENTNILK
SATGPFDRER
SLNMoxESNFITGAGVLALIDALR
SLNMoxESNFITGAGVLALIDALRDNETLMoxELK
SNDPVAVAFADMLK
SNDPVAVAFADMoxLK
SNDPVAVAFADMoxLKVNK
TLEANTHVKHFSLAATR	
368	Triosephosphate isomerase [Mus musculus]	Q64513
	22,5	5,6	300	87	21	DLGATWVVLGHSER
DLGATWVVLGHSERR
ELASQPDVDGFLVGGASLKPEFVDIINAK
ELASQPDVDGFLVGGASLKPEFVDIINAKQ
GWLKSNVNDGVAQSTR
HVFGESDELIGQK
HVFGESDELIGQKVSHALAEGLGVIACcamIGEK
IAVAAQNCcamYK
IIYGGSVTGATCcamK
LDEREAGITEK
RHVFGESDELIGQK
SNVNDGVAQSTR
TATPQQAQEVHEK
TATPQQAQEVHEKLR
VIADNVKDWSK
VSHALAEGLGVIACcamIGEK
VSHALAEGLGVIACcamIGEKLDER
VTNGAFTGEISPGMIK
VTNGAFTGEISPGMIKDLGATWVVLGHSER
VTNGAFTGEISPGMoxIK
VVLAYEPVWAIGTGK	
369	Tropomyosin	Q63610
	29	4,7	105	50	14	AREQAEAEVASLNR
CcamREMDEQIR
CcamREMoxDEQIR
CcamTKEEHLCcamTQR
EAETRAEFAER
EQAEAEVASLNR
EQAEAEVASLNRR
IQLVEEELDRAQER
IQVLQQQADDAEERAER
KLVIIEGDLER
LATALQKLEEAEK
LQREVEGER
MoxLDQTLLDLNEMox
TEERAELAESR	
370	Similar to Endoplasmin precursor (Endoplasmic reticulum protein 99) (94 kDa glucose-regulated protein) (GRP94) (ERP99) (Polymorphic tumor rejection antigen 1) (Tumor rejection antigen gp96) [Rattus norvegicus]	Q66HD0
	92,8	4,7	206	41	35	AQAYQTGKDISTNYYASQK
EAESSPFVER
EFEPLLNWMoxK
EKNLLHVTDTGVGMoxTR
ELISNASDALDKIR
EVEEDEYKAFYK
FAFQAEVNR
FAFQAEVNRMoxMoxK
FQSSHHSTDITSLDQYVER
FQSSHHSTDITSLDQYVERMoxK
GLFDEYGSK
GTTITLVLKEEASDYLELDTIK
GVVDSDDLPLNVSR
GVVDSDDLPLNVSRETLQQHK
GYEVIYLTEPVDEYCcamIQALPEFDGKR
HPLIRDMoxLR
IADEKYNDTFWK
IYFMoxAGSSR
IYFMoxAGSSRK
KEAESSPFVER
LGVIEDHSNR
LGVIEDHSNRTR
LISLTDENALAGNEELTVK
LLRFQSSHHSTDITSLDQYVER
LTESPCcamALVASQYGWSGNMER
NLLHVTDTGVGMoxTREELVK
NLLHVTDTGVGMTR
oxRVFITDDFHDMMPK
oxVFITDDFHDMMPK
RVFITDDFHDMoxMoxPK
SEKFAFQAEVNR
SILFVPTSAPR
TFEINPRHPLIR
VFITDDFHDMoxMoxPK
YSQFINFPIYVWSSK	
371	Trap1 protein [Mus musculus]	Q922Z3
	80	6,2	302	52	34	AFLEALQNQAETSSK
AQLLQPTLEINPR
DISEFQHEEFYR
EGIVTTAEQDIKEDIAK
ELGSSVALYSR
ELISNASDALEKLR
ELLQESALIR
ELLQESALIRK
FFEDYGLFMoxR
FFEDYGLFMR
FIRGVVDSEDIPLNLSR
FTLHYKTDAPLNIR
GSVSKHEFQAETK
GVVDSEDIPLNLSR
HKLVCcamEGQVLPEMEIHLQTDAK
HKLVCcamEGQVLPEMoxEIHLQTDAK
HLAEHSPYYEAMK
HLAEHSPYYEAMoxK
HLAEHSPYYEAMoxKQK
KGTITIQDTGIGMoxTQEELVSNLGTIAR
LLRYESSALPAGQLTSLPDYASR
LSEKETEDLMoxAWMoxR
NIYYLCcamAPNR
oxSIFYVPEMKPSMFDVSR
SAAPESPGYQWLSDGSGVFEIAEASGVRPGTK
SDCcamKDFASESR
SIFYVPEMoxKPSMoxFDVSR
SLYSEKEVFIR
TQEERAQLLQPTLEINPR
VQDVVTKYSNFVSFPLYLNGK
YESSALPAGQLTSLPDYASR
YIAQAYDKPR
YSNFVSFPLYLNGK
YSNFVSFPLYLNGKR	
372	Transferrin; hypotransferrinemia with hemochromatosis [Mus musculus]	Q921I1
	76,7	6,8	485	60	41	ASDTSITWNNLKGK
AVSSFFSGSCcamVPCcamADPVAFPK
CcamAPNNKEEYNGYTGAFR
CcamFVKLPEGTTPEK
CcamISFRDHMoxK
CcamLKDGGGDVAFVK
CcamLVEKGDVAFVK
DFASCcamHLAQAPNHVVVSR
DFQLFSSPLGK
DLLFKDSAFGLLR
DQYELLCcamLDNTR
DSAFGLLR
DSAFGLLRVPPR
GTDFQLNQLEGKK
GYYAVAVVK
HQTVLDNTEGKNPAEWAK
IPSHAVVAR
KPVDQYEDCcamYLAR
KPVKDFASCcamHLAQAPNHVVVSR
KSCcamHTGLGR
LCcamQLCcamPGCcamGCcamSSTQPFFGYVGAFK
LLEACcamTFHKH
LYLGHNYVTAIR
NLKQEDFELLCcamPDGTR
NQQEGVCcamPEGSIDNSPVK
oxSCcamHTGVDRTAGWNIPMGMLYNR
oxTAGWNIPMGMLYNR
oxYLGAEYMQSVGNMRK
SAGWVIPIGLLFCcamK
SCcamHTGLGR
SCcamHTGVDR
SKDFQLFSSPLGK
TAGWNIPMGMLYNR
TAGWNIPMoxGMoxLYNR
TVLPPDGPR
VAQEHFGK
VAQEHFGKGK
VKAVLTSQETLFGGSDCcamTGNFCcamLFK
VPPRMDYR
VPPRMoxDYR
WCcamALSHLER
WCcamAVSEHENTK
YLGAEYMoxQSVGNMoxR
YLGAEYMoxQSVGNMoxRK	
373	Thiosulfate sulfurtransferase, mitochondrial [Mus musculus]	P52196
	33,5	7,6	174	56	16	ALVSTKWLAESIR
DGFEKSPEELR
EGHPVTSEPSRPEPAVFK
EYQERHVPGASFFDIEECcamR
GSVNMoxPFMoxDFLTKDGFEK
RFQLVDSR
TVSVLNGGFR
TVSVLNGGFRNWLK
TYEQVLENLQSKR
VDLSQPLIATCcamR
VDLSQPLIATCcamRK
VHQVLYR
VLDASWYSPGTR
VWWMFR
VWWMoxFR
YLGTQPEPDIVGLDSGHIR	
374	Transthyretin Mus musculus	P07309
	15,8	5,5	127	94	10	FVEGVYRVELDTK
GSPAVDVAVKVFK
HYTIAALLSPYSYSTTAVVSNPQN
KTSEGSWEPFASGK
LFLLCcamLAGLVFVSEAGPAGAGESKCcamPLMoxVK
TAESGELHGLTTDEK
TAESGELHGLTTDEKFVEGVYR
TLGISPFHEFADVVFTANDSGHR
TSEGSWEPFASGK
VLDAVRGSPAVDVAVK	
375	class II beta tubulin isotype [Homo sapiens]	Q13885
	49,9	4,8	217	70	34	AILVDLEPGTMDSVR
AILVDLEPGTMoxDSVR
ALTVPELTQQMFDSK
ALTVPELTQQMoxFDSKNMoxMoxAACcamDPR
EIVHIQAGQCcamGNQIGAK
ESESCcamDCcamLQGFQLTHSLGGGTGSGMGTLLISK
FPGQLNADLR
FPGQLNADLRK
FWEVISDEHGIDPTGSYHGDSDLQLER
GHYTEGAELVDSVLDVVR
GHYTEGAELVDSVLDVVRK
HGRYLTVAAIFR
INVYYNEAAGNK
INVYYNEAAGNKYVPR
IREEYPDR
ISEQFTAMFR
ISEQFTAMFRR
ISEQFTAMoxFR
ISEQFTAMoxFRR
KLAVNMoxVPFPR
LAVNMoxVPFPR
LAVNMVPFPR
LHFFMoxPGFAPLTSR
LHFFMPGFAPLTSR
LTTPTYGDLNHLVSATMoxSGVTTCcamLR
LTTPTYGDLNHLVSATMSGVTTCcamLR
MoxREIVHIQAGQCcamGNQIGAK
MoxSATFIGNSTAIQELFKR
MREIVHIQAGQCcamGNQIGAK
MSATFIGNSTAIQELFKR
NSSYFVEWIPNNVK
SGPFGQIFRPDNFVFGQSGAGNNWAK
TAVCcamDIPPRGLK
YLTVAAIFR	
376	Tubulin, beta, 2 [Mus musculus]	P68372
	49,8	4,6	192	55	27	ALTVPELTQQMoxFDAK
AVLVDLEPGTMoxDSVR
FPGQLNADLR
FPGQLNADLRK
FWEVISDEHGIDPTGTYHGDSDLQLER
GHYTEGAELVDSVLDVVR
GHYTEGAELVDSVLDVVRK
INVYYNEATGGK
ISEQFTAMFR
ISEQFTAMoxFR
ISEQFTAMoxFRR
KLAVNMoxVPFPR
LAVNMoxVPFPR
LAVNMVPFPR
LHFFMoxPGFAPLTSR
LHFFMPGFAPLTSR
LTTPTYGDLNHLVSATMoxSGVTTCcamLR
LTTPTYGDLNHLVSATMSGVTTCcamLR
MoxREIVHLQAGQCcamGNQIGAK
MoxSATFIGNSTAIQELFK
MoxSATFIGNSTAIQELFKR
MREIVHLQAGQCcamGNQIGAK
MSATFIGNSTAIQELFK
MSATFIGNSTAIQELFKR
NSSYFVEWIPNNVK
SGPFGQIFRPDNFVFGQSGAGNNWAK
YLTVAAVFR	
377	Tu translation elongation factor, mitochondrial [Mus musculus]	Q8BFR5
	49,5	7,1	269	67	22	ADAVQDSEMoxVELVELEIR
ADAVQDSEMVELVELEIR
AEAGDNLGALVR
ARGITINAAHVEYSTAAR
DKPHVNVGTIGHVDHGK
DKPHVNVGTIGHVDHGKTTLTAAITK
DLDKPFLLPVESVYSIPGR
ELAMoxPGEDLK
ELAMoxPGEDLKLSLILR
ELLTEFGYKGEETPVIVGSALCcamALEQR
GITINAAHVEYSTAAR
GLVMoxVKPGSIQPHQK
GTVVTGTLERGILK
HYAHTDCcamPGHADYVK
KGDECcamELLGHNK
KYEEIDNAPEER
LLDAVDTYIPVPTR
NMoxITGTAPLDGCcamILVVAANDGPMoxPQTR
QIGVEHVVVYVNK
SLERAEAGDNLGALVR
TIGTGLVTDVPAMoxTEEDKNIK
TIGTGLVTDVPAMTEEDKNIK
TVVTGIEMFHK
VEAQVYILSK
VEAQVYILSKEEGGR	
378	Thioredoxin domain containing 4; endoplasmic reticulum resident protein 44kDa [Mus musculus]	Q9D1Q6
	46,9	5	114	44	15	DDTESLEIFQNEVAR
DSENYRVFER
FRHPLLHIQK
GTINFLHADCcamDKFR
HMoxYVFGDFKDVLIPGK
LKQFVFDLHSGK
MNPAVFLSLADLR
NIIGYFEQK
NIIGYFEQKDSENYR
QFVFDLHSGK
SNPVHEIQSLDEVTNLDR
TPADCcamPVIAIDSFR
VASILHDDCcamAFLSAFGDLSKPER
VDCcamDQHSDIAQR
YNGDNVIYKPPGR	
379	Txndc5 protein [Mus musculus]	Q91W90
	39,2	5,2	212	66	24	ALAPTWEQLALGLEHSETVK
DLDSLHSFVLR
EFPGLSDVTIAEVDCcamTAERNVCcamSK
FFKPGQEAVK
FFKPGQEAVKYQGPR
FYAPWCcamGHCcamK
GYPTLKFFKPGQEAVK
GYPTLLLFR
GYPTLLLFRGGEK
GYPTLLWFR
GYPTLLWFRDGK
IGKVDCcamTQHYAVCcamSEHQVR
LQPTWNDLGDKYNSMEDAK
LQPTWNDLGDKYNSMoxEDAK
NLAPTWEELSK
NLAPTWEELSKK
QGLYELSANNFELHVSQGNHFIK
SFEDTIAQGITFVK
VDCcamTADSDVCcamSAQGVR
VDCcamTADSDVCcamSAQGVRGYPTLK
VDCcamTQHYAVCcamSEHQVR
VGEHNGGRDLDSLHSFVLR
VYVAKVDCcamTADSDVCcamSAQGVR
YSVRGYPTLLLFR	
380	Thioredoxin-like 1; thioredoxin-related protein; thioredoxin-like (32kD); thioredoxin-like [Mus musculus]	Q8CDN6
	32,2	4,8	195	70	19	AGCcamECcamLNESDEHGFDNCcamLR
AGCcamECcamLNESDEHGFDNCcamLRK
FQGPDNGQGPK
FQGPDNGQGPKYVK
FQNVNSVTLFVQSNQGEEETTR
FTMoxRGCcamGPCcamLR
GYMoxDLMoxPFINK
IDQYQGADAVGLEEK
IKQHLENDPGSNEDADIPK
ISYFTFIGTPVQATNMNDFKR
ISYFTFIGTPVQATNMoxNDFKR
LYSMoxKFQGPDNGQGPK
oxGYMDLMPFINK
SEPTQALELTEDDIKEDGIVPLR
SMDFEEAER
SMoxDFEEAER
VGVKPVGSDPDFQPELSGAGSR
VRIDQYQGADAVGLEEK
YVKIFINLPR	
381	Thioredoxin-like 2; PKC interacting cousin of thioredoxin [Mus musculus]	Q9CQM9
	37,8	5,3	114	45	11	GDLVGGLDIVKELK
GTPQEPRCcamGFSK
HNIQFSSFDIFSDEEVR
HNIQFSSFDIFSDEEVRQGLK
HVSSGAFPPSTNEHLKEDLSLR
KLTHAAPCcamMoxLFMoxK
LTHAAPCcamMoxLFMoxK
QILEILNSTGVEYETFDILEDEEVRQGLK
TFSNWPTYPQLYVR
VDRLDGAHAPELTK
YEISSVPTFLFFK	
382	Ubiquitin carboxyl-terminal esterase L3 (ubiquitin thiolesterase) [Mus musculus]	Q9JKB1
	26,2	4,9	191	62	16	AKFLENYDAIR
FLEESVSMoxSPEER
FLEESVSMSPEER
FLENYDAIR
FMERDPDELR
FMoxERDPDELR
KFLEESVSMoxSPEER
KPFPINHGK
QTISNACcamGTIGLIHAIANNKDK
SQGQDVTSSVYFMK
SQGQDVTSSVYFMoxK
TSDETLLEDAIEVCcamK
TSDETLLEDAIEVCcamKK
VTHETSAHEGQTEAPSIDEK
WLPLEANPEVTNQFLK
YEVFRTEEEEK	
383	Ugp2 protein [Mus musculus]	Q8R0M2
	55,5	6,7	259	66	34	AHVDEFKSVSK
ARGLPDNISSVLNK
CcamEFVMEVTNKTR
CcamEFVMoxEVTNK
CcamEFVMoxEVTNKTR
FLQEKGPSVDWGK
GGTLTQYEGK
GGTLTQYEGKLR
GLPDNISSVLNKLVVVK
GTVIIIANHGDR
GTVIIIANHGDRIDIPPGAVLENK
IFNTNNLWISLGAVKR
ILQKYNHCcamR
ILTTAASHEFEHTKK
INKESLLPIAK
IQRPPEDSIQPYEK
IQRPPEDSIQPYEKIK
IYTFNQSRYPR
LQEQNAIDMEIIVNPK
LQEQNAIDMoxEIIVNPK
NENTFLDLTVQQIEHLNK
oxTTSDLLLVMSNLYSLNAGSLTMSEKR
RCcamEFVMoxEVTNK
REFPTVPLVK
RFESIPDMLELDHLTVSGDVTFGK
RFESIPDMoxLELDHLTVSGDVTFGK
RLQEQNAIDMEIIVNPK
SFENSLGINVPR
SLIGVRNENTFLDLTVQQIEHLNK
TLDGGLNVIQLETAVGAAIK
TLDGGLNVIQLETAVGAAIKSFENSLGINVPR
TTSDLLLVMoxSNLYSLNAGSLTMoxSEKR
TYNTDVPLVLMoxNSFNTDEDTKK
VKIYTFNQSR	
384	Urate oxidase; uricase [Mus musculus]	P25688
	29	8,5	194	74	23	AHVYVEEVPWK
AHVYVEEVPWKR
CcamFATQVYCcamK
DRCcamFATQVYCcamK
DVDFEAIWGAVR
DVDFEAIWGAVRDIVLQK
DYLHGDNSDIIPTDTIK
DYLHGDNSDIIPTDTIKNTVHVLAK
FAGPYDKGEYSPSVQK
GIRNIETFAMoxNICcamEHFLSSFNHVTR
HVHAFIHTPTGTHFCcamEVEQMoxR
HVHAFIHTPTGTHFCcamEVEQMR
MGLINKEEVLLPLDNPYGK
MoxGLINKEEVLLPLDNPYGK
NGIKHVHAFIHTPTGTHFCcamEVEQMoxR
NGIKHVHAFIHTPTGTHFCcamEVEQMR
NGPPVIHSGIKDLK
NIETFAMNICcamEHFLSSFNHVTR
NIETFAMoxNICcamEHFLSSFNHVTR
RDVDFEAIWGAVR
TTQSGFEGFLK
TTQSGFEGFLKDQFTTLPEVK
YHSIKEVATSVQLTLR	
385	Ureidopropionase, beta [Mus musculus]	Q8VC97
	43,9	6,2	241	63	25	AASERNFELK
AHHDLGYFYGSSYVAAPDGSR
DREHGGVLWNTAVVISNSGLVMoxGK
EKLPWTEFAESAEDGLTTR
ELAEAVKPNYSPNIVK
ELAEAVKPNYSPNIVKEDLVLAPSSG
EQQRCcamPQIVR
GYAFGAAKEQQR
HLPPDDLAQVKR
HNMoxVVVSPILER
HNMVVVSPILER
IPLPTSAPVAEQVSALHK
KAHHDLGYFYGSSYVAAPDGSR
KHNMoxVVVSPILER
KHNMVVVSPILER
LPWTEFAESAEDGLTTR
MoxTGRLEMoxYAR
NAAIANHCcamFTCcamALNR
NLDLPREALK
oxMTGRLEMYAR
VGDFNESTYYMEGNLGHPVFQTQFGR
VGDFNESTYYMoxEGNLGHPVFQTQFGR
VGLVQNRIPLPTSAPVAEQVSALHK
VGQEHFPNEFTSGDGK
VGQEHFPNEFTSGDGKK	
386	Ubiquinol-cytochrome c reductase core protein 1 [Mus musculus]	Q9CZ13
	52,9	5,7	236	52	28	AACcamSGTQVLLRTR
EVESIGAHLNAYSTR
EVESIGAHLNAYSTREHTAYLIK
GTKNRPGNALEK
IPLAEWESR
IQEVDAQMLR
IQEVDAQMoxLR
IQEVDAQMoxLRDICcamSK
LCcamTSATESEVTR
LCcamTSATESEVTRGK
LSRTDLTDYLNR
MoxVLAAAGGVEHQQLLDLAQK
MoxVLAAAGGVEHQQLLDLAQKHLSSVSR
MVLAAAGGVEHQQLLDLAQKHLSSVSR
NALVSHLDGTTPVCcamEDIGR
NNGAGYFLEHLAFKGTK
NRPGNALEKEVESIGAHLNAYSTR
RIPLAEWESR
SGMFWLRF
SGMoxFWLRF
TDLTDYLNR
VASEQSSHATCcamTVGVWIDAGSR
VVELLADIVQNSSLEDSQIEKER
VYEEDAVPGLTPCcamR
VYEEDAVPGLTPCcamRFTGSEIR
YFYDQCcamPAVAGYGPIEQLPDYNR
YFYDQCcamPAVAGYGPIEQLPDYNRIR	
387	Probable urocanate hydratase;cDNA sequence BC022133 [Mus musculus]	Q8VC12
	74,6	7,1	129	43	25	AAAIVGCcamIGVIAEVDKAALVK
ARMMLSWDVSNGVAR
AYPIDQYPCcamR
AYPIDQYPCcamRTR
EAAKHQLVVGSQAR
EVLSLGYHGNVVDLWER
HLVQESLRR
HQLVVGSQAR
HRQGWLMoxEVTDSLDHCcamIAR
ILYSDQKGR
LAQEKFFFWDYGNAFLLEAQR
LLALEFAQELR
LQYMoxDNIR
LVITNGMoxVIPNYSSR
MoxMoxLSWDVSNGVAR
MoxTEEQTLVMoxYSGHPLGLFPSSPR
oxMMLSWDVSNGVAR
QFGHIYMoxYR
QFGHIYMYR
TPNLSPEEEQLALR
VAIAVAINQAIASGK
VFVTSGLGGMoxSGAQAK
WAGVPHAPVR
WVCcamTSGDPQDLAVTDHLATSVLEK
YLGIENLAGKVFVTSGLGGMoxSGAQAK	
388	Uroporphyrinogen decarboxylase [Mus musculus]	P70697
	40,7	6	165	54	16	AAQDFFSTCcamR
AAWGEETDYTPVWCcamMoxR
AAWGEETDYTPVWCcamMR
AGLAPVPMoxIIFAK
AVTLQGNLDPCcamALYASEEEIGR
DGHFALEELAQAGYEVVGLDWTVAPK
FALPYIRDVAK
GPSFPEPLREER
LRDPAAAASELGYVFQAITLTR
LVQQMLDDFGPQR
LVQQMoxLDDFGPQR
SPEACcamCcamELTLQPLR
SPEACcamCcamELTLQPLRR
VGAFVDAVHKHSR
YIANLGHGLYPDMoxDPER
YLPEFRETR	
389	Vinculin; metavinculin [Mus musculus]	Q64727
	116,7	5,7	151	25	17	AAVHLEGKIEQAQR
AIPDLTAPVAAVQAAVSNLVR
CcamDRVDQLTAQLADLAAR
EAFQPQEPDFPPPPPDLEQLR
GILEYLTVAEVVETMoxEDLVTYTK
GILSGTSDLLLTFDEAEVR
GILSGTSDLLLTFDEAEVRK
GWLRDPNASPGDAGEQAIR
IRTDAGFTLR
MoxSAEINEIIR
NQGIEEALKNR
oxMALLMAEMSR
QQELTHQEHR
SFLDSGYRILGAVAK
TIESILEPVAQQISHLVIMoxHEEGEVDGK
TNLLQVCcamERIPTISTQLK
WIDNPTVDDRGVGQAAIR	
390	Murine valosin-containing protein [Mus musculus]	Q01853
	89,3	5,1	360	64	44	AFEEAEKNAPAIIFIDELDAIAPK
AHVIVMAATNRPNSIDPALR
AHVIVMoxAATNRPNSIDPALR
AVANETGAFFFLINGPEIMoxSK
AVANETGAFFFLINGPEIMSK
DVDLEFLAK
EAVCcamIVLSDDTCcamSDEKIR
EDEEESLNEVGYDDVGGCcamRK
ELQELVQYPVEHPDKFLK
EMoxVELPLRHPALFK
EMVELPLRHPALFK
ESIESEIRR
ETVVEVPQVTWEDIGGLEDVKR
EVDIGIPDATGRLEILQIHTK
FDREVDIGIPDATGR
FPSGNQGGAGPSQGSGGGTGGSVYTEDNDDDLYG
GILLYGPPGTGK
GPELLTMoxWFGESEANVR
GVLFYGPPGCcamGK
GVLFYGPPGCcamGKTLLAK
IVSQLLTLMDGLK
IVSQLLTLMDGLKQR
IVSQLLTLMoxDGLK
IVSQLLTLMoxDGLKQR
KYEMFAQTLQQSR
KYEMoxFAQTLQQSR
LADDVDLEQVANETHGHVGADLAALCcamSEAALQAIR
LAGESESNLR
LAGESESNLRK
LDQLIYIPLPDEK
LGDVISIQPCcamPDVK
LIVDEAINEDNSVVSLSQPK
MoxDELQLFRGDTVLLK
NAPAIIFIDELDAIAPK
NAPAIIFIDELDAIAPKR
NVFIIGATNRPDIIDPAILRPGR
oxVINQILTEMDGMSTKK
QAAPCcamVLFFDELDSIAK
QTNPSAMoxEVEEDDPVPEIRR
RDHFEEAMoxR
RIVSQLLTLMDGLK
RIVSQLLTLMoxDGLK
VRLGDVISIQPCcamPDVK
WALSQSNPSALR	
391	Porin isoform 1 [Homo sapiens]	P21796
	30,7	8,6	140	72	17	DVFTKGYGFGLIK
FGIAAKLQIDPDACcamFSAK
GLKLTFDSSFSPNTGK
GYGFGLIKLDLK
KLETAVNLAWTAGNSNTR
LETAVNLAWTAGNSNTR
LTFDSSFSPNTGK
LTFDSSFSPNTGKK
LTLSALLDGKNVNAGGHK
NVNAGGHKLGLGLEFQA
REHINLGCcamDMoxDFDIAGPSIR
TDEFQLHTNVNDGTEFGGSIYQK
VNNSSLIGLGYTQTLKPGIK
VTQSNFAVGYK
WNTDNTLGTEITVEDQLAR
WTEYGLTFTEK
YRWTEYGLTFTEK	
392	Voltage-dependent anion channel 1 [Mus musculus]	Q60932
	30,8	8,6	198	88	20	DVFTKGYGFGLIK
EHINLGCcamDVDFDIAGPSIR
GALVLGYEGWLAGYQMoxNFETSK
GLKLTFDSSFSPNTGK
GYGFGLIKLDLK
KLETAVNLAWTAGNSNTR
LETAVNLAWTAGNSNTR
LTFDSSFSPNTGKK
LTLSALLDGKNVNAGGHK
NVNAGGHKLGLGLEFQA
REHINLGCcamDVDFDIAGPSIR
SENGLEFTSSGSANTETTKVNGSLETK
SRVTQSNFAVGYK
TDEFQLHTNVNDGTEFGGSIYQK
VNNSSLIGLGYTQTLKPGIK
VTQSNFAVGYK
WNTDNTLGTEITVEDQLAR
WTEYGLTFTEK
YQVDPDACcamFSAK
YRWTEYGLTFTEK	
393	Vimentin [Mus musculus]	P20152
	53,7	5	260	81	33	DGQVINETSQHHDDLE
EKLQEEMLQR
ETNLESLPLVDTHSKR
FADLSEAANR
FADLSEAANRNNDALR
FANYIDKVR
HLREYQDLLNVK
ILLAELEQLK
ILLAELEQLKGQGK
ISLPLPTFSSLNLR
ISLPLPTFSSLNLRETNLESLPLVDTHSK
LGDLYEEEMoxRELR
LGDLYEEEMRELR
LHDEEIQELQAQIQEQHVQIDVDVSKPDLTAALR
LLEGEESRISLPLPTFSSLNLR
LLQDSVDFSLADAINTEFK
LLQDSVDFSLADAINTEFKNTR
LQDEIQNMoxKEEMoxAR
MALDIEIATYR
MALDIEIATYRK
MoxALDIEIATYR
MoxALDIEIATYRK
MoxFGGSGTSSRPSSNR
MoxSTRSVSSSSYR
NLQEAEEWYKSK
QMoxREMoxEENFALEAANYQDTIGR
QVDQLTNDKAR
QVQSLTCcamEVDALKGTNESLER
RMoxFGGSGTSSRPSSNR
SLYSSSPGGAYVTR
TNEKVELQELNDR
TVETRDGQVINETSQHHDDLE
TYSLGSALRPSTSR
VESLQEEIAFLKK
VEVERDNLAEDIMoxR
VEVERDNLAEDIMR	
394	WD repeat domain 1; WD40 repeat protein 1 [Mus musculus]	O88342
	66,4	6,1	236	66	26	AHDGGIYAISWSPDSTHLLSASGDKTSK
CcamVAVGPGGYTVVVCcamIGQIVLLKDQK
DHLLSISLSGYINYLDKNNPSKPLR
DYSGQGVVKLDVQPK
FATASADGQIFIYDGK
FGAVFLWDTGSSVGEITGHNK
FKFTIGDHSR
FSPDGNRFATASADGQIFIYDGK
GPVTDVAYSHDGAFLAVCcamDASK
IAVVGEGREK
ILGGDPKGDHFLYTNGK
IWDTTQKEHLLK
KVFASLPQVER
LATGSDDNCcamAAFFEGPPFK
LATGSDDNCcamAAFFEGPPFKFK
LHHVSSLAWLDEHTLVTTSHDASVK
NIDNPAIADIYTEHAHQVVVAK
oxMTVNESEQLVSCcamSMDDTVR
SIQCcamLTVHR
VFASLPQVER
VFASLPQVERGVSK
VINSVDIKQTRPYR
VVTVFSVADGYSENNVFYGHHAK
VYSILASTLKDEGK
YAPSGFYIASGDISGKLR
YEYQPFAGK
YTNLTLRDYSGQGVVK	
395	Zinc binding alcohol dehydrogenase, domain containing (unnamed protein product) [Mus musculus]	Q8VDQ1
	34,4	5,2	132	49	13	CcamLFLTSELGFDAAVNYK
ERFTVLNYK
GDFVTSFYWPWQTK
GHISAGSNQTMoxVVSGAAGACcamGSLAGQIGHLLGCcamSR
GHISAGSNQTMVVSGAAGACcamGSLAGQIGHLLGCcamSR
NGNPVAENFR
NGNPVAENFRVEEFSLPDALNEGQVQVR
TLYLSVDPYMoxR
TLYLSVDPYMR
VDPQLVDGHLSYFLGAIGMoxPGLTSLIGVQEK
VEEFSLPDALNEGQVQVR
VVGICcamGTQEK
VVGICcamGTQEKCcamLFLTSELGFDAAVNYK	
