# Supplementary material for: An update on the mouse liver proteome
Source: Proteome Sci. 2009 Sep 8;7:35. doi: 10.1186/1477-5956-7-35 (PMC2752743; doi:10.1186/1477-5956-7-35)
Supplement: Additional file 6 — Table 3. List of the 55 proteins not found in the work of Lai et al. [file 1477-5956-7-35-S6.doc]

### Table 3

| **SWISSPROT ID**  **(GAZZANA, BORLAK)** | SOURCE | **IPI ID** | PROTEIN NAME |
| --- | --- | --- | --- |
| Q9CR00 | MOUSE | IPI00761650 | 26s proteasome non-atpase regulatory subunit 9 |
| Q99J99 | MOUSE | IPI00762185 | 3-mercaptopyruvate sulfurtransferase |
| Q9D404 | MOUSE | IPI00136333 | 3-oxoacyl-[acyl-carrier-protein] synthase, mitochondrial precursor |
| P63260 | MOUSE | IPI00874482 | actin, cytoplasmic 2 |
| Q9CPW4 | MOUSE | IPI00399943 | actin-related protein 2/3 complex subunit 5 |
| Q9QYR9 | MOUSE | IPI00136683 | acyl-coenzyme a thioesterase 2, mitochondrial precursor |
| Q07076 | MOUSE | IPI00653636 | adult male medulla oblongata cdna, riken full-length enriched library, clone:6330438j06 product:annexin a7, full insert sequence |
| Q91X42 | MOUSE | IPI00471137 | aldo-keto reductase family 1, member c12 |
| Q00896 | MOUSE | IPI00123920 | alpha-1-antitrypsin 1-3 precursor |
| Q9DBF1 | MOUSE | IPI00890092 | alpha-aminoadipic semialdehyde dehydrogenase |
| Q99KR3 | MOUSE | IPI00116221 | beta-lactamase-like protein 2 |
| P24270 | MOUSE | IPI00312058 | catalase |
| P10605 | MOUSE | IPI00113517 | cathepsin b precursor |
| Q8BXK9 | MOUSE | IPI00226730 | chloride intracellular channel protein 5 |
| Q8BJ64 | MOUSE | IPI00273146 | choline dehydrogenase, mitochondrial precursor |
| P18760 | MOUSE | IPI00890117 | cofilin-1 |
| Q9WU84 | MOUSE | IPI00124103 | copper chaperone for superoxide dismutase |
| P19536 | MOUSE | IPI00785410 | cytochrome c oxidase subunit 5b, mitochondrial precursor |
| O08749 | MOUSE | IPI00874456 | dihydrolipoyl dehydrogenase, mitochondrial precursor |
| Q9QZD9 | MOUSE | IPI00269613 | eukaryotic translation initiation factor 3 subunit i |
| P63242 | MOUSE | IPI00108125 | eukaryotic translation initiation factor 5a-1 |
| P29391 | MOUSE | IPI00762203 | ferritin light chain 1 |
| Q9CQM9 | MOUSE | IPI00315550 | glutaredoxin-3 |
| Q64471 | MOUSE | IPI00554933 | glutathione s-transferase theta-1 |
| P51855 | MOUSE | IPI00127691 | glutathione synthetase |
| Q9R111 | MOUSE | IPI00469987 | guanine deaminase |
| Q7TSZ0 | MOUSE | IPI00880839 | heat shock protein 9 |
| P11499 | MOUSE | IPI00554929 | heat shock protein hsp 90-beta |
| Q9Z2X1 | MOUSE | IPI00226073 IPI00798511 | isoform 1 of heterogeneous nuclear ribonucleoprotein f |
| Q9D6J6 | MOUSE | IPI00169925 IPI00845716 | isoform 1 of nadh dehydrogenase [ubiquinone] flavoprotein 2, mitochondrial precursor |
| Q8R0M2 | MOUSE | IPI00279474 | isoform 2 of utp--glucose-1-phosphate uridylyltransferase |
| P14602 | MOUSE | IPI00128522 IPI00468068 IPI00623819 | isoform a of heat shock protein beta-1 |
| Q4FZE8 | MOUSE | IPI00880740 | major urinary protein 1 |
| P02762 | MOUSE | IPI00655227 | major urinary protein 6 precursor |
| Q9ERE7 | MOUSE | IPI00349285 | mesoderm development candidate 2 |
| P35700 | MOUSE | IPI00121788 | peroxiredoxin-1 |
| Q9DBM2 | MOUSE | IPI00554834 | peroxisomal bifunctional enzyme |
| P09411 | MOUSE | IPI00555069 | phosphoglycerate kinase 1 |
| Q9Z2M7 | MOUSE | IPI00131228 | phosphomannomutase 2 |
| Q80V68 | MOUSE | IPI00626741 | phytanoyl-coa dioxygenase domain-containing protein 1 |
| P49722 | MOUSE | IPI00890001 | proteasome subunit alpha type-2 |
| P99026 | MOUSE | IPI00129512 | proteasome subunit beta type-4 precursor |
| P70195 | MOUSE | IPI00136483 | proteasome subunit beta type-7 precursor |
| P09103 | MOUSE | IPI00133522 | protein disulfide-isomerase precursor |
| Q63836 | MOUSE | IPI00323816 | selenium-binding protein 2 |
| Q64442 | MOUSE | IPI00753038 | sorbitol dehydrogenase |
| Q9CQT1 | MOUSE | IPI00885509 | translation initiation factor eif-2b subunit alpha/beta/delta-like protein |
| O88342 | MOUSE | IPI00314748 | wd repeat-containing protein 1 |
| P35738 | RAT | IPI00115302 IPI00661338 | isoform 2 of 2-oxoisovalerate dehydrogenase subunit beta, mitochondrial precursor |
| Q2KII5 | BOVINE | n.f. |  |
| Q862E5 | BOVINE | n.f. |  |
| Q91Z81 | HAMSTER | n.f. |  |
| Q700Z7 | HUMAN | n.f. |  |
| Q9GLW6 | MACAQUE | n.f. |  |
| Q66HD0 | RAT | n.f. |  |

n.f. = IPI orthologue not found in MOUSE
